# Controlled Amine-Borane Dehydropolymerization Enabled by Mechanistic Insight Using the Ir(^t^Bu-POCOP)H_2_ Catalyst

**DOI:** 10.1021/jacs.5c21118

**Published:** 2026-07-07

**Authors:** Chloe M. Van Beek, M. Arif Sajjad, Joe C. Goodall, Catherine L. Lyall, John P. Lowe, Simon B. Duckett, J. Scott McIndoe, Charles Killeen, Guy C. Lloyd-Jones, Ulrich Hintermair, Stuart A. Macgregor, Richard E. Douthwaite, Andrew S. Weller

**Affiliations:** † Department of Chemistry, University of York, Heslington, York YO10 5DD, U.K.; ‡ EaStCHEM School of Chemistry, 7486 North Haugh, University of St Andrews, St Andrews KY16 9ST, U.K.; § Department of Chemistry, University of Bath, Claverton Down, BA2 7AY Bath, U.K.; ∥ Dynamic Reaction Monitoring Facility, University of Bath, Claverton Down, BA2 7AY Bath, U.K.; ⊥ Department of Chemistry, University of Victoria, 3800 Finnerty Rd, Victoria, BC V8P 5C2, Canada; # School of Chemistry, 3124 University of Edinburgh, Edinburgh, Scotland EH9 3FJ, U.K.

## Abstract

A detailed kinetic, mechanism, and *operando* speciation study on the controlled, catalytic, cascade-like dehydropolymerization of H_3_B·NMeH_2_ to form *N*-methyl polyaminoborane [H_2_BNMeH]_n_ (**Me-PAB**) using Ir­(^t^Bu-POCOP)­H_2_ as a precatalyst is reported. Catalyst speciation, as monitored using online FlowNMR, shows the formation of a borohydride complex Ir­(^t^Bu-POCOP)­(H)­(BH_4_) during an induction period, that rapidly speciates to Ir­(^t^Bu-POCOP)­H_4_ during productive turnover. Kinetic studies in the roles of NMeH_2_, trace water, and [H_3_B·NMeH_2_] on the induction period, productive catalysis and speciation reveal a complex set of processes that provide a framework for numerical and computational (DFT) modeling. Collectively these combine to support a mechanism for the dehydrogenation of H_3_B·NMeH_2_ to form the actual monomer, H_2_B=NMeH, that involves concerted B–H/N–H activation. By understanding the factors that control the maximum rate of dehydrogenation [ν_(max)_] and catalyst speciation, and deploying temperature variation and amine additives, a wide-range of **Me-PAB** molecular weights (*M*_n_ = 35,000–191,200 g·mol^–1^) can be achieved; in addition to low catalyst loadings (21 ppm), multigram scales (16 g) and water/air tolerance. The development of such systems which operate to selectively produce **Me-PAB**, using as-supplied substrates and simple catalysts, that also work on a scale useful for materials testing, promotes the wider exploitation of **Me-PAB** as a general preceramic precursor to *hex*-boron nitride.

## Graphical abstract

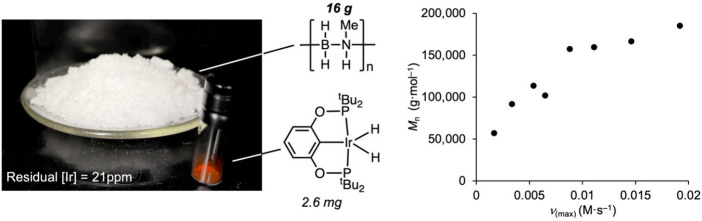

## 1 Introduction

Structured 1-D, 2-D and 3-D hexagonal boron nitride (*h*-BN) materials are technologically important ceramics.^1,2^ *h*-BN ceramics have the rare set of collective properties of being mechanically strong, chemically robust, oxidation resistant, wide-band gap insulators, and excellent thermal conductors. Examples of the uses of such materials include: *h*-BN fibers that have the potential for use in applications where carbon fiber fails,^2^ *h*-BN-derived catalysts,^3^ *h*-BN nanotubes,^4^ and porous *h*-BN materials for gas separation and storage.^5^ The polymer-derived precursor route is a particularly important technique for the manufacture of *h*-BN ceramic materials, as structure and function can be encoded into a shaped preceramic that is subsequently processed by curing, then annealing at high temperature (∼1400 °C).^1,6^ A notable example is the generation of shaped *h*-BN materials from polyborazylene, [B_3_N_3_H_4_]_n_, derived precursors, as demonstrated independently by Sneddon^6–8^ and Miele.^1,2,9^ However, the precursor to polyborazylene is borazine, which is a volatile, water-sensitive, flammable liquid. This is a challenging material to handle and make on scale; while polyborazylene itself is best made from the self-condensation of borazine under high pressure conditions,^7,10,11^ solution routes have been reported.^7^ Potentially safer, accessible, and cheaper *h*-BN main-group^12^ polymer preceramics are polyaminoboranes,^13^ e.g. [H_2_BNHR]_n_, which are B–N main-chain polymers isosteric with polyolefins.^14^ While the parent [H_2_BNH_2_]_n_ is an insoluble, poorly defined, material,^15,16^ *N*-methyl polyaminoborane, [H_2_BNMeH]_n_ **Me-PAB**, is an air-stable solid that is soluble in common solvents (e.g., THF), making it an attractive target for further processing, for example by electrospinning to make preceramic BN-fibers.^17,18^ Although stoichiometric routes are known,^19,20^ **Me-PAB** is best synthesized using metal-catalyzed routes.^18,21,22^ Here, the commercially available premonomer H_3_B·NMeH_2_ undergoes a cascade-like^23^ dehydropolymerization, Scheme 1, to first produce the transient^24^ aminoborane active monomer H_2_B=NMeH, from which head-to-tail B–N bond formation gives **Me-PAB**.^15,21,22,25^ H_2_ is the only byproduct. If these two processes are not geared well, however, unselective dehydrocoupling produces borazines, B–N cleavage products, oligomers or ill-defined, insoluble materials.^21^

**Scheme 1:**
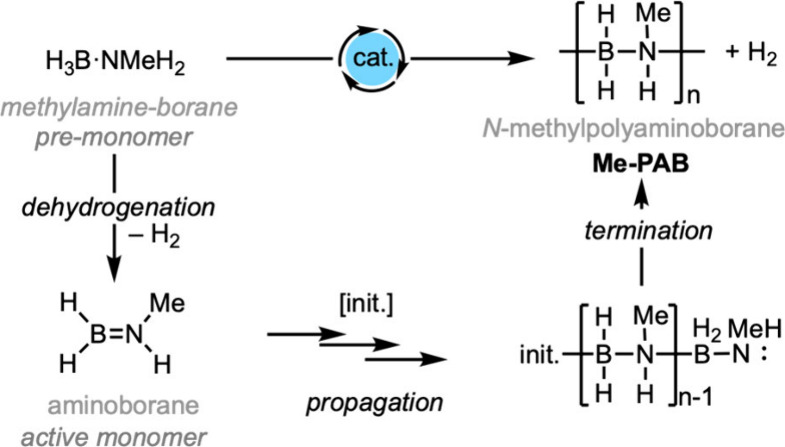
Dehydropolymerization of H_**3**_B·NMeH_**2**_

A controlled^26,27^ polymerization, where these dehydrogenation and chain-propagation processes are holistically coupled, would ideally lead to well-defined polymeric products, where the degree of polymerization can be systematically modified. Noncatalytic thermal decomposition of H_3_B·NMeH_2_ leads to amorphous, cross-linked, materials,^28^ or cyclic borazines,^29^ while low temperature stoichiometric routes lead to **Me-PAB** being formed less selectively.^19,20,24^

The dehydropolymerization of premonomer H_3_B·NMeH_2_ to afford **Me-PAB** was first reported independently by Manners and co-workers, and Goldberg, Heinekey and co-workers, using Brookhart’s^30^ Ir­(^t^Bu-POCOP)­H_2_ catalyst, **1** (^t^Bu-POCOP = κ^3^-2,6-(^t^Bu_2_PO)_2_C_6_H_3_), Chart 1.^15,25,31,32^ Detailed characterization of the −BN– product by Manners^25^ demonstrated the formation of polymeric material (0.3 mol % **1**, *M*_n_ = 55,000 g·mol^–1^, *Đ* = 2.9). This catalyst has since been used to generate **Me-PAB** for onward exploitation,^33–35^ as well as Ph-PAB,^36^ and PAB copolymers.^31^

**Chart 1:**
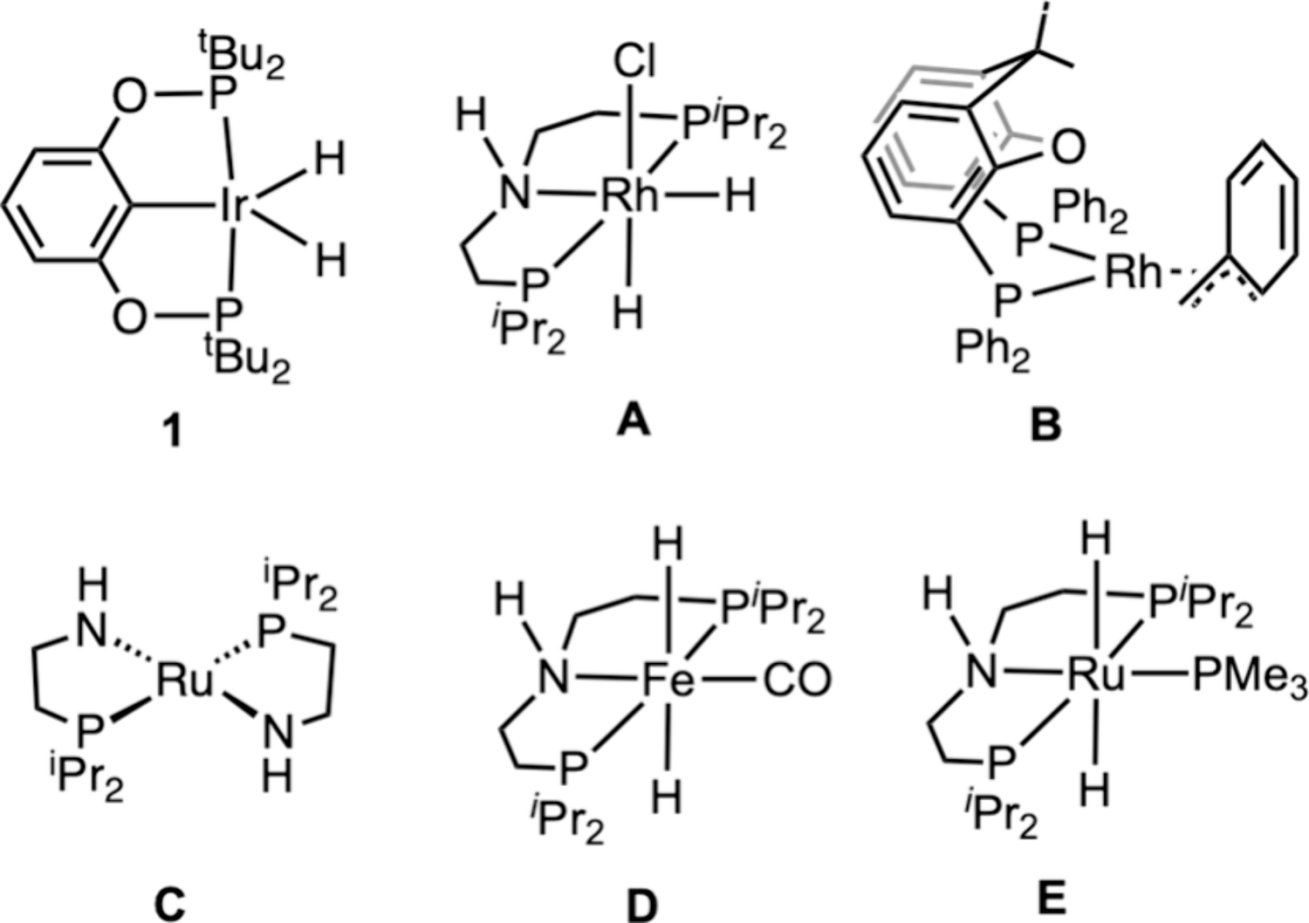
Selected Amine-borane Dehydropolymerization Catalysts

Computational studies using **1**/H_3_B·NH_3_ suggest a mechanism of concerted B–H/N–H transfer for the initial dehydrogenation to form free aminoborane. Complex **1**,^37^ or free amine,^20^ then acts as an initiator to promote subsequent head-to-tail B–N bond formation, through an end-chain *N*-lone pair on the growing polymer chain, Scheme 2. While both are relatively low energy processes, dehydrogenation has a higher calculated barrier (20.7 kcal·mol^–1^)^38^ than chain propagation (ΔG^‡^ < 10 kcal·mol^–1^),^20,37^ albeit the former was modeled using a truncated-phosphine model and only reported computed enthalpies of activation. Thus, while dehydrogenation to form the active monomer will likely be overall rate-determining, chain-propagation/termination will control selectivity. Experimental and computational studies on other catalyst systems that selectively dehydropolymerize amine-boranes, e.g. Chart 1 complexes **A–E**,^39–50^ support this mechanistic landscape.

**Scheme 2:**
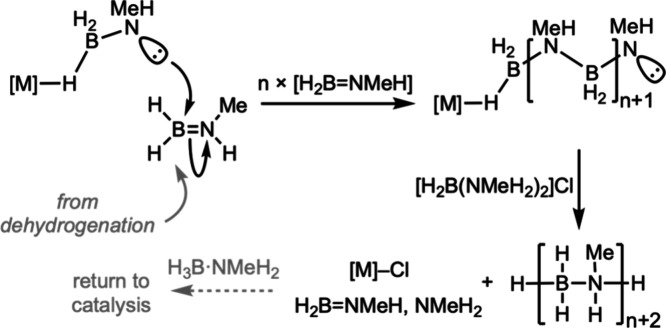
Proposed Propagation/Chain Transfer

Chain propagation occurs via a chain-growth polymerization^21,25,46,48,50^ – although step-growth mechanisms have been proposed for some systems^51,52^ that are similar to related phosphine-borane dehydrocoupling.^53^ Chain transfer, and thus a way to controllably lower molecular weight of the isolated **Me-PAB**, has been shown to occur on addition of boronium, [H_2_B­(NMeH_2_)_2_]^+^, in Rh-based systems, **A** and **B**.^45,48^ This is proposed to operate by protonation of the end-chain lone pair, Scheme 2. Despite these studies, the mechanistic details of how the catalyst system **1** promotes dehydropolymerization remains to be fully determined.

In this contribution we report a kinetic and mechanistic study on the dehydropolymerization of H_3_B·NMeH_2_ to form **Me-PAB**, using complex **1** as a precatalyst. By understanding the factors that control the rate of dehydrogenation and catalyst speciation, and deploying temperature variation and amine additives, we show that a wide-range of molecular weights (*M*_n_ = 35,000–191,200 g·mol^–1^) can be achieved, as well as very low catalyst loadings (21 ppm w/w), and multigram scales, Scheme 3. The development of such systems that operate to selectively produce **Me-PAB**, that are also water- and air-tolerant, use as-supplied substrates and simple catalysts, and work on a scale useful for materials testing, promotes the wider exploitation of **Me-PAB** as a general preceramic precursor to *h*-BN. We have recently reported that catalyst **A** can also be used to make **Me-PAB** of different molecular weights that can be processed by electrospinning to make precursor fibers to 1-D *h*-BN materials, and briefly reported that **1** is also useful in this regard.^17^ The ability to control polymer chain-length (and resulting chain-entanglement^54,55^ ) was central to the production of these ceramic fibers.

**Scheme 3:**
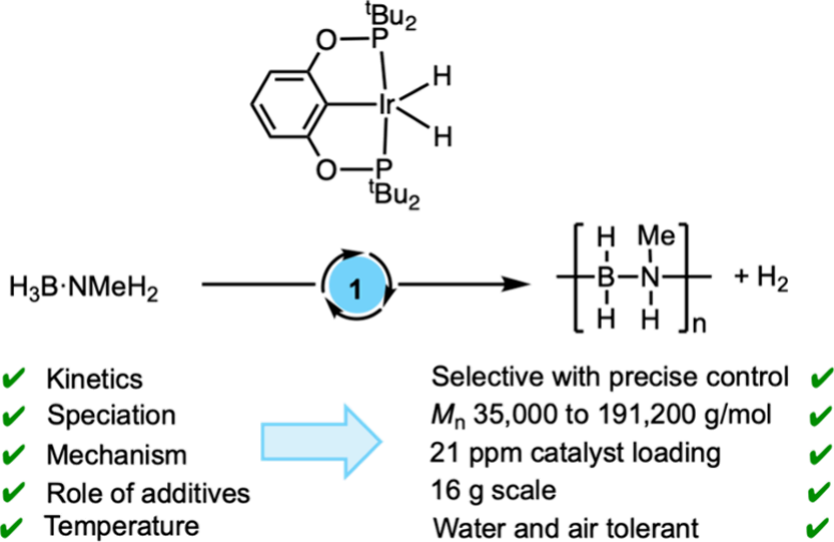
This Work

## 2 Results and Discussion

### 2.1 Baseline Dehydropolymerization Kinetics Using Homogeneous Catalyst 1: Induction Periods, Repeatability and a Classical Chain Growth Polymerization

In their 2008 communication, and follow-up full paper in 2010, Manners and co-workers reported the use of catalyst **1** at 0.3 mol % loading in THF (10 M H_3_B·NMeH_2_) that resulted in isolation of **Me-PAB** as an “off-white” solid in 60% yield.^15,25^ As measured by Gel Permeation Chromatography (GPC, relative to polystyrene standards), this polymer had a *M*_n_ = 55,200 g·mol^–1^ (*Đ* = 2.9). Detailed kinetics were not reported.^56^ A related study by Goldberg^32^ focused on H_2_ release using precatalyst **1** at 0.5 mol % loading (0.5 M H_3_B·NMeH_2_), but temporal profiles were not reported. Thus, in our preliminary mechanistic studies, the kinetics of H_2_ evolution as a proxy for *in situ* formation of H_2_B=NMeH were determined. Standard conditions of 0.1 mol % catalyst **1** (2 mM), and 2 M H_3_B·NMeH_2_ in THF (10 ppm residual H_2_O) were used. Reactions were performed on 112 mg scale, using recrystallized H_3_B·NMeH_2_, in a jacketed Schlenk tube equipped with a magnetic stirrer (400 rpm), that was temperature controlled to 20 ± 0.1 °C using an external circulating cryostat. This was connected to a eudiometer which measured H_2_ evolution volumetrically (Figure S2) under isobaric conditions. At the end of the reaction, as determined by cessation of H_2_ generation, an aliquot was analyzed by ^11^B NMR spectroscopy to determine conversion and selectivity (Figure S3). In all cases reactions went to completion (i.e., ∼1 equiv of H_2_ was formed) and showed >99% conversions to **Me-PAB** with >99% selectivity. In the ^11^B NMR spectrum **Me-PAB** is observed as a distinctive broad resonance at δ −6.7 (CDCl_3_, Figure S69).^15,25,45,46^ No other B-containing species were observed, e.g. *N*,*N*,*N*-trimethylborazine, δ 33.2.^57^ Precipitation of **Me-PAB** using pentane gave white solids in 55–75% isolated yield after drying under vacuum. The isolated polymer is stable as a solid under ambient conditions for over 1 year (^11^B NMR spectroscopy and GPC).

Using these conditions a repeatable and reliable temporal profile and degree of polymerization are achieved. Figure 1a shows exemplar H_2_ evolution traces from four separate runs using different batches of recrystallized H_3_B·NMeH_2_. Very similar GPC traces are obtained from the polymer that is isolated from each repeat experiment (*M*_n_ = 95,200–107,100 g·mol^–1^, *Đ* = 1.5), Figure 1b. The temporal reaction profile showed a variable induction period (100–200 s) during which time a small amount of gas is released (<5%), followed by the onset of an accelerating then decelerating phase of H_2_ evolution. Under these standard conditions, Figure 1a, the central region of the mildly sigmoidal temporal concentration profile reaches a maximum rate of H_2_-evolution, *v*_max_ = (6.4 ± 0.3) × 10^–3^ M s^–1^, based on [H_2_B=NMeH] equivalents,^58^ corresponding to a TOF_(max)_ ∼3 s^–1^. The productive phase of catalysis lasts ∼600 s. We,^45–47,49,59^ and others,^60–62^ have reported similar profiles previously, albeit with different time scales. Such induction periods can signal the evolution of a molecular precatalyst into a heterogeneous, or colloidal, active species.^63^ Addition of Hg_(l)_ (200 equiv) to catalysis during productive turnover (∼25% conversion) resulted in no diminution of activity, Figure 1c, and reaction mixtures were pale-yellow, clear, solutions throughout (Figure S7) with no visible precipitate – in contrast to systems where colloidal catalysts are suggested to operate.^59,62^ Addition of substoichiometric^63^ amounts of PMe_3_ did not significantly slow catalysis, while 5 equiv completely suppressed turnover, likely through formation of Ir­(^t^Bu-POCOP)­H_2_(PMe_3_), similar to the PPh_3_-analog reported by Findlater and co-workers.^64^ Collectively, these observations suggest a homogeneous, molecular, regime for dehydrogenation catalysis using **1**.

**Figure 1:**
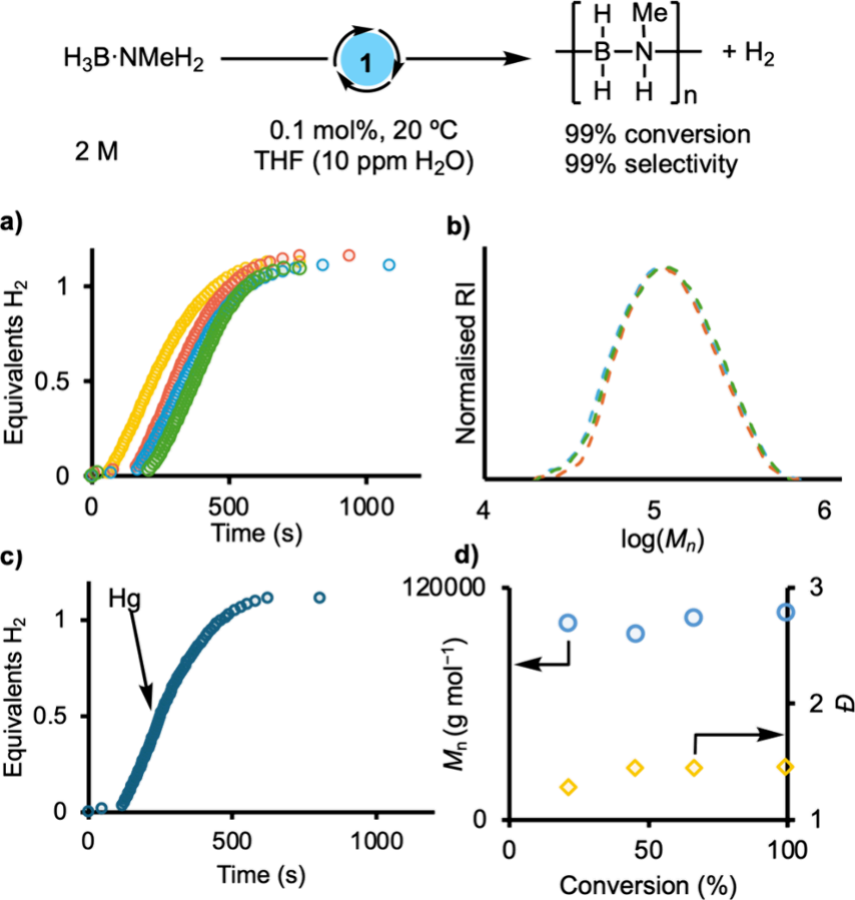
Benchmarking catalyst **1** in the dehydropolymerization of H_3_B·NMeH_2_. a) H_2_ evolution (eudiometer) from four independent catalyst runs. b) Overlaid GPC traces of isolated polymer from these separate experiments. c) Hg-drop test. d) Polymer growth kinetics, samples quenched with PMe_3_ (5 equiv). *M*_n_ and *Đ* measured by GPC. Conversion measured by ^11^B NMR spectroscopy.

A plot of conversion versus *M*_n_, Figure 1d, is characteristic of a non-living chain-growth polymerization, at pseudo steady-state.^21,25,46,48,50^ At low conversions of H_3_B·NMeH_2_, high *M*_n_ polymer is formed, which remains essentially constant throughout catalysis.^65^ Repeating with different batches of H_3_B·NMeH_2_ and **1** results in the same profile.

These studies benchmark the dehydrogenation and polymer growth kinetics of H_3_B·NMeH_2_ dehydropolymerization using catalyst **1**. This is characterized by being a homogeneous system that shows an induction period, and then relatively fast and stable turnover for a sustained period, to selectively form **Me-PAB** in a chain-growth process. With these fundamental observations established, the catalyst speciation in each phase of turnover was investigated.

### 2.2 Catalyst Speciation under *Operando* Conditions: A Ir­(^t^Bu-POCOP)­H_4_ Resting State, and the Temporal Evolution of Borohydride and Amine Complexes in the Catalytic Manifold

Given the rapid evolution of H_2_ at low catalyst loadings, following reaction progress and catalyst speciation in a sealed NMR tube under turnover conditions is challenging. Using the standard conditions, sampling aliquots from a eudiometric experiment run at 10 °C and rapidly cooling to −80 °C, to thermally quench the reaction, provided speciation by analysis of the resulting ^1^H and ^31^P­{^1^H} NMR spectra (h_8_-THF, 128 scans) at three time points. At the early stages of the reaction (∼120 s), just within the induction period, this analysis showed the consumption of **1** to form a mixture of the previously reported complexes: tetrahydride Ir­(^t^Bu-POCOP)­H_4_ **2** ^66–69^ [δ­(^1^H) −8.79, δ­(^31^P) 183] and dihydrideborane/borohydride Ir­(^t^Bu-POCOP)­H­(BH_4_) **3** ^70–72^ [δ­(^1^H) −5.55, −6.73, −20.13; δ­(^31^P) 170] in an approximate 60:40 ratio, Figure 2a. An unidentified species in low concentration is also observed at δ­(^31^P) 177.5 that correlates to a signal at δ­(^1^H) −10.5 in the ^1^H NMR spectrum. During, and at the end of, productive catalysis the system evolves to give complex **2**, with **3** no longer observed.

**Figure 2:**
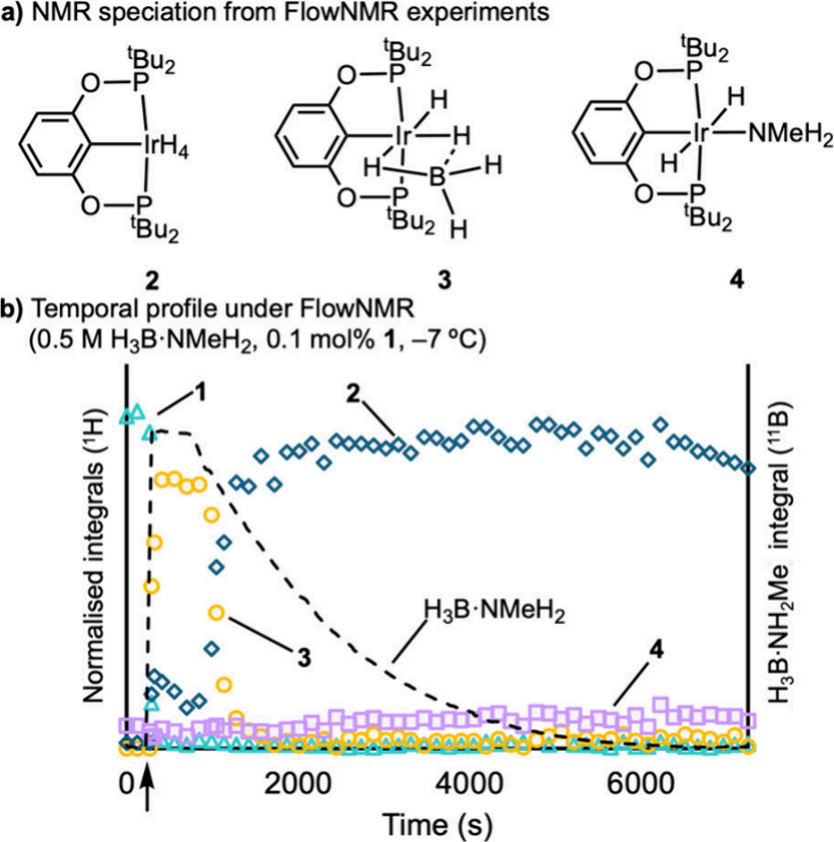
a) Organometallic complexes identified by FlowNMR experiments during catalysis. b) Temporal evolution from ^1^H, ^11^B and ^31^P­{^1^H} FlowNMR experiments. Dotted line represents [H_3_B·NMeH_2_] added to the system at 300 s, as measured by ^11^B NMR spectroscopy. Conditions: Normalized integrals from ^1^H NMR spectroscopy as referenced to an internal standard of 1,3,5-trimethoxybenzene. Cr­(tmhd)_3_ (10 mM) was added as a relaxation agent [tmhd = tris­(2,2,6,6-tetramethyl-3,5-heptanedionate)].

This sampling protocol, however, does not provide real-time analysis under *operando* conditions or high data-density. For a more detailed investigation we turned to multinuclear high resolution *online* FlowNMR spectroscopy^73^ using a closed-loop system that circulates the reaction solution from a sealed flask into a 500 MHz spectrometer equipped with a cryoprobe.^74^ The optimal conditions for analysis using this method were determined to be −7 °C, 0.5 M H_3_B·NMeH_2_ and 0.1 mol % **1** (see Supporting Information). ^1^H (selective excitation conditions), ^31^P­{^1^H} and ^11^B NMR spectra were collected in separate experimental runs using Cr­(tmhd)_3_ as relaxation agent.^75^ After baseline spectra of starting materials had been collected, H_3_B·NMeH_2_ was added after 300 s to the reaction solution containing **1** to start catalysis. Figure 2b shows the evolution of reaction progress from ^1^H and ^11^B FlowNMR monitoring (see Figures S59–62 for the stacked NMR spectra). This shows that on addition of premonomer H_3_B·NMeH_2_, complex **1** is immediately consumed to give dihydrideborane/borohydride **3** as the major component (∼80%) with tetrahydride **2** also observed.

While there is a small drop in [H_3_B·NMeH_2_] (<5%) over the next 450 s, this represents an induction period with respect to polymer formation. After this time, rapid consumption of H_3_B·NMeH_2_ occurs, concomitant with a step-change shift in speciation to give **2** as the major species. A small amount of a new species is observed (∼8%) that is characterized by signals at δ −9.7 and δ 169 in the ^1^H and ^31^P­{^1^H} NMR spectra respectively. As shown in the next section, this species is identified as the *trans*-dihydride amine complex Ir­(^t^Bu-POCOP)­H_2_(NMeH_2_), **4**.^76^ This overall speciation profile does not change significantly at the end of catalysis (7000 s), and a ^11^B NMR spectrum shows the selective formation of **Me-PAB**, confirmed by GPC analysis (*M*_n_ = 104,700 g·mol^–1^, *Đ* 1.5). During turnover the monomer H_2_B=NMeH^24^ is not observed [δ­(^11^Β) 37.1], consistent with rapid chain growth.

Using complex **2** as a precatalyst did not change the temporal profile for H_2_ evolution significantly, with a similar induction period (∼300 s) compared to using **1**, Figure 3a. In contrast using **3** as a precatalyst resulted in a much longer induction period (3500 s) before the onset of catalysis and the release of 1 equiv of H_2_. Addition of NMeH_2_ to **3** (5 equiv) reduced the induction period to ∼200 s, with the ensuing turnover at a similar rate to catalysis by **1**, *v*_max_ = 6.55(2) × 10^–3^ M·s^–1^. Added amine has been used to attenuate the formation of inactive borohydride complexes in the dehydrocoupling of H_3_B·NH_3_ using catalyst **D**,^42^ as well as removing induction periods for catalyst **B** ^48^ and related systems,^47,59,77^ by promoting the formation of the active catalyst.

**Figure 3:**
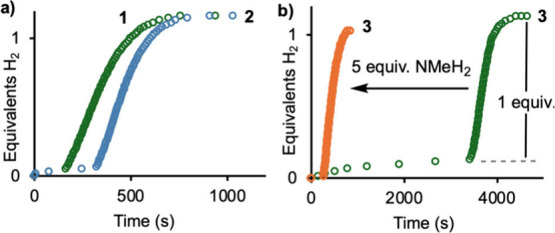
H_2_ evolution under standard conditions (a) using catalyst **1** and **2**; (b) **3** and **3** + 5 equiv of NMeH_2_.

These combined observations show that Ir­(^t^Bu-POCOP)­H_4_ **2** is the resting state during catalysis, and Ir­(^t^Bu-POCOP)­H­(BH_4_) **3** is the major species observed during the induction period, which must itself be formed from complex **1**. At the onset of dehydropolymerization, the switch from dormant **3** to **2** is relatively rapid and may be promoted by the buildup of free NMeH_2_.

### 2.3 Reactivity of Ir­(^t^Bu-POCOP)­H_4_, Ir­(^t^Bu-POCOP)­H_2_ and Ir­(^t^Bu-POCOP)­(H)­(BH_4_) with Dihydrogen, Amines and Water: Building a Model for On-Cycle, Off-Cycle and Induction Speciation

Brookhart^66^ and Wendt^67^ have described the reversible reaction of **1** with H_2_ to form **2**, with the latter reporting NMR-kinetic data from 2D-EXSY experiments and H/D exchange with D_2_. Addition of H_2_ to **1** results in the clean and complete conversion to **2**, while placing pale-yellow solutions of **2** under dynamic vacuum quickly restores (seconds) dark-red **1**, Scheme 4. Under the conditions of catalysis (eudiometric or FlowNMR), **2** will thus be formed quickly, starting from **1**.

**Scheme 4:**
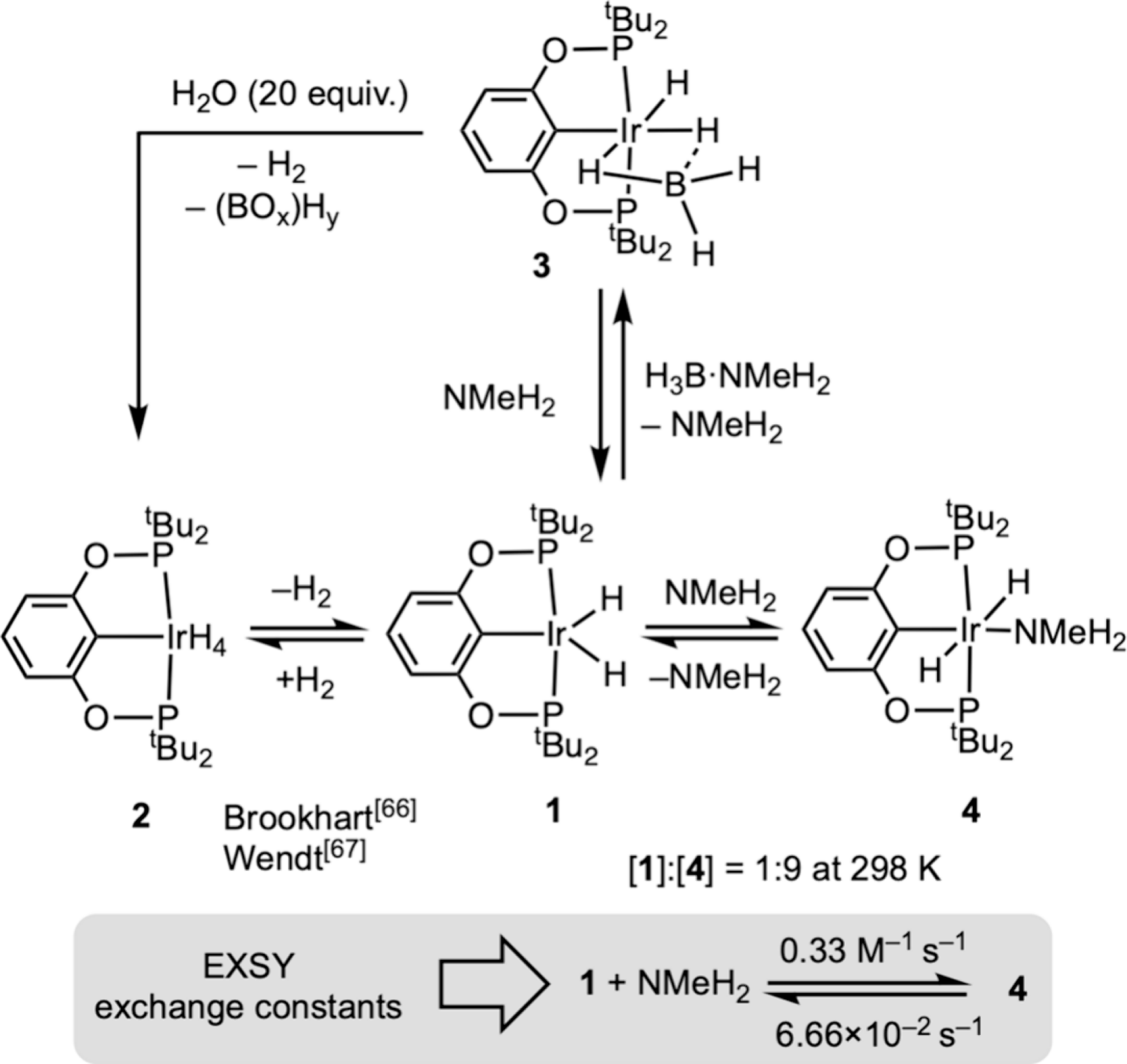
Reactivity Relationships between Complexes 1 to 4 (Solvent = *h*_*8*_/*d_8_*-THF)

Amine complex **4** can be formed by addition of excess NMeH_2_ (2 M in THF) to **1**. Complex **1** is reformed by the application of a dynamic vacuum, showing that the amine is labile.

Complex **4** is best synthesized by adding gaseous NMeH_2_ to **1** in d_8_-THF, allowing for full characterization *in situ* by ^1^H and ^31^P­{^1^H} NMR spectroscopy. A single-crystal X-ray diffraction study on material recrystallized from pentane at −80 °C with excess amine confirmed its structure (). While not located in the X-ray diffraction analysis, the hydrides are *trans*-disposed, identified by a characteristic chemical shift in the ^1^H NMR spectrum at δ −9.7 (2 H).^78^ EXSY experiments show that **1**/NMeH_2_ and **4** are in exchange on the NMR time scale in d_8_-THF, and provide an estimate for the rate constants for this process from consideration of the exchange constants, in the manner of Wendt,^67^ when **4** is dissolved in d_8_-THF. For the dissociation of amine, *k*_diss_ = 6.6 × 10^–2^ s^–1^ and ΔG^‡^ = 19.1 kcal·mol^–1^, in reasonable agreement with the 20.8 kcal·mol^–1^ calculated by DFT (Section 2.7). Addition of H_2_ to **4** reforms tetrahydride **2**, consistent with a rather balanced equilibrium, and supported by DFT calculations.

As the FlowNMR experiments show, addition of H_3_B·NMeH_2_ to red/orange complex **1** in THF resulted in the immediate (∼10 s) formation of pale yellow dihydrideborane/borohydride **3** as the main product, alongside some complex **2**, Figure 2. DFT studies (see Section 2.7) confirm that **3** arises from facile B–N bond cleavage in an unobserved σ-amine-borane complex, i.e. Ir­(^t^Bu-POCOP)­(H)_2_(H_3_B·NMeH_2_) **5**, liberating NMeH_2_ as coproduct. Similar B–N bond cleavage to form borohydride-like complexes has been reported from addition of H_3_B·NMe_3_ to Ru­(Me-PNP)­H_2_(H)_2_ [Me-PNP = κ^3^-(^t^Bu_2_PCH_2_CH_2_)_2_NMe].^79^ Pure, independently synthesized,^70^ **3** reacts with 2 equiv of NMeH_2_ to form a mixture of **1**, **2** and **4**, Scheme 4. This shift in speciation establishes the role of NMeH_2_ in significantly reducing the induction period when using **3** (Figure 3b), and the switch from **3** to **2** observed in the FlowNMR studies (Figure 2b). While the equilibrium **3**/NMeH_2_ ⇌ **5** is finely balanced (ΔG = +1.0 kcal·mol^–1^, Section 2.7), more detailed measurements are hampered by the onward dehydrocoupling of H_3_B·NMeH_2_, to form BN products, and **1**, **2** and **4**.

As NMeH_2_ is a likely promoter for the onset of dehydrogenation, its mode of formation is of considerable interest. While the dissociation of H_3_B·NMeH_2_ in THF is one possible route, as we have speculated on before,^45,48^ this reaction is slow^80^ and is thus not consistent with the acute respeciation observed, Figure 2. While the precise mechanism that generates the active catalyst is not currently known, some possible scenarios are suggested. *(i)* Complex **3** undergoes hydrolysis^81^ with trace H_2_O, likely from the H_3_B·NMeH_2_ starting material, to form borates/H_2_, releasing NMeH_2_ and regenerating complexes **1/2/4**.^82^ Similar processes have been noted for the hydrolysis of H_3_B·NH_3_ using Ir­(III)-NHC catalysts.^83^ Once sufficient free NMeH_2_ is generated the equilibrium between **3** and **1/2/4** shifts toward the latter (Scheme 4) and productive dehydropolymerization catalysis starts.^84^ *(ii)* Aminoborane, H_2_B=NMeH, is slowly formed during the induction period that can either undergo hydrolysis with trace H_2_O to form borates/H_2_ and NMeH_2_ ^85^ or sequester BH_3_ from **3** to form catalyst **1** and an aminodiborane^86^

### 2.4 Postinduction Period Dehydrogenation Kinetics: Determination of Order in Catalyst, H_3_B·NMeH_2_, and NMeH_2_, and Isotope Effects

With the catalyst speciation and likely role of amine in modifying the induction period established, an analysis of the effects of relative concentration of the various components was undertaken to provide kinetic data on the dehydrogenation process that is controlling polymerization. By systematic variation of the concentrations of **1**, H_3_B·NMeH_2_ and NMeH_2_, the effect of each component was determined by interrogation of the rate-maximum in H_2_ evolution experiments.^58^ Full details of determined rates and resulting variation in isolated **Me-PAB** molecular weights are given in Tables S1, S4 and S6. Consistent batches of recrystallized H_3_B·NMeH_2_ were used for each analysis, and repeats were run. Figure 4 details these analyses graphically.

**Figure 4:**
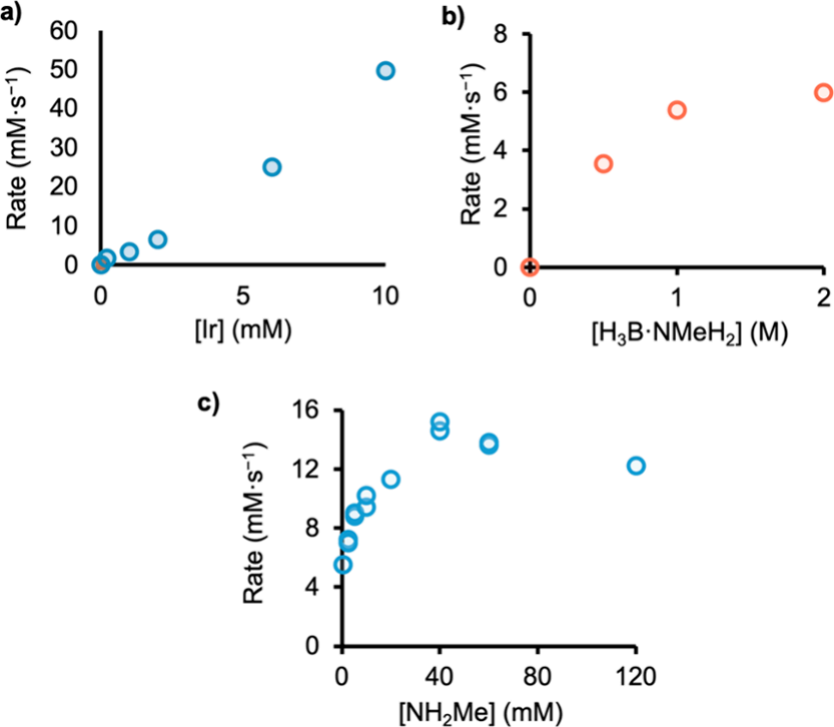
Variation of reaction components and maximum rate (mM s^–1^). Conditions (unless otherwise quoted): [**1**] = 2 mM, [H_3_B·NMeH_2_] = 2 M, 20 °C, THF (10 ppm of H_2_O). Induction periods discounted. a) Variation in [**1**]; b) Variation in [H_3_B·NMeH_2_]; c) Variation in [NMeH_2_].

The effect of [catalyst] was determined by varying [**1**]_TOTAL_ from 0.2 to 10 mM (i.e., 0.01 mol % to 0.5 mol %) while keeping [H_3_B·NMeH_2_] fixed at 2 M. Plotting [**1**]_TOTAL_ versus maximum rate resulted in an approximately first-order dependency on [**1**] (Figure 4a).^87^ Lower catalyst loadings resulted in longer induction periods; e.g., at 0.01 mol % there is a 4600 s induction period (Figure S11). Keeping [**1**] constant (2 mM) and varying [H_3_B·NMeH_2_] indicates that saturation kinetics are approached, Figure 4b. Variation of added NMeH_2_ from 0.5 mM to 120 mM (i.e., 0.25 to 60 equiv relative to [**1**]) had two consequences. First, induction periods shortened considerably with increasing [NMeH_2_], consistent with the activating role of amine with first-formed **3**. Second a plot of rate versus increasing [NMeH_2_] shows significant curvature, Figure 4c, with a promoting effect at lower concentrations and an inhibitory effect at higher concentrations, presumably by formation of **4**. Based on the maximum rate data, apparent activation barriers to turnover of ΔG^‡^_293_ 16(1) kcal·mol^–1^ and ΔS^‡^ −17(2) cal·K^–1^·mol^–1^ are estimated from an Eyring analysis (Figure S13).

The effect of isotopic substitution on the reaction profile was studied. There was a normal isotope effect on comparing H_3_B·NMeH_2_/H_3_B·NMeD_2_, *v*_max_(H/D) ∼ 1.7, and no appreciable effect observed for H_3_B·NMeH_2_/D_3_B·NMeH_2_, *v*_max_(H/D) ∼ 1. However, there were changes for the profiles of the induction periods (Figure S17). Such isotopologue-dependent profiles have been noted previously,^43,45^ and may indicate a change in the rate-determining steps, mechanisms, or off-cycle speciation (i.e., [Ir]_active_) that are finely balanced, and thus values must be interpreted with caution.

These kinetic data, alongside speciation and stoichiometric reactivity observations, suggest a relatively complex set of equilibria are associated with the catalytic dehydrogenation of H_3_B·NMeH_2_ using catalyst **1**. Combined they provide a framework to explore possible mechanistic scenarios.

### 2.5 Possible Mechanisms of Dehydrogenation: Concerted B–H/N–H Activation versus Amine Deprotonation via an Independently Isolated [Ir­(^t^Bu-POCOP)­H_3_]^−^ Intermediate

Two mechanistic possibilities that are consistent with the experimentally determined data have been considered, Figure 5. Both operate via initial, reversible, coordination of H_3_B·NMeH_2_ to catalyst **1** to form σ-complex **5**, Scheme 5.^41,46,59,77,88^ The mechanisms are then differentiated by the activation processes that follow. Mechanism A invokes inner-sphere B–H/N–H activation (**TS-F**), as originally suggested by Paul and Musgrave,^38^ to form **2** and free aminoborane. Mechanism B produces **2**/aminoborane via an outer-sphere base-promoted hydride transfer to first form the anionic trihydride, [Ir­(^t^Bu-POCOP)­H_3_]­[NMeH_3_], **[6]­[NMeH**_**3**_ **]**, that reprotonates to give **2** and NMeH_2_. Related “proton-rebound” mechanisms have been proposed to occur in other amine-borane dehydrogenation processes,^46,77,89^ as well Si–Cl,^90^ Si–H^91^ and H_2_ ^92^ bond activation processes. Brookhart has reported the synthesis of trihydride [Ir­(^t^Bu-POCOP)­H_3_]Na by addition of NaH to **1**,^66^ while Cantat reported that [Ir­(^t^Bu-POCOP)­H_3_]­[^iPr^VBH] is formed using Verkade's’s base (^iPr^VB) with **1**/H_2_.^90^

**Scheme 5:**
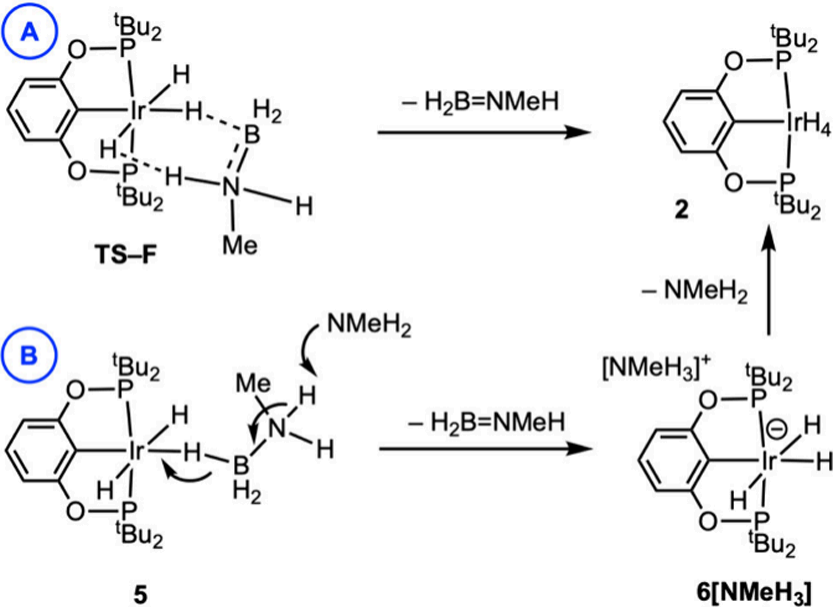
Key Differences between Mechanism A and B

**Figure 5:**
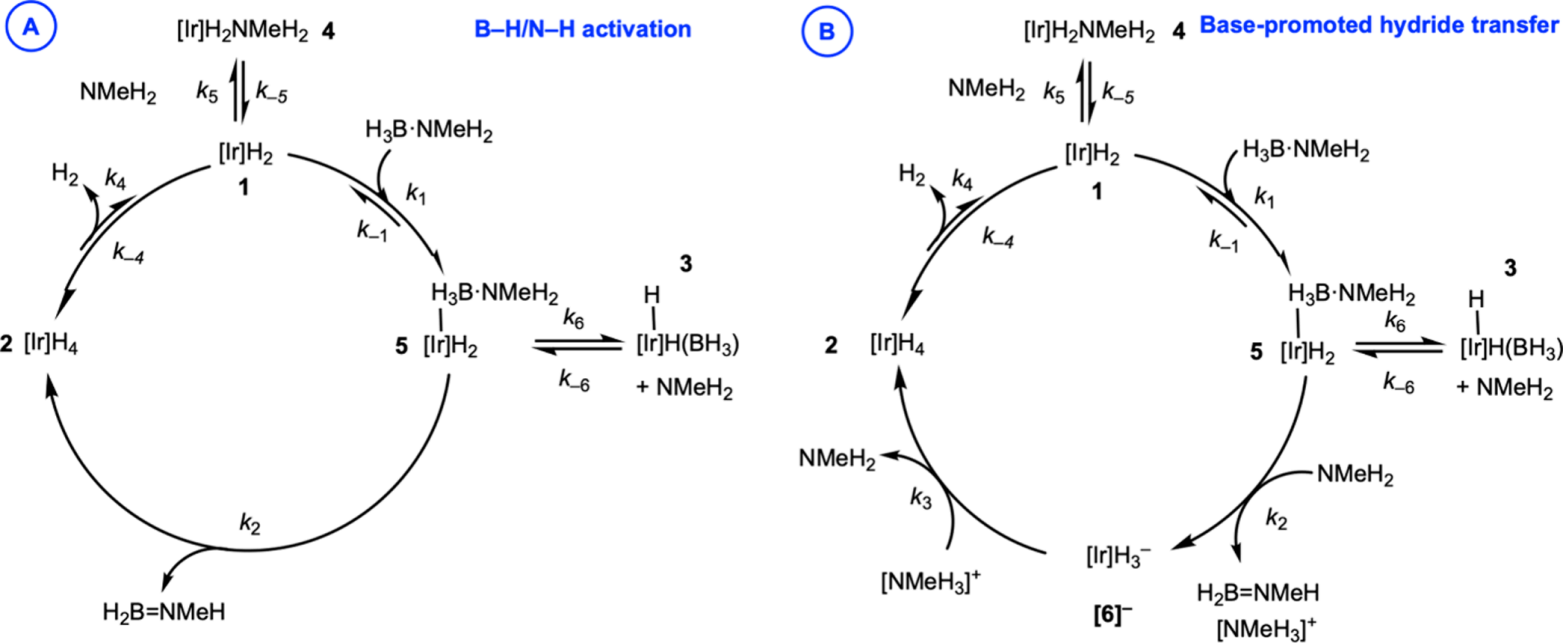
Proposed dehydrogenation mechanisms. (A) Mechanism A: B–H/N–H transfer. (B) Mechanism B: Base-promoted hydride transfer. Note: for ease of comparison with Mechanism B, *k*_2_ connects directly to *K*_4_, i.e. *k*_4_/*k*_–4_ in Mechanism A.

In both mechanisms, A and B, subsequent H_2_ loss^66,67^ from **2** regenerates **1**. Additional roles for amine are provided by the reversible formation of **4**, which removes the catalyst off-cycle, and the reaction with dormant **3** to bring the catalyst onto cycle.

To probe the potential role of anionic trihydride [**6**]^−^ (Mechanism B, Figure 5) in catalysis, **[6]­[Na­(18-crown-6)­(THF)**_**2**_ **]** was independently synthesized as a colorless solid in 62% yield,^66^ and characterized using single crystal X-ray diffraction and NMR spectroscopy. The former analysis was of sufficient quality [*R*(2σ) = 4.26 %, *R*(int) = 3.39 %] to allow for the location of the three hydride ligands, Figure 6. There are no close (<2.7 Å ^93,94^ ) Ir–H···H–C_(crown)_ interactions in the solid-state. However, in the solution ^1^H NMR spectrum (*d_8_*-THF) there is a significant difference in the chemical shifts of the hydride signals in **[6]­[Na­(18-crown-6)­(THF)**_**2**_ **]** [δ −11.58 (1H), δ −13.22 (2H)] compared with **[6]­Na­(THF)**_**x**_ ^66^ [δ −13.35 (1H), δ −13.55 (2H)], that may reflect nonclassical Ir–H···H–C_(crown)_ dihydrogen bonding, or direct Ir–H···Na^+^ interactions respectively.^95^ Support for the former comes from a 1-D selective ROESY experiment that showed correlations between both of the Ir–H signals and the C–H groups on the crown ether [δ 3.56], while comparison of ^23^Na chemical shifts with [Na­(18-crown-6)­(THF)_2_]­[BAr^F^_4_] Ar^F^ = 3,5-(CF_3_)_2_C_6_H_3_] shows a small but significant difference [δ −15 vs δ −16, respectively, Figure S49]. Analysis by ESI-MS (negative mode) shows the expected isotopologue distribution.

**Figure 6:**
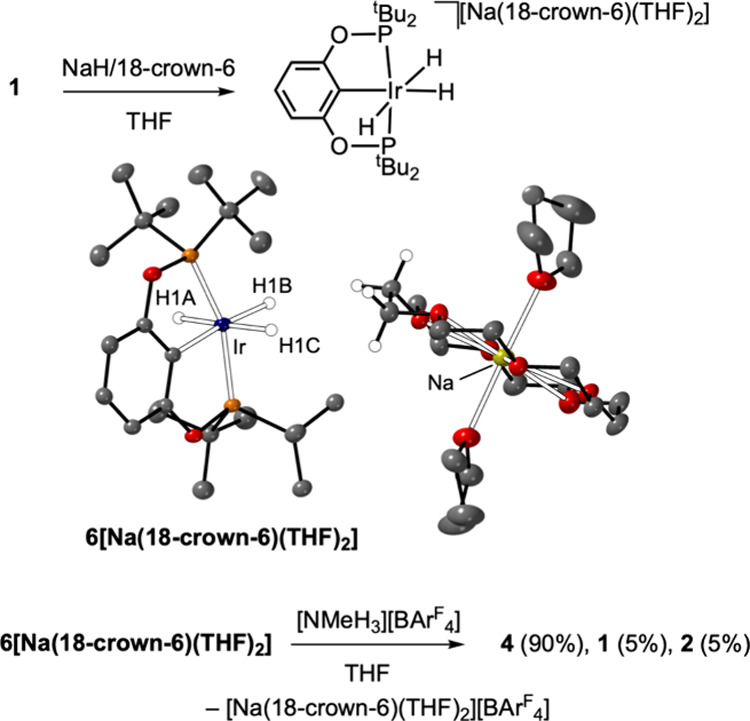
Synthesis and reactivity of **[6]­[Na­(18-crown-6)­(THF)**_**2**_] and single crystal X-ray structure (50% displacement ellipsoids, selected H-atoms shown). See for full details.

Addition of 1 equiv of [NMeH_3_]­[BAr^F^_4_] to **[6]­[Na­(18-crown-6)­(THF)**_**2**_ **]** resulted in the immediate (on time of analysis by ^1^H NMR spectroscopy) formation of amine complex **4** (90%), alongside **1** and **2**. A likely sequence of events is protonation to form **2** and NMeH_2_ and then rapid equilibration to a mixture dominated by **4** (Scheme 4). Consistent with these stoichiometric experiments, **[6]­[Na­(18-crown-6)­(THF)**_**2**_ **]**/[NMeH_3_]­[BAr^F^_4_] is an effective catalyst system for dehydropolymerization and operates without an induction period (Figure S21).

Interestingly, the rate of dehydrogenation is much faster with **[6]­[Na­(18-crown-6)­(THF)**_**2**_ **]**/[NMeH_3_]­[BAr^F^_4_] than when using **1** with 1 equiv of [NMeH_3_]­[BAr^F^_4_]: *v*_max_ = 19.2(3) × 10^–3^ M·s^–1^ versus 5.7(3) × 10^–3^ M·s^–1^ respectively. This is also considerably faster than when using **1**/NMeH_2_ (*v*_max_ = 7.20(5) × 10^–3^ M·s^–1^, ). While the promoting effect of crown ethers, or group 1 salts, in organometallic catalysis has been discussed,^96–99^ and the solution NMR data for **[6]­[Na­(18-crown-6)­(THF)**_**2**_ **]** may support a close ion-pairing, the precise role of any such interactions in promoting the dehydropolymerization catalysis remains unclear.

### 2.6 Simulated Reaction Kinetics and Catalyst Speciation

While **[6]­[Na­(18-crown-6)­(THF)**_**2**_ **]**/[NMeH_3_]­[BAr^F^_4_] is an effective precatalyst system, this does not discriminate between its active participation in turnover (i.e., Mechanism B) or by precatalyst conscription into Mechanism A by forming **4**. We thus turned to numerical methods modeling of these two scenarios.^100^ The two pathways, as diagramed in Figure 5, were simulated using eight independent sets of starting conditions that were holistically and simultaneously modeled against the corresponding experimental data, time-shifted to remove induction periods: [**1**]_TOTAL_ (0.5–2.0 mM), [H_3_B·NMeH_2_] (0.5–2.0 M) and NMeH_2_ (1.0–20 mM). Where available, experimentally determined rate constants were used, i.e. **2** ⇌ **1** + H_2_ (*k*_4_/*k*_–4_, measured in d_8_-toluene)^67^ and **1** + NMeH_2_ ⇌ **4** (*k*_5_/*k*_–5_) to reduce parametrization. The very slow^80^ self-dissociation of H_3_B·NMeH_2_ was included, as discussed previously in other models.^48^ The solution phase H_2_ concentration was limited to 0.04 M, to reflect the isobaric conditions of eudiometric H_2_ evolution. N–H/B–H activation in Mechanism A was truncated to a single step in the model. Figure 7a shows the fits of simulated to experimental data for the measured evolution of H_2_, as expressed in equivalents of H_2_B=NMeH. The kinetics of both mechanisms can be satisfactorily simulated, and capture the post induction period temporal profile well.

**Figure 7:**
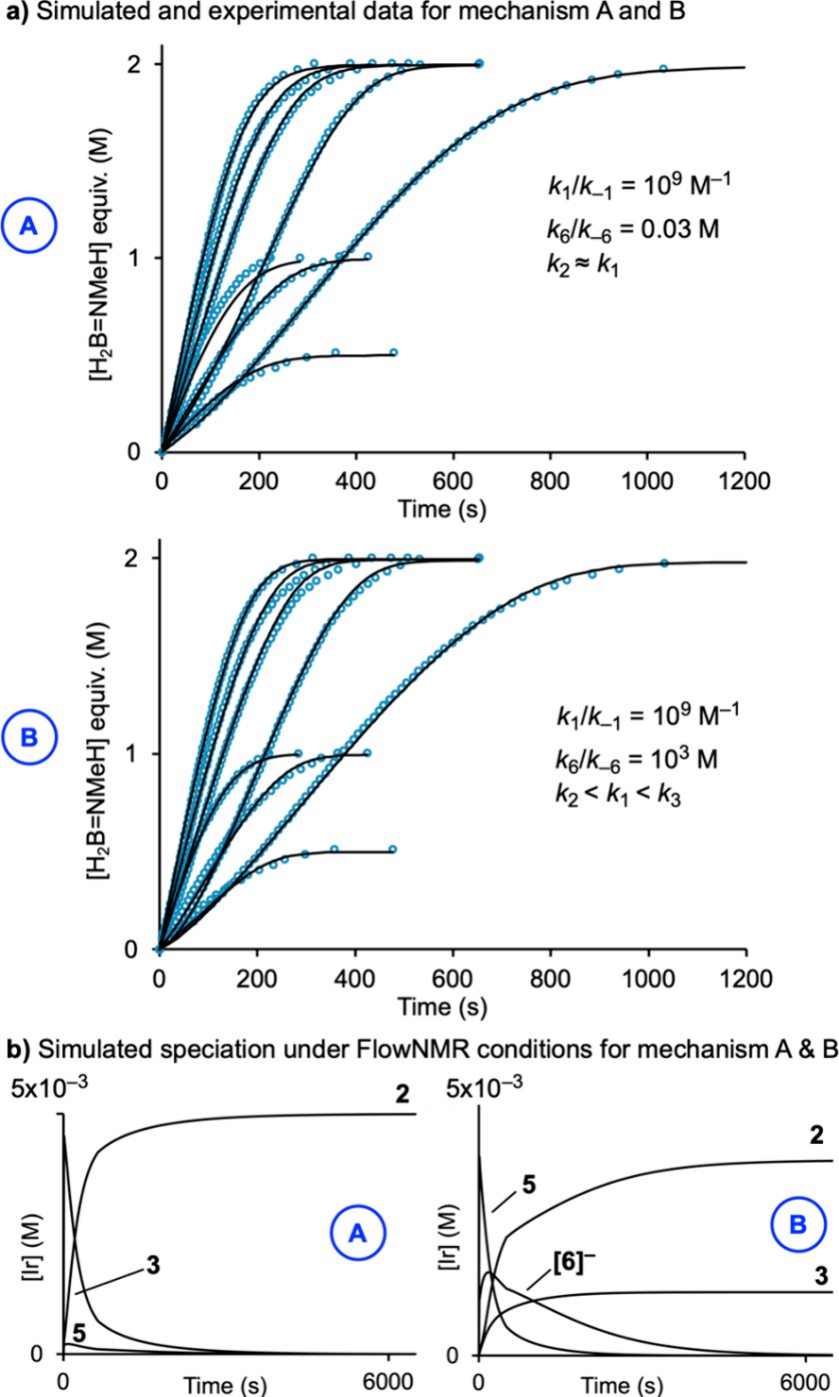
a) Concentration of [H_2_B=NMeH] as a proxy for H_2_ evolution versus time for eight different starting concentrations of **1**, H_3_B·NMeH_2_ and NMeH_2_. [H_2_] limited to 0.04 M. See Supporting Information for full details. Open circles = experimental data; solid lines = holistically simulated data derived for the two catalytic manifolds A and B. Data is time-shifted to remove induction periods. b) Simulated species versus time-shifted plots for mechanism A and B under FlowNMR conditions: [**1**] = 0.5 mM, [H_3_B·NMeH_2_] = 0.5 M, [NMeH_2_] = 0.5 mM, [H_2_]_dissolved_ limited to 0.2M.

While the simulation of H_2_ evolution data, and other kinetic and reactivity observations, does not allow for the discrimination between the two mechanistic pathways, consideration of the catalyst speciation does provide a distinction when compared to the experimentally collected data from the FlowNMR experiments, i.e. Figure 2b. Time-course plots for catalyst speciation were generated using the rate constants determined from the two models, but under the conditions used for the FlowNMR experiments ([**1**] 0.1 mol %, [H_3_B·NMeH_2_] 0.5 M). By manually iterating the limiting concentration of solution phase H_2_, which plays an inhibitory role in turnover [**2** ⇌ **1** + H_2_ (*k*_4_/*k*_–4_)], a satisfactory profile for H_3_B·NMeH_2_ consumption was achieved using a concentration 5-fold greater than estimated for eudiometric simulations. This reflects the hydrostatic backpressure generated by 4 mL/min flow through 12 m of 0.8 mm ID tubing under the FlowNMR conditions.^74^ Using these parameters, catalyst speciation was simulated, Figure 7b. Mechanism A satisfactorily captures the experimentally determined speciation to **2** as the resting state and no other significant species observed. Under these constraints, Mechanism B presents a more complex speciation that is not observed under any conditions, where **5** and **[6]** ^**–**^ have significant concentrations early in turnover, and evolve to a mixture of **2** and **3**.

### 2.7 Computational Studies

DFT calculations have been employed to model the formation of off-cycle species **3** and **4**, and to compare H_3_B·NMeH_2_ dehydrogenation via Mechanisms A (concerted B–H/N–H transfer) and B (base-promoted hydride transfer).

For the off-cycle speciation, Figure 8a shows **1** readily adds H_3_B·NMeH_2_ to give the *trans*-dihydride adduct, **5**, at +1.3 kcal·mol^–1^; the *cis* isomer is 5.3 kcal·mol^–1^ higher in energy. B–N bond cleavage via **TS­(5–3)** then gives **3** at +0.3 kcal·mol^–1^ with an overall barrier (relative to **1**) of 16.5 kcal·mol^–1^. In comparison, B–N bond dissociation in free H_3_B**·**NH_2_Me has a computed free energy of +30.5 kcal·mol^–1^ while BH_3_ dissociation from **3** is endergonic by 30.3 kcal·mol^–1^_._ Thus, an initial equilibrium can be established that accesses **3** and free NH_2_Me from **1** and H_3_B**·**NH_2_Me. The addition of NH_2_Me to **1** involves **TS­(1–4)** at 18.0 kcal·mol^–1^ and forms **4** at −2.8 kcal·mol^–1^, consistent with this species becoming formed postcatalysis. Dissociation of BH_3_ from **3** is unfavorable in the absence of NMeH_2_, consistent with the observed induction periods and promoting effects of amine observed.

**Figure 8:**
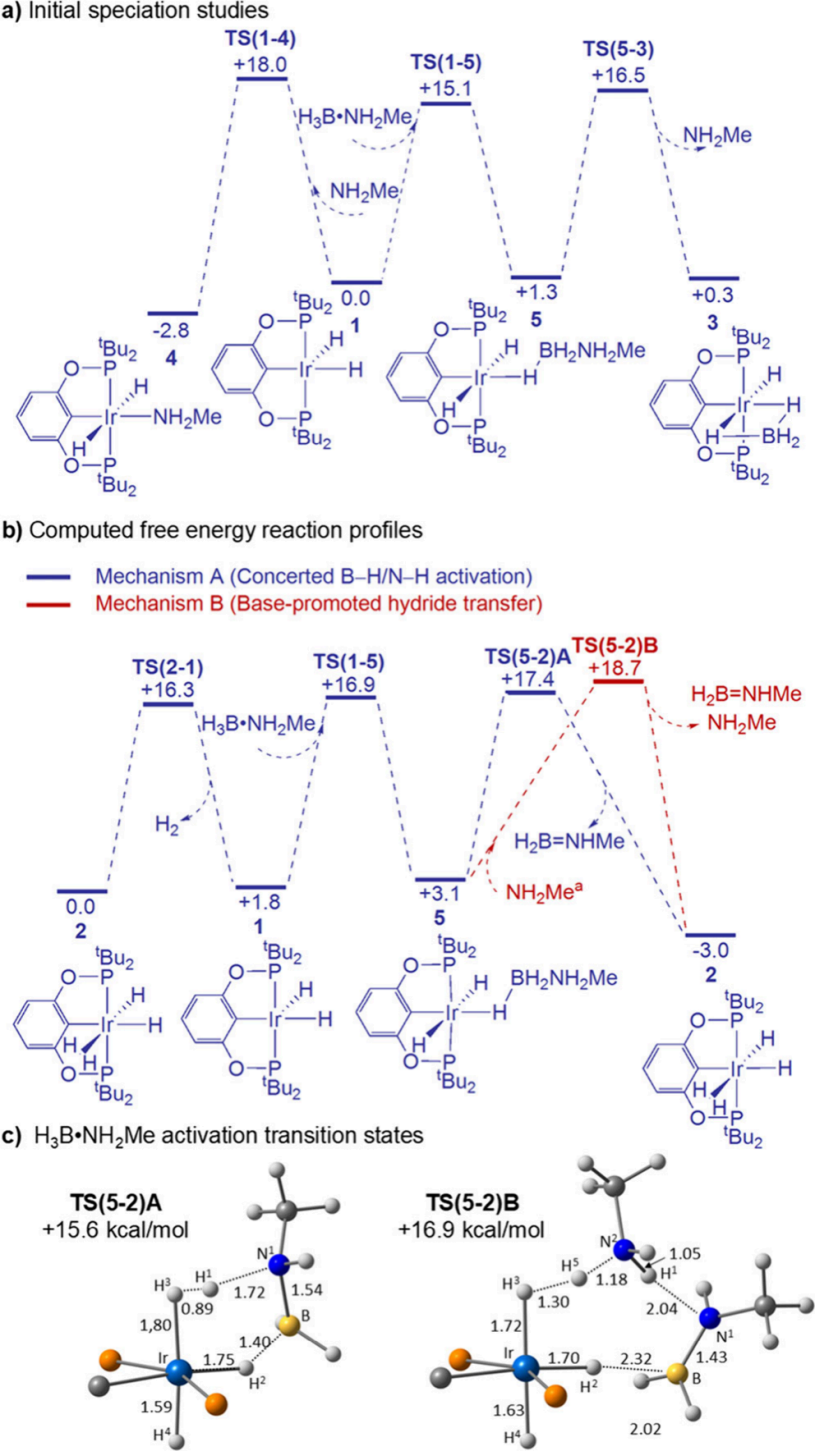
Computed free energy profiles (kcal/mol) for a) interconversion between **1**, **3** and **4**. b) Mechanisms A and B with profiles starting from catalytic resting state, **2** reset to 0.0 kcal·mol^–1^. c) Computed H_3_B·NMeH_2_ activation transition states (with selected distances in Å; only the P and C_aryl_ atoms of the POCOP ligand are shown for clarity). Level of theory: PBE0-D3BJ­(def2-tzvp, THF)//BP86/SDD (Ir, P with polarization on P); 6–31G** other atoms). ^a^An outer-sphere NH_2_Me adduct is formed at +4.3 kcal·mol^–1^ but is omitted for clarity: see Supporting Information for full details.

Figure 8b compares computed pathways for Mechanisms A and B, starting from **2**, the catalytic resting state determined experimentally. Complex **2** is computed to be most stable as the dihydrogen dihydride tautomer, although the tetrahydride form is only 0.7 kcal/mol higher in energy and the two can readily interconvert with a minimal barrier as noted previously (see Figure S79).^67,69^ H_2_ loss from **2** proceeds directly via **TS­(2–1)** to form **1** with a barrier of 16.3 kcal·mol^–1^, a similar value to that determined experimentally by Wendt and co-workers in *d* ^*_8_*^-toluene solution (ΔG^‡^ = 15.6 ± 0.5 kcal·mol^–1^).^67^ Reversible addition of H_3_B·NMeH_2_ to **1**, to form σ-adduct **5**, competes with regeneration of the tetrahydride **2** by addition of H_2_. Catalyst turnover is induced by dehydrogenation of the σ-adduct, **5**, to regenerate the dihydrogen dihydride resting state, **2**, and release the amino-borane product. The overall H_3_B·NH_2_Me dehydrogenation process is thermodynamically favorable (ΔG = −3.0 kcal·mol^–1^). Mechanisms A and B diverge at the stage of dehydrogenation of **5**. In mechanism A, concerted unimolecular B–H/N–H activation proceeds via **TS­(5–2)­A**. The process is asynchronous, with N–H activation (via protonation of H^3^) more advanced (N···H^1^ = 1.72 Å; H^1^···H^3^ = 0.89 Å) than B–H bond cleavage (B···H^2^ = 1.40 Å; Ir···H^2^ = 1.75 Å, Figure 8c). In Mechanism B, NH_2_Me assists the H_3_B·NMeH_2_ N^1^–H^1^ deprotonation and concomitantly induces B^1^–H^2^ cleavage. Several variants of this process were defined, and the most accessible via **TS­(5–2)­B** features a [NH_3_Me]^+^ moiety acting as a proton shuttle between N^1^ and H^3^, with stabilizing dihydrogen (H^3^···H^5^ = 1.30 Å) and H-bonding (H^1^···N^1^ = 2.04 Å) interactions. This cyclic arrangement is significant as alternative structures where NH_2_Me is placed *anti* with respect to Ir are much higher in energy (see Figure S85). In **TS­(5–2)­B** both the N^1^–H^1^ and B–H^2^ distances are significantly elongated (2.04 Å and 2.32 Å respectively) and a short, product-like B–N^1^ distance (1.43 Å) is computed in the aminoborane fragment.

1 $$\begin{array}{c} \mathrm{TOF},\;s^{-1}\approx \frac{1}{a+\frac{b+c\left[H_{2}\right]}{\left[H_{3}\mathrm{BNMe}H_{2}\right]}}\\ a=\frac{1}{k_{5,2}}+\frac{1}{k_{2,1}};\\ b=\frac{1}{K_{1,5}k_{5,2}}+\frac{1}{k_{1,5}};\\ c=\frac{1}{K_{2,1}K_{1,5}k_{5,2}}+\frac{1}{K_{2,1}k_{1,5}} \end{array}$$

2 $$\begin{array}{c} \mathrm{TOF}\approx \frac{4.1}{1+\frac{0.38}{\left[H_{3}{\mathrm{BNMeH}}_{2}\right]}},\\ \mathrm{when}\;\left[H_{2}\right]=0.04\;M \end{array}$$

The computed free energy profile for turnover, Figure 8b, connects the catalyst resting state **2** via sequential, concentration-dependent, equilibria to an irreversible dehydrogenation of the σ-adduct (**2** ↔ **1** ↔ **5** → **2**). Analysis of the relative barriers for dehydrogenation (**5** → **2**) shows that **TS­(5–2)­A** will be kinetically dominant over **TS­(5–2)­B** under all of the experimental conditions ([NMeH_2_] ≤ 120 mM, Figure 4c). While the observed isotope effects reflect the overall reaction manifold, they are consistent with an asynchronous transition state for mechanism A, in which N–H activation is preceded by B–H precomplexation to Ir, that accounts for the N–D KIE being observed.^101,102^

With the simplification to turnover solely by mechanism A, the computed three-step sequence (**2** ↔ **1** ↔ **5** → **2**), Figure 8b, can be compared with the empirical kinetic data by application of a steady-state approximation, eq 1, see the Supporting Information Section 6.2. The relationship shows that the concentrations of both H_2_ and H_3_B·NMeH_2_ impact the catalyst turnover frequency (TOF). Equation 1 can be further simplified, eq 2, by setting the solution phase concentration of H_2_ to 40 mM, the maximum solubility in THF at room temperature and pressure.^103^ Under this regime, catalysis is on the cusp of saturation kinetics, Figure 9, with half of the maximum rate, TOF_*max*_ ≈ 4.1 s^–1^, attained when [H_3_B·NMeH_2_] = 0.38 M. Under these conditions, the phenomenological barrier to turnover is approximately 16.7 kcal·mol^–1^, in agreement with that estimated experimentally, 16(1) kcal·mol^–1^. The three-step sequence from resting state to turnover (**2** ↔ **1** ↔ **5** → **2**) precludes a nuanced interpretation of the experimentally determined activation parameters.

**Figure 9:**
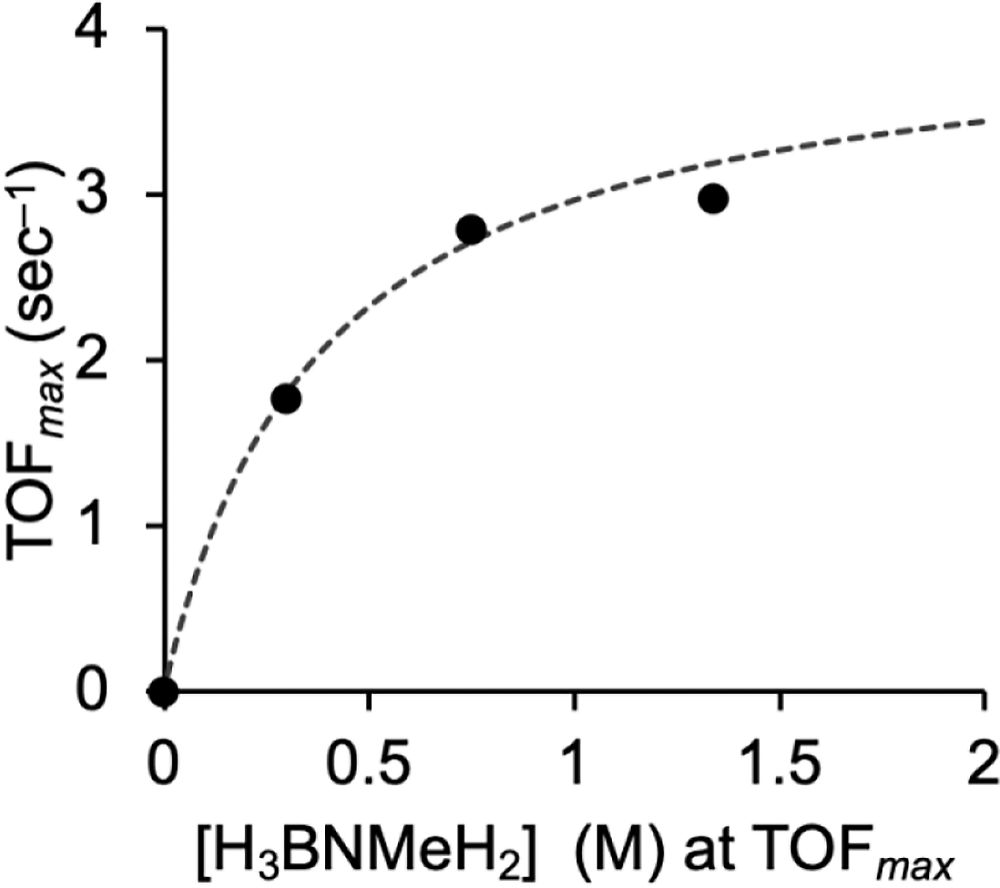
Measured (solid-points) and calculated TO*F*_max_ (dashed line, using eq 2) for different [H_3_B·NMeH_2_] at TOF_max_, as taken from data used in Figure 4b.

Calculations on Mechanism B also confirm the facile access to the catalytic cycle upon addition of [NMeH_3_]­[BAr^F^_4_] to **[6]­[Na­(18-crown-6)­(THF)**_**2**_ **]**; no local minimum corresponding to **[6]­[NMeH**_**3**_ **]** could be located, but rather H^+^ transfer to form **2** and NH_2_Me occurs spontaneously.^104^ Comparing the reactivity of H_3_B·NMeH_2_ adduct **5** shows B–N activation to form **3** (**TS­(5–3)** at +16.5 kcal·mol^–1^) is less accessible than B–H/N–H activation to form **2** and amino-borane (**TS­(5–2)­A** at +15.6 kcal·mol^–1^). While these relative barriers could imply that no induction period should be observed, the computational and kinetic models only consider turnover during productive catalysis. It is plausible that trace water (or other impurities) would bias both the kinetics and thermodynamics associated with the formation of **3** during induction, and have less of an influence during turnover. With this caveat, the overall picture presented by the calculations is coherent with the experimental speciation and kinetic studies observed during catalysis, and point to a mechanism that operates by concerted B–H/N–H activation.

This proposed mechanism is related to the profile put forward by Musgrave and Paul in their 2007 study, although one point of difference here is that the reaction proceeds through the *trans*-dihydride H_3_B·NMeH_2_ adduct **2**. With the present model, concerted B–H/N–H activation at the *cis*-isomer involves a higher energy transition state at +19.9 kcal·mol^–1^. Full details of these and other alternative pathways, as well as functional testing, are provided in the Supporting Information.

### 2.8 Control of Degree of Polymerization Using Catalyst Loading, Amine and Temperature To Give Me-PAB between *M*_n_ 57k–213k g·mol^–1^

The preceding kinetic and speciation data were collected to probe the dehydrogenation of H_3_B·NMeH_2_, using a wide range of initial conditions where the concentrations of catalyst, amine, and H_3_B·NMeH_2_ were all varied. In all cases >99% selectivity and conversions to **Me-PAB** were measured, and polymer was isolated in yields of 47–74%. Analysis by GPC (Table 1, entries 1–8) and comparison with the corresponding *v*_max_ for the dehydrogenation showed a positive correlation between rate of turnover and *M*_n_, Figure 10. The proposed^20,37^ **Me-PAB** chain growth mechanism is related to classical anionic and radical polymerizations,^105^ for which the degree of polymerization (D.P.) depends on the rate of propagation/rate of termination, i.e. D.P. ∝ *R*_(prop)_/*R*_(term)_. The observation that faster turnover results in longer polymer is consistent with the initial dehydrogenation of H_3_B·NMeH_2_ to give the active monomer H_2_B=NMeH being rate-limiting compared to chain-growth. Therefore, faster dehydrogenation leads to higher degrees of polymerization, assuming termination and initiation events are affected proportionally. We recently commented on such a relationship using catalyst **C**, Chart 1.^49^

**Figure 10:**
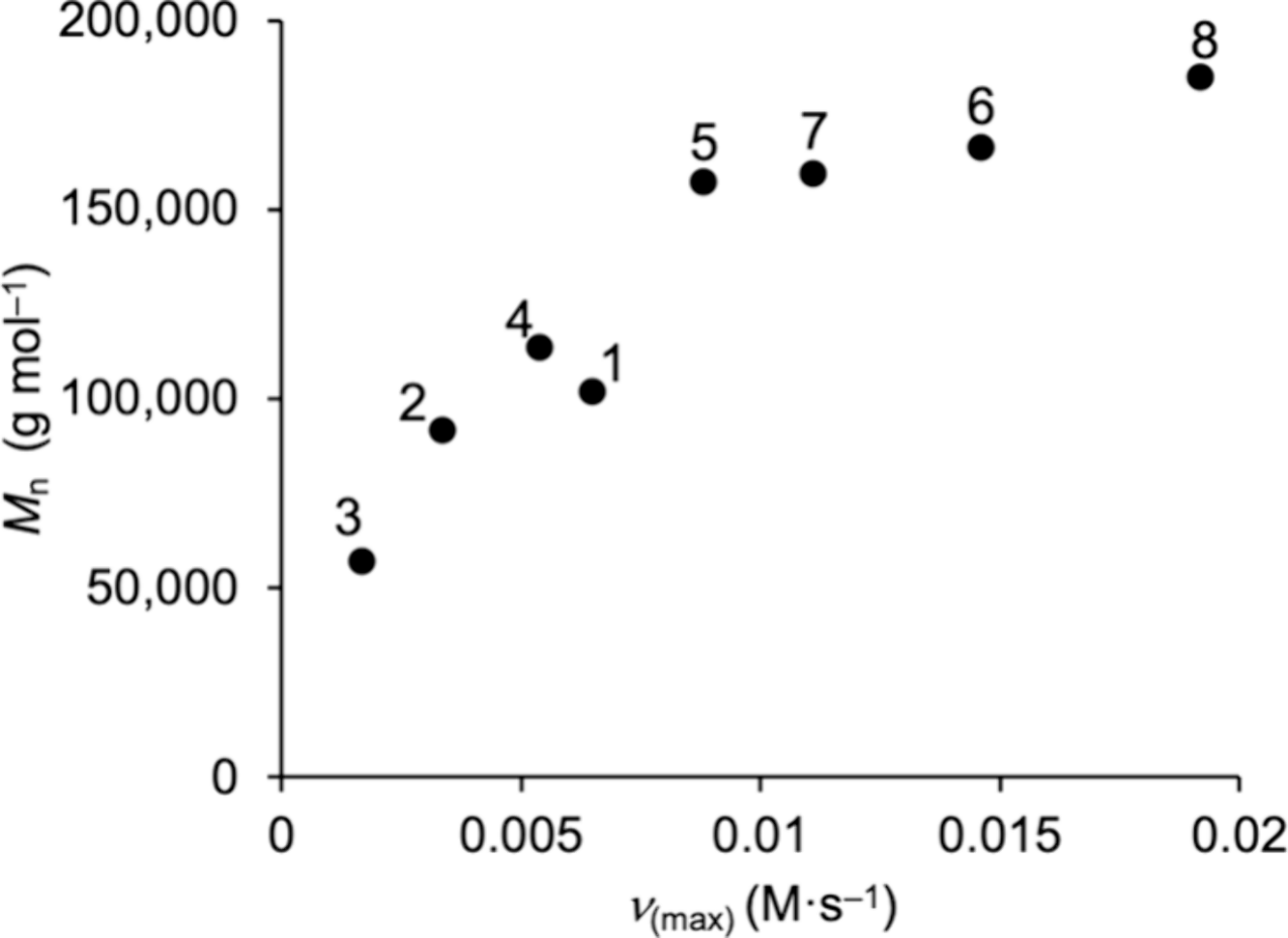
Relationship between *M*_n_ and maximum rate of turnover, *v*_max_, at 20 °C, under the conditions shown in Table 1.

**Table 1:** Experimental Conditions for Dehydropolymerization in THF under Eudiometric Conditions, Maximum Rate, *v*_max_, and Degree of Polymerization, *M*_n_

Entry	Catalyst	[Ir]_TOTAL_(mM) [mol %]	[H_3_B·NMeH_2_]^a^ (M)	[NMeH_2_]^b^ (mM)	Temp (°C)	*v*_max_ (× 10^3^ M·s^–1^)	*M*_n_ (g·mol^–1^)^c^
1	**1**	2.0 [0.1]	2.0	–	20	6.5^e^	102,000^e^
2	**1**	1.0 [0.05]	2.0	–	20	3.4	91,700
3	**1**	0.2 [0.01]	2.0	–	20	1.7	57,300
4	**1**	2.0 [0.2]	1.0	–	20	5.4	113,700
5	**1**	2.0 [0.1]	2.0	5.0	20	8.8	157,300
6	**1**	2.0 [0.1]	2.0	40.0	20	14.6	166,600
7	**1**	2.0 [0.2]	1.0	10.0	20	11.1	159,800
8	**[6]** ^**–**^ ^d^	2.0 [0.1]	2.0	–	20	19.2	185,200
9	**1**	2.0 [0.1]	2.0	–	10	3.60	124,400
10	**1**	2.0 [0.1]	2.0	–	0	1.68	166,100
11	**1**	2.0 [0.1]	2.0	–	–10	0.7	191,200

a Reactions on a 0.112 g scale using recrystallized H_3_B·NMeH_2_.

b Added as a 2 M solution in THF, conversion and selectivity >99% (^11^B NMR spectroscopy).

c Measured relative to polystyrene standards, monomodal distributions, *Đ* = 1.3 to 1.6.s

d **[6]­[Na­(18-crown-6)­(THF)]**/[NMeH_3_]­[BAr^F^_4_].**2**

e Average of four repeat measurements, see the text.

Further support for the essential characteristics of a classical chain-growth process comes from the variation in degree of polymerization with temperature. Anionic chain-growth polymerizations often have a higher barrier to termination compared with propagation, and thus lower temperatures favor the latter.^65^ Reduction of the dehydropolymerization reaction temperature from 20 to 0 to −10 °C, entries 9–11 Table 1, results in increasingly higher degrees of polymerization. Thus reactant and catalyst concentrations, amine-additive, and temperature can all be used to control the rate of monomer generation/termination, and in turn the degree of polymerization, Table 1.

While the precise influence of catalyst identity/loading, amine and other additives (e.g., [Na­(18-crown-6)]^+^) on the *polymerization* process are yet to be fully delineated in this complex system, the ability to control the degree of polymerization simply through consideration of dehydrogenation rate, *v*_max_, additives and temperature is of significant practical value. This leads to a range of isolated polymer molecular weights, *M*_n_ = 57,300 to 191,200 g·mol^–1^, as shown by overlaid GPC traces in Figure 11.

**Figure 11:**
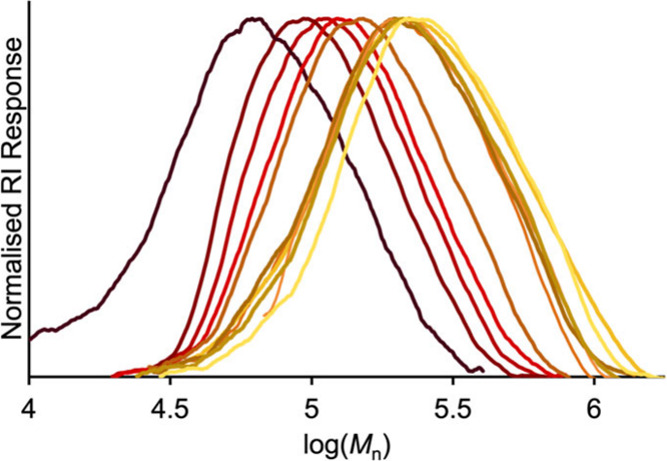
Overlaid GPC traces of **Me-PAB** from Table 1.

We have recently reported an equivalent, but wholly empirical, approach that focuses on Rh-based system **A** (Chart 1) to generate polymers of molecular weight suitable for electrospinning **Me-PAB** fibers that are precursors for h-BN 1-D materials (*M*_n_ range from 73,700 to 128,300 g·mol^–1^).^17^

Thermal Gravimetric Analysis (TGA) was used to analyze polymer samples ranging from *M*_n_ = 54,700 to 191,200 g·mol^–1^. These showed 50% mass loss events (*T*_decomp_) occurred in a narrow range (178–185 °C), resulting in low ceramic yields of <6.5% (500 °C), similar to previously reported **Me-PAB** materials.^25,45,52^ We have recently shown that curing **Me-PAB** at 100 °C (vacuum) results in cross-linking that significantly increases the ceramic yield to up to 63% (1400 °C).^17^ All the polymeric materials were formed as amorphous powders (PXRD).

### 2.9 Polymer Analysis Using ESI-MS

In their original report of the synthesis of **Me-PAB** using catalyst **1**, Manners and co-workers reported that a detailed analysis by mass spectrometry showed two polymer distributions were formed: [H­(NMeHBH_2_)_n_NMeH_2_]^+^ and [H­(NMeHBH_2_)_n_]^+^.^25^ We have also reported very similar data for deuterated **Me-PAB**.^52^

Analysis of the wide range of **Me-PAB** generated (Table 1) using ElectroSpray Ionization Mass Spectrometry (ESI-MS, positive mode, CH_2_Cl_2_ solvent) revealed essentially the same distribution, with degrees of polymerization ∼70 [*M*_n_ ∼ 3000 g·mol^–1^], irrespective of the molecular weights determined by GPC, Figure 12a, as also reported by Manners.^25^ MS/MS experiments on [H­(NMeHBH_2_)_18_NMeH_2_]^+^, Figure 12b, show initial loss of NMeH_2_ (31.04 Da) is followed by sequential BH_2_NMeH loss (43.06 Da). For [H­(NMeHBH_2_)_n_]^+^ (Figure S77) only sequential BH_2_NMeH loss is observed. These results come with the caveat that they may reflect the conditions of analysis and not the full compositional identity of the isolated **Me-PAB** (e.g., in comparison with GPC data), as previously noted.^21,25^

**Figure 12:**
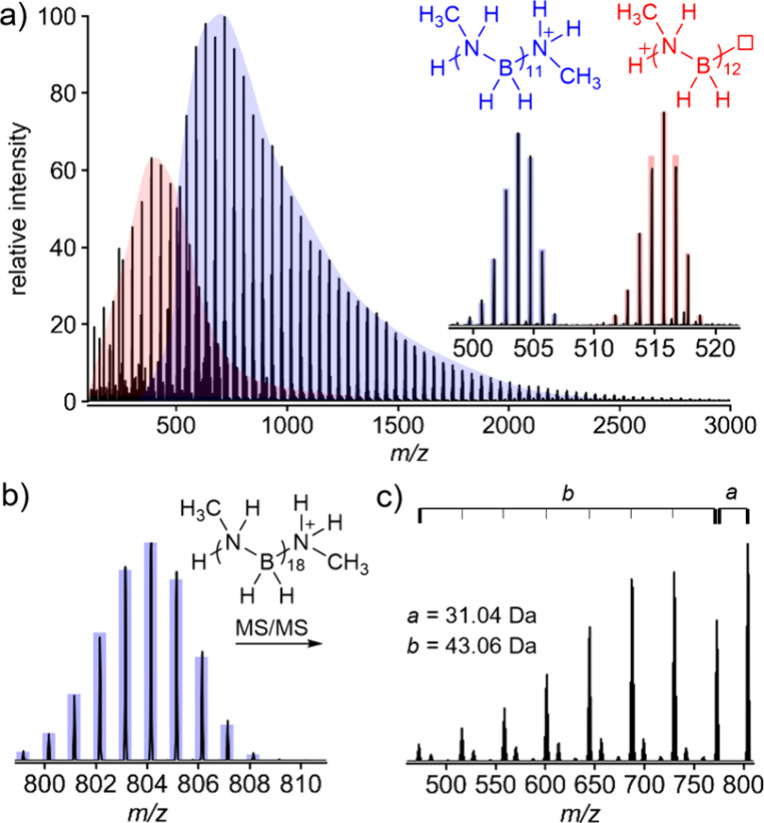
a) ESI-MS (positive mode, CH_2_Cl_2_) of a representative sample of **Me-PAB** produced using **1**. b) ESI-MS/MS of [H­(NMeHBH_2_)_18_NMeH_2_]^+^.

The N–H end group present in both analytes suggests a protonation event terminates the end of the growing polymer chain, as has been proposed for decreased molecular weight when using boronium chain transfer agents.^45,48^ How the polymer chain is released from the initiating metal center is currently opaque to experiment. The observed distributions from ESI-MS may suggest free NMeH_2_ displaces the metal hydride, or B–N bond scission occurs to form **3** (under H_2_ conditions) that is then conscripted back into catalysis. Amine may also sequester free H_3_B·THF that could conceivably terminate the polymer, but as resulting H_3_B­(NMeHBH_2_)_n_NMeH_2_ would be overall neutral with no basic sites, it would not be revealed by ESI-MS analysis.

### 2.10 Low (21 ppm) Catalyst Loadings Using As-Supplied H_3_B·NMeH_2_. A Practical System for the Production of *N*-Methylpolyaminoborane on Scale, That Is Water and Air Tolerant

The applicability of this system for the synthesis of **Me-PAB** under more practical conditions has been explored. Use of commercial unpurified H_3_B·NMeH_2_ under standard conditions (0.1 mol %) resulted in the selective formation of **Me-PAB** that was indistinguishable, by ^11^B NMR spectroscopy and GPC (*M*_n_ = 100,000 g·mol^–1^, *Đ* = 1.4), from that obtained using recrystallized starting materials. Catalyst loading can be reduced to 0.001 mol % using as-supplied H_3_B·NMeH_2_ on a 20 g scale using a reaction flask equipped with an overhead mechanical stirrer. For ease of addition, the majority of the H_3_B·NMeH_2_ was added dropwise over 3 h to a THF solution of **1** (2.6 mg). To avoid prohibitively long induction periods at this very low catalyst loading we have found that an additional promoter of [NMeH_3_]Cl (5 equiv to [Ir]_TOTAL_) initiates catalysis with no significant induction period, as effectively as excess NMeH_2_ (see Supporting Information for a full discussion). The reaction starts essentially immediately (visual gas evolution) and is complete after 2 days of stirring (as monitored by periodic sampling by ^11^B NMR spectroscopy) to selectively form **Me-PAB**, isolated as 16 g of white free-flowing solid in 80% yield, Figure 13. However, the polymer chain length is considerably shorter than formed under standard conditions (*M*_n_ = 35,000 g·mol^–1^, *Đ* = 1.9), which may reflect mass-transport^106,107^ effects in the reaction vessel.^108^ Nevertheless, this catalyst loading is an order of magnitude lower than previously reported using catalyst **A** (0.01 mol %) on a similar scale,^17,45^ and results in a very low residual metal content in the isolated **Me-PAB** (21 ppm w/w, compared with 1002 ppm w/w using **1** at 0.1 mol %, as measured by ICP-OES). This initial low catalyst loading also reduces the cost of catalyst to ∼£0.02 per gram of **Me-PAB** (see Table S16). While lower residual metal loadings in isolated **Me-PAB** have been reported previously, this required postpolymerization cleanup and considerable product loss.^59^

**Figure 13:**
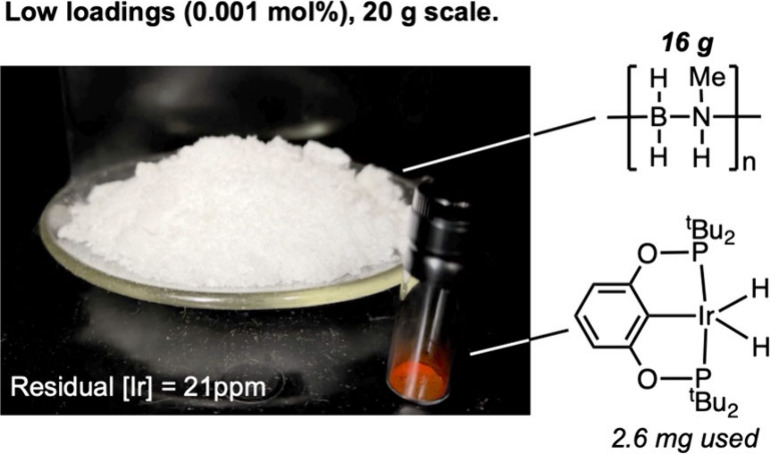
**Me-PAB** isolated from 0.001 mol % catalyst loading (i.e., using 2.6 mg of **1** and 20 g of H_3_B·NMeH_2_).

Finally, addition of catalyst **1** (0.05 mol %, 5 equiv. NMeH_2_) to a vial open to air resulted in 98% selective dehydropolymerization to give **Me-PAB** (*M*_n_ = 103,250 g·mol^–1^, *Đ* = 1.5, Figure S71), although an additional sharp signal observed at ∼δ­(^11^Β) 2 may reflect a small amount of cross-linking (i.e., “BN_4_”)^17,43,46^ or borates.^109^ This demonstrates air-tolerance and, when combined with the formation of **Me-PAB** using 1500 ppm of added H_2_O (vide infra), provides a practical system for the synthesis of **Me-PAB**.

## 3 Conclusions

We describe here a simple-to-use and efficient catalytic system for the controlled synthesis, on scale, of *N*-methyl polyaminoboranes over a wide range of polymer molecular weights. As well as being remarkably tolerant to water and air, the ability to control the degree of polymerization through simple changes to process conditions (temperature, added amine, catalyst loading) leads to a practical system where fine-tuning of the final product is possible. When combined with our previously disclosed Rh-,^45^ Ru-,^49^ and Co-^104^ based systems, this leads to a suite of catalysts and conditions that allow the practical deployment of dehydropolymerization methods to make polyaminoboranes. Such straightforward catalytic methods and processes will be important if these BN-polymeric materials are to be used for the manufacture of BN-ceramic materials.^17^ Finally, the powerful combination of *in situ* speciation, kinetics, simulation, and DFT modeling provides holistic mechanistic insight into a complex catalytic system that was first reported 15 years ago^25^ and under the right conditions produces a main-group BN polymeric material with remarkable selectively and fidelity.

## Supplementary Material





## Notes

Computed geometries as a combined file (XYZ)
Full experimental details, including synthesis and characterization of new complexes, speciation data, kinetics analysis, DFT computation (PDF)
