# Supplementary material for: Controlled Amine-Borane Dehydropolymerization Enabled by Mechanistic Insight Using the Ir(tBu-POCOP)H2 Catalyst
Source: J Am Chem Soc. 2026 Jul 7;148(28):29669–83. doi: 10.1021/jacs.5c21118 (PMC13397567; doi:10.1021/jacs.5c21118)
Supplement: Supplementary file 2 [file ja5c21118_si_002.pdf]

Supporting Information

# Controlled Amine-Borane Dehydropolymerization Enabled by Mechanistic Insight Using the Ir($^t$Bu-POCOP)$H_2$ Catalyst

Chloe M. Van Beek,[a] M. Arif Sajjad,[b] Joe C. Goodall,[a] Catherine L. Lyall,[c,d] John P. Lowe,[c,d] Simon B. Duckett,[a] J. Scott McIndoe,[e] Charles Killeen,[e] Guy C. Lloyd-Jones,*[f] Ulrich Hintermair,*[c,d] Stuart A. Macgregor,*[b] Richard E. Douthwaite,*[a] and Andrew S. Weller*[a]

[a] Department of Chemistry, University of York, Heslington, York YO31 1ES, UK.
[b] EaStCHEM School of Chemistry, North Haugh, University of St Andrews, St Andrews KY16 9ST, UK.
[c] Department of Chemistry, University of Bath, Claverton Down, BA2 7AY Bath, UK.
[d] Dynamic Reaction Monitoring Facility, University of Bath, Claverton Down, BA2 7AY Bath, UK.
[e] Department of Chemistry, University of Victoria, 3800 Finnerty Rd, Victoria, BC, Canada.
[f] School of Chemistry, University of Edinburgh, Edinburgh, Scotland EH9 3FJ, UK.

**Contents**


1 Experimental ..... 4
1.1 General Procedures ..... 4
1.2 Synthesis of Complexes ..... 5
1.2.1 [Ir($^t$Bu-POCOP)$H_2$($NH_2$Me)] – Complex 4 ..... 5
1.2.2 [Ir($^t$Bu-POCOP)$H_3$][Na-18-crown-6($THF)_2$] – Complex [6][(Na(18-crown-6)($THF_2$] ..... 7
1.3 Catalytic Dehydropolymerisation of $H_3B{\cdot}NH_2Me$ ..... 9
1.3.1 Poisoning Experiments ..... 11
1.4 Kinetic Analysis for the dehydropolymerisation of $H_3B{\cdot}NH_2Me$ with Ir($^t$Bu-POCOP)$H_2$ (1) ..... 13
1.4.1 Effect of [$H_3B{\cdot}NH_2Me$] ..... 13
1.4.2 Recharge experiments ..... 14
1.4.3 Effect of [$H_3B{\cdot}NH_2Me$] with $NH_2Me$ ..... 15
1.4.4 Effect of catalyst loading ..... 16
1.4.5 Eyring Analysis ..... 18
1.4.6 Effect of $NH_2Me$ addition ..... 20
1.4.7 Effect of $[NH_3Me]^+$ and $Cl^-$ ..... 21
1.4.8 Effect of water addition ..... 23
1.4.9 Effect of isotopic substitution of $H_3B{\cdot}NH_2Me$ on dehydropolymerisation of $H_3B{\cdot}NH_2Me$ with Ir($^t$Bu-POCOP)$H_2$ ..... 24
1.5 Kinetic Analysis for the dehydrocoupling reaction of $H_3B{\cdot}NH_2Me$ with Ir($^t$Bu-POCOP)$H_2$($BH_3$) (3) ..... 25
1.6 Kinetic analysis for the dehydropolymerisation of $H_3B{\cdot}NH_2Me$ with Ir($^t$Bu-POCOP)$H_4$ (2) ..... 27
1.7 Kinetic analysis for the dehydropolymerisation of $H_3B{\cdot}NH_2Me$ with [Ir($^t$Bu-POCOP)$H_3$][Na(18-crown-6)($THF)_2$] (6) ..... 28
1.8 Catalyst speciation during the dehydropolymerisation of $H_3B{\cdot}NH_2Me$ ..... 29
2 NMR Spectra ..... 34
2.1 Ir($^t$Bu-POCOP)$H_2$($NH_2$Me) ..... 34
2.2 [Ir($^t$Bu-POCOP)$H_3$][Na(18-crown-6)($THF_2$)] ..... 36
2.3 *In situ* [Ir($^t$Bu-POCOP)$H_3$]Na ..... 39
2.4 [$NH_3$Me][$BAr^F{}_4$] ..... 40
3 NMR experiments ..... 43
3.1 Effect of $[Na(18\text{-crown-}6)]^+$ on $[Ir(^tBu\text{-POCOP})H_3]^-$ hydride region ..... 43
3.2 [Ir($^t$Bu-POCOP)H3][Na(18-crown-6)($THF)_2$] with [$NH_3$Me][$BAr^F{}_4$] (1 equiv.) ..... 45
3.3 Ir($^t$Bu-POCOP)(H)($BH_4$) with 20 equiv. $H_2O$ in THF ..... 46

3.4 Addition of $[NH_3Me]Cl$ to Ir($^t$Bu-POCOP)(H)($BH_4$) (3) ........ 49
4 Flow NMR ........ 50
4.1 Speciation data ........ 51
4.2 Reaction progress data ........ 54
5 Polymer Analysis ........ 56
5.1 Scale up procedure ........ 56
5.2 Molecular weight vs. conversion ........ 60
5.3 NMR spectra ........ 61
5.4 GPC ........ 62
5.5 TGA and DSC ........ 63
5.6 ICP ........ 67
5.7 Mass Spectrometry ........ 68
6 COPASI ........ 69
7 Cost Analysis of Ir($^t$Bu-POCOP)$H_2$ ........ 71
8 Computational Studies ........ 73
8.1 Computational details. ........ 73
8.2 Computed Dehydrogenation Mechanisms ........ 74
9 Cartesian coordinates (Å) and energies (a.u.) for the computed structures. ........ 86
10 Single Crystal X-Ray Diffraction Data ........ 131
References ........ 134

# 1 Experimental

## 1.1 General Procedures

Unless otherwise stated all experiments were carried out under argon atmosphere using standard glovebox and Schlenk techniques. Glassware and cannulas were dried overnight at 140 °C. SPS THF, pentane and toluene were dried using a Grubbs' solvent purification system[1] and degassed by 3 freeze-pump-thaw cycles. THF (pretreated over $AlO_3$ then 3 Å molecular sieves) was distilled over sodium/benzophenone and degassed by 3 freeze-pump-thaw cycles. All solvents were stored over 3 Å molecular sieves. $[Ir(^tBu\text{-}POCOP)H_2]$ (**1**) ($^t$Bu-POCOP = k$^3$-2,6-($^tBu_2PO)_2C_6H_3$),[2, 3] $[Ir(^tBu\text{-}POCOP)H_4]$ (**2**),[3] $[Ir(^tBu\text{-}POCOP)(H)(BH_4)]$ (**3**)[4] and $[Ir(^tBu\text{-}POCOP)H_3]Na$[3] were prepared by literature methods. Commercially sourced $H_3B{\cdot}NH_2Me$ was recrystallised from minimum amount of $OEt_2$ at −20 °C and filtered prior to use and stored at −40 °C. All other reagents were obtained from commercial sources and used without further purification unless stated.

NMR spectra were collected using a Bruker Advance III 600 MHz spectrometer or Bruker Avance III 500 MHz spectrometer at 298 K unless otherwise stated. Chemical shifts are expressed in ppm, with coupling expressed in Hz. $^1$H NMR spectra were referenced to residual protio solvent peaks. $^{31}$P {$^1$H} NMR spectra were externally referenced to 85% $H_3PO_4$ in $H_2O$ and $^{11}$B NMR spectra were referenced to 15% $BF_3OEt_2$ in $CDCl_3$.

All polymeric materials were analysed by GPC (Gel Permeation Chromatography) measured on a Malvern Viskotec $GPC_{max}$ together with a Viskotec TDA 305 RI detector. Polymer $M_n$ is referenced to polystyrene standards between $M_n$ 474-476,500 g mol$^{-1}$. All samples were passed through 3 columns consisting of a porous styrene divinylbenzene copolymer (column set: 2 × T5000 and 1 × T4000 Malvern columns). The eluent used was GPC grade THF containing $[NBu_4]Br$ (2.7 mmol dm$^{-3}$) and the flow rate was 1 cm$^3$ min$^{-1}$. All polymer samples were dissolved in GPC grade THF $[NBu_4]Br$ (2 g cm$^{-3}$) and filtered through a PTFE filter (pore size: 45 μm) prior to analysis.

## 1.2 Synthesis of Complexes

### 1.2.1 [Ir($^t$Bu-POCOP)$H_2$($NH_2Me$)] – Complex 4

$$[NH_3Me]Cl + NaH \xrightarrow[\text{12 hours}]{d^8\text{-THF}} NH_2Me_{(dissolved)} + NaCl + H_2$$

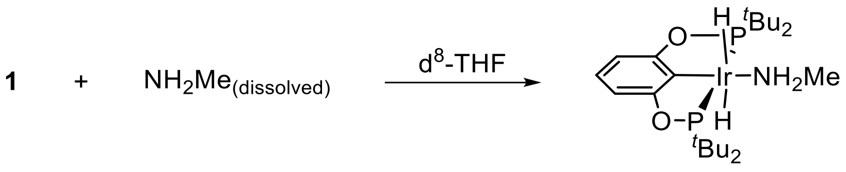

**4**

**4** can be formed *in-situ* by two methods. The addition of $NH_2Me$ (2 M in THF, 5 equiv.) to **1** (Ir($^t$Bu-POCOP)$H_2$) to form a yellow solution or by synthesis of $NH_2Me_{(g)}$ in $d^8$-THF then vacuum transferring this mixture onto **1**. To form **4** cleanly excess $NH_2Me$ is required as **4** is unstable and is in equilibrium with **1** and 'free' $NH_2Me$.

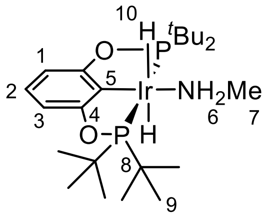

**4**

A mixture of [$NH_3Me$]Cl (5.7 mg, 84.5 μmol) and NaH (4 mg, 169 μmol) we dissolved in THF-$d^8$ in a J-Youngs NMR tube, sonicated for 20 minutes and then inverted for 12 hours with intermittent mixing (until bubbling ceased). The NMR tube was cooled to 77 K and the hydrogen gas removed under vacuum. The THF-$d^8$ and $NH_2Me_{(g)}$ were vacuum transferred onto solid Ir($^t$Bu-POCOP)$H_2$ **1** (5 mg, 8.4 μmol) and the solution thawed and agitated. The colour of the solution was pale yellow and formed **4** cleanly as measured by NMR spectroscopy (Figure S31-S34).

$^1$H NMR (600 MHz; THF-$d^8$) $\delta_H$: 6.31 (1H, vt, *J* = 7.7 Hz, Ar-<u>H</u>2), 6.07 (2H, d, *J* = 7.7 Hz, Ar-<u>H</u>1), 3.23 (2H, s (br), N-<u>H</u>$_2$(6)), 2.88 (3H, t, *J* = 6.6 Hz, N-C<u>H</u>$_3$(7)), 1.42 (36 H, t, *J* = 6.6 Hz, $^t$Bu-<u>H</u>9), −9.19 (2H, t, *J* = 16.1 Hz, Ir-<u>H</u>10).

$^{31}$P{$^1$H} NMR (243 MHz; THF-$d^8$) $\delta_P$: 170.13.

$^{13}$C{$^1$H} NMR (151 MHz; THF-$d^8$) $\delta_C$: 163.55 (t, *J* = 6.6 Hz, <u>C</u>5), 129.20 (s, <u>C</u>4), 120.55 (s, <u>C</u>2), 103.39 (t, *J* = 6.5 Hz, <u>C</u>1), 44.38 (s, <u>C</u>7), 40.44 (t, *J* = 12.5 Hz, <u>C</u>8), 29.05 (t, *J* = 3.44 Hz, <u>C</u>9).

To study the complex using EXSY $^1$H NMR spectroscopy a solution of [Ir($^t$Bu-POCOP)$H_2$] **1** (10 mg, 16.9 μmol) and $NH_2Me$ 2 M in THF (42.2 μL, 84.4 μmol) in THF (0.5 mL) was stirred for 12 hours. Analysis by $^1$H and $^{31}$P{$^1$H} NMR spectroscopies showed complete conversion to **4**. The solvent was removed (flow of argon), and $d^8$-THF was vacuum transferred onto the yellow residue.

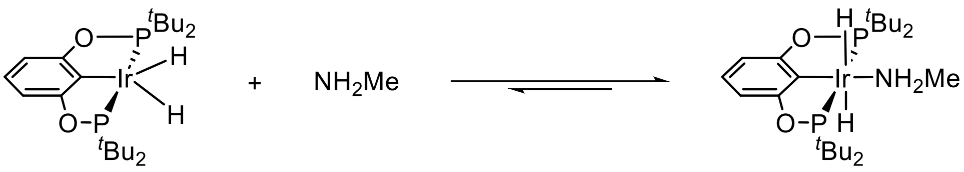

**1** **4**

The exchange constant for the forward reaction = 0.33 $M^{-1}$ $s^{-1}$

The exchange constant for the backward reaction = 0.0666 $s^{-1}$

A small number of crystals were obtained by removal of THF (flow of argon at RT), then recrystallisation from pentane at −80 °C.

Compositional purity was determined to the detection limit of $^{1}H$ NMR spectroscopy. Elemental analysis was not collected for this complex as it is only stable in the presence of excess $NH_2Me$.

### 1.2.2 [Ir($^t$Bu-POCOP)$H_3$][Na-18-crown-6(THF)$_2$] – Complex [6][(Na(18-crown-6)(THF$_2$]

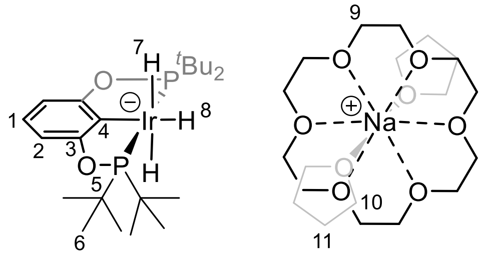

A solution of Ir($^t$Bu-POCOP)$H_2$ (50 mg, 84.5 μmol), NaH (4 mg, 177 μmol) and 18-crown-6 (46.8 mg, 177 μmol) in THF (3 mL) were stirred at 298 K for 24 hours. The reaction was filtered by cannula filtration and layered with hexane to afford colourless crystals (46 mg, 52.3 mmol, 62 % yield). For NMR spectroscopy data see Figure S35-S38.

$^1$H NMR (600 MHz; THF-d$^8$) $\delta_H$: 6.16 (1H, t, $J$ = 7.5 Hz, Ar-H1), 6.03 (2H, d, $J$ = 7.5 Hz, Ar-H2), 3.61 (5H, m, crown-bound THF (10)), 3.56 (24H, s, C-$H_2$(9)), 1.77 (5H, m, crown-bound THF (11)), 1.33 (36H, t, $J$ = 6.3 Hz, $^t$Bu-H6), −11.58 (1H, septet app, $J$ = 5.5 Hz, Ir-H(8)), −13.22 (2H, td, $J$ = 16.7, 5.5 Hz, Ir-H7,).

$^{31}$P{$^1$H} NMR (243 MHz; THF-d$^8$) $\delta_P$: 192.31.

$^{13}$C{$^1$H} NMR (151 MHz; THF-d$^8$) $\delta_C$: 165.0 (t, $J$ = 7.5 Hz, C3), 140.0 (s, C4), 117.6 (s, C1), 101.3 (t, $J$ = 5.5 Hz, C2), 70.9 (s, C9), 68.4 (s, C10), 39.6 (t, $J$ = 11.5 Hz, C5), 30.3 (t, $J$ = 3.5 Hz, C6), 26.6 (s, C11).

Compositional purity was determined to the detection limit of $^1$H NMR spectroscopy. Elemental analysis was attempted on this complex but due to the air sensitive nature the results were inconclusive.

Crystals suitable for single crystal X-ray diffraction were obtained by recrystallisation from THF/hexane.

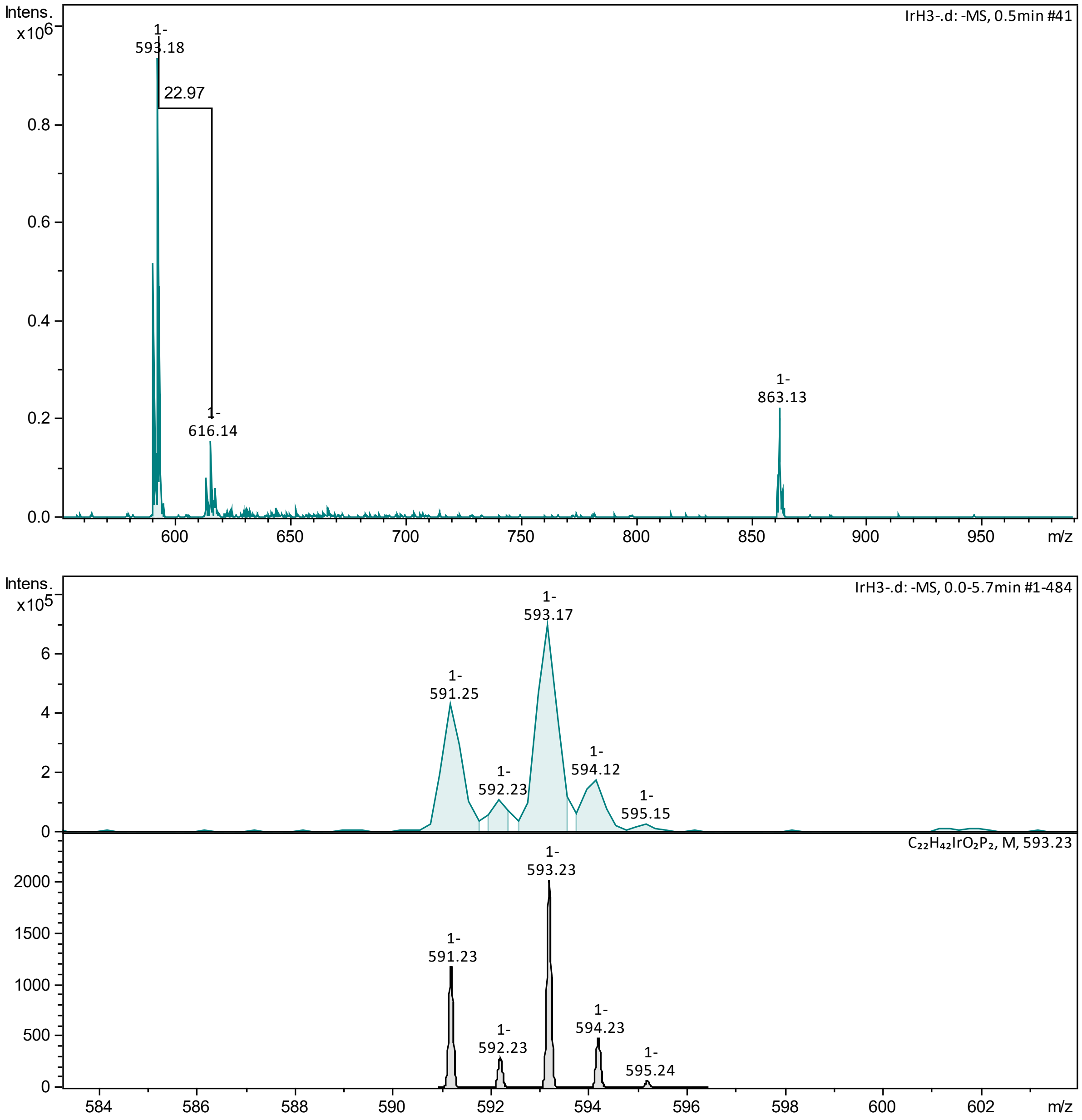


**Figure S1.** TOP: ESI negative mode mass spectrum of [Ir($^t$Bu-POCOP)H$_3$][Na-18-crown-6] (**6**) recorded in THF, [M]$^-$ 593.2 m/z [M+Na]$^-$ 616.1 m/z. BOTTOM: shows an expanded view of the observed molecular ion and the simulated isotope pattern showing a good fit. The peak at m/z 863 is a persistent contaminate in the mass spectrometer which we attribute to [BAr$^F_4$]$^-$ (tetrakis[3,5-bis(trifluoromethyl)phenyl]borate).

## 1.3 Catalytic Dehydropolymerisation of $H_3B{\cdot}NH_2Me$

### Typical eudiometric dehydropolymerisation procedure

Eudiometric measurements of hydrogen gas production were performed using an upturned burette filled with water that was displaced as hydrogen gas was produced.

Under standard conditions: Mono-methyl amine-borane, $H_3B{\cdot}NH_2Me$ (MMAB) (112mg, 2.5 mmol) was placed in a two-neck jacketed Schlenk flask with temperature maintained by a circulating cooler (20 °C). The catalyst (1.5 mg, 0.1 mol%) was dissolved in a known volume of THF (1.25 mL) separately. The flask was connected to the water filled upturned burette via PTFE tubing. The catalyst solution was added to the jacketed flask and the solution stirred at 400 rpm. The volume and time were recorded at 1 $cm^3$ increments of gas collected, aided by video recording when required. After completion of gas evolution, an in-situ NMR sample (0.5 mL) was taken and analysed. Pentane (50 mL) was added, and the solution stirred rapidly to induce polymer precipitation. The off-white solid was isolated by filtration and remaining volatiles were removed in vacuo. The polymer was analysed by $^{1}H$, $^{31}P\{^{1}H\}$c and $^{11}B$ NMR spectroscopies and gel permeation chromatography (GPC).

Kinetic profiles are expressed in equivalents of $H_2$ plotted against time. The equivalents of $H_2$ are proportional to [$H_2B{=}NHMe$] as the catalysis is highly selective for polymer formation (<1% other BN dehydrocoupling products) and $H_2$ is the major by-product. The equivalents of gas released exceed 1 in most cases. We attribute this to THF evaporation which has been measured independently (Figure S4). This effect is most obvious during long induction periods and when turnover is fast (bubbling). Rates are measured from plots of [$H_2B{=}NHMe$] against time from the pseudo-order regime, with errors from regression analysis.

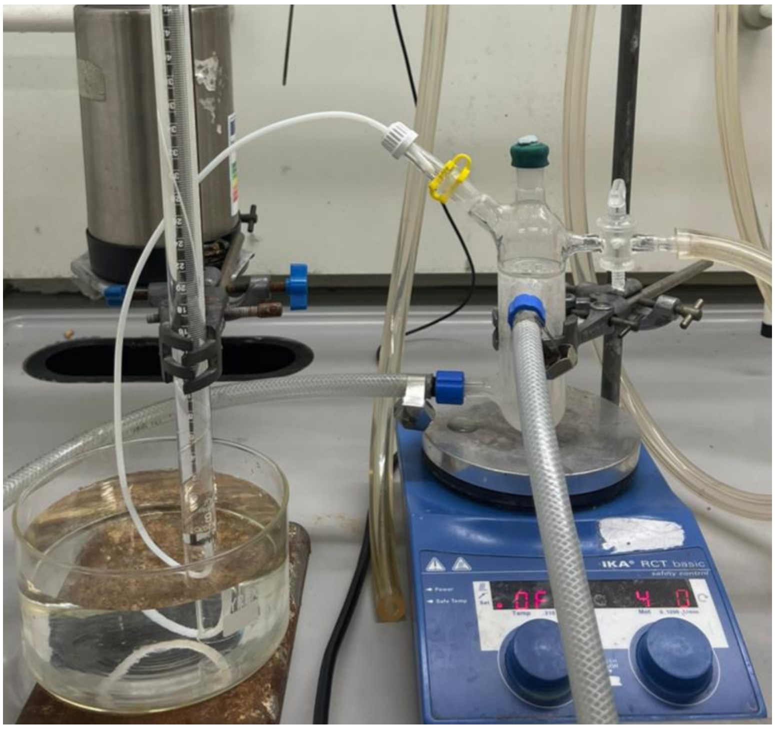

**Figure S2.** Eudiometric setup consisting of a jacketed Schlenk flask, upturned water-filled burette and magnetic stirring plate set at 400 rpm. A recirculating cooler controls the temperature with a 1:1 water:polyethylene glycol mixture.

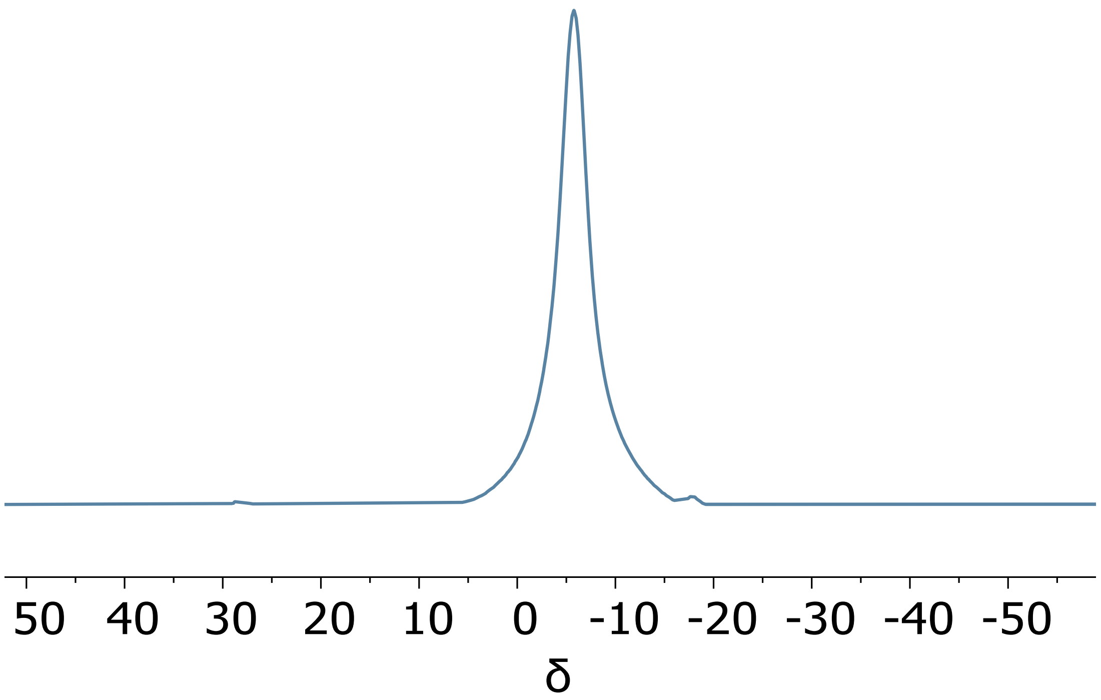


**Figure S3.** Representative $^{11}B$ NMR spectrum taken on catalysis completion showing >99% conversion and >99% selectivity for formation of polyaminoborane under standard conditions, H$^{8}$-THF, 193 MHz, 298 K.

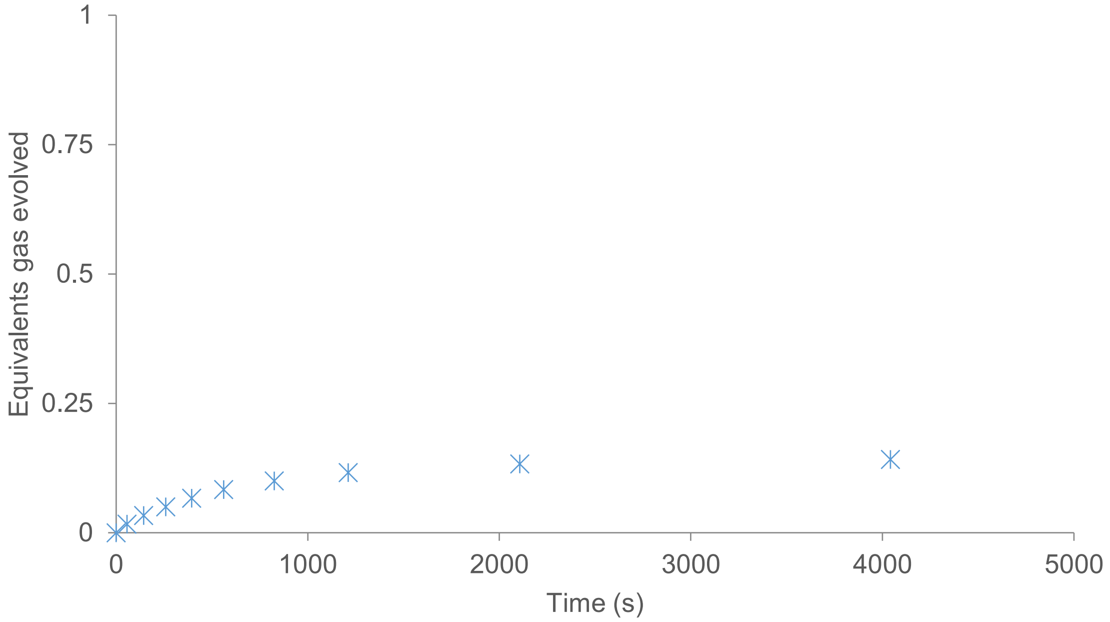


**Figure S4.** Plot showing the 'equivalents' of gas evolved when THF is stirred over time under eudiometric conditions, showing the effect THF evaporation can have on the apparent equivalents of $H_2$ produced over the course of a reaction.

### 1.3.1 Poisoning Experiments

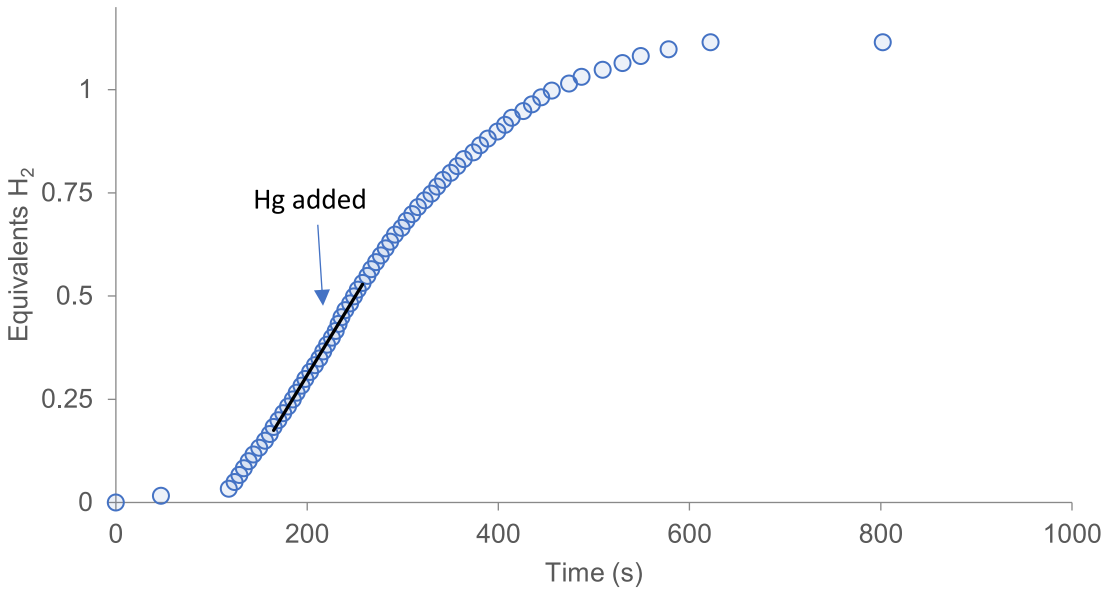


**Figure S5.** Poisoning test for kinetics of hydrogen release from $H_3B{\cdot}NH_2Me$ with Ir($^t$Bu-POCOP)$H_2$ (**1**). Conditions: 0.1 mol% [Ir], 2 M $H_3B{\cdot}NH_2Me$, THF, 293 K, 0.1 g Hg.

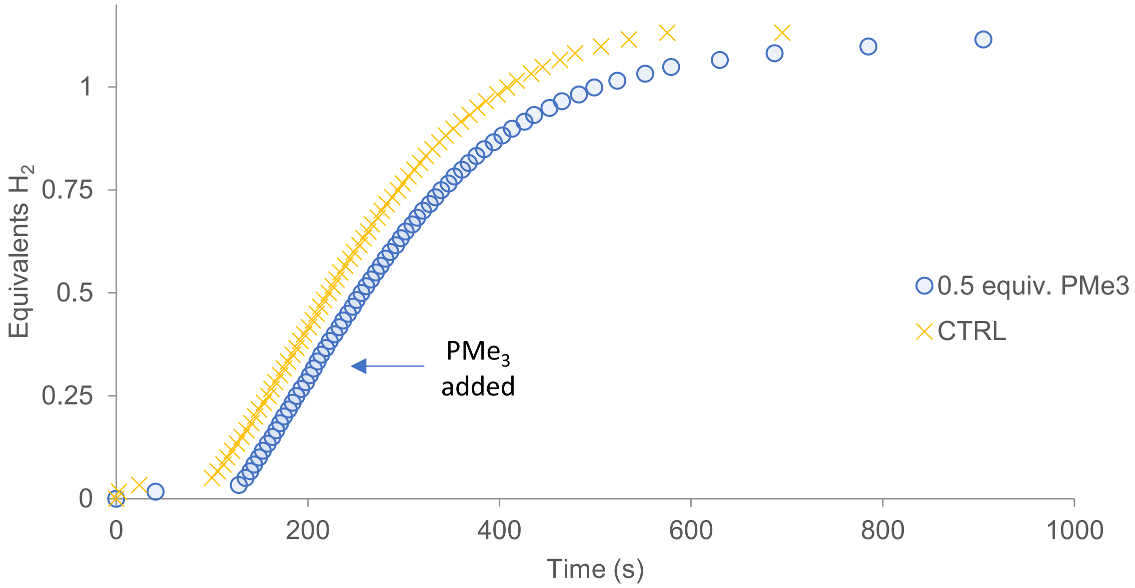


**Figure S6.** Poisoning test for kinetics of hydrogen release from $H_3B{\cdot}NH_2Me$ with Ir($^t$Bu-POCOP)$H_2$ (**1**). Conditions: 0.1 mol% [Ir], 2 M $H_3B{\cdot}NH_2Me$, THF, 293 K, 0.5 equiv. $PMe_3$.

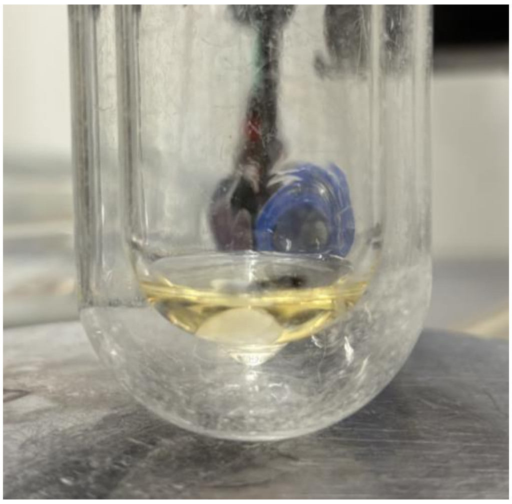

**Figure S7.** Image of reaction solution at the end of catalysis, showing a clear, light-coloured solution.

### 1.4 Kinetic Analysis for the dehydropolymerisation of $H_3B·NH_2Me$ with Ir(tBu-POCOP)$H_2$ (1)

#### 1.4.1 Effect of [$H_3B·NH_2Me$]

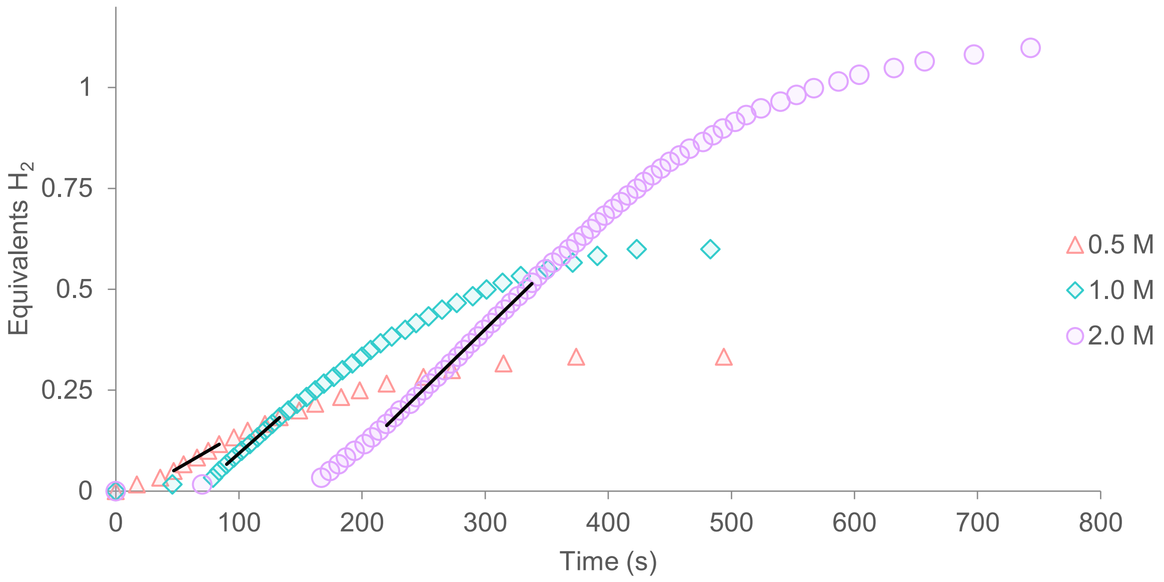


**Figure S8.** Kinetics of hydrogen release from $H_3B·NH_2Me$ using Ir(tBu-POCOP)$H_2$ (**1**) at different [$H_3B·NH_2Me$]. Conditions: 2 mM Ir(tBu-POCOP)$H_2$, THF, 293 K.

**Table S1.** $H_3B·NH_2Me$ dehydropolymerisation using Ir(tBu-POCOP)$H_2$ (**1**) at different [$H_3B·NH_2Me$]. [a]Determined by the pseudo zero order region of the profile [b]Measured by $^{11}B$ NMR spectroscopy [c]Relative to polystyrene standards.

| Entry | [$H_3B·NH_2Me$] (M) | Rate[a] (mM $s^{-1}$) | Induction period (s) | Conv.[b] (%) | Selectivity[b] (%) | $M_n$[c] (g $mol^{-1}$) | Đ[c] | Yield (mg (%)) |
|---|---|---|---|---|---|---|---|---|
| 1 | 0.5 | 3.53(10) | 17 | >99 | >99 | 96,000 | 1.4 | 14 (52) |
| 2 | 1.0 | 5.38(5) | 79 | >99 | >99 | 113,700 | 1.5 | 30 (60) |
| 3 | 2.0 | 5.95(3) | 167 | >99 | >99 | 103,100 | 1.3 | 66 (62) |

### 1.4.2 Recharge experiments

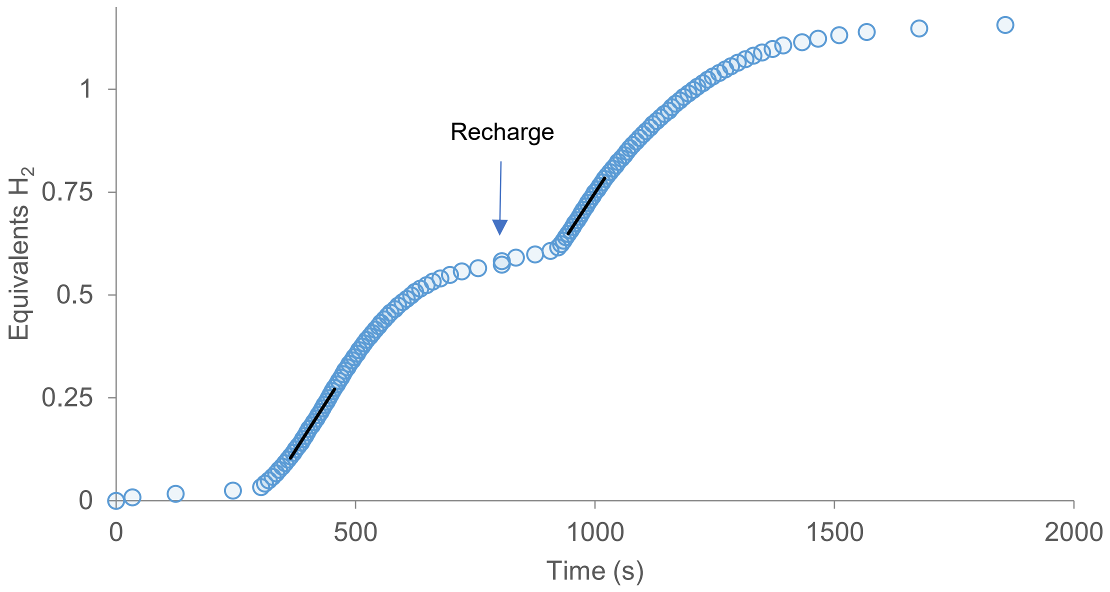


**Figure S9.** Eudiometric measurement of $H_2$ release using pre-catalyst Ir($^t$Bu-POCOP)$H_2$ (**1**) with recharge, indicated by the arrow. Conditions: 2M $H_3B{\cdot}NH_2Me$, 0.1 mol% catalyst loading, temperature 20 °C, without amine**.**

**Table S2.** Dehydropolymerisation of $H_3B{\cdot}NH_2Me$ with Ir($^t$Bu-POCOP)$H_2$ (**1**) (Recharge). Conditions: Initially 2M $H_3B{\cdot}NH_2Me$, 0.1 mol% catalyst loading, temperature 20 °C, without amine. $^a$Determined by the pseudo zero order region of the profile $^b$Measured by $^{11}$B NMR spectroscopy $^c$Relative to polystyrene standards.

| Recharge | Induction period (s) | Rate$^a$ (mM s$^{-1}$) | Conv.$^b$ (%) | Selectivity$^b$ (%) | $M_n$$^c$ (g mol$^{-1}$) | Đ |
|---|---|---|---|---|---|---|
| N/A | 303 | 6.30(5) | | | - | - |
| Recharge | 118 | 6.14(5) | >99 | >99 | 128,300 | 1.6 |

### 1.4.3 Effect of [$H_3B{\cdot}NH_2Me$] with $NH_2Me$

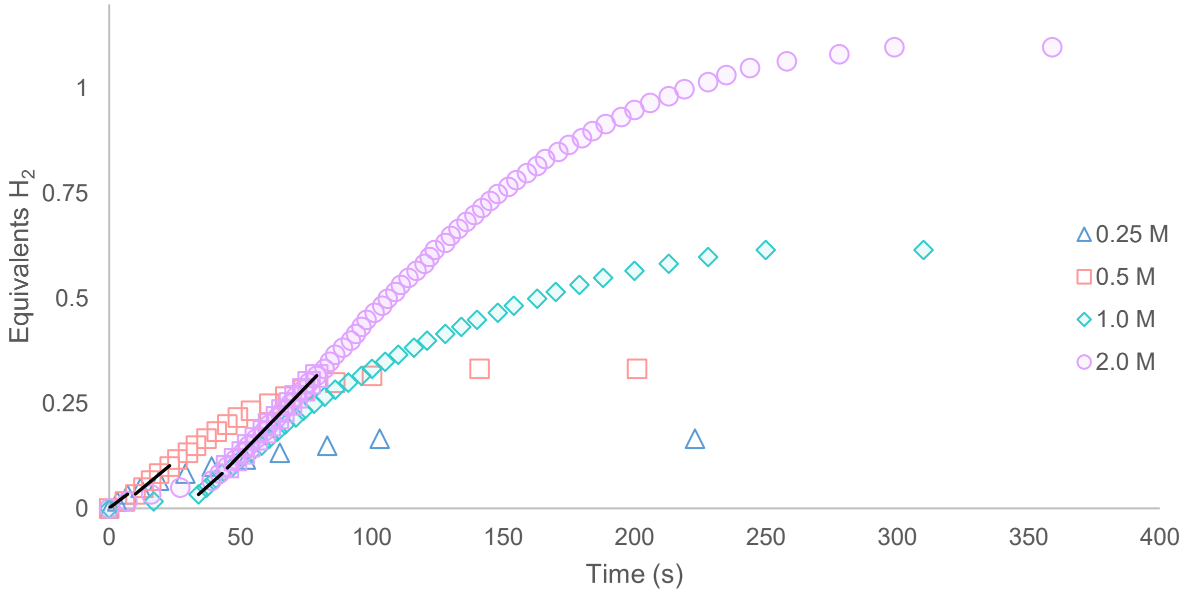


**Figure S10.** Kinetics data for the dehydropolymerisation of $H_3B{\cdot}NH_2Me$ with Ir($^t$Bu-POCOP)$H_2$ (**1**) at varied [$H_3B{\cdot}NH_2Me$] with added $NH_2Me$ (5 equiv. with regard to [cat.]). Conditions: 2 mM Ir($^t$Bu-POCOP)$H_2$, THF, 293 K and 5 equiv. $NH_2Me$ added with regard to catalyst.

**Table S3.** $H_3B{\cdot}NH_2Me$ dehydropolymerisation using Ir($^t$Bu-POCOP)$H_2$ (**1**) at different [$H_3B{\cdot}NH_2Me$] with added $NH_2Me$. [a]Determined by the pseudo zero order region of the profile [b]Measured by $^{11}$B NMR spectroscopy [c]Relative to polystyrene standards.

| Entry | [$H_3B{\cdot}NH_2Me$] (M) | [$NH_2Me$] (mM) | Rate[a] (mM $s^{-1}$) | Induction period (s) | Conv.[b] (%) | Selectivity[b] (%) | $M_n$[c] (g $mol^{-1}$) | Đ[c] | Yield (mg (%)) |
|---|---|---|---|---|---|---|---|---|---|
| 1 | 0.25 | 10 | 9.4(8) | 0 | >99 | >99 | 152,400 | 1.5 | 4 (30) |
| 2 | 0.5 | 10 | 9.5(3) | 6 | >99 | >99 | 178,800 | 1.5 | 15 (56) |
| 3 | 1.0 | 10 | 11.1(2) | 37 | >99 | >99 | 159,800 | 1.5 | 34 (63) |
| 4 | 2.0 | 10 | 12.9(1) | 53 | >99 | >99 | 176,300 | 1.5 | 72 (67) |

### 1.4.4 Effect of catalyst loading

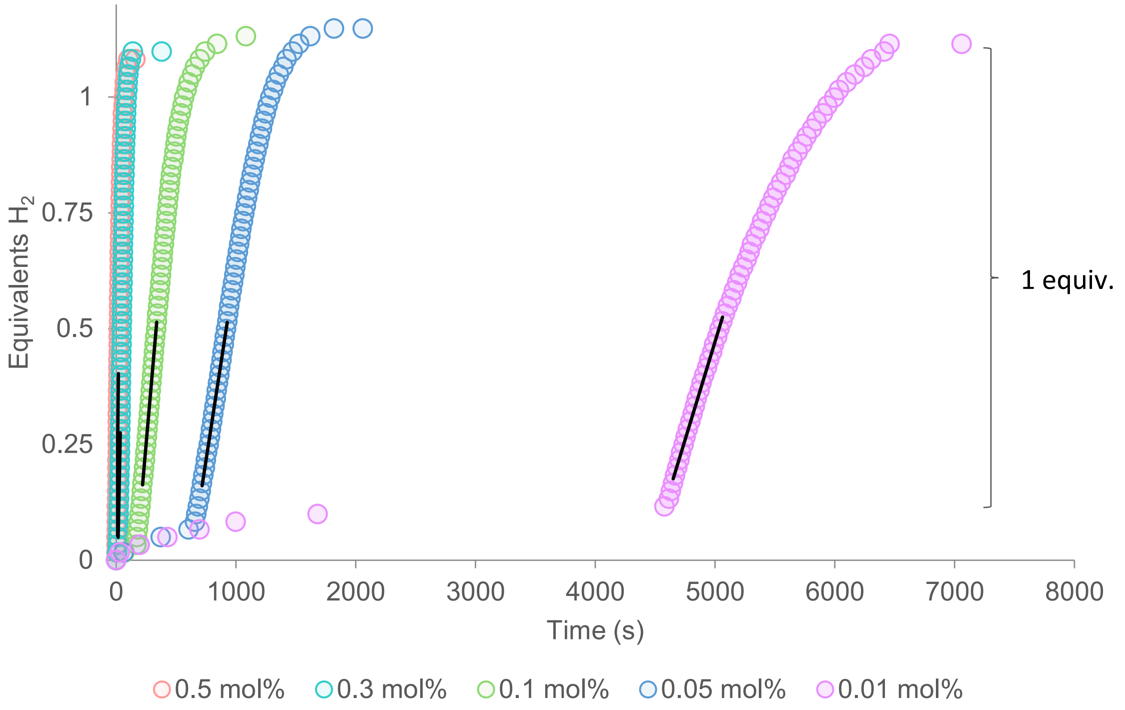


**Figure S11.** Dehydropolymerisation of $H_3B{\cdot}NH_2Me$ with Ir($^t$Bu-POCOP)$H_2$ (**1**) at different catalyst concentrations, (0.2 mM – 10 mM, 0.01 mol%-0.5 mol%). Conditions: 2M $H_3B{\cdot}NH_2Me$, THF, 293 K.

**Table S4.** Dehydropolymerisations of $H_3B{\cdot}NH_2Me$ with Ir($^t$Bu-POCOP)$H_2$ (**1**, [Ir]) at different catalyst concentrations. [a]Determined by the pseudo zero order region of the profile [b]Measured by $^{11}$B NMR spectroscopy [c]Relative to polystyrene standards.

| Entry | [Ir] (mM) | Cat. loading (mol %) | Rate[a] (mM $s^{-1}$) | Induction period (s) | Conv.[b] (%) | Selectivity[b] (%) | $M_n$[c] (g $mol^{-1}$) | Đ[c] | Yield (mg (%)) |
|---|---|---|---|---|---|---|---|---|---|
| 1 | 10 | 0.5 | 49.8(9) | 0 | >99 | >99 | 125,700 | 1.4 | 70 (65) |
| 2 | 6 | 0.3 | 25.1(5) | 36 | >99 | >99 | 131,600 | 1.4 | 60 (56) |
| 3 | 2.0 | 0.1 | 5.95(3) | 167 | >99 | >99 | 103,100 | 1.3 | 66 (62) |
| 4 | 1 | 0.05 | 3.36(2) | 658 | >99 | >99 | 91,700 | 1.3 | 50 (47) |
| 5 | 0.2 | 0.01 | 1.69(2) | 4577 | >99 | >99 | 57,300 | 1.5 | 66 (62) |

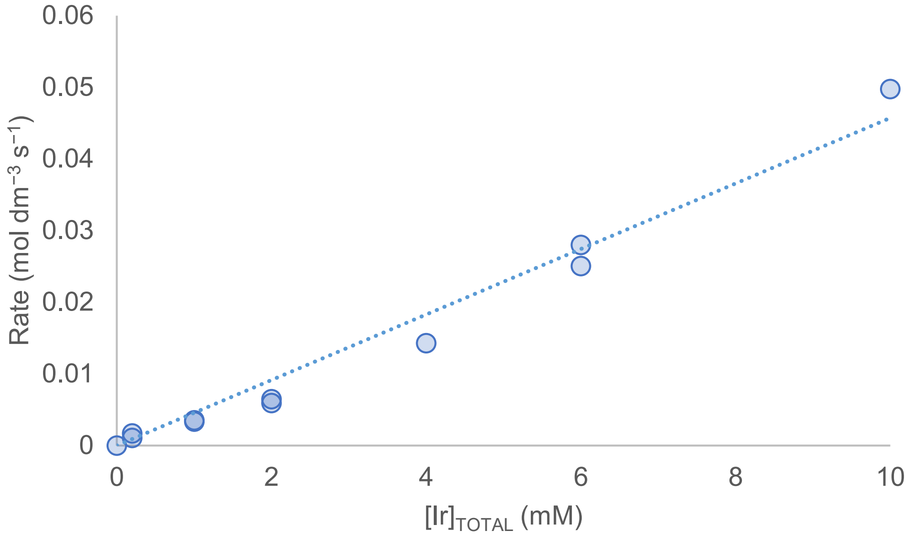


**Figure S12.** Plot of rate against concentration of Ir.

### 1.4.5 Eyring Analysis

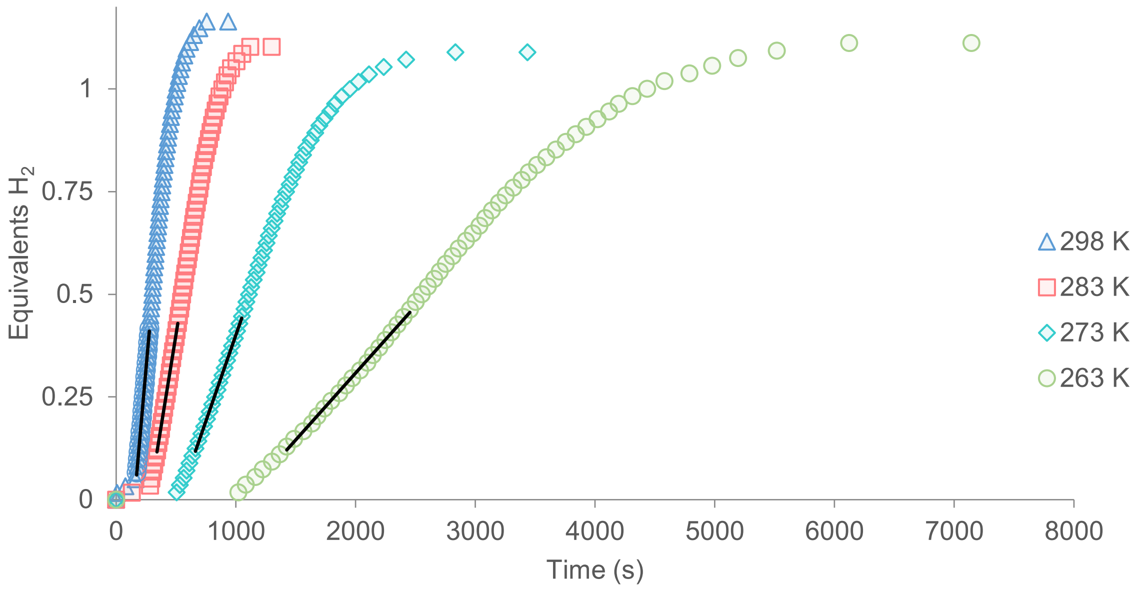


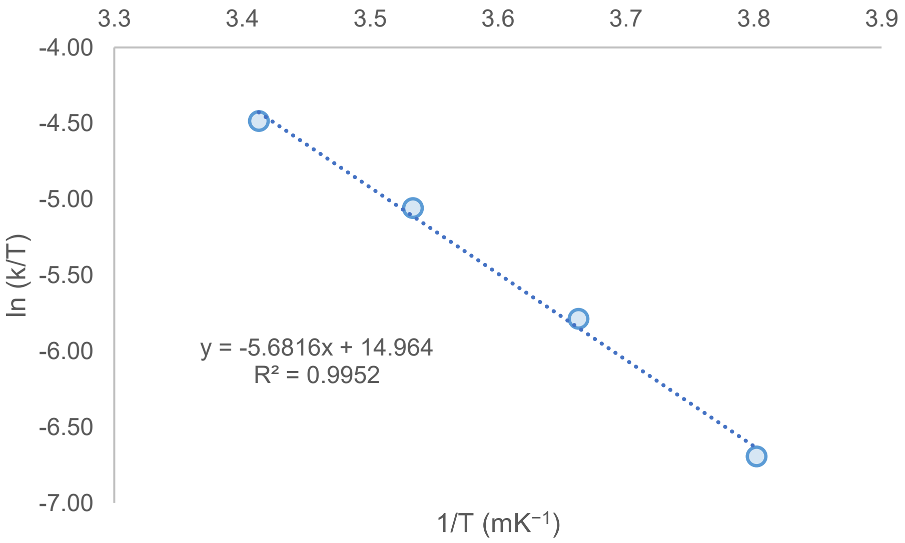


**Figure S13.** Dehydropolymerisation of $H_3B{\cdot}NH_2Me$ at various temperatures (top) and corresponding Eyring plot (bottom). Conditions: 2 M $H_3B{\cdot}NH_2Me$, 2 mM cat., THF.

| | |
|---|---|
| $\Delta H^{\ddagger}$ (kcal mol$^{-1}$) | 11 (1) |
| $\Delta S^{\ddagger}$ (cal K$^{-1}$ mol$^{-1}$) | −17 (2) |
| $\Delta G_{293}^{\ddagger}$ (kcal mol$^{-1}$) | 16 (1) |

**Table S5.** Dehydropolymerisations of $H_3B{\cdot}NH_2Me$ with Ir($^t$Bu-POCOP)$H_2$ (**1**) at different temperatures. [a]Determined by the pseudo zero order region of the profile [b]Measured by $^{11}$B NMR spectroscopy [c]Relative to polystyrene standards.

| Entry | Temp. (K) | Rate[a] (mM $s^{-1}$) | Induction period (s) | Conv.[b] (%) | Selectivity[b] (%) | $M_n$[c] (g $mol^{-1}$) | Đ[c] | Yield (mg (%)) |
|---|---|---|---|---|---|---|---|---|
| 1 | 293 | 6.61(6) | 163 | >99 | >99 | 95,200 | 1.4 | 63 (59) |
| 2 | 283 | 3.60(2) | 282 | >99 | >99 | 124,200 | 1.4 | 58 (54) |
| 3 | 273 | 1.68(1) | 506 | >99 | >99 | 166,100 | 1.5 | 59 (55) |
| 4 | 263 | 0.65(1) | 1020 | >99 | >99 | 191,200 | 1.5 | 53 (49) |

### 1.4.6 Effect of $NH_2Me$ addition

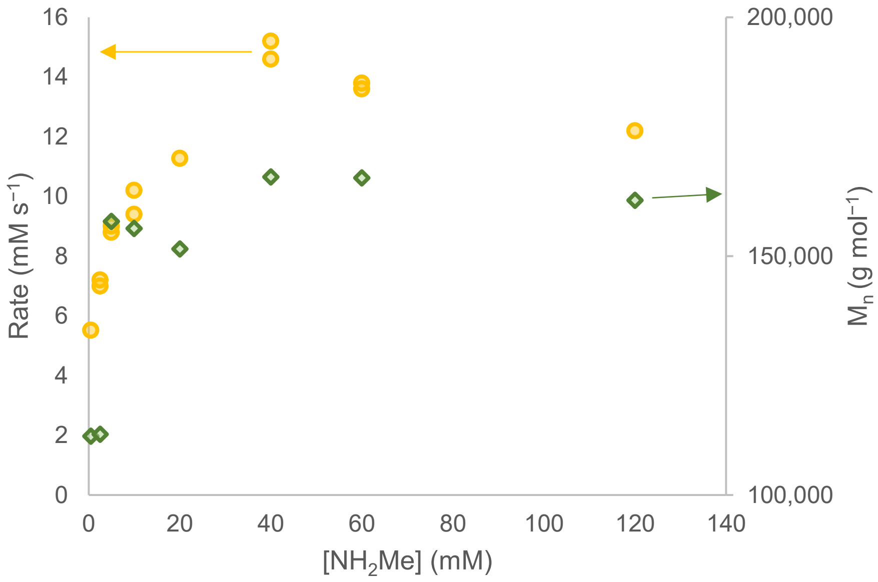


**Figure S14.** Plot of rate against concentration of added $NH_2Me$ to the dehydropolymerisation of $H_3B{\cdot}NH_2Me$ with Ir($^t$Bu-POCOP)$H_2$ (**1**). $NH_2Me$ 2 M in THF.

**Table S6.** Effect of $NH_2Me$ (2 M in THF) addition on the dehydropolymerisation of $H_3B{\cdot}NH_2Me$ with Ir($^t$Bu-POCOP)$H_2$ (**1**). Conditions: 2 M $H_3B{\cdot}NH_2Me$, 2 mM (0.1 mol%) Ir($^t$Bu-POCOP)$H_2$, 293 K, THF. [a]Determined by the pseudo zero order region of the profile [b]Measured by $^{11}$B NMR spectroscopy [c]Relative to polystyrene standards.

| Entry | [$NH_2Me$] (mM) | Rate[a] (mM $s^{-1}$) | Induction period (s) | Conv.[b] (%) | Selectivity[b] (%) | $M_n$[c] (g $mol^{-1}$) | Đ[c] | Yield (mg (%)) |
|---|---|---|---|---|---|---|---|---|
| 1 | 0.5 | 5.52(2) | 317 | >99 | >99 | 112,300 | 1.6 | 48 (45) |
| 2 | 2.5 | 7.20(5) | 298 | >99 | >99 | 112,700 | 1.6 | 54 (50) |
| 3 | 5 | 8.80(3) | 139 | >99 | >99 | 157,300 | 1.5 | 49 (46) |
| 4 | 10 | 10.2(1) | 100 | >99 | >99 | 155,800 | 1.5 | 79 (74) |
| 5 | 20 | 11.3(1) | 41 | >99 | >99 | 151,500 | 1.7 | 67 (63) |
| 6 | 40 | 14.6(1) | 20 | >99 | >99 | 166,600 | 1.6 | 64 (60) |
| 7 | 60 | 13.6(1) | 19 | >99 | 98 | 166,400 | 1.6 | 59 (55) |
| 8 | 120 | 12.2(1) | 7 | >99 | 96 | 161,700 | 1.6 | 56 (52) |

### 1.4.7 Effect of $[NH_3Me]^+$ and $Cl^-$

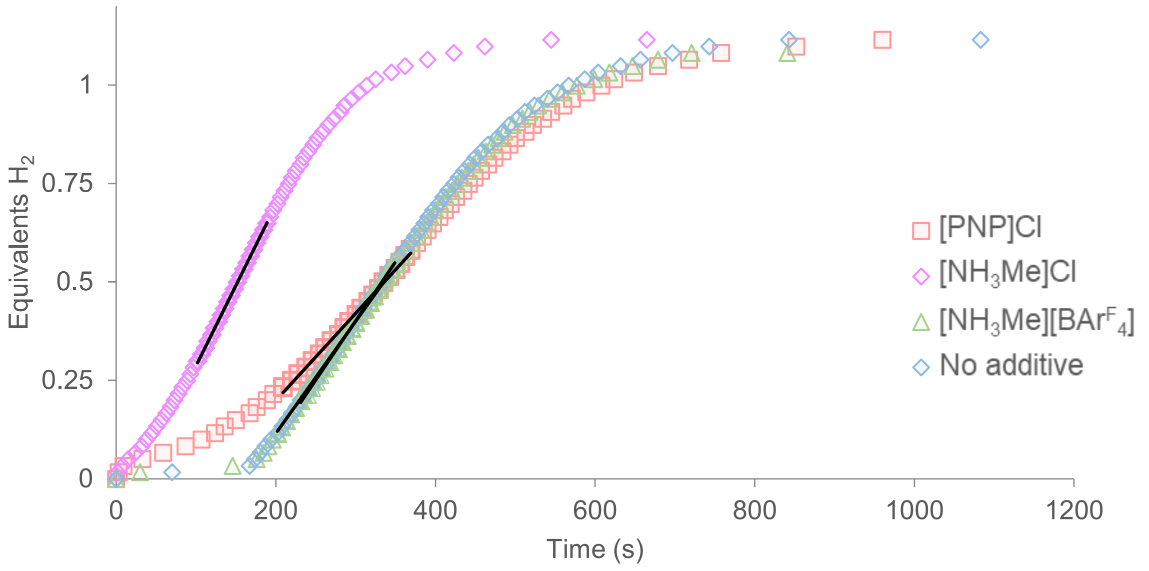


**Figure S15.** Dehydropolymerisation of $H_3B{\cdot}NH_2Me$ with Ir($^t$Bu-POCOP)$H_2$ (**1**), showing the effect of the addition of ammonium and chloride. Conditions: 2 M $H_3B{\cdot}NH_2Me$, 2 mM catalyst, THF, 298 K, 5 equivalents of additive.

**Table S7.** Effect of ammonium and chloride additives on dehydropolymerisation with Ir($^t$Bu-POCOP)$H_2$ (**1**). Conditions: 2 M $H_3B{\cdot}NH_2Me$, 2 mM (0.1 mol%) of 1, 293 K, THF. [a]Determined by the pseudo zero order region of the profile [b]Measured by $^{11}$B NMR spectroscopy [c]Relative to polystyrene standards. $[BAr^F_4]^-$ - tetrakis[3,5-bis(trifluoromethyl)phenyl]borate, [PNP]Cl - Bis(triphenylphosphine)iminium chloride. *The polymer and $[BAr^F_4]^-$ peaks could not be deconvoluted in the chromatogram and artificially leads to skewed distribution.[5]

| Entry | Additive | Rate[a] (mM s$^{-1}$) | Induction period (s) | Conv.[b] (%) | Selectivity[b] (%) | $M_n$[c] (g mol$^{-1}$) | Đ[c] | Yield (mg (%)) |
|---|---|---|---|---|---|---|---|---|
| 1 | None | 5.93(3) | 167 | >99 | >99 | 103,100 | 1.3 | 66 (62) |
| 2 | $[NH_3Me]Cl$ | 8.17(3) | 0 | >99 | >99 | 93,500 | 1.7 | 60 (56) |
| 3 | $[NH_3Me][BAr^F_4]$ | 5.73(2) | 179 | >99 | >99 | 57,700* | 1.4* | 77 (72) |
| 4 | [PNP]Cl | 4.63(4) | 139 | >99 | >99 | 92,800 | 1.5 | 71 (66) |

Stoichiometric experiments point to the role of $[NMeH_3]Cl$ in reducing the induction period for these very low catalyst loadings. Addition of five equivalents of sparingly soluble $[NMeH_3]Cl$ to **3** in THF results in the formation of the hydrido-chloride complex Ir($^t$Bu-POCOP)HCl,[6] **7** (~20%), alongside $H_3B{\cdot}NMeH_2$ [d($^{11}$B) –17.5, quartet] and unreacted **3** (~80%). We have recently shown that complex **7** is an active pre-catalyst for dehydropolymerisation, likely via the formation of **1** via base/promoted hydride transfer (Scheme S1).[7, 8] Thus, under the condition used, the rapid formation of the active catalyst occurs, that would be refreshed by $[NMeH_3]Cl/NMeH_2$. Eudiometric studies under standard conditions (Fig. S15) demonstrate the requirement for both $[NMeH_3]^+$ and $Cl^-$ in removing the induction period. Addition of $[NMeH_3][BAr^F_4]$ or [PPN]Cl resulted in significant induction periods, while addition of $[NMeH_3]Cl$ removed the induction period completely.

**Scheme S1.** Suggested activation of complex 3 under $[NMeH_3]Cl/NMeH_2$ conditions.

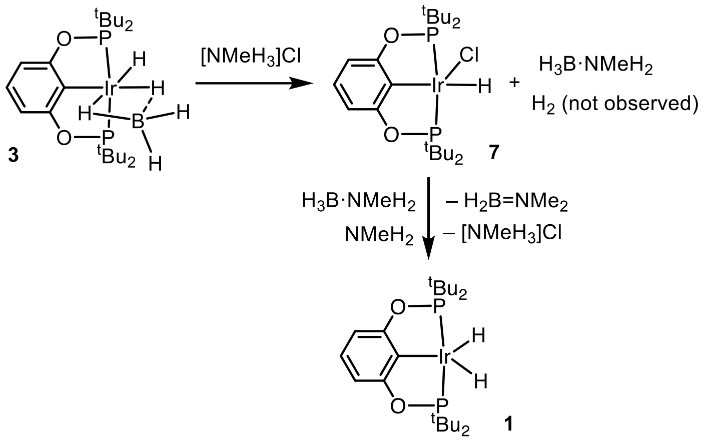

## 1.4.8 Effect of water addition

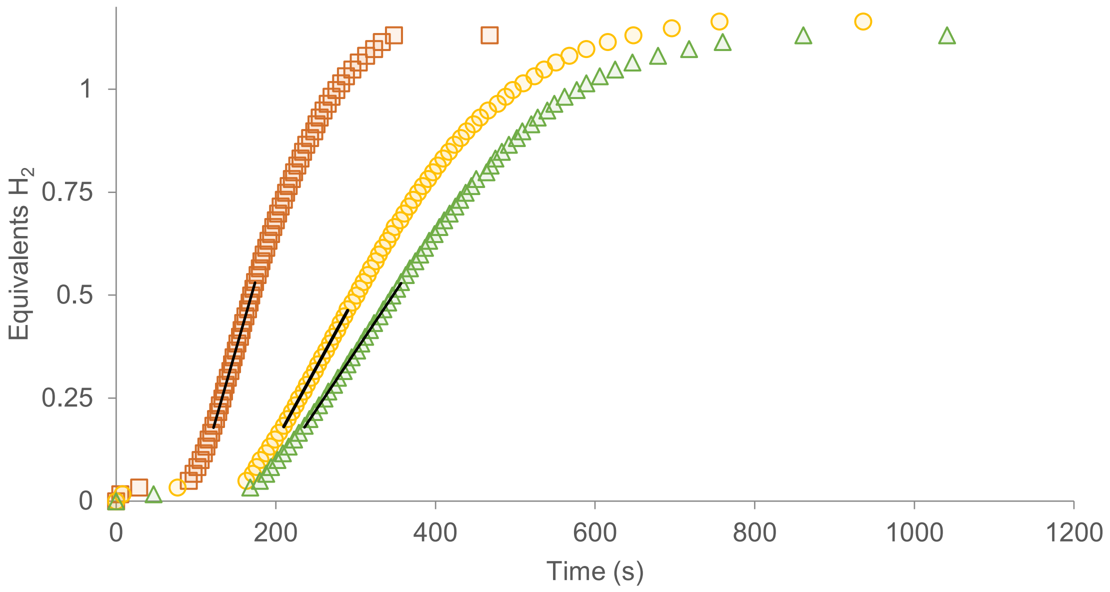


**Figure S16.** Dehydropolymerisation of $H_3B{\cdot}NH_2Me$ with Ir(tBu-POCOP)$H_2$ (**1**), showing the effect of $H_2O$ concentration in THF. Conditions: 2 M $H_3B{\cdot}NH_2Me$, 2 mM catalyst, THF, 293 K □ 1400 ppm $H_2O$ ○ 10 ppm $H_2O$ (stills) △ <10 ppm $H_2O$ (dried over NaH, stored over K and distilled prior to use).

**Table S8.** Effect of $H_2O$ on dehydropolymerisation with Ir(tBu-POCOP)$H_2$ (**1**). Conditions: 2 M $H_3B{\cdot}NH_2Me$, 2 mM (0.1 mol%) of 1, 293 K, THF. [a]Determined by the pseudo zero order region of the profile [b]Measured by $^{11}$B NMR spectroscopy [c]Relative to polystyrene standards.

| Entry | $H_2O$ (ppm) | Rate[a] (mM $s^{-1}$) | Induction period (s) | Conv.[b] (%) | Selectivity[b] (%) | $M_n$[c] (g $mol^{-1}$) | Đ[c] | Yield (mg (%)) |
|---|---|---|---|---|---|---|---|---|
| 1 | 1400 | 13.5(1) | 77 | >99 | >98 | 168,200 | 1.4 | 64 (60) |
| 2 | 10 | 6.61(3) | 163 | >99 | >99 | 95,200 | 1.4 | 63 (59) |
| 3 | <10 | 5.89(2) | 168 | >99 | >99 | 110,000 | 1.4 | 75 (70) |

### 1.4.9 Effect of isotopic substitution of $H_3B·NH_2Me$ on dehydropolymerisation of $H_3B·NH_2Me$ with Ir($^t$Bu-POCOP)$H_2$

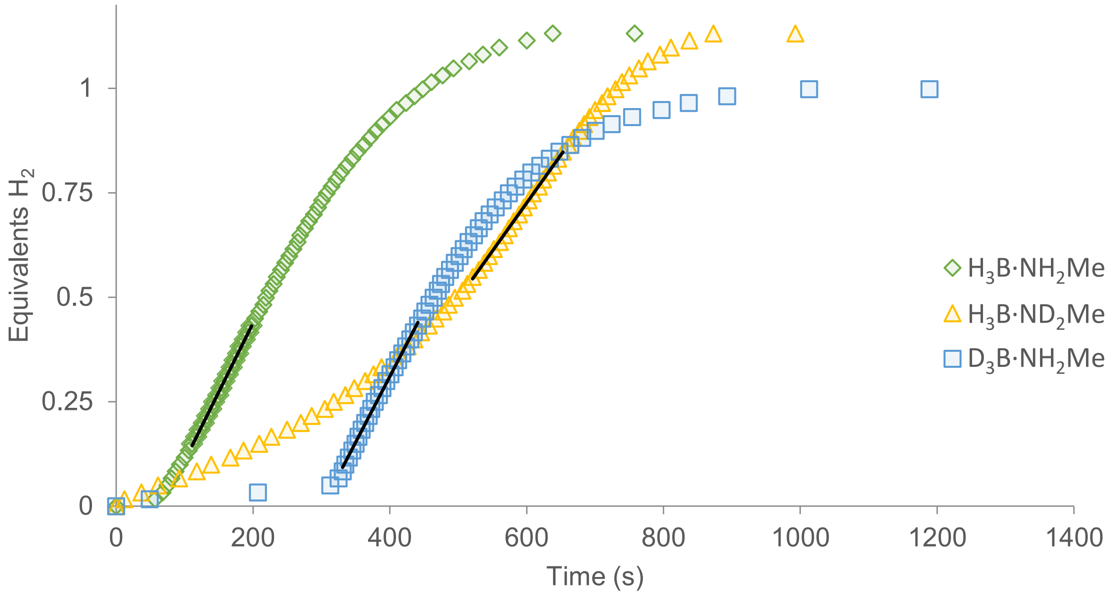


**Figure S17.** Kinetics of the dehydropolymerisation of $H_3B·NH_2Me$ (isotopically substituted) with Ir($^t$Bu-POCOP)$H_2$ (**1**). Conditions: 2 M $H_3B·NH_2Me$, 2 mM cat., THF, 293 K.

**Table S9.** Effect of isotope substitution on dehydropolymerisation with Ir($^t$Bu-POCOP)$H_2$ (**1**). Conditions: 2 M $H_3B·NH_2Me$, 2 mM (0.1 mol%) of Ir($^t$Bu-POCOP)$H_2$, 293 K, THF. [a]Determined by the pseudo zero order region of the profile [b]Measured by $^{11}$B NMR spectroscopy [c]Relative to polystyrene standards.

| Entry | Substrate | Rate[a] (mM s$^{-1}$) | Induction period (s) | Conv.[b] (%) | Selectivity[b] (%) | $M_n$[c] (g mol$^{-1}$) | Đ[c] | Yield (mg (%)) |
|---|---|---|---|---|---|---|---|---|
| 1 | $H_3B·NH_2Me$ | 6.6(2) | 57 | >99 | >99 | 107,100 | 1.4 | 61 (54) |
| 2 | $D_3B·NH_2Me$ | 6.4(3) | 325 | >99 | >99 | 60,500 | 1.3 | 67 (56) |
| 3 | $H_3B·ND_2Me$ | 4.6(1) | 0 | >99 | >99 | 213,900 | 1.8 | 35 (30) |

### 1.5 Kinetic Analysis for the dehydrocoupling reaction of $H_3B{\cdot}NH_2Me$ with Ir($^t$Bu-POCOP)$H_2(BH_3)$ (3)

Eudiometric measurements of hydrogen gas production were performed using an upturned burette filled with water that was displaced as hydrogen gas was produced.

Mono-methyl amine-borane ($H_3B{\cdot}NH_2Me$) (112mg, 2.5 mmol) was placed in a two-neck jacketed Schlenk flask with temperature (293 K) maintained by a circulating cooler. The Ir($^t$Bu-POCOP)$H_2(BH_3)$ (0.1 mol%) was dissolved in a known volume of THF separately. The flask was connected to the water filled burette via PTFE tubing. The catalyst solution was added to the jacketed flask and the solution stirred at 400 rpm. The volume and time were recorded at 1 $cm^3$ increments of gas collected, aided by video recording when required. After completion of gas evolution, an in-situ NMR sample (0.5 mL) was taken and analysed. Pentane (50 mL) was added, and the solution stirred rapidly to induce polymer precipitation. The white solid was isolated by filtration and remaining volatiles were removed in vacuo. The polymer was analysed by $^1$H, $^{31}$P{$^1$H} and $^{11}$B NMR spectroscopies and GPC.

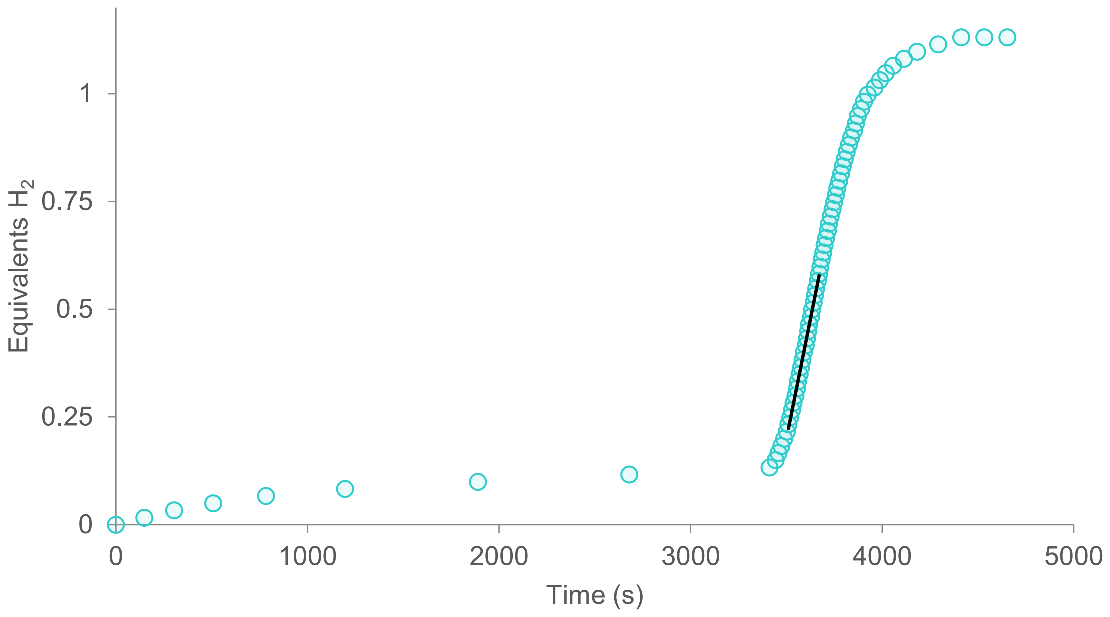


**Figure S18.** Dehydropolymerisation of $H_3B{\cdot}NH_2Me$ with Ir($^t$Bu-POCOP)$H_2(BH_3)$ (**3**) displaying a considerably longer induction period than reaction with Ir($^t$Bu-POCOP)$H_2$ (55 minutes and 2-3 minutes respectively). Conditions: 0.1 mol% catalyst, 2 M $H_3B{\cdot}NH_2Me$, 293 K, 1.25 mL THF.

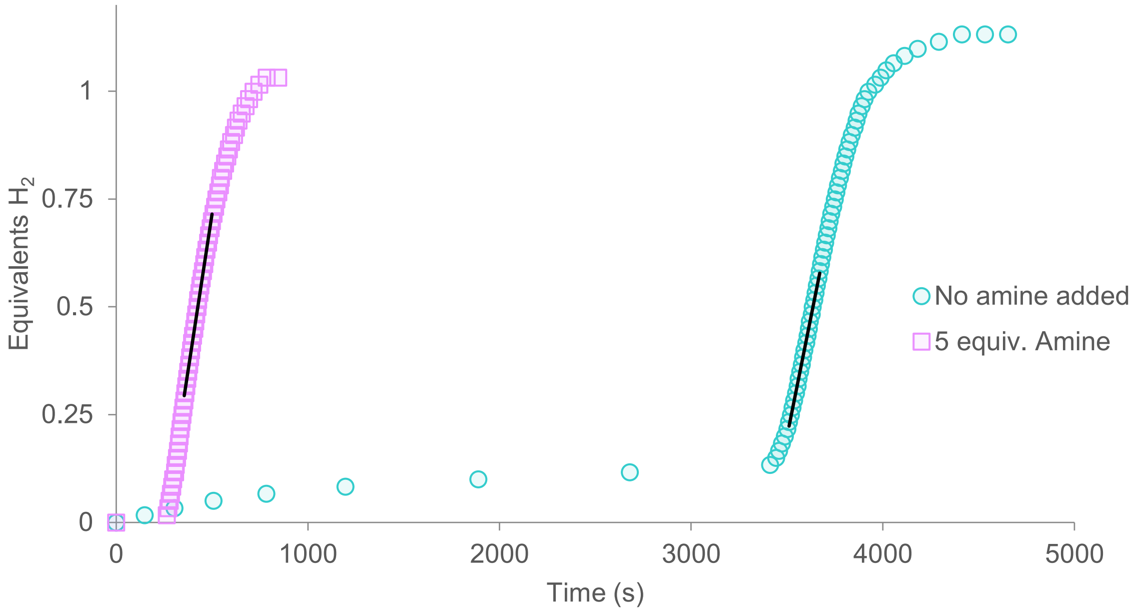


**Figure S19.** Dehydropolymerisation of $H_3B·NH_2Me$ with Ir($^t$Bu-POCOP)$H_2(BH_3)$ (**3**), showing the effect of 5 equivalents of $NH_2Me$ (2 M in THF) on the rate and induction period.

**Table S10.** Effect of $NH_2Me$ (2 M in THF) addition on dehydropolymerisation of $H_3B·NH_2Me$ with Ir($^t$Bu-POCOP)$H_2(BH_3)$ (**3**). Conditions: 2 M $H_3B·NH_2Me$, 2 mM (0.1 mol%), 293 K, THF. [a]Determined by the pseudo zero order region of the profile [b]Measured by $^{11}$B NMR spectroscopy [c]Relative to polystyrene standards.

| Entry | Amine Equiv. | Rate[a] (mM $s^{-1}$) | Induction period (s) | Conv.[b] (%) | Selectivity[b] (%) | $M_n$[c] (g $mol^{-1}$) | Đ[c] | Yield (mg (%)) |
|---|---|---|---|---|---|---|---|---|
| 1 | 0 | 4.44(2) | 3002 | >99 | >99 | 117,300 | 1.4 | 79 (74) |
| 2 | 5 | 6.55(2) | 272 | >99 | >99 | 170,500 | 1.4 | 60 (56) |

## 1.6 Kinetic analysis for the dehydropolymerisation of $H_3B{\cdot}NH_2Me$ with Ir($^tBu$-POCOP)$H_4$ (2)

Eudiometric measurements of hydrogen gas production were performed using an upturned burette filled with water that was displaced as hydrogen gas was produced.

Mono-methyl amine-borane ($H_3B{\cdot}NH_2Me$) (112mg, 2.5 mmol) was placed in a two-neck jacketed Schlenk flask with temperature (293 K) maintained by a circulating cooler. The Ir($^tBu$-POCOP)$H_4$ (0.1 mol%) was formed by taking Ir($^tBu$-POCOP)$H_2$ in a known volume of THF and putting it under $H_2$ (the solution turned colourless). The flask was connected to the water filled burette via PTFE tubing. The catalyst solution was added to the jacketed flask and the solution stirred at 400 rpm. The volume and time were recorded at 1 $cm^3$ increments of gas collected, aided by video recording when required. After completion of gas evolution, an in-situ NMR sample (0.5 mL) was taken and analysed. Pentane (50 mL) was added, and the solution stirred rapidly to induce polymer precipitation. The white solid was isolated by filtration and remaining volatiles were removed in vacuo. The polymer was analysed by $^1H$, $^{31}P\{^1H\}$ and $^{11}B$ NMR spectroscopies and GPC.

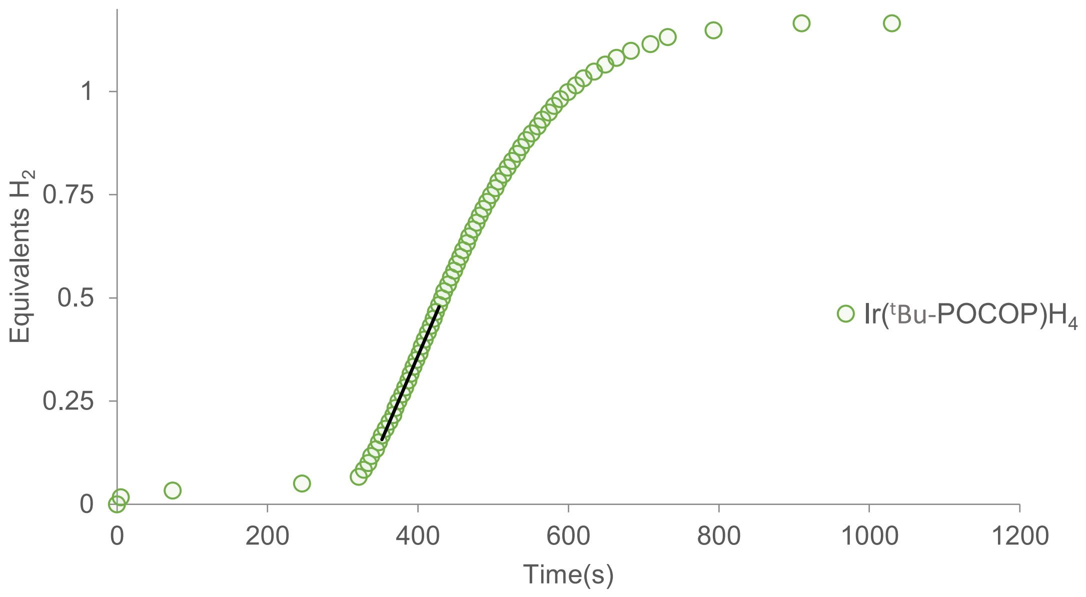


**Figure S20.** Dehydropolymerisation of $H_3B{\cdot}NH_2Me$ with Ir($^tBu$-POCOP)$H_4$ (**2**). Conditions: 2 M $H_3B{\cdot}NH_2Me$, 0.1 mol % catalyst, THF, 293 K.

**Table S11.** Effect of $NH_2Me$ (2 M in THF) addition on dehydropolymerisation of $H_3B{\cdot}NH_2Me$ with Ir($^tBu$-POCOP)$H_4$ (**2**). Conditions: 2 M $H_3B{\cdot}NH_2Me$, 2 mM (0.1 mol%), 293 K, THF. [a]Determined by the pseudo zero order region of the profile [b]Measured by $^{11}B$ NMR spectroscopy [c]Relative to polystyrene standards.

| Entry | Amine Equiv. | Rate[a] (mM $s^{-1}$) | Induction period (s) | Conv.[b] (%) | Selectivity[b] (%) | $M_n$[c] (g $mol^{-1}$) | Đ[c] | Yield (mg (%)) |
|---|---|---|---|---|---|---|---|---|
| 1 | 0 | 8.47(7) | 328 | >99 | >99 | 119,100 | 1.6 | 60 (56) |

### 1.7 Kinetic analysis for the dehydropolymerisation of $H_3B{\cdot}NH_2Me$ with $[Ir(^tBu\text{-}POCOP)H_3][Na(18\text{-}crown\text{-}6)(THF)_2]$ (6)

Eudiometric measurements of hydrogen gas production were performed using an upturned burette filled with water that was displaced as hydrogen gas was produced.

Mono-methyl amine-borane ($H_3B{\cdot}NH_2Me$) (112mg, 2.5 mmol) was placed in a two-neck jacketed Schlenk flask with temperature (293 K) maintained by a circulating cooler. In the experiments where $[[NH_3Me]\ [BAr^F{}_4]$ was added this was added to the two-neck jacketed Schlenk flask too. The $[Ir(^tBu\text{-}POCOP)H_3][Na\text{-}18\text{-}crown\text{-}6\text{-}(THF)_2]$ (0.1 mol%) was dissolved in a known volume of THF separately. The flask was connected to the water filled burette via PTFE tubing. The catalyst solution was added to the jacketed flask and the solution stirred at 400 rpm. The volume and time were recorded at 1 $cm^3$ increments of gas collected, aided by video recording when required. After completion of gas evolution, an in-situ NMR sample (0.5 mL) was taken and analysed. Pentane (50 mL) was added, and the solution stirred rapidly to induce polymer precipitation. The white solid was isolated by filtration and remaining volatiles were removed in vacuo. The polymer was analysed by $^1H$, $^{31}P\{^1H\}$ and $^{11}B$ NMR spectroscopies and GPC.

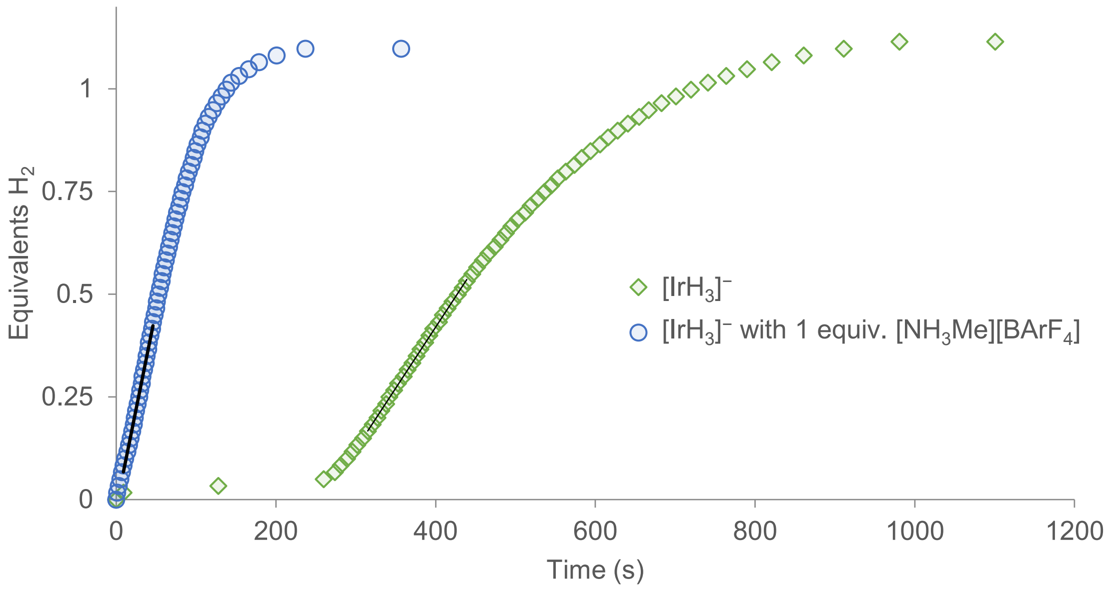


**Figure S21.** Dehydropolymerisation of $H_3B{\cdot}NH_2Me$ with $[Ir(^tBu\text{-}POCOP)H_3][Na\text{-}18\text{-}crown\text{-}6\text{-}(THF)_2]$, showing the effect of addition $[NH_3Me][BAr^F{}_4]$ (1 equivalent with regard to catalyst). Conditions: 2 M $H_3B{\cdot}NH_2Me$, 0.1 mol % catalyst, THF, 293 K.

**Table 12.** Effect of $[NH_3Me][BAr^F{}_4]$ addition on dehydropolymerisation of $H_3B{\cdot}NH_2Me$ with $[Ir(^tBu\text{-}POCOP)H_3]^-$. Conditions: 2 M $H_3B{\cdot}NH_2Me$, 2 mM (0.1 mol%), 293 K, THF. [a]Determined by the pseudo zero order region of the profile [b]Measured by $^{11}B$ NMR spectroscopy [c]Relative to polystyrene standards.

| Entry | $[NH_3Me]$ $[BAr^F{}_4]$ Equiv. | Rate[a] (mM $s^{-1}$) | Induction period (s) | Conv.[b] (%) | Selectivity[b] (%) | $M_n$[c] (g $mol^{-1}$) | Đ[c] | Yield (mg (%)) |
|---|---|---|---|---|---|---|---|---|
| 1 | 0 | 5.97(3) | 274 | >99 | >99 | 95,600 | 1.4 | 67 (63) |
| 2 | 1 | 19.2(3) | 0 | >99 | >99 | 185,200 | 1.4 | 63 (59) |

## 1.8 Catalyst speciation during the dehydropolymerisation of $H_3B \cdot NH_2Me$

General procedure for *in situ* NMR speciation experiments:

In a typical experiment, mono-methyl amine-borane ($H_3B \cdot NH_2Me$) (112mg, 2.5 mmol) was placed in a two-neck jacketed Schlenk flask with temperature (283 K) maintained by a circulating cooler. The Ir($^t$Bu-POCOP)$H_2$ (0.1 mol%) was dissolved in a known volume of THF separately. The flask was connected to the water filled burette via PTFE tubing. The catalyst solution was added to the jacketed flask and the solution stirred at 400 rpm. The reactions were allowed to run to the desired point and were transferred into NMR tubes that were cooled to −94 °C to thermally quench the reaction and stop hydrogen production. The NMR tubes were then stored in liquid nitrogen until thawed in liquid nitrogen/ethyl acetate and put into a pre-cooled spectrometer at 198 K for analysis by NMR spectroscopies. An additional experiment was carried out at 0.3 mol% (**1**) to investigate the induction period with better signal enhancement. The reaction was carried out at 283 K and was sampled at after 100 s (Figure S22).

CAUTION: $H_2$ production at room temperature is fast. To limit the risk of explosion, NMR experiments were carried out in high pressure NMR tubes rated up to 12 bar pressure, the taps were fitted with HPLC tubing that was connected to a 5 bar pressure release valve as a safety precaution. The NMR tube, HPLC tubing and pressure release valve were all under argon atmosphere.

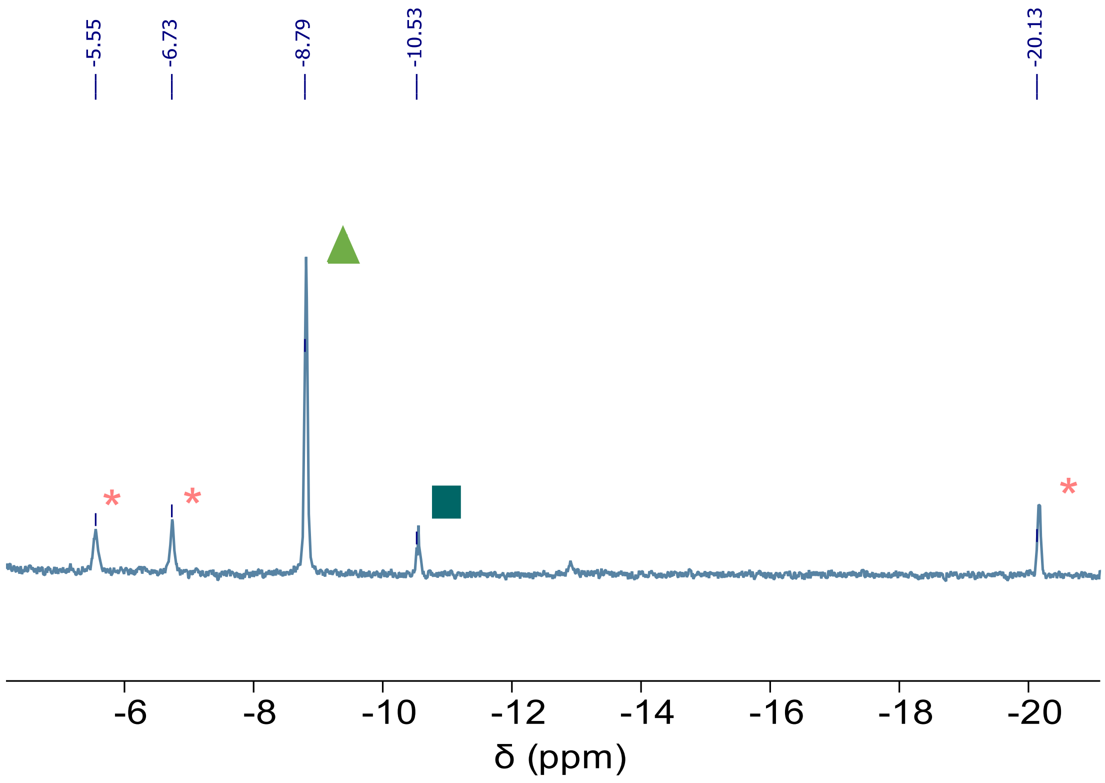


**Figure S22.** $^1$H NMR spectrum (THF-$H^8$, 500 MHz, 198 K) taken of the hydride region from the reaction mixture of $H_3B \cdot NH_2Me$ and Ir($^t$Bu-POCOP)$H_2$ taken after 100 s. Reaction conditions: 2 M $H_3B \cdot NH_2Me$, 0.3 mol% catalyst, 283 K. * Ir($^t$Bu-POCOP)(H)($BH_4$), ▲ Ir($^t$Bu-POCOP)$H_4$, ■ unknown.

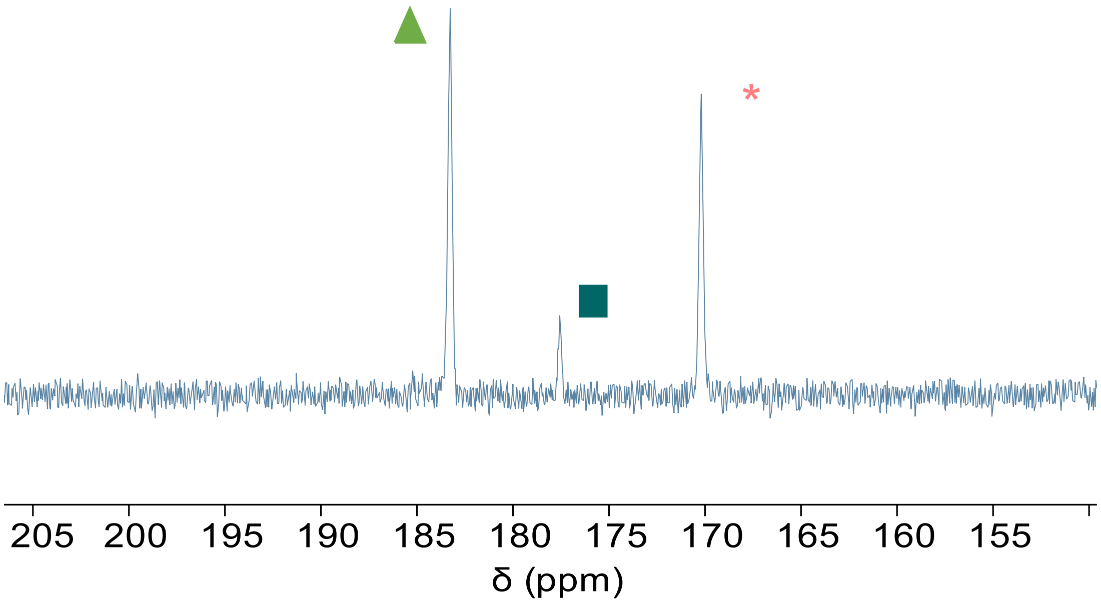


**Figure S23.** $^{31}P\ \{^1H\}$ NMR spectrum (THF-$H^8$, 203 MHz, 198 K) from the reaction mixture of $H_3B{\cdot}NH_2Me$ and Ir($^tBu$-POCOP)$H_2$ taken after 100 s. Reaction conditions: 2 M $H_3B{\cdot}NH_2Me$, 0.3 mol% catalyst, 283 K. * Ir($^tBu$-POCOP)(H)($BH_4$), ▲ Ir($^tBu$-POCOP)$H_4$, ■ unknown.

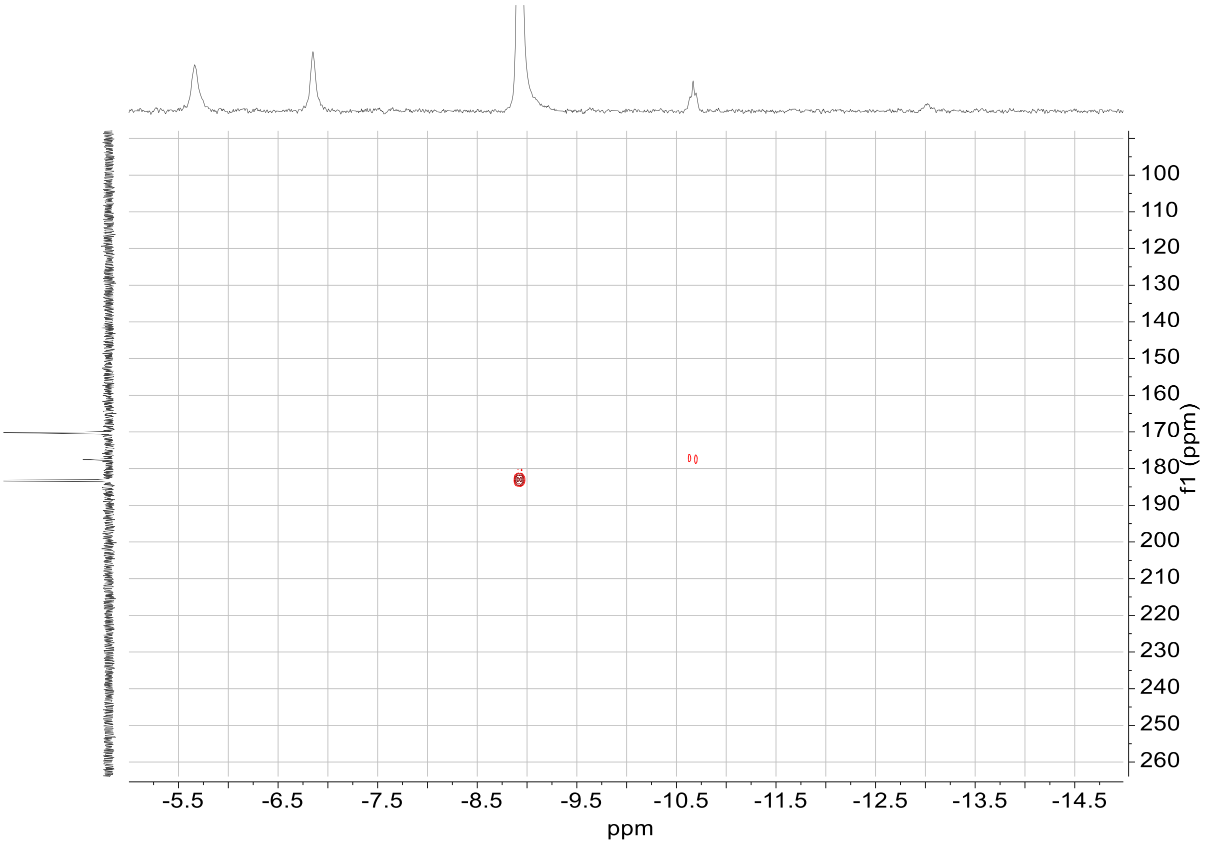


**Figure S24.** $^1H$-$^{31}P$ HMBC spectrum (THF-$H^8$, 500/202 MHz, 198 K) from the reaction mixture of $H_3B{\cdot}NH_2Me$ and Ir($^tBu$-POCOP)$H_2$ taken during the induction period, showing correlation between the hydride peak at −10.6 ppm and the $^{31}P$ signal at 177 ppm.

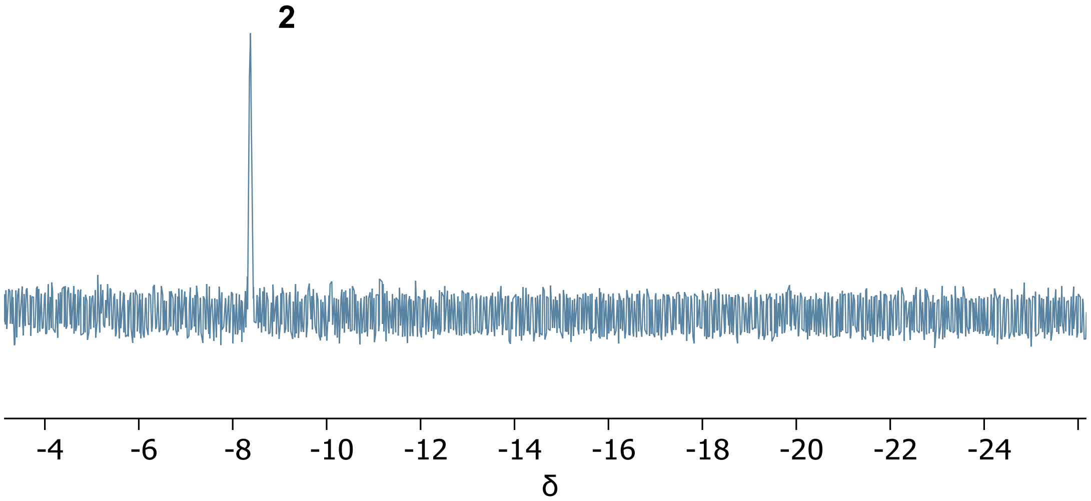


**Figure S25.** $^{1}H$ NMR spectrum (THF-$H^8$, 500 MHz, 198 K) taken of the hydride region from the reaction mixture of $H_3B{\cdot}NH_2Me$ and Ir($^{t}$Bu-POCOP)$H_2$ taken after ~0.25 equivalents of hydrogen produced. Reaction conditions: 2 M $H_3B{\cdot}NH_2Me$, 0.1 mol% catalyst, 283 K.

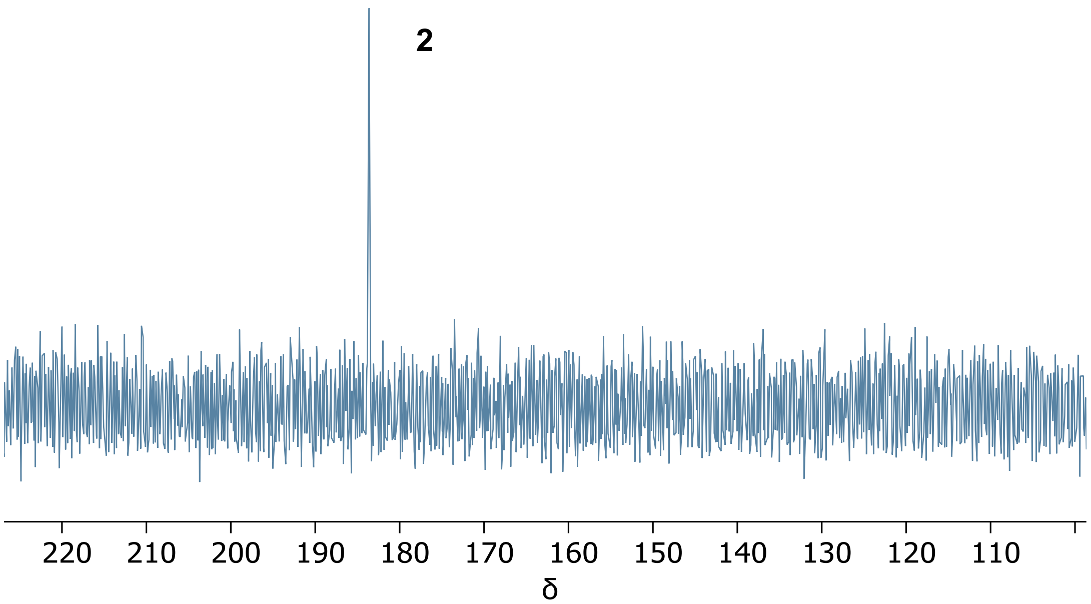


**Figure S26.** $^{31}P\{^{1}H\}$ NMR spectrum (THF-$H^8$, 203 MHz, 198 K) from the reaction mixture of $H_3B{\cdot}NH_2Me$ and Ir($^{t}$Bu-POCOP)$H_2$ taken after ~0.25 equivalents of hydrogen produced. Reaction conditions: 2 M $H_3B{\cdot}NH_2Me$, 0.1 mol% catalyst, 283 K.

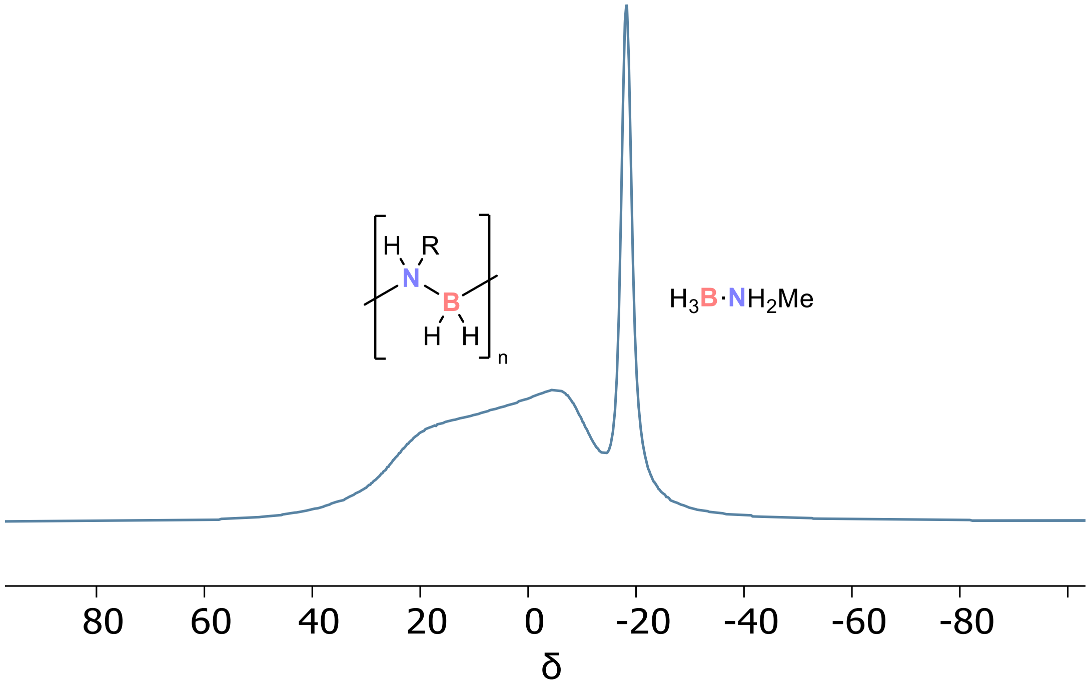


**Figure S27.** $^{11}B$ NMR spectrum (THF-H[8], 160 MHz, 198 K) from the reaction mixture of $H_3B{\cdot}NH_2Me$ and Ir($^tBu$-POCOP)$H_2$ taken after ~0.25 equivalents of hydrogen produced. Reaction conditions: 2 M $H_3B{\cdot}NH_2Me$, 0.1 mol% catalyst, 283 K. The polymer peak at this temperature is very broad and so cannot be deconvoluted from the signal from the glass.

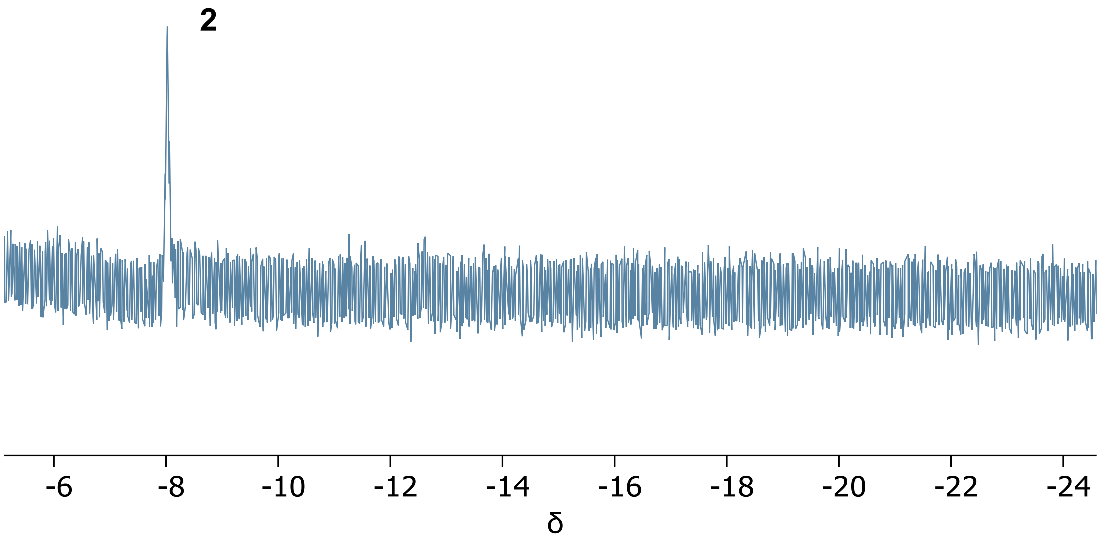


**Figure S28.** $^1H$ NMR spectrum (THF-H[8], 500 MHz, 198 K) taken of the hydride region from the reaction mixture of $H_3B{\cdot}NH_2Me$ and Ir($^tBu$-POCOP)$H_2$ taken after ~0.75 equivalents of hydrogen produced. Reaction conditions: 2 M $H_3B{\cdot}NH_2Me$, 0.1 mol% catalyst, 283 K.

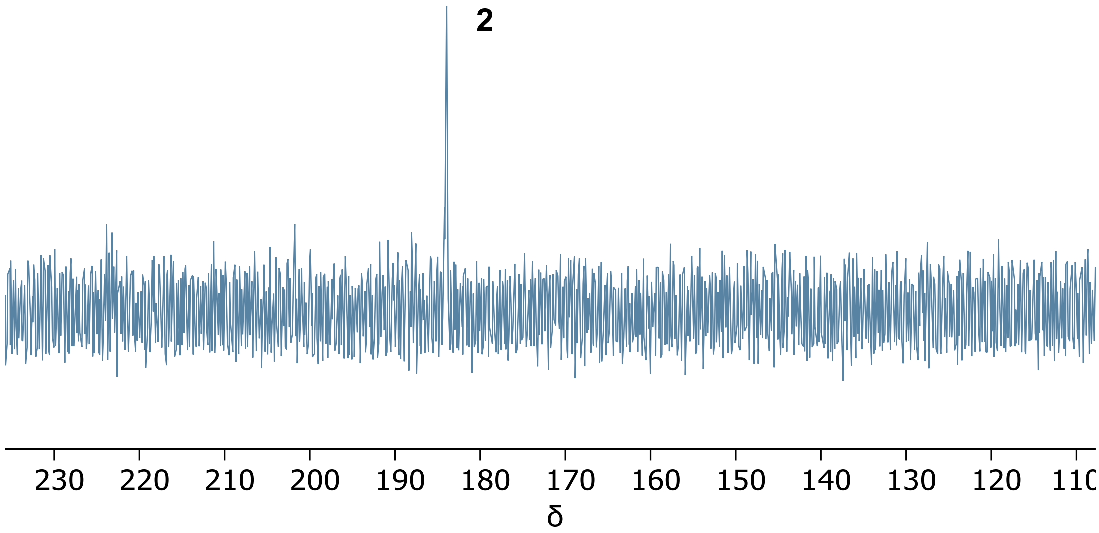


**Figure S29.** $^{31}P\{^{1}H\}$ NMR spectrum (THF-$H^8$, 203 MHz, 198 K) from the reaction mixture of $H_3B{\cdot}NH_2Me$ and Ir($^t$Bu-POCOP)$H_2$ taken after ~0.75 equivalents of hydrogen produced. Reaction conditions: 2 M $H_3B{\cdot}NH_2Me$, 0.1 mol% catalyst, 283 K.

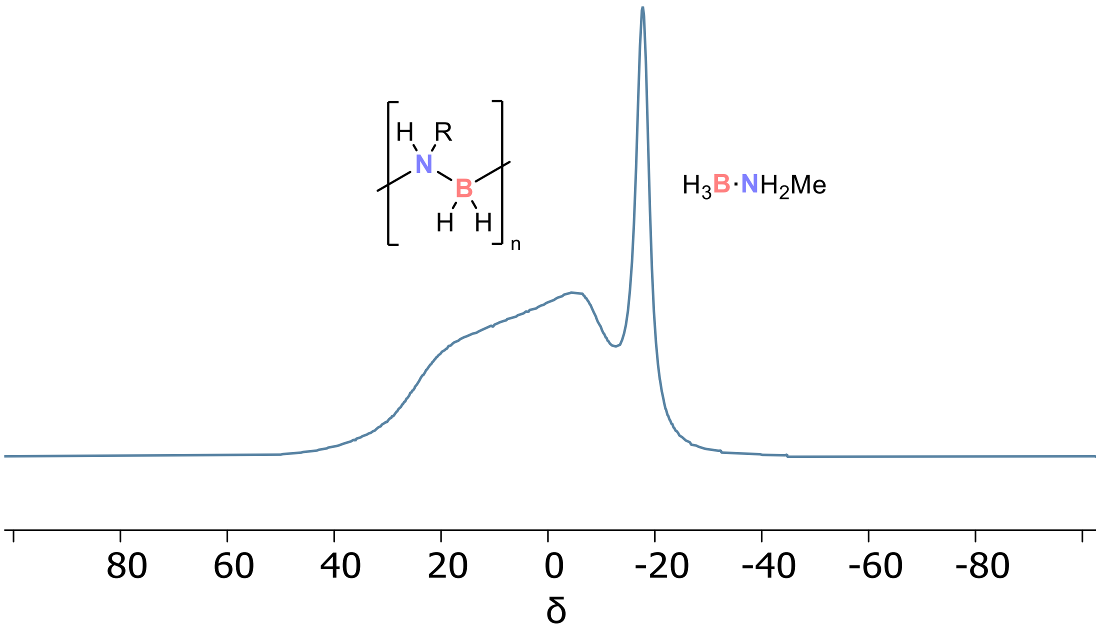


**Figure S30.** $^{11}B$ NMR spectrum (THF-$H^8$, 160 MHz, 198 K) from the reaction mixture of $H_3B{\cdot}NH_2Me$ and Ir($^t$Bu-POCOP)$H_2$ taken after ~0.75 equivalents of hydrogen produced. Reaction conditions: 2 M $H_3B{\cdot}NH_2Me$, 0.1 mol% catalyst, 283 K. The polymer peak at this temperature is very broad and so cannot be deconvoluted from the signal from the glass.

## 2 NMR Spectra

### 2.1 Ir($^t$Bu-POCOP)$H_2$($NH_2$Me)

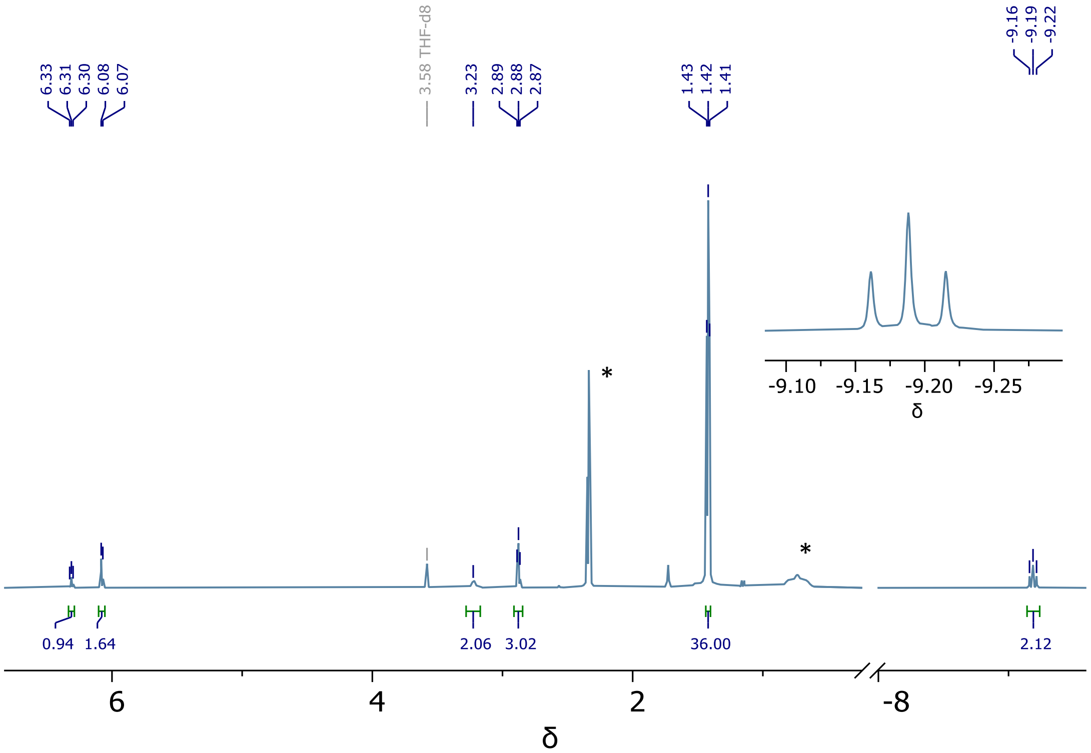


**Figure S31.** $^1$H NMR spectrum (THF-d$^8$, 600 MHz, 298 K) of Ir($^t$Bu-POCOP)$H_2$($NH_2$Me) – $NH_2$Me gas dissolved in THF indicated by *.

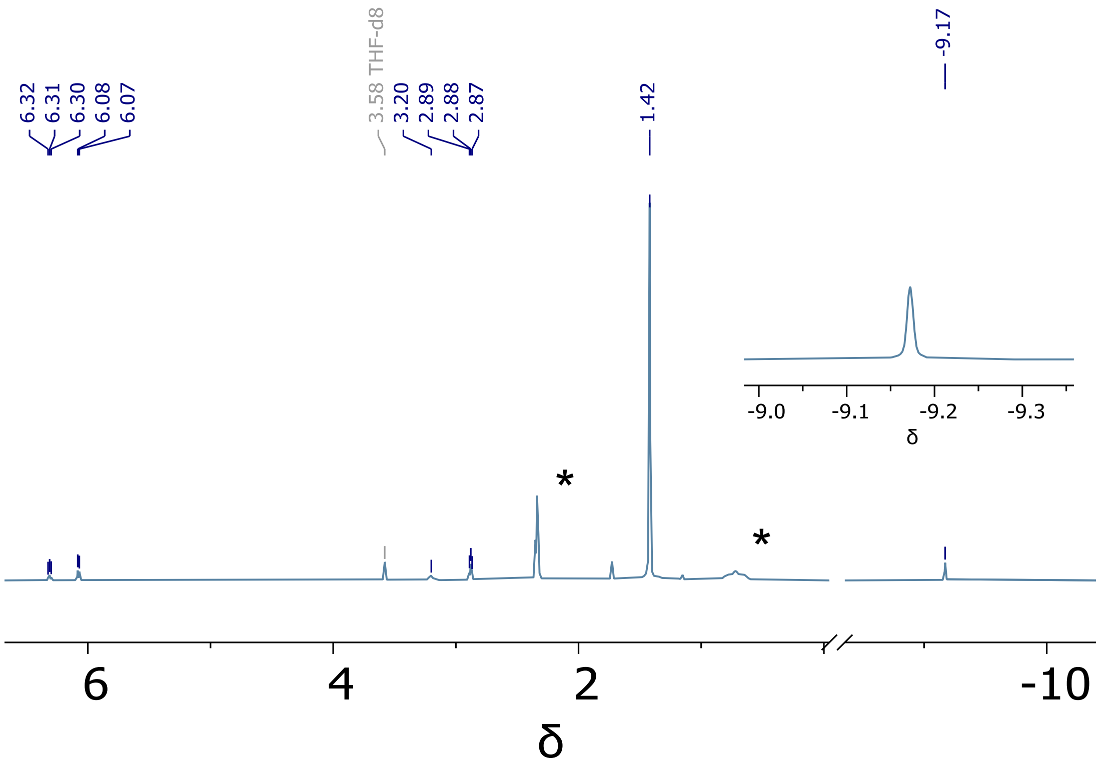


**Figure S32.** $^1$H{$^{31}$P} NMR spectrum (THF-d$^8$, 600 MHz, 298 K) of Ir($^t$Bu-POCOP)$H_2$($NH_2$Me) –$NH_2$Me gas dissolved in THF indicated by *.

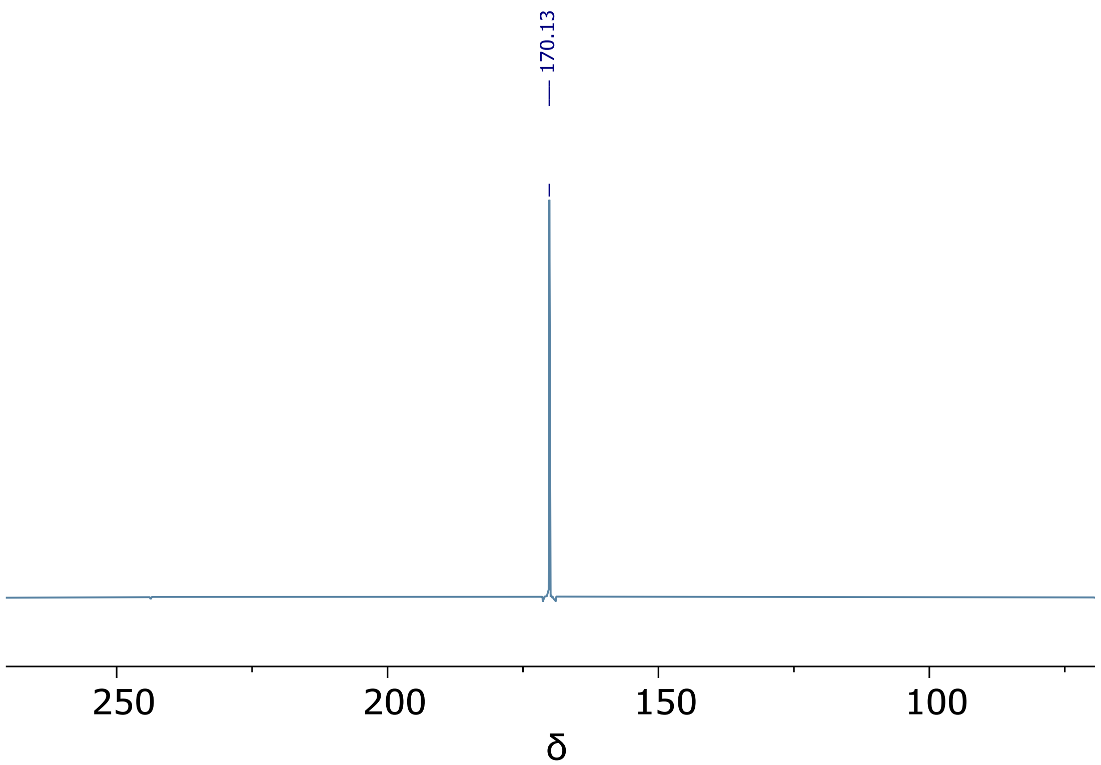


**Figure S33.** $^{31}P\{^{1}H\}$ NMR spectrum (THF-d$^{8}$, 243 MHz, 298 K) of Ir($^{t}$Bu-POCOP)$H_2$($NH_2$Me).

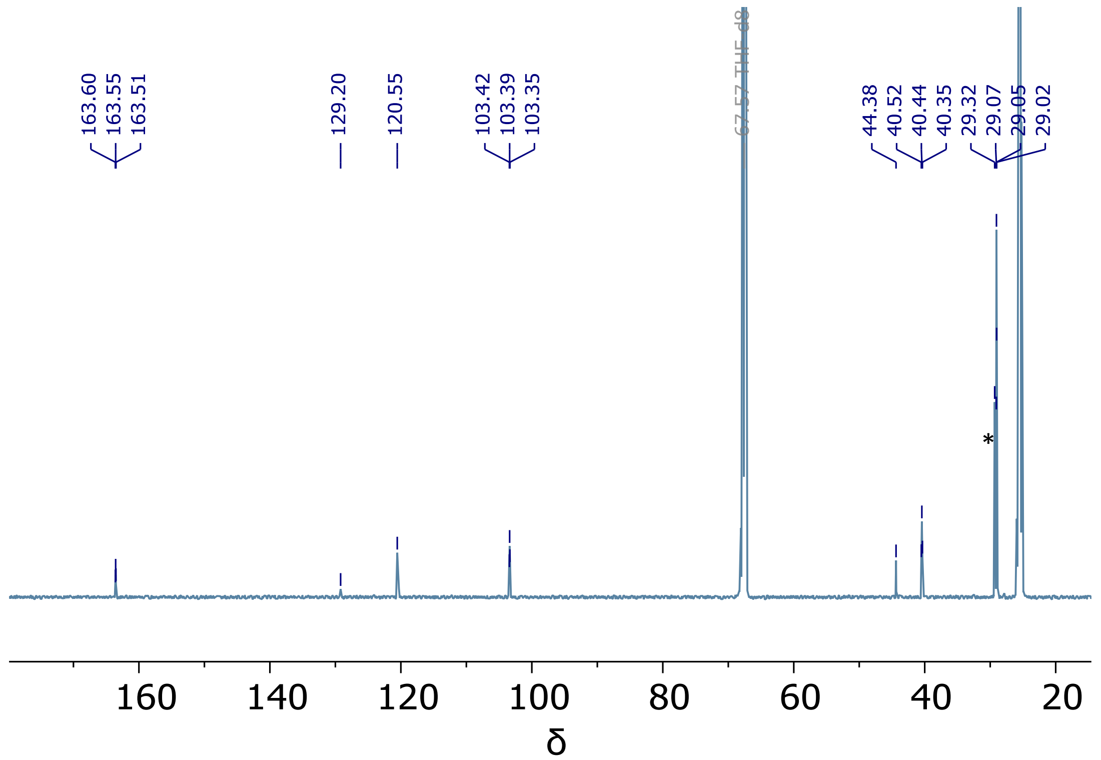


**Figure S34.** $^{13}C\{^{1}H\}$ NMR spectrum (THF-d$^{8}$, 151 MHz, 298 K) of Ir($^{t}$Bu-POCOP)$H_2$($NH_2$Me) –$NH_2$Me gas dissolved in THF indicated by *.

**2.2 [Ir($^{t}$Bu-POCOP)$H_3$][Na(18-crown-6)(THF$_2$)]**

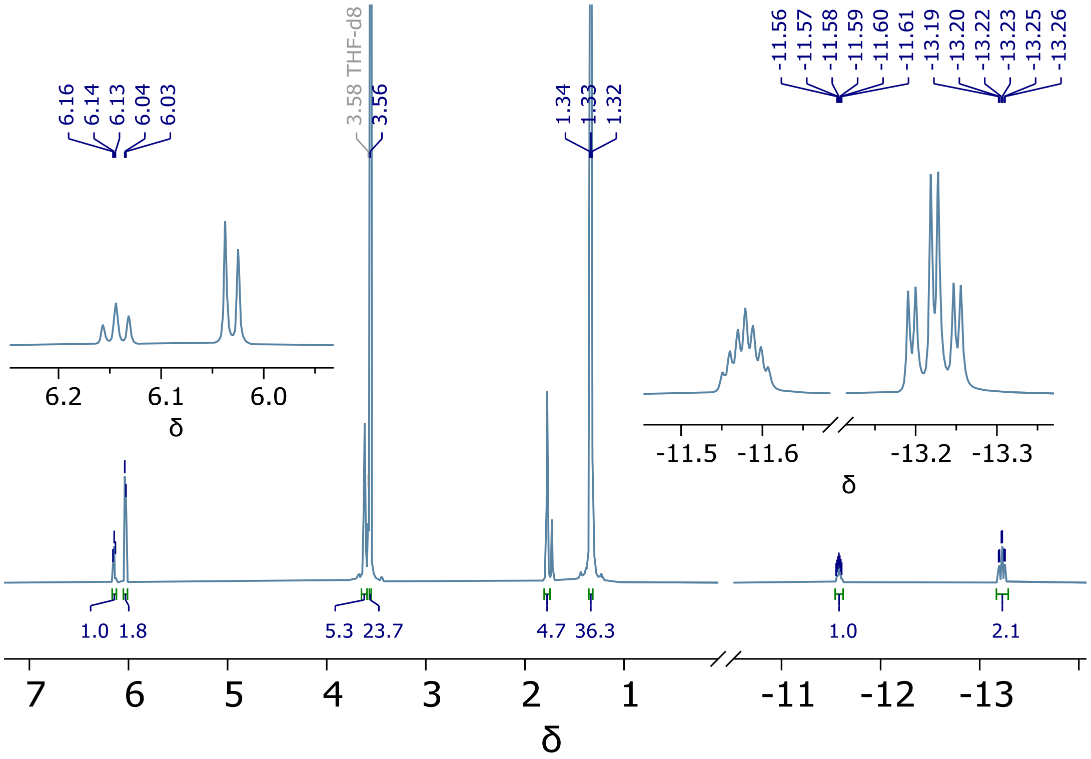


**Figure S35.** $^{1}H$ NMR spectrum (THF-d$^{8}$, 600 MHz, 298 K) of [Ir($^{t}$Bu-POCOP)$H_3$][Na(18-crown-6)(THF)$_2$].

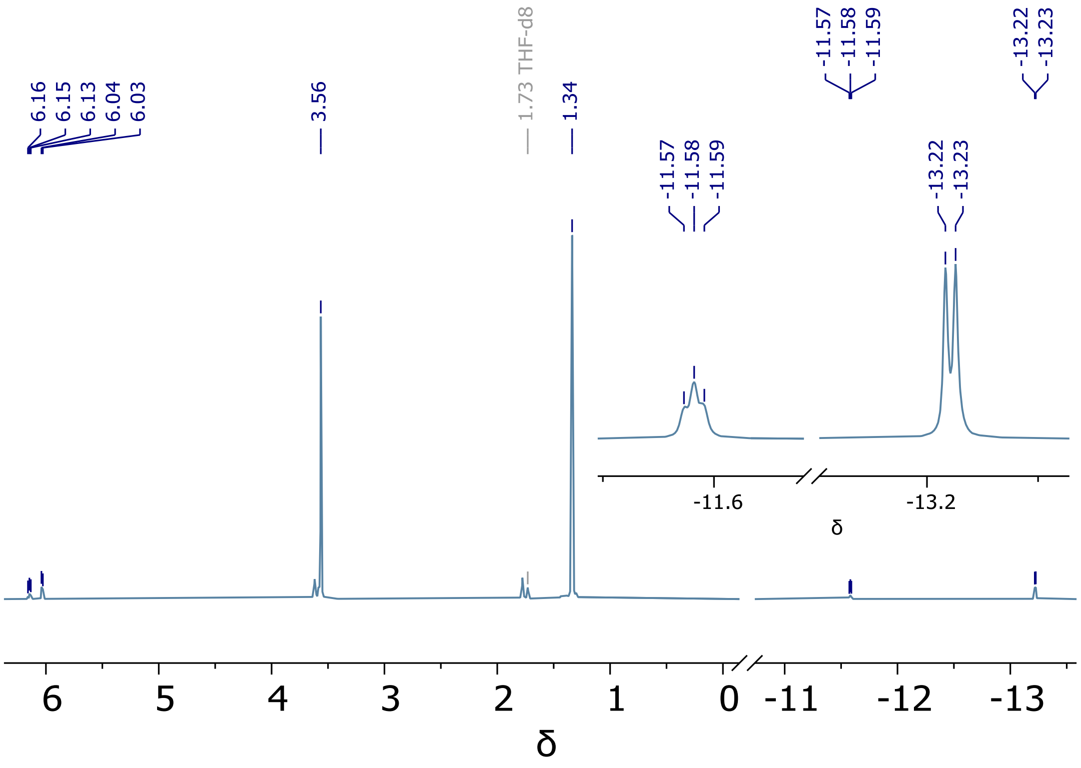


**Figure S36.** $^{1}H\{^{31}P\}$ NMR spectrum (THF-d$^{8}$, 600 MHz, 298 K) of [Ir($^{t}$Bu-POCOP)$H_3$][Na(18-crown-6)(THF)$_2$].

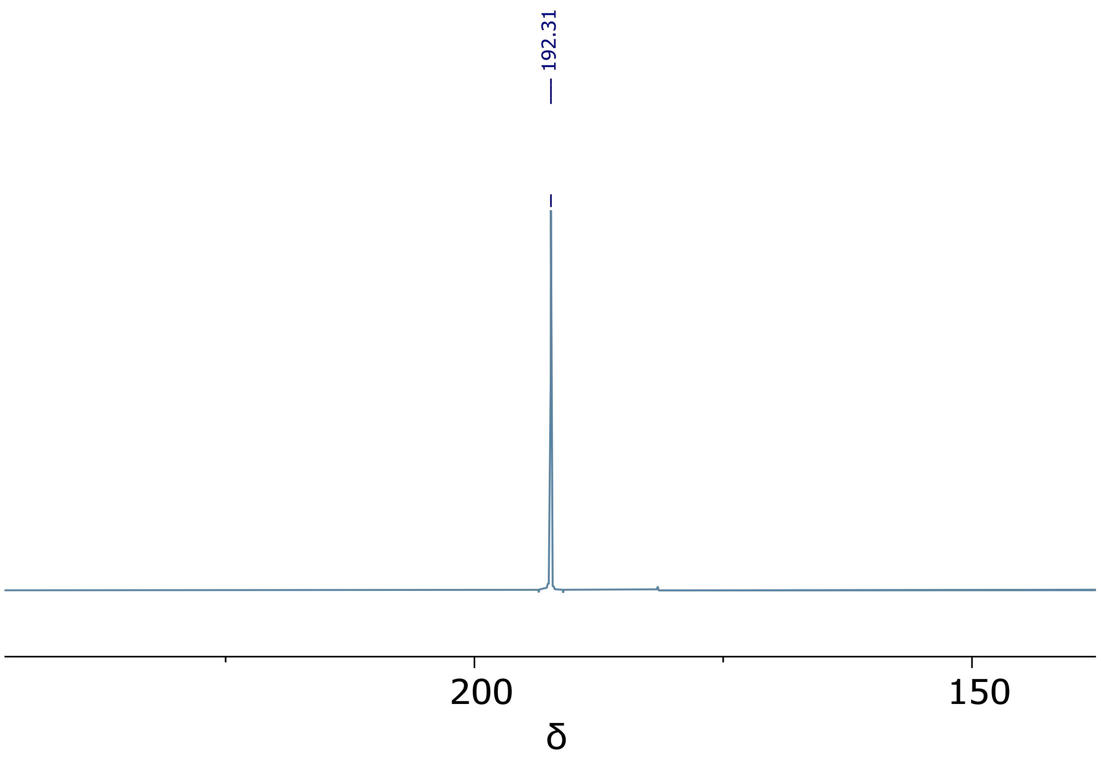


**Figure S37.** $^{31}P\{^{1}H\}$ NMR spectrum (THF-d[8], 243 MHz, 298 K) of [Ir($^{t}$Bu-POCOP)$H_3$][Na(18-crown-6)(THF)$_2$].

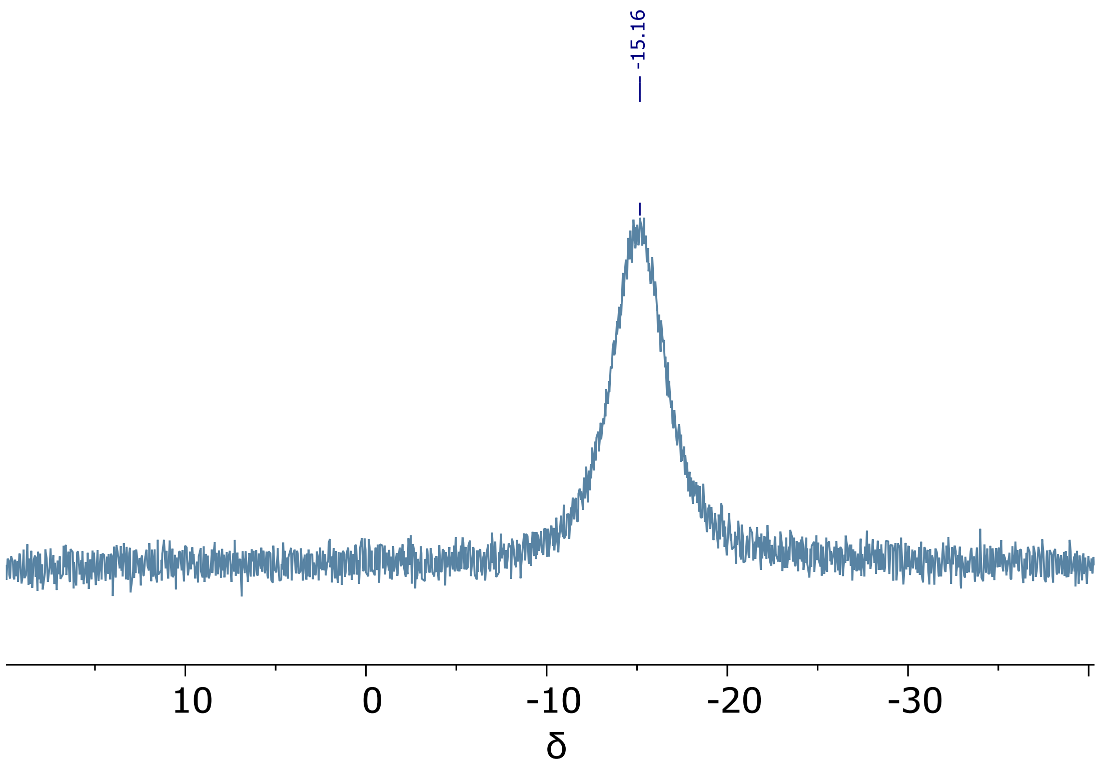


**Figure S38.** $^{23}Na$ NMR spectrum (THF-d[8], 159 MHz, 298 K) of [Ir($^{t}$Bu-POCOP)$H_3$][Na(18-crown-6)(THF)$_2$].

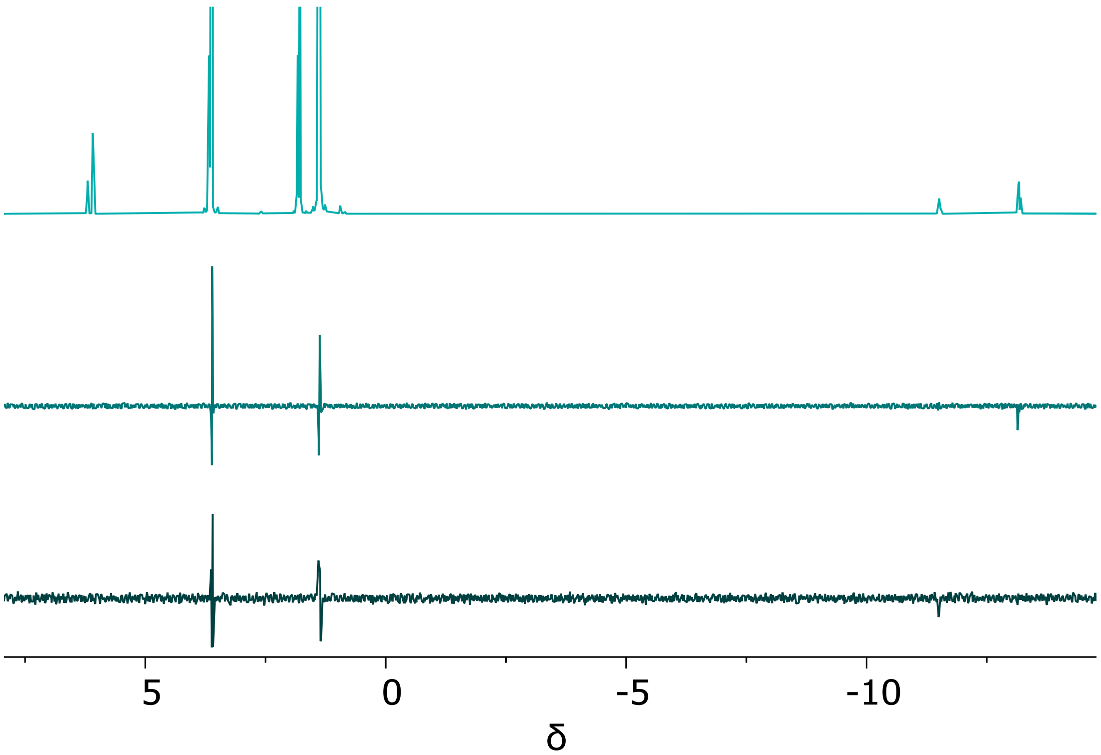


**Figure S39.** Stacked $^{1}H$ NMR spectra (THF-$d^8$, 500 MHz, 298 K) of [Ir($^{t}$Bu-POCOP)$H_3$][Na(18-crown-6)(THF)$_2$] showing correlation between the hydride signals and crown protons, $^{1}H$ NMR spectrum (top), 1D selective ROESY excited at δ −13.2 hydride (middle), 1D selective ROESY excited at δ −11.6 hydride (bottom).

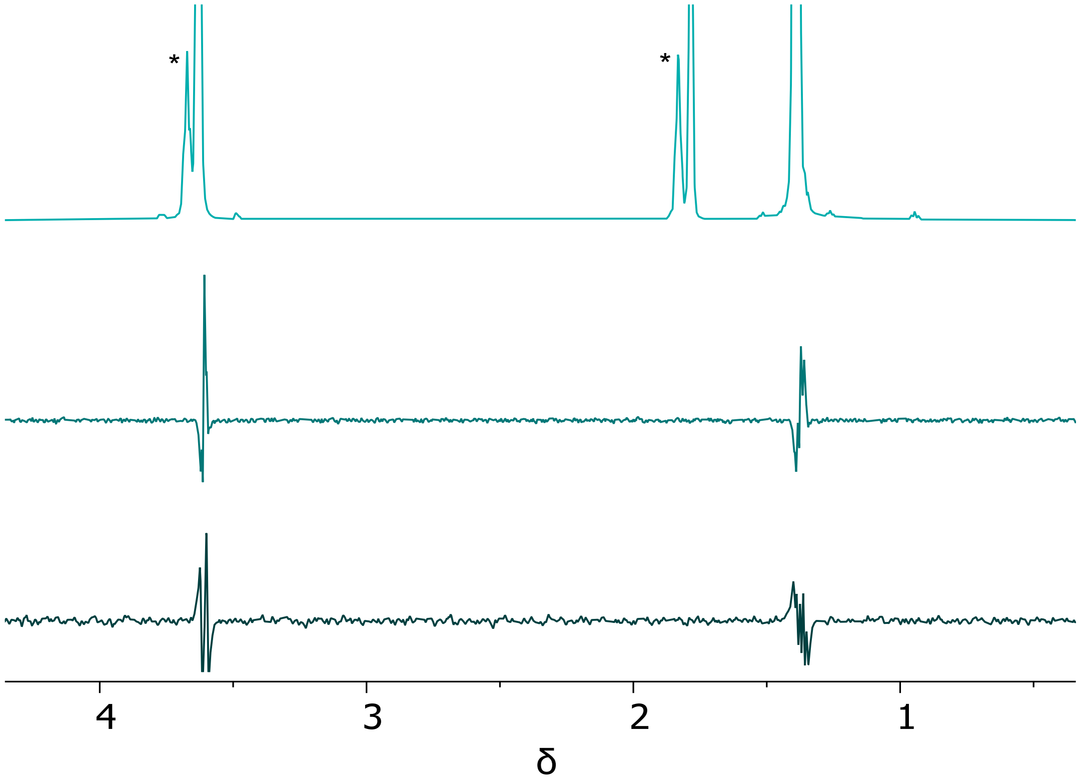


**Figure S40.** Expansion of Figure S39 showing correlation to crown and $^{t}$Bu signals. * Indicates crown bound THF.

## 2.3 *In situ* [Ir($^t$Bu-POCOP)$H_3$]Na

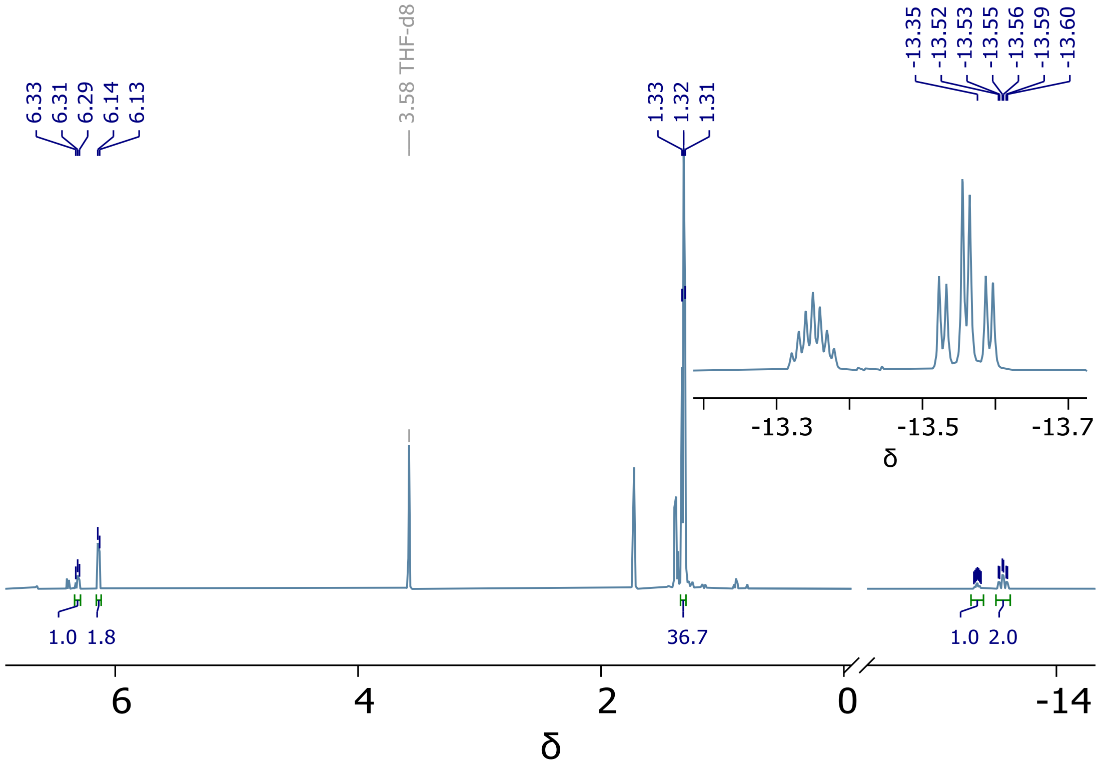


**Figure S41.** $^1$H NMR spectrum (THF-d$^8$, 500 MHz, 298 K) of [Ir($^t$Bu-POCOP)$H_3$]Na made *in-situ*.

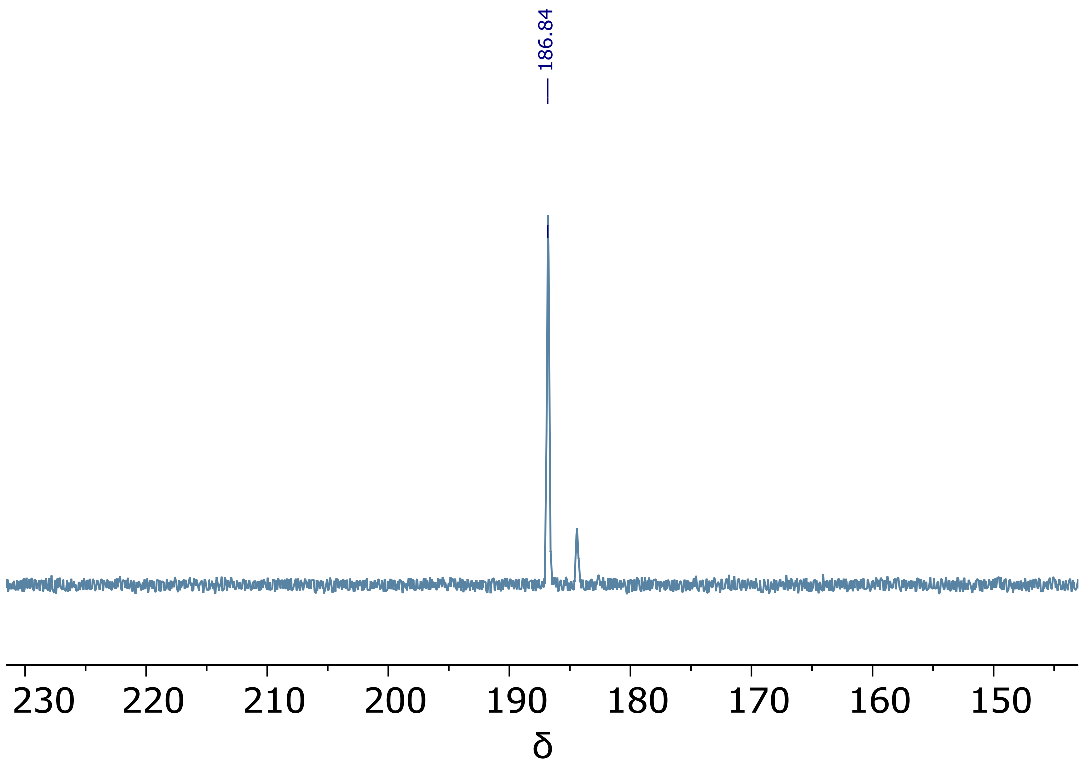


**Figure S42.** $^{31}$P {$^1$H} NMR spectrum (THF-d$^8$, 203 MHz, 298 K) of [Ir($^t$Bu-POCOP)$H_3$]Na made *in-situ*.

**2.4 $[NH_3Me][BAr^F_4]$**

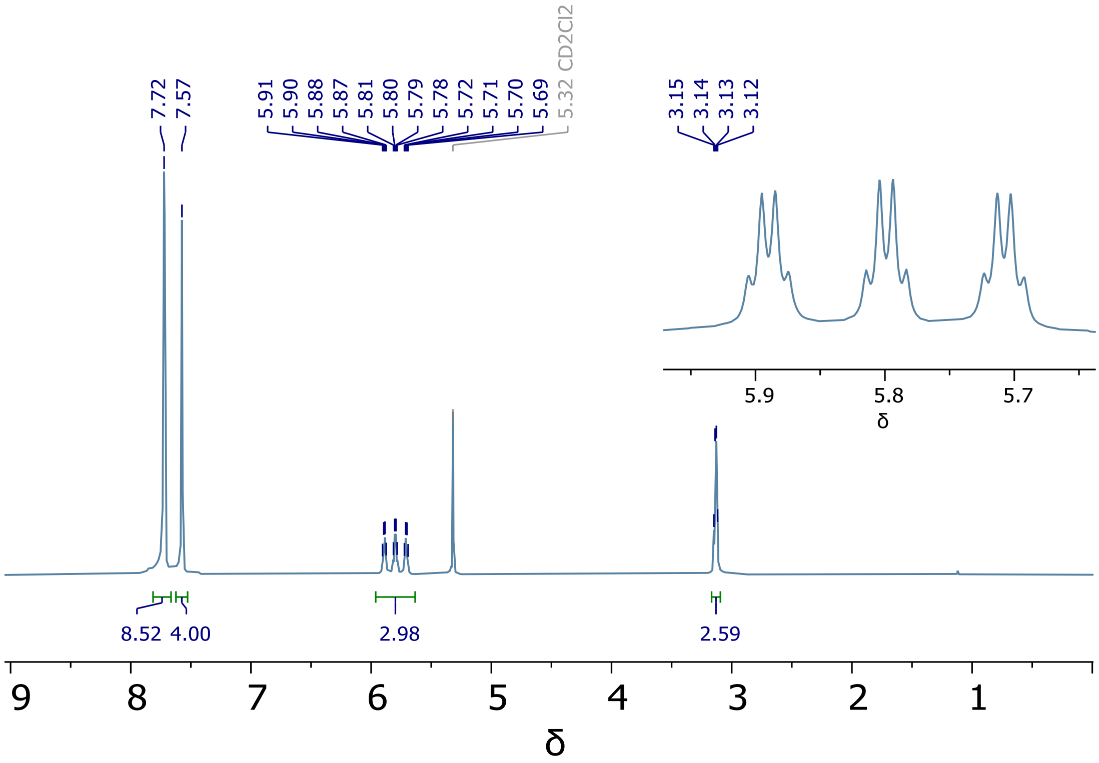


**Figure S43.** $^1H$ NMR spectrum (DCM-d$^2$, 600 MHz, 298 K) of $[NH_3Me][BAr^F_4]$.

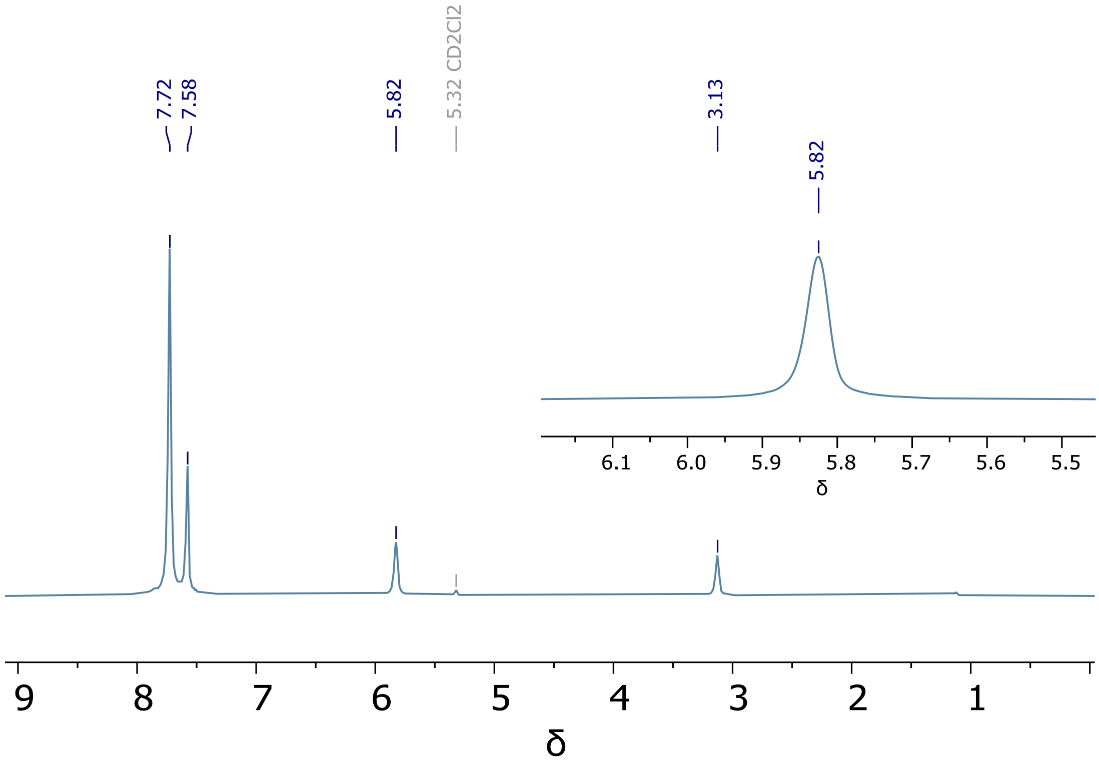


**Figure S44.** $^1H\{^{14}N\}$ NMR spectrum (DCM-d$^2$, 600 MHz, 298 K) of $[NH_3Me][BAr^F_4]$.

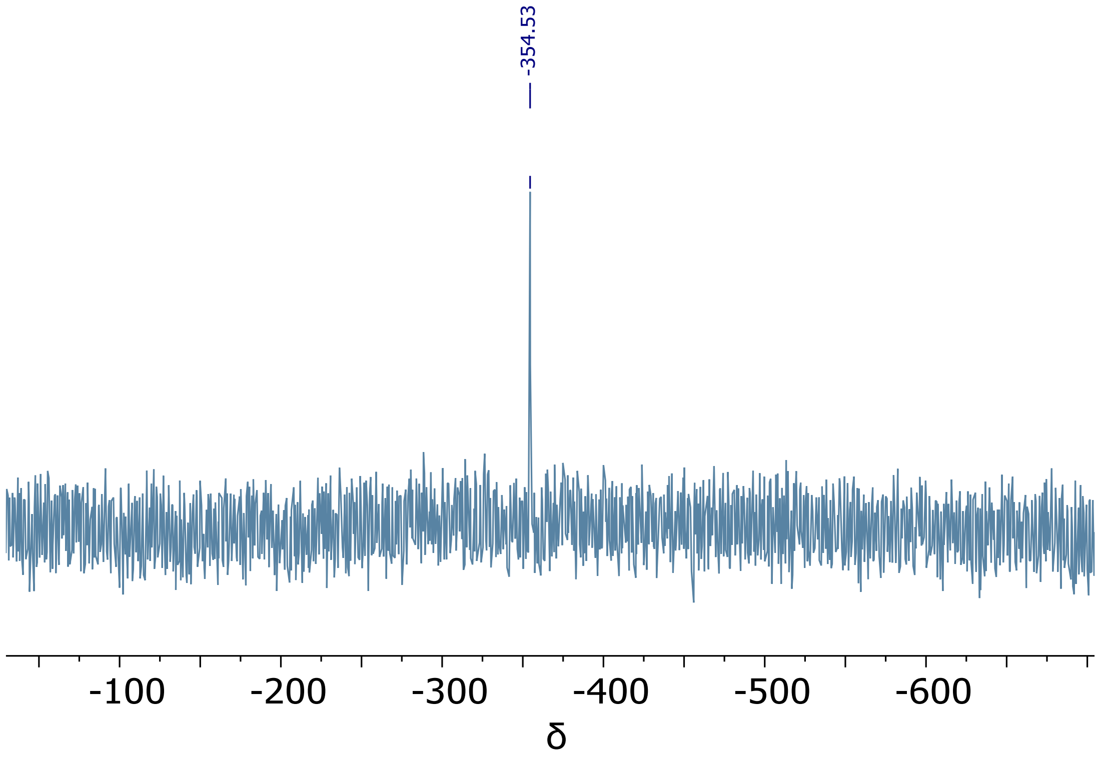


**Figure S45.** $^{14}N\{^{1}H\}$ NMR spectrum (DCM-$d^2$, 43 MHz, 298 K) of $[NH_3Me][BAr^F{}_4]$.

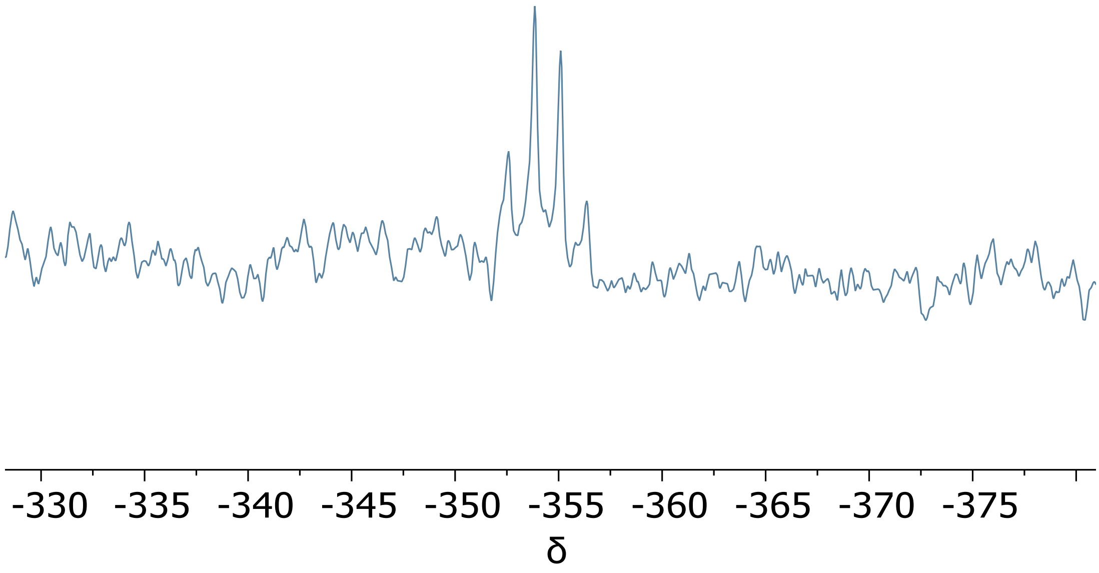


**Figure S46.** $^{14}N$ NMR spectrum (DCM-$d^2$, 43 MHz, 298 K) of $[NH_3Me][BAr^F{}_4]$.

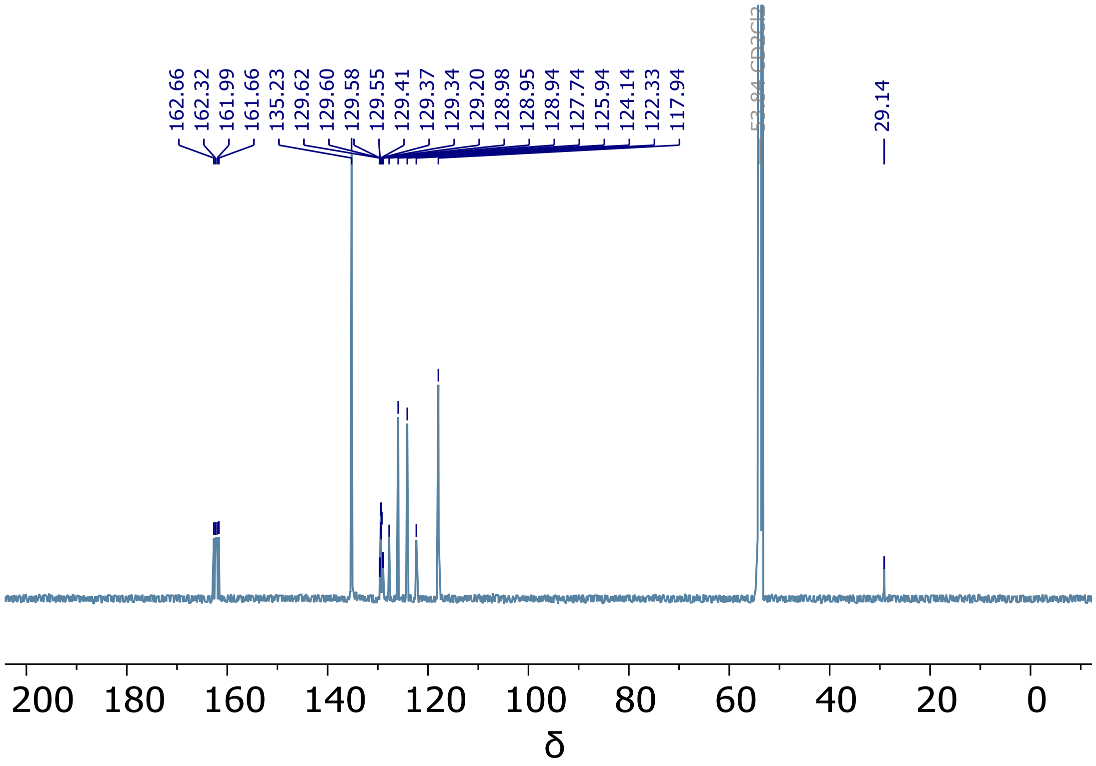


**Figure S47.** $^{13}C$ NMR spectrum (DCM-d$^2$, 151 MHz, 298 K) of $[NH_3Me][BAr^F{}_4]$.

## 3 NMR experiments

### 3.1 Effect of [Na(18-crown-6)]$^+$ on [Ir($^t$Bu-POCOP)H$_3$]$^-$ hydride region

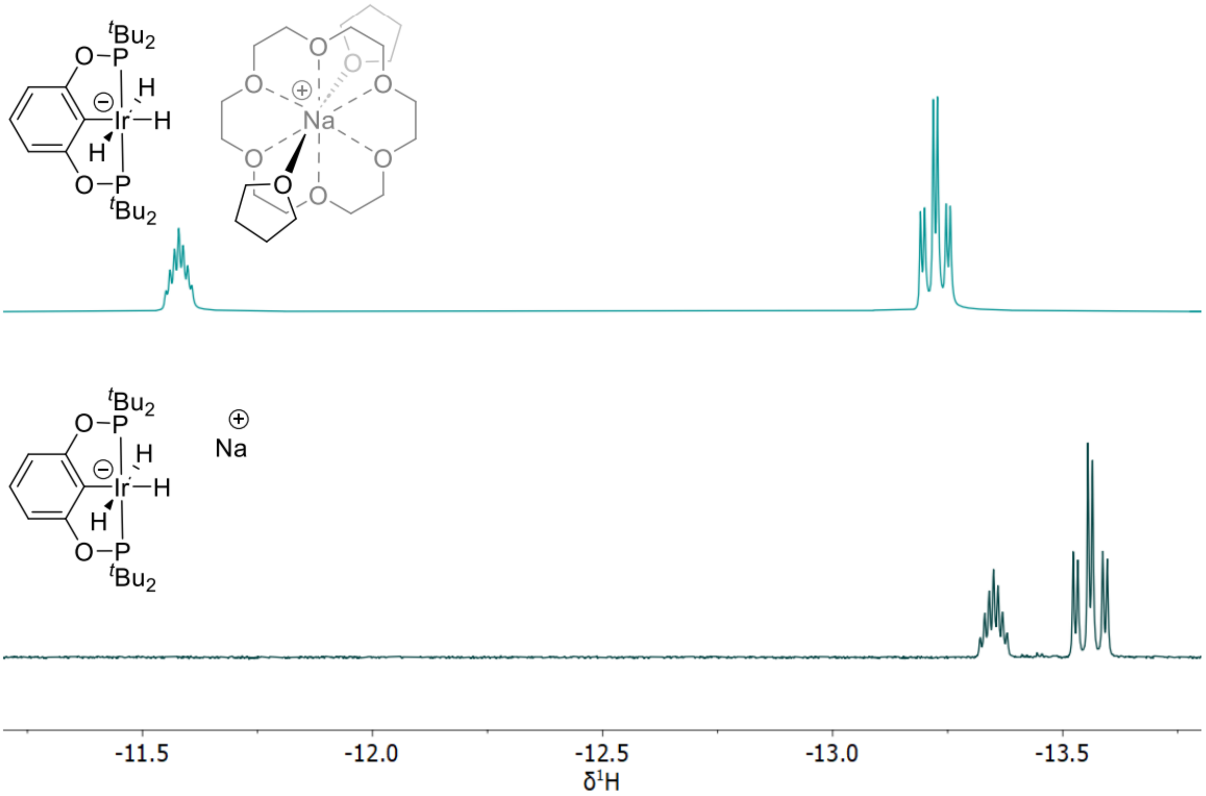


**Figure S48.** Stacked $^1$H NMR spectra (THF-d$^8$, 600 MHz, 298 K) (hydride region) showing the downfield shift of [Ir($^t$Bu-POCOP)H$_3$]$^-$ hydride signals with the [Na(18-crown-6)]$^+$ cation compared with the Na$^+$ cation.

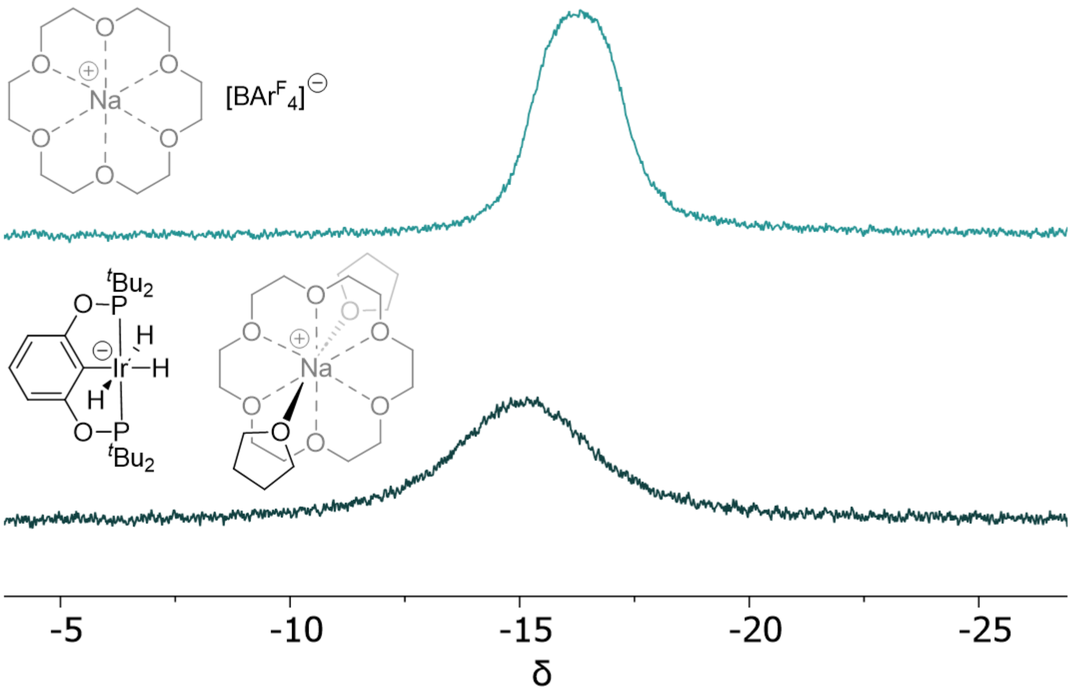


**Figure S49.** [23]Na NMR spectra (THF-d[8], 159 MHz, 298 K) of [Ir($^t$Bu-POCOP)$H_3$][Na(18-crown-6)$(THF)_2$] compared with [Na(18-crown-6)$(THF)_2$][$BAr^F_4$].

### 3.2 [Ir($^t$Bu-POCOP)H3][Na(18-crown-6)(THF)$_2$] with [$NH_3$Me][BAr$^F_4$] (1 equiv.)

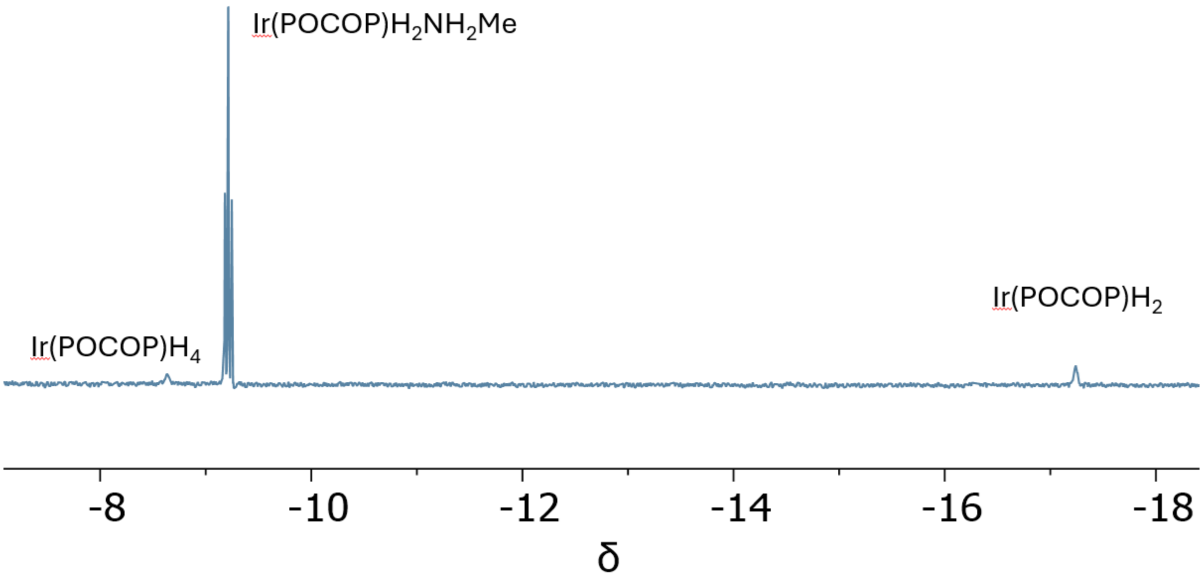


**Figure S50.** $^1$H NMR spectrum (THF-H$^8$, 500 MHz, 298 K) of an NMR scale reaction of [Ir($^t$Bu-POCOP)H$_3$][Na(18-crown-6)(THF)$_2$] and [$NH_3$Me][BAr$^F_4$] (1equiv.).

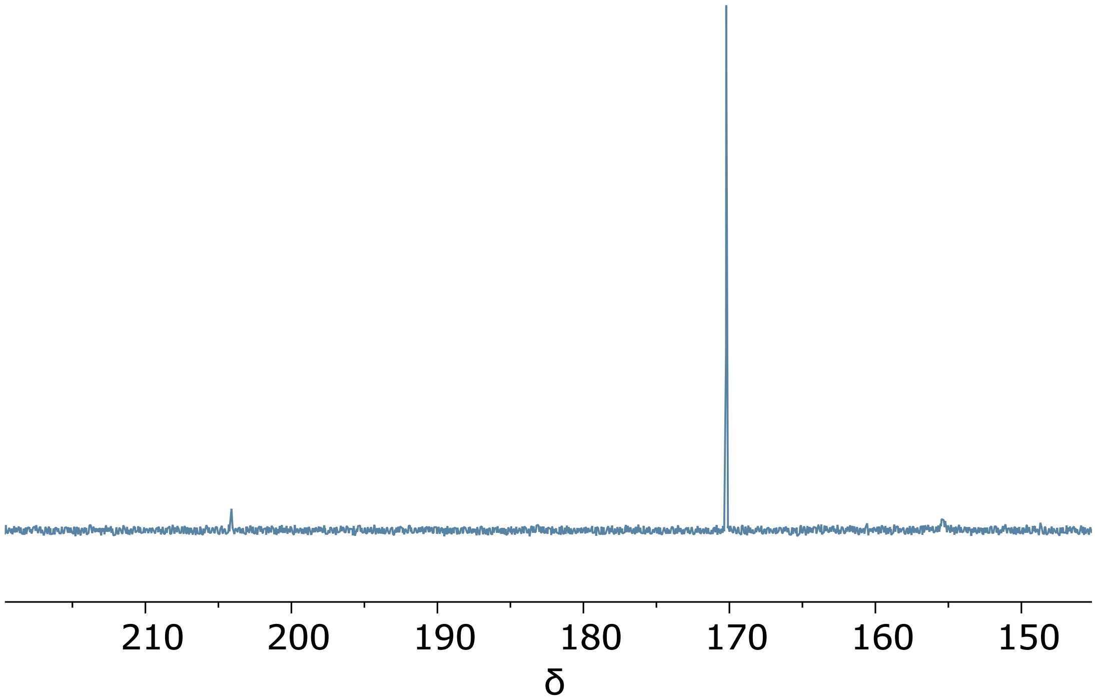


**Figure S51.** $^{31}$P {$^1$H} NMR spectrum (THF-H$^8$, 203 MHz, 298 K) of an NMR scale reaction of [Ir($^t$Bu-POCOP)H$_3$][Na(18-crown-6)(THF)$_2$] and [$NH_3$Me][BAr$^F_4$] (1equiv.). Products: 170 ppm Ir($^t$Bu-POCOP)H$_2$($NH_2$Me) and 203 ppm Ir($^t$Bu-POCOP)H$_2$.

### 3.3 Ir($^t$Bu-POCOP)(H)($BH_4$) with 20 equiv. $H_2O$ in THF

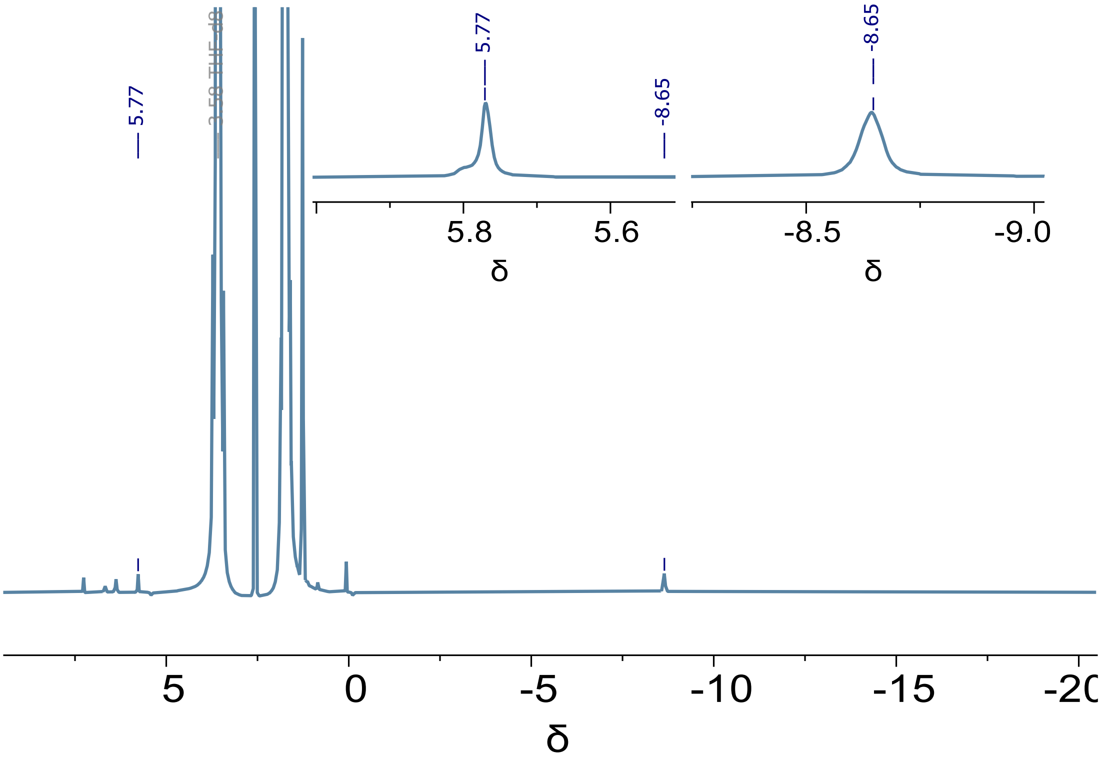


**Figure S52.** $^1$H NMR spectrum (THF-H$^8$, 500 MHz, 298 K) of Ir($^t$Bu-POCOP)(H)($BH_4$) with $H_2O$ (20 equiv.) in THF. Peak at 5.77 ppm is indicative of boric acid, peak at −8 ppm indicates Ir($^t$Bu-POCOP)$H_4$.

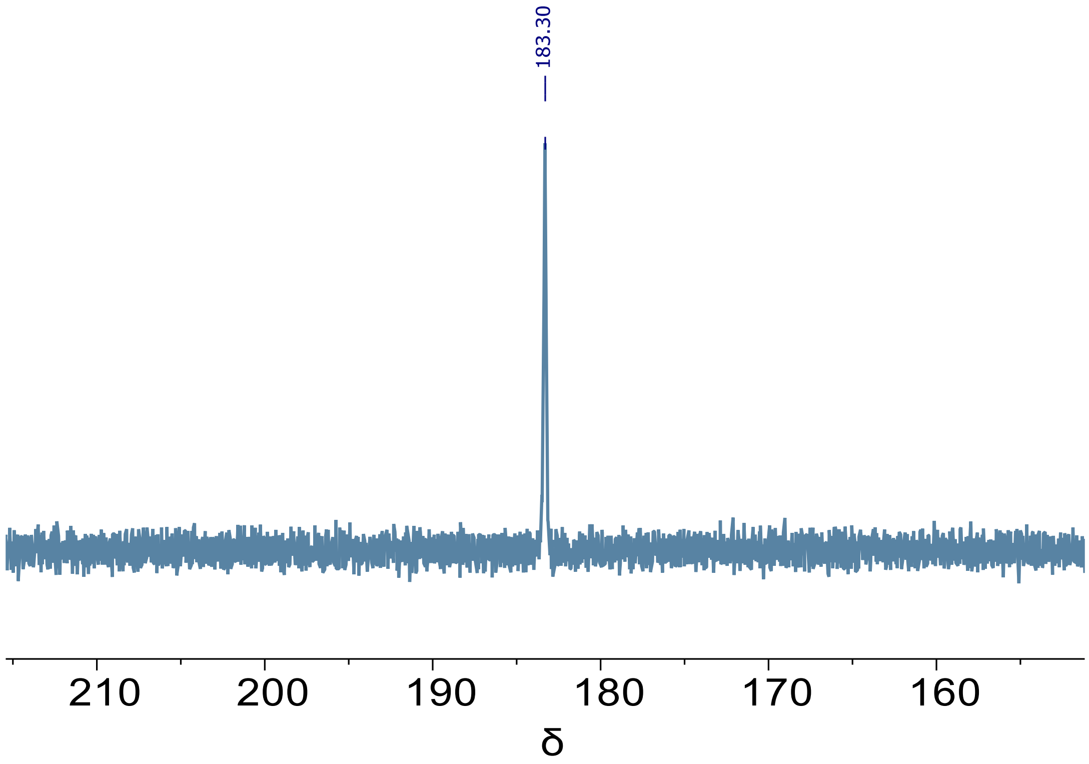


**Figure S53.** $^{31}$P {$^1$H} NMR spectrum (THF-H$^8$, 203 MHz, 298 K) of Ir($^t$Bu-POCOP)(H)($BH_4$) with $H_2O$ (20 equiv.) in THF. Peak at 183 ppm is indicative of Ir($^t$Bu-POCOP)$H_4$.

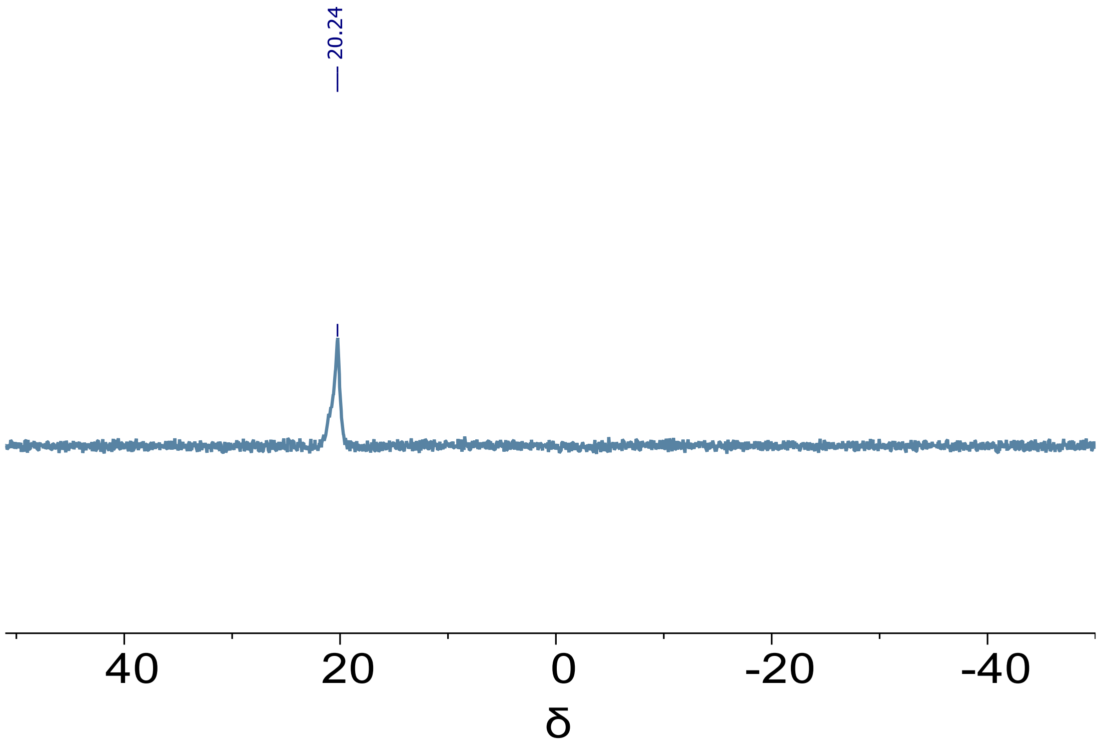


**Figure S54.** $^{11}B$ NMR spectrum (THF-H$^8$, 193 MHz, 298 K) of Ir($^t$Bu-POCOP)(H)($BH_4$) with $H_2O$ (20 equiv.) in THF. Peak at 20.2 ppm is indicative of boric acid.

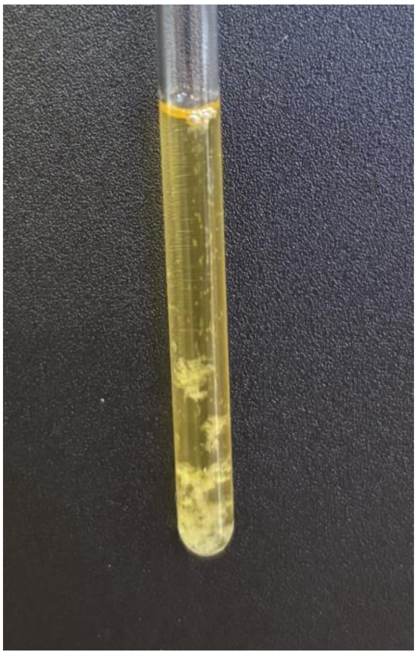

**Figure S55.** Image of NMR tube for the reaction of Ir($^t$Bu-POCOP)(H)($BH_4$) with $H_2O$ (20 equiv.) in THF showing insoluble white precipitate formed.

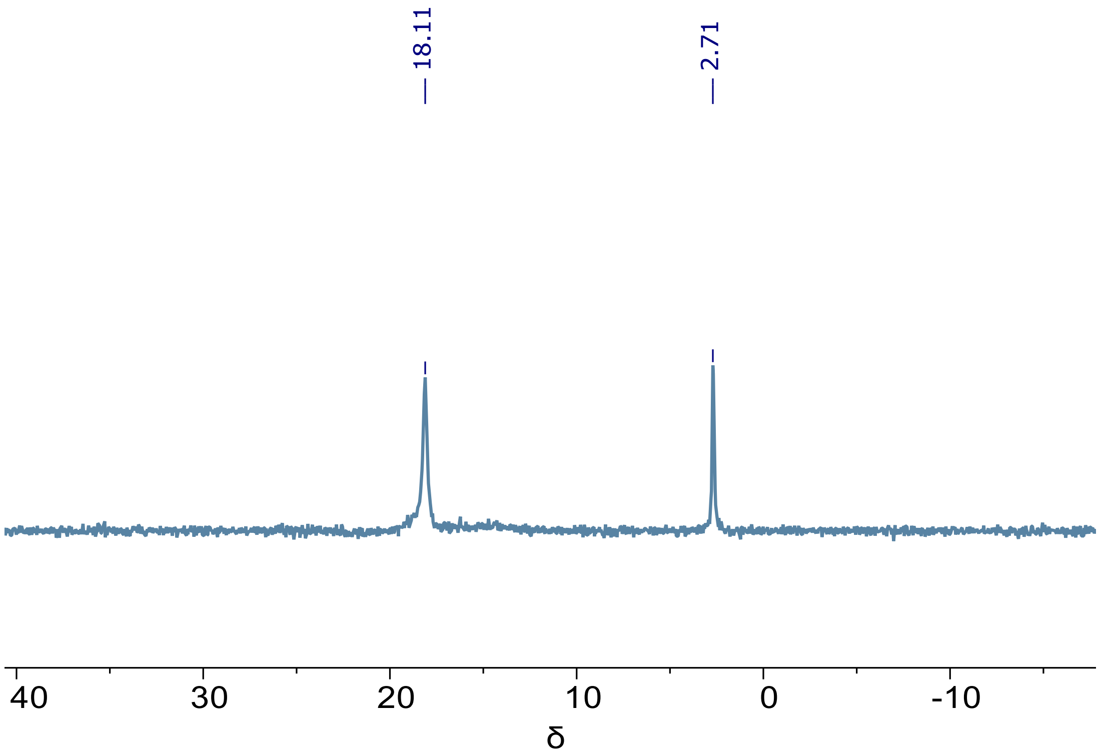


**Figure S56.** $^{11}B$ NMR spectrum (EtOH-H[6], 193 MHz, 298 K) of white precipitate formed on reaction of Ir($^t$Bu-POCOP)(H)($BH_4$) with $H_2O$ (20 equiv.). Peak at ~18 ppm is indicative of boric acid and peak at ~2 ppm is indicative of borates.

### 3.4 Addition of $[NH_3Me]Cl$ to Ir(tBu-POCOP)(H)($BH_4$) (3)

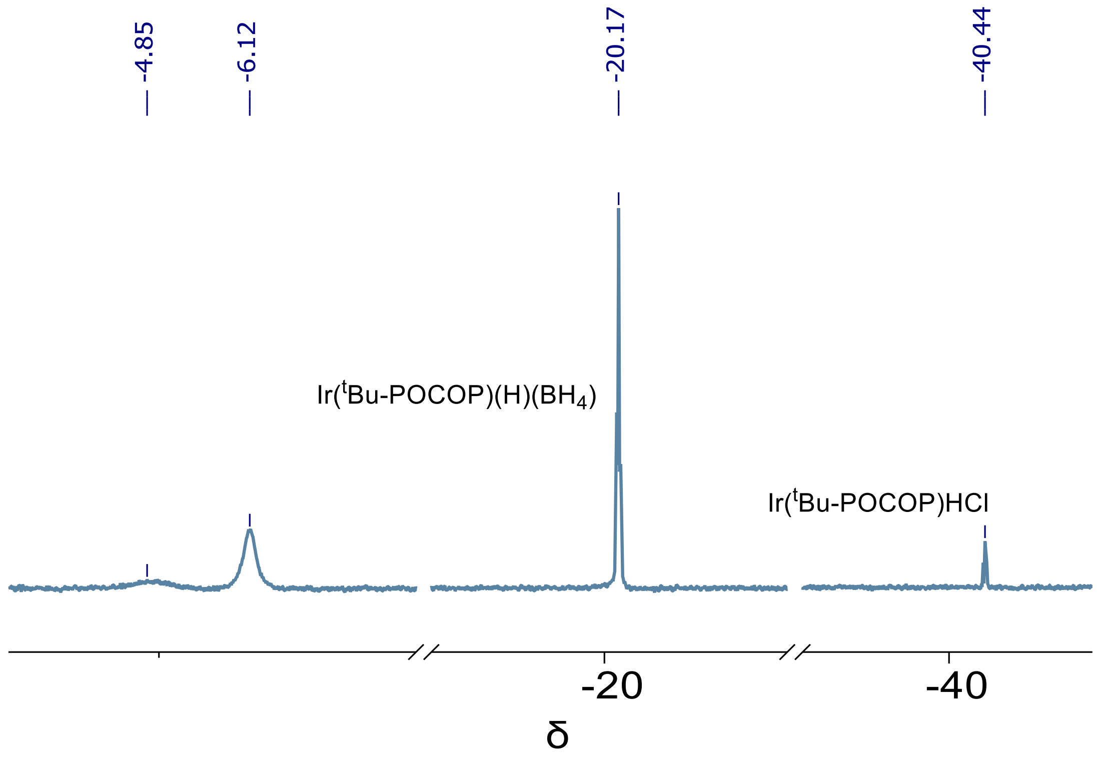


**Figure S57.** $^1$H NMR spectrum (THF-H$^8$, 600 MHz, 298 K) of Ir(tBu-POCOP)(H)($BH_4$) with $[NH_3Me]Cl$ (5 equiv.) in THF.

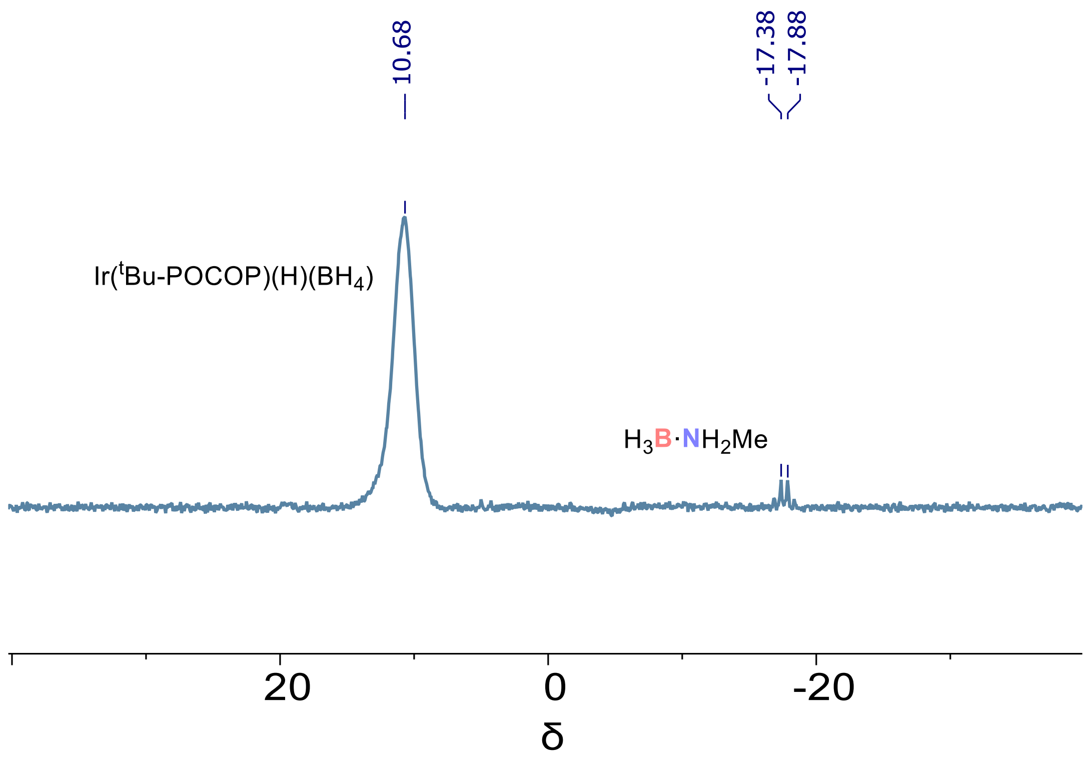


**Figure S58.** $^{11}$B NMR spectrum (THF-H$^8$, 193 MHz, 298 K) of Ir(tBu-POCOP)(H)($BH_4$) with $[NH_3Me]Cl$ (5 equiv.) in THF.

## 4 Flow NMR

### General procedure

FlowNMR experiments at the DReaM Facility were carried out with a Bruker AVIIIHD 500 MHz spectrometer equipped with a Prodigy cryoprobe using a modified InsightMR flow tube with a fused silica injection capillary as described earlier.[9] All manipulations were carried out under a dry argon atmosphere using a combination of glovebox and Schlenk techniques, and reactions were carried out with 10 mM [Cr(tmhd)$_3$] added as chemically inert paramagnetic relaxation agent.[10] All spectra were acquired under flow conditions, using InsightMR to acquire $^1$H, $^1$H with selective excitation (to just excite the hydride signals), $^{31}$P and $^{11}$B spectra, either individually or interleaved as appropriate. $^1$H experiments were typically carried out with WET solvent suppression (both THF resonances suppressed), 8 scans per experiment, a flip angle of 90°, a spectral width of 60 ppm centred at -17 ppm, and a repetition time of 4.1 s. Selective excitation $^1$H experiments were carried out using a double pulsed field gradient spin echo, typically centred at -15 ppm and with an excitation bandwidth of ~20 ppm, 8 scans per experiment and a repetition time of 3 s. $^{31}$P{$^1$H} experiments were carried out with inverse gated decoupling, 32 scans per experiment, a flip angle of 60°, a spectral width of 400 ppm centred at 200 ppm, and a repetition time of 0.9 s. $^{11}$B spectra were carried out with 16 scans per experiment, a flip angle of 90°, a spectral width of 200 ppm centred at 0 ppm, and a repetition time of 2 s. In all cases the flow rate was set to 4 mL/min, with active temperature regulation of the sample, transfer lines and NMR probe to achieve the required reaction temperature. At the start of end of flow reactions, $^1$H and inverse-gated $^{31}$P{$^1$H} spectra were also acquired under static, fully quantitative conditions (0 mL/min flow, repetition times >5T$_1$) to allow for the determination of flow correction factors for the integrals of the main peaks of interest; these were subsequently applied to the integrals obtained from flow data to ensure accurate quantitation.[11]

Ir($^t$Bu-POCOP)H$_2$ (4.44 mg, 7.5 μmol), [Cr(tmhd)$_3$] (4.5 mg, 7.5 μmol) and 1,3,5-trimethoxybenzene (2.5 mg, 15 μmol) were dissolved in dry THF$_{STILLS}$ (15 mL). This solution was stirred to ensure thorough mixing, then filtered through a syringe filter (0.2 μm) into the reaction vessel. H$_3$B·NH$_2$Me (336.5 mg, 7.5 mmol) was weighed out separately. The reaction vessel was cooled to −7 °C. The iridium solution was allowed to flow through the system and pre-catalysis spectra taken. The pump was stopped and a portion of the THF solution (~5 mL) was transferred onto the H$_3$B·NH$_2$Me, this was agitated, taken up and filtered through a syringe filter (0.2 μm) into the reaction vessel (QUICKLY). The pump was restarted, and data collection was started immediately and continued until bubble formation had stopped and catalysis was complete ($^{11}$B NMR). Catalysis conditions: 0.5 M H$_3$B·NH$_2$Me, 0.1 mol% catalyst, −7 °C.

Conditions for the study of this reaction were developed over multiple days. The first set of conditions used were 0.5 M H$_3$B·NH$_2$Me, 0.5 mol% catalyst and −7 °C, they were chosen to avoid polymer precipitation in the flow set-up, to give good signal to noise, and to slow the reaction down, respectively. These conditions resulted in the reaction progressing quickly, so that in-situ monitoring of the induction period was impossible. Over the next two days the reaction conditions were optimised to allow for the in-situ monitoring of the induction period as well as throughout the duration of catalysis. These conditions were: 0.5 M H$_3$B·NH$_2$Me, 0.1 mol% catalyst, −7 °C. Pre-catalysis spectra were taken prior to all runs, for the first three runs (days 1-2) only **1** Ir($^t$Bu-POCOP)H$_2$ was present during these baseline spectra. For runs 4-7

(days 2-4) the presence of **4** Ir($^t$Bu-POCOP)$H_2$($NH_2$Me) as well as **1** was persistent in the baseline spectra.

### 4.1 Speciation data

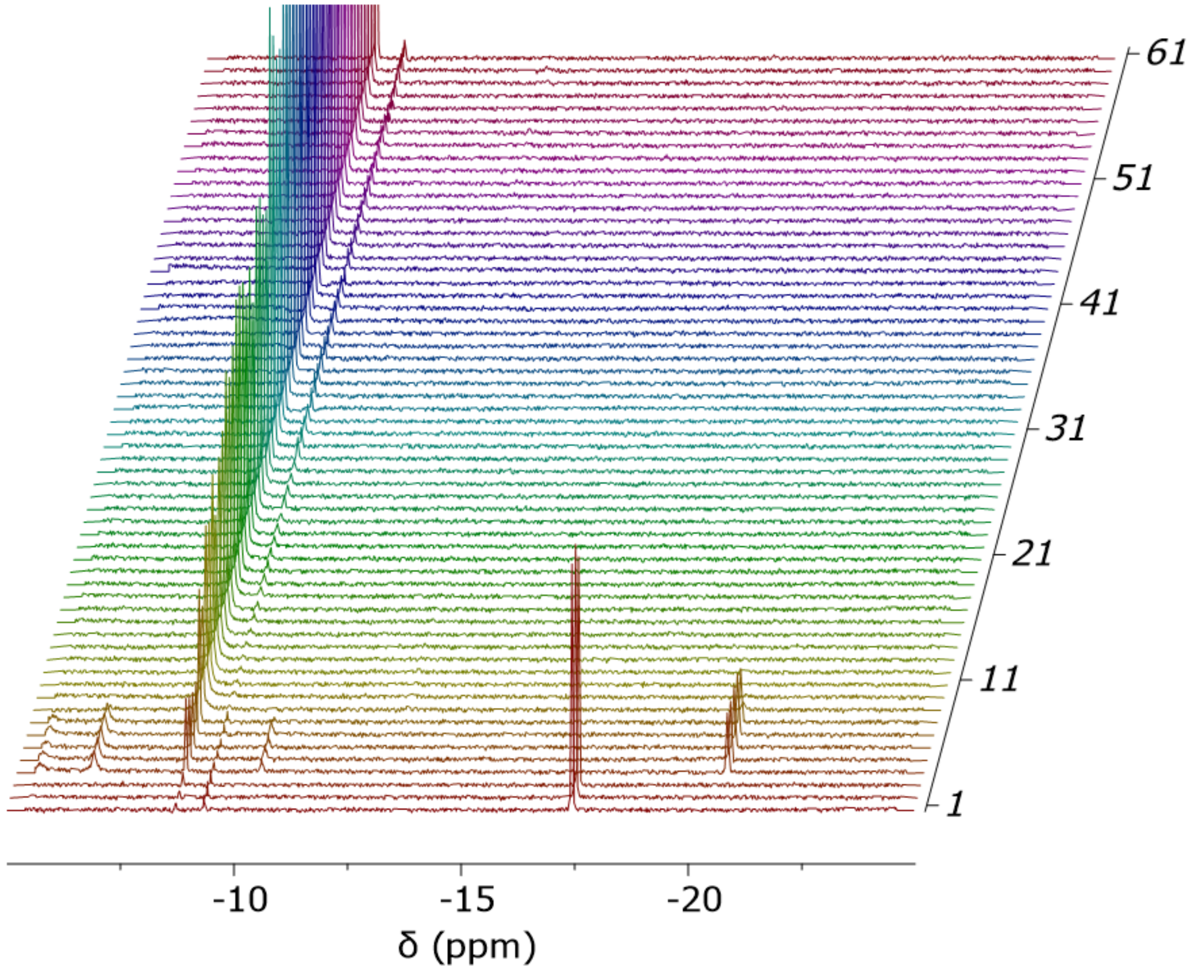


**Figure S59.** $^1$H NMR spectra (THF-$H^8$, 500 MHz, 266 K) throughout catalysis starting at spectrum 1 (hydride region only) – selective excitation experiment. Peak at −17.5 ppm corresponds to Ir($^t$Bu-POCOP)$H_2$, peaks at −5.6, −6.8 and −20.6 ppm correspond to Ir($^t$Bu-POCOP)(H)($BH_4$), peak at −8.7 ppm corresponds to Ir($^t$Bu-POCOP)$H_4$, peak at −9.4 ppm corresponds to Ir($^t$Bu-POCOP)$H_2$($NH_2$Me), peak at −10.4 ppm corresponds to an unknown Ir-hydride species.

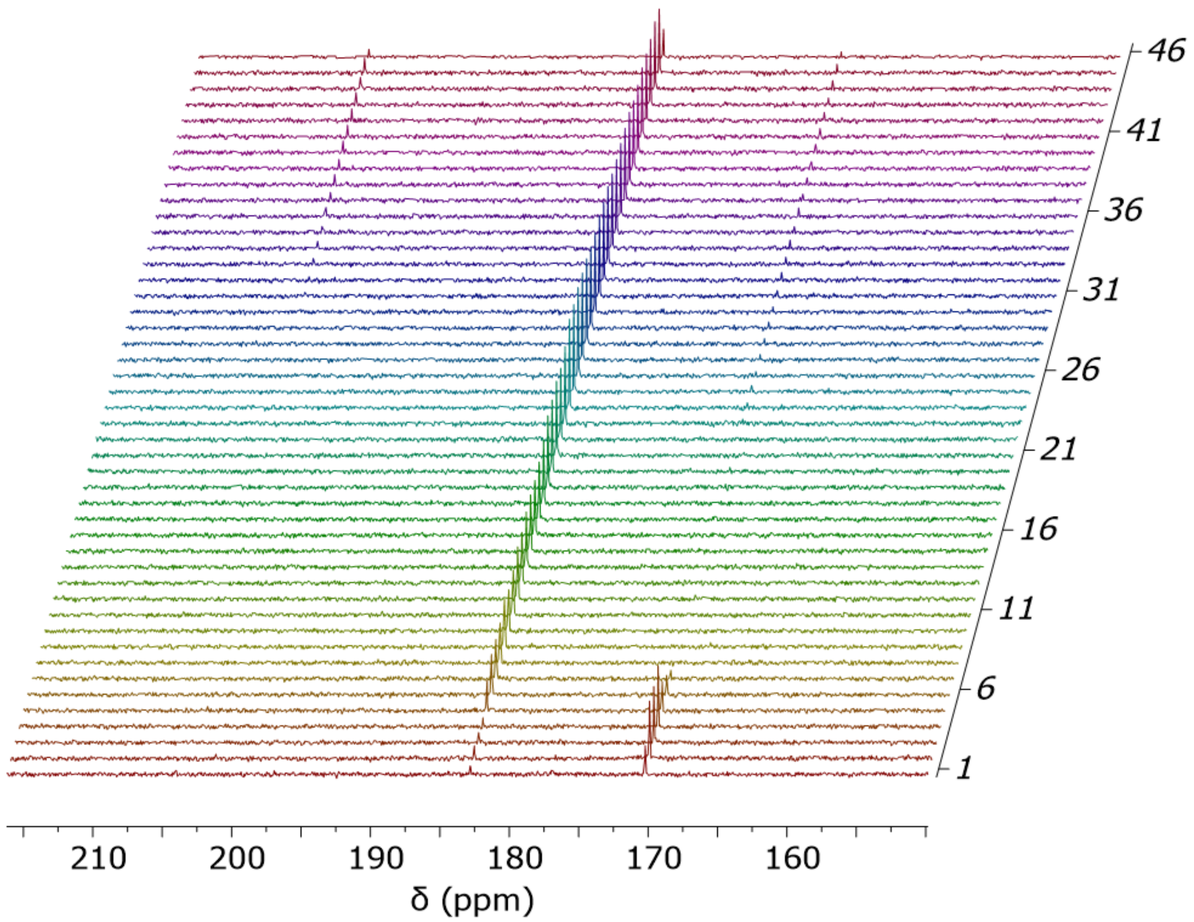


**Figure S60.** $^{31}P\{^{1}H\}$ NMR spectra (THF-$H^8$, 203 MHz, 266 K) tracking speciation throughout catalysis, once $H_3B{\cdot}NH_2Me$ had been added to **1** starting at spectrum 1. Peak at 203 ppm corresponds to Ir($^t$Bu-POCOP)$H_2$, 183 ppm corresponds to Ir($^t$Bu-POCOP)$H_4$, 170 ppm corresponds to Ir($^t$Bu-POCOP)(H)($BH_4$), 169 ppm corresponds to Ir($^t$Bu-POCOP)$H_2$($NH_2$Me) (spectrum 23 onwards)

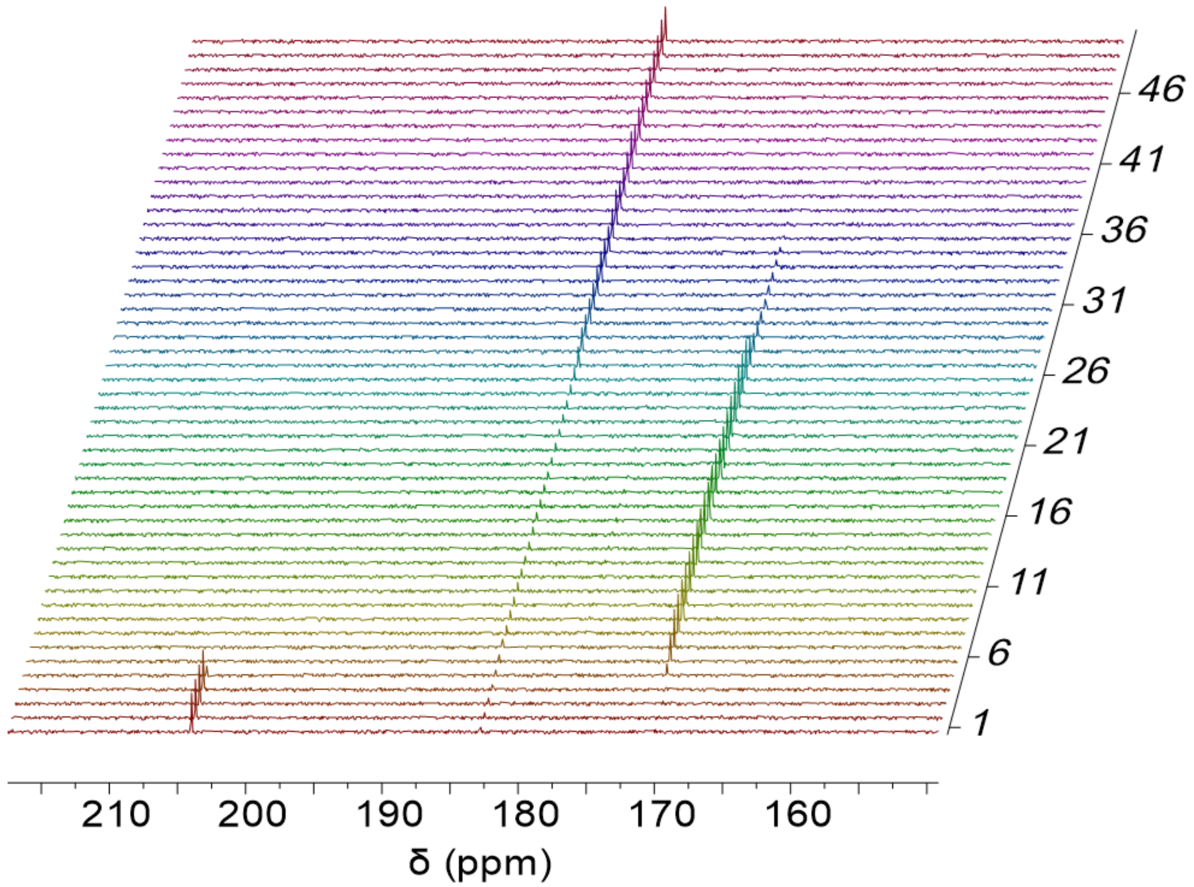


**Figure S61.** $^{31}P\{^{1}H\}$ NMR spectra (THF-$H^8$, 203 MHz, 266 K) recorded during induction period. Peak at 203 ppm corresponds to Ir($^t$Bu-POCOP)$H_2$, 183 ppm corresponds to Ir($^t$Bu-POCOP)$H_4$, 170 ppm corresponds to Ir($^t$Bu-POCOP)(H)($BH_4$).

### 4.2 Reaction progress data

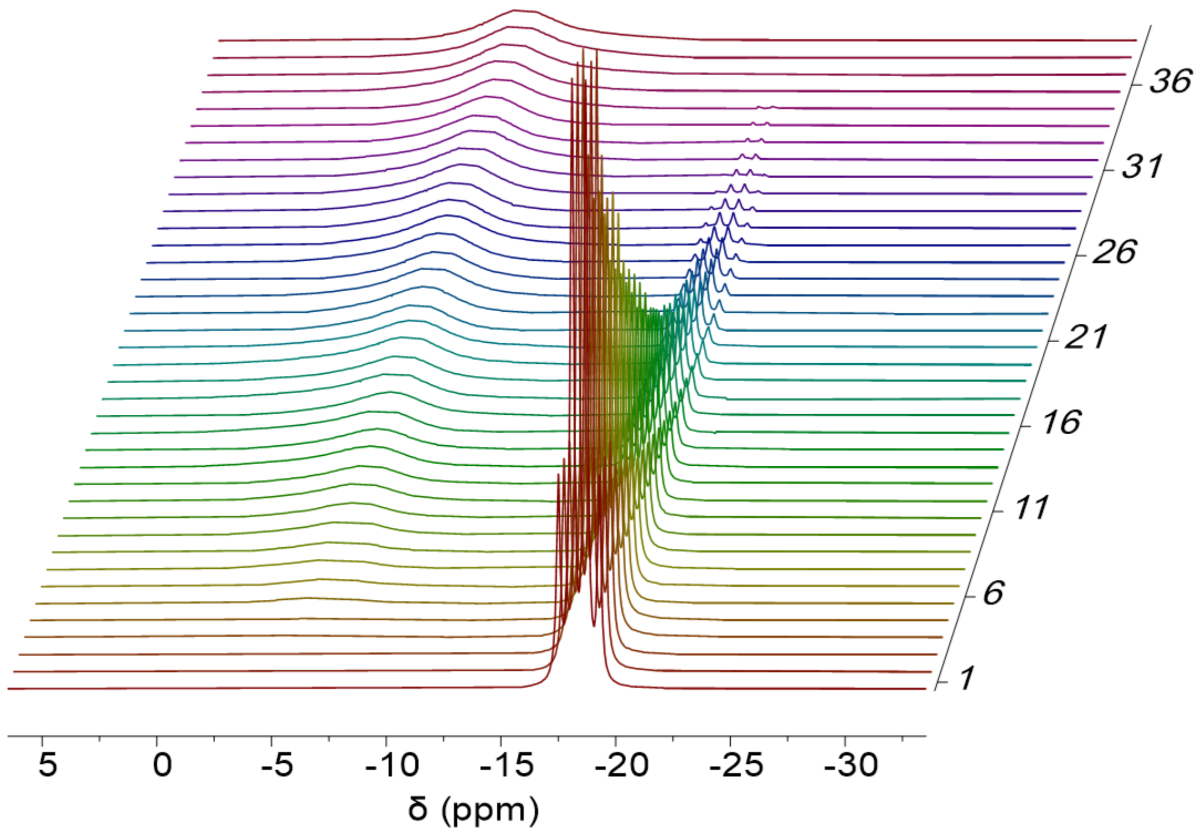


**Figure S62.** Stacked [11]B NMR spectra (THF-H[8], 160 MHz, 266 K) showing reaction progression from $H_3B{\cdot}NH_2Me$ (−18 ppm, quartet) to polyaminoborane (−6 ppm, broad singlet).

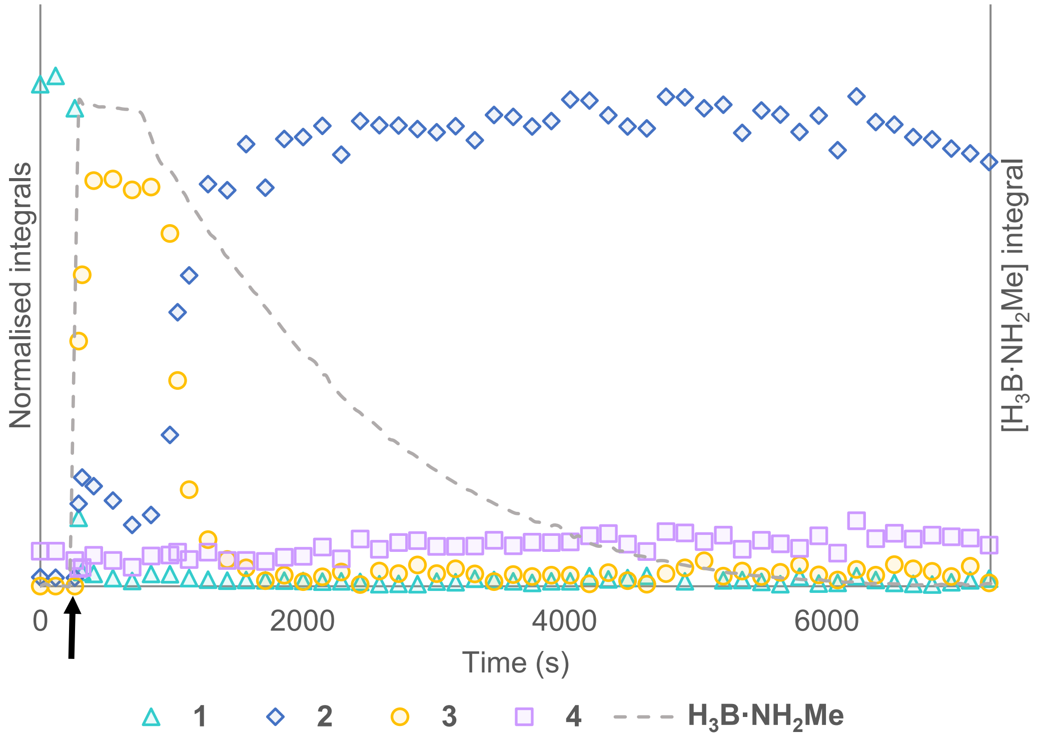


**Figure S63.** Graph showing speciation of catalyst throughout reaction from $^1$H NMR spectroscopy selective excitation experiments, with $H_3B{\cdot}NH_2Me$ conversion overlapped from $^{11}$B NMR spectra. **1** Ir($^t$Bu-POCOP)$H_2$, **2** Ir($^t$Bu-POCOP)$H_4$, **3** Ir($^t$Bu-POCOP)(H)($BH_4$), **4** Ir($^t$Bu-POCOP)$H_2$($NH_2$Me). Arrow indicates $H_3B{\cdot}NH_2Me$ addition.

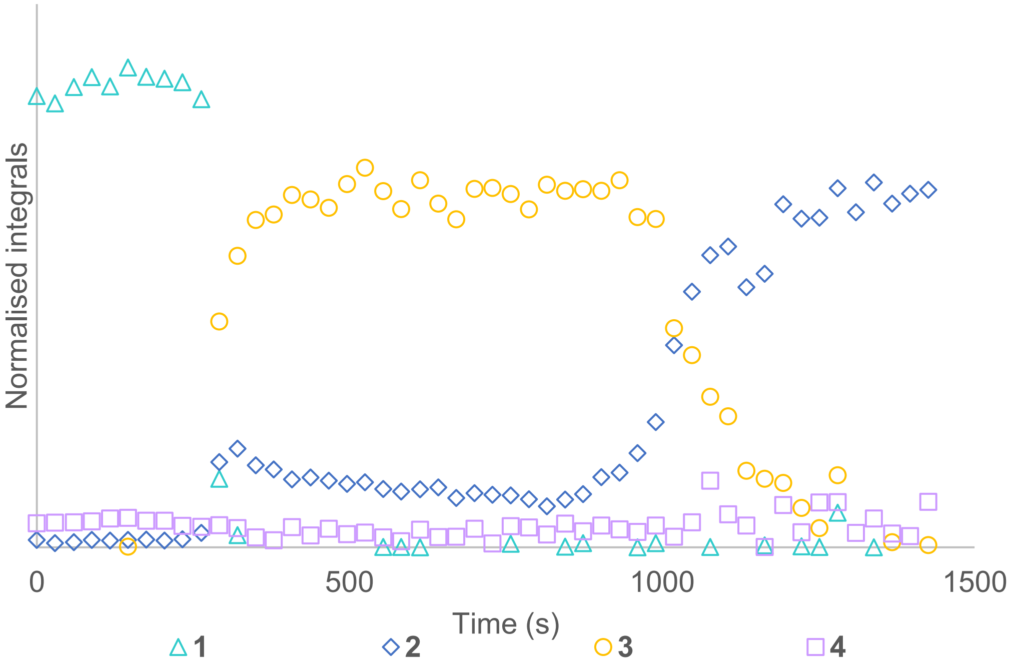


**Figure S64.** Expansion of graph showing speciation of catalyst during induction period from $^1$H NMR spectroscopy selective excitation experiments. **1** Ir($^t$Bu-POCOP)$H_2$, **2** Ir($^t$Bu-POCOP)$H_4$, **3** Ir($^t$Bu-POCOP)(H)($BH_4$), **4** Ir($^t$Bu-POCOP)$H_2$($NH_2$Me). Arrow indicates $H_3B{\cdot}NH_2Me$ addition.

## 5 Polymer Analysis

### 5.1 Scale up procedure

For scale up of reactions on a 1 g scale.:

$H_3B{\cdot}NH_2Me$ (112 mg, 2.5 mmol) and $[NH_3Me]Cl$ (0.1 mg, 1.25 µmol) was dissolved in THF (1.25 mL) in a jacketed schleck flask fitted with a circulating cooler set to 0 °C. Ir($^t$Bu-POCOP)$H_2$ (0.15 mg) was dissolved and added to the solution and stirred mechanically. Once catalysis had initiated, identified by observed effervescence, the remaining (90%) of $H_3B{\cdot}NH_2Me$ (1.008 mg, 22.5 mmol) was dissolved in THF (11.25 mL) and added dropwise by syringe pump at a flow rate of 0.1 mL/min. $NH_2Me$ (10 µL) was also added. The reaction was left to stir at 0 °C over-night with the progress checked by $^{11}B$ NMR spectroscopy. Once full conversion of pre-monomer to polymer was observed by $^{11}B$ NMR spectroscopy the polymer was precipitated in pentane (200 mL), filtered and dried *in vacuo*. White free-flowing polymer (865 mg, 81 %) was isolated and analysed by GPC to give a $M_n$ = 122,300 g mol−1, Đ = 1.5.

For scale up to 20 g:

The reactions took place in a 500 mL reaction vessel with a mechanical overhead stirrer (Figure S65), that was purged with $N_2$ for 1 hour prior to use.

$H_3B{\cdot}NH_2Me$ (as supplied, 2.1 g) and $[NH_3Me]Cl$ (1.6 mg) was added to the reaction vessel and dissolved in THF (22 ML) at room temperature (~20 °C). The remaining $H_3B{\cdot}NH_2Me$ (18.9 g) was dissolved in THF (211 mL) and connected to the rection vessel via dropping funnel. Ir($^t$Bu-POCOP)$H_2$ (2.8 mg) was dissolved in THF (1 mL) and added to the reaction vessel. Once catalysis had initiated, identified by observed effervescence, $NH_2Me$ (120 µL) was added and the remaining (90%) of $H_3B{\cdot}NH_2Me$ was added dropwise using the dropping funnel. The reaction was left over night with a slow constant flow of $N_2$ throughout. Overnight the reaction had reached 70% completion and effervescence had ceased, addition of further $NH_2Me$ (120 µL) resulted in further gas evolution, the addition of $NH_2Me$ was repeated 2 further times at ~ 5 hour time intervals. After 48 hours total reaction time the polymer was precipitated in pentane (1 L), filtered and dried *in vacuo*. White free-flowing polymer (16 g, 80%) was isolated and analysed by GPC to give a $M_n$ = 35,300 g mol−1, Đ = 1.9.

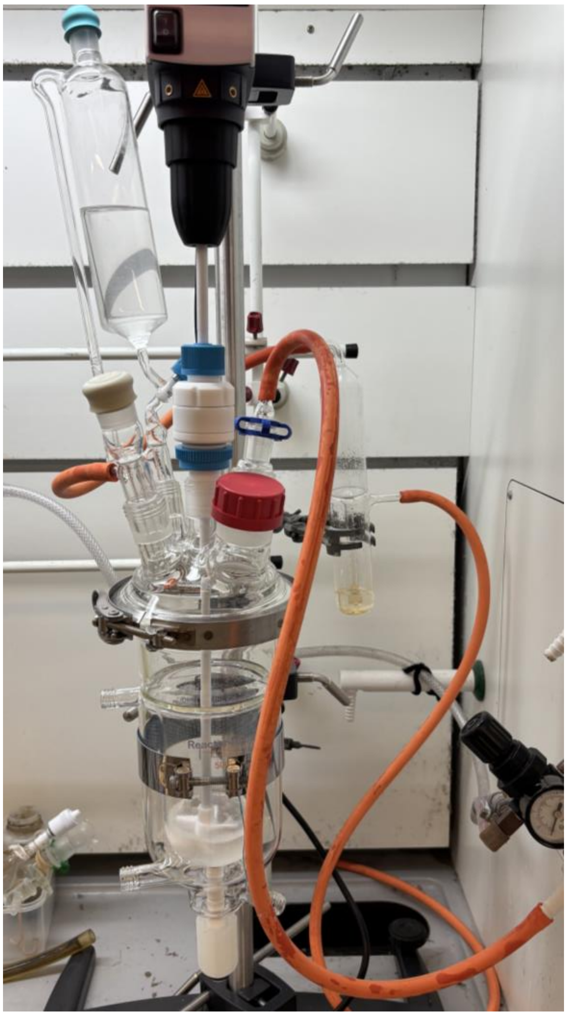

**Figure S65.** 20 g reaction set-up in 500 mL reaction vessel with a mechanical overhead stirrer.

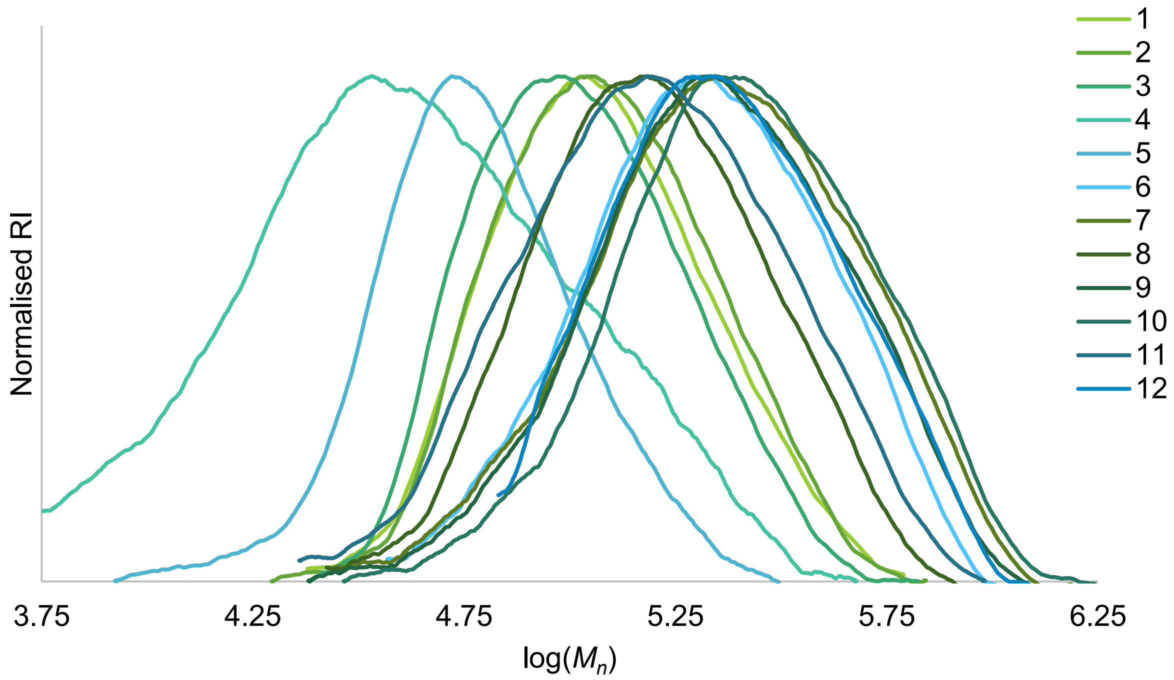


**Figure S66.** GPC traces of polymers from Table S17 showing range of molecular weights from $M_n$ = 35,000 – 191,000 g mol$^{-1}$.

**Table S13.** Control of $M_n$ via a range of different reaction conditions exemplifying range. Conditions unless stated otherwise: 2 M $H_3B·NH_2Me$, 2 mM (0.1 mol%), 293 K, THF. [a]Determined by the pseudo zero order region of the profile [b]Relative to polystyrene standards.

| Entry | Catalyst loading (mol%) | Temp. (°C) | $H_3B·NH_2Me$ | Conditions | Stirring (rpm) | Scale (g) | Rate[a] (mM $s^{-1}$) | $M_n$[b] (g $mol^{-1}$) | Đ |
|---|---|---|---|---|---|---|---|---|---|
| 1 | **0.1** | 20 | As supplied | | 400 | 0.1 | 9.30 | 100,000 | 1.4 |
| 2 | **0.1** | 20 | Recryst. | Standard Conditions | 400 | 0.1 | 5.95 | 103,100 | 1.3 |
| 3 | **0.05** | 20 | Recryst. | - | 400 | 0.1 | 3.36 | 91,700 | 1.3 |
| 4 | **0.001** | 20 | As supplied | **5 equiv. $[NH_3Me]Cl$, 5 equiv. $NH_2Me$,** Mechanical overhead stirrer | 100 | 20 | - | 35,000 | 1.9 |
| 5 | 0.1 | 20 | Recryst. | **1 mol% boronium** | 400 | 0.1 | 4.0 | 54,700 | 1.3 |
| 6 | 0.1 | 20 | Recryst. | **2.5 equiv. $NH_2Me$** | 400 | 0.1 | 10.2 | 156,000 | 1.5 |
| 7 | 0.1 | 20 | Recryst. | **30 equiv. $NH_2Me$** | 400 | 0.1 | 13.6 | 170,000 | 1.6 |
| 8 | 0.1 | **10** | Recryst. | - | 400 | 0.1 | 3.60 | 124,200 | 1.4 |
| 9 | 0.1 | **0** | Recryst. | - | 400 | 0.1 | 1.68 | 166,100 | 1.5 |
| 10 | 0.1 | **−10** | Recryst. | - | 400 | 0.1 | 0.68 | 191,200 | 1.5 |
| 11 | 0.001 | −10 | Recryst. | **5 equiv. $[NH_3Me]Cl$, 5 equiv. $NH_2Me$** | 400 | 1 | - | 122,200 | 1.5 |
| 12 | 0.1 | 20 | Recryst. | **$[Ir(^tBu\text{-}POCOP)H_3]^-$, 1 equiv. $[NH_3Me]^+$** | 400 | 0.1 | 19.2 | 185,200 | 1.4 |

### 5.2 Molecular weight vs. conversion

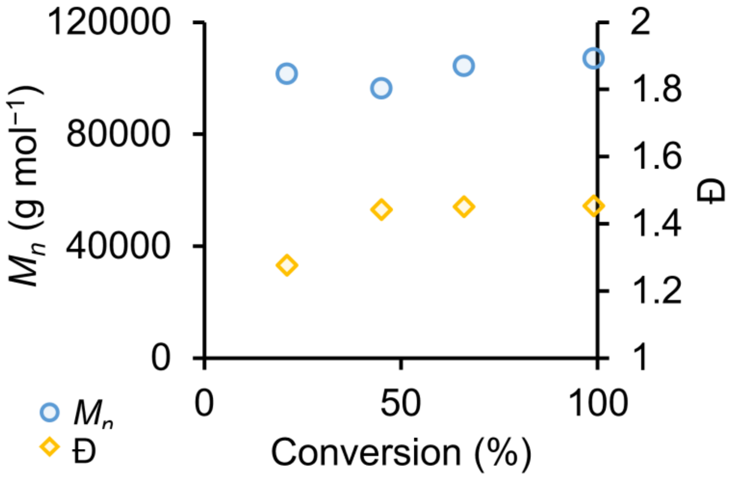


**Figure S67.** Graph showing molecular weight of polymer against conversion (%). The reaction was halted using excess $PMe_3$ (1 M in THF). Conditions: 0.1 mol% [Ir], 2 M $H_3B{\cdot}NH_2Me$, 293 K, THF.

### 5.3 NMR spectra

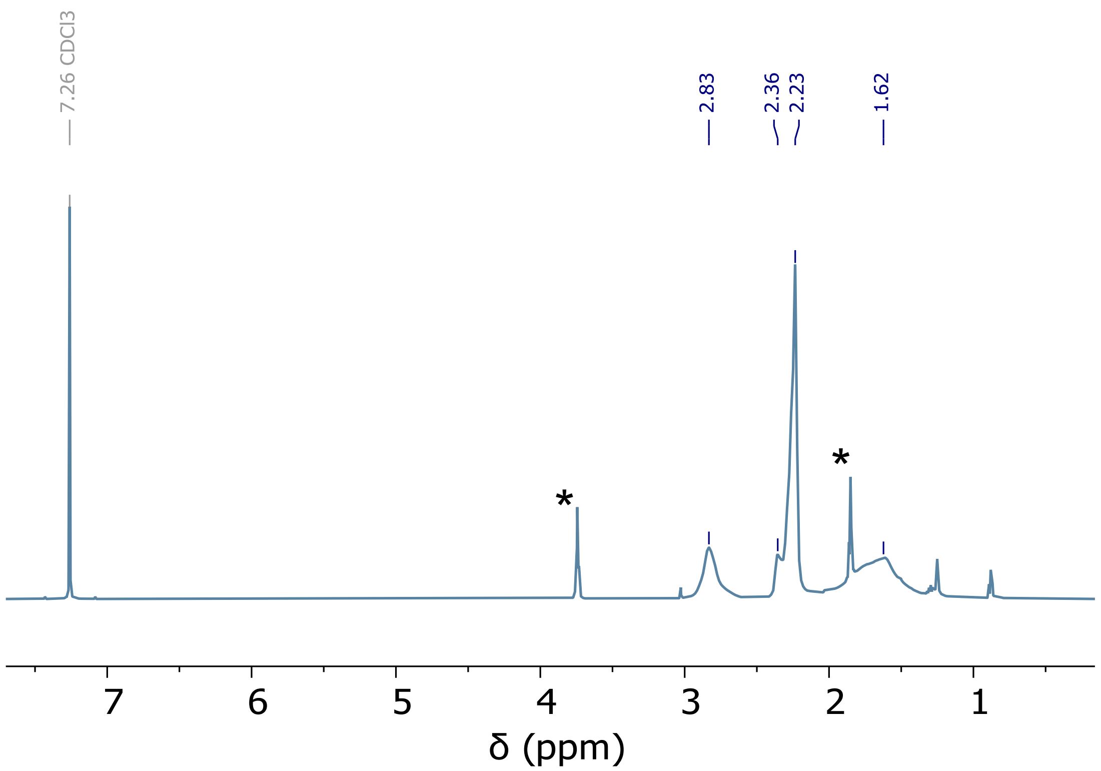


**Figure S68.** Typical $^{1}H$ NMR spectrum ($CDCl_3$, 600 MHz, 298 K) of polyaminoborane, *indicates residual solvent.

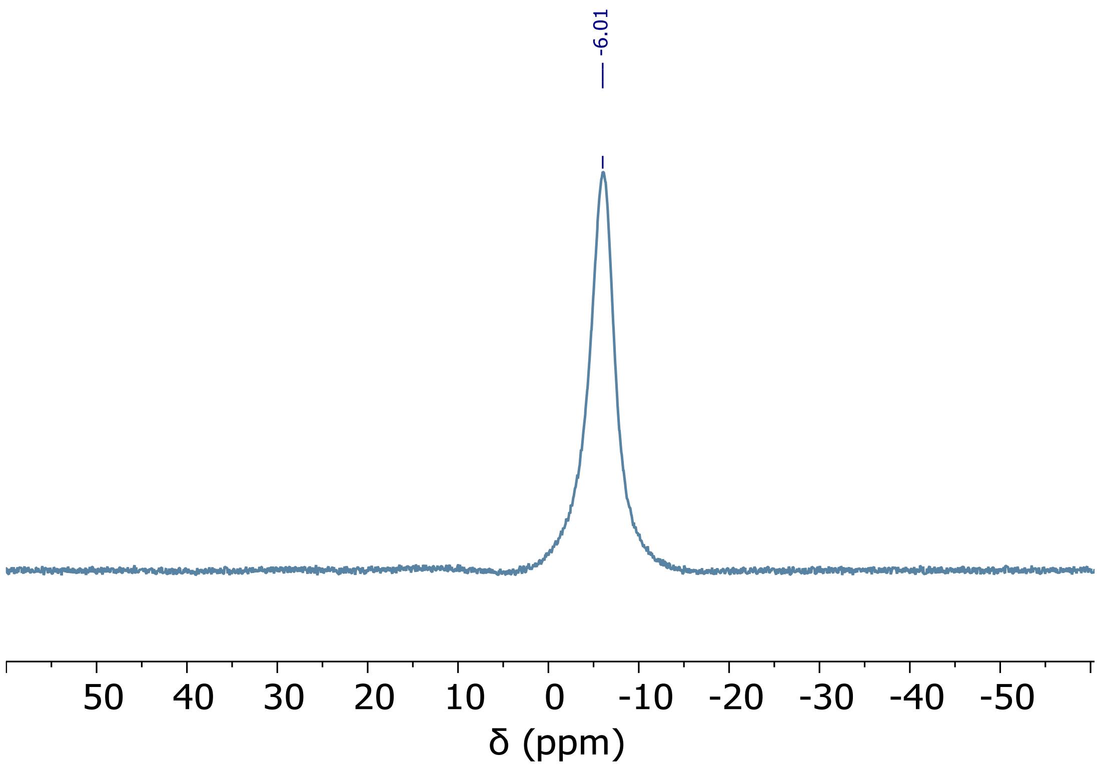


**Figure S69.** Typical $^{11}B$ NMR spectrum ($CDCl_3$, 193 MHz, 298 K) of polyaminoborane.

**5.4 GPC**

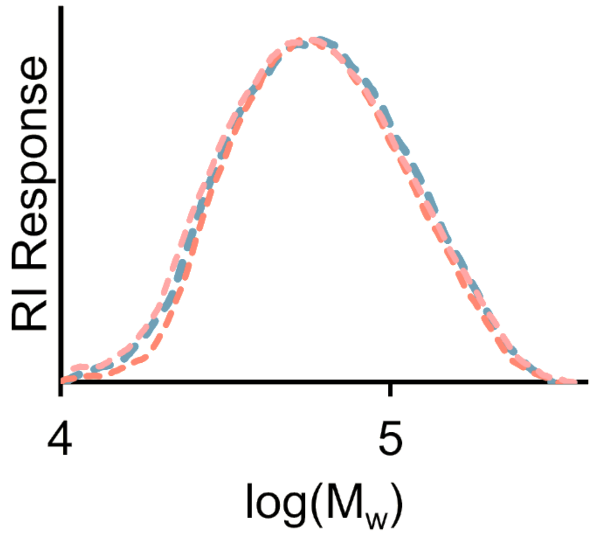


**Figure S70.** Overlayed GPC traces showing reliability of polymer formation. Conditions: 0.1 mol% [Ir], 2 M $H_3B·NH_2Me$, 293 K, THF.

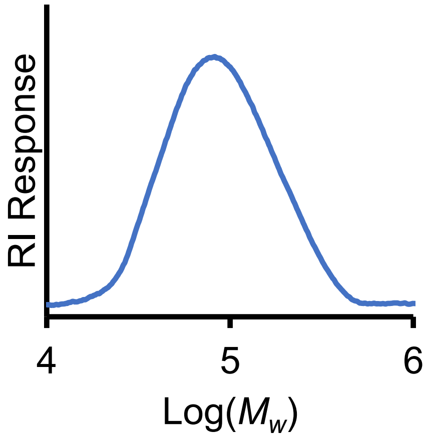


**Figure S71.** GPC trace for dehydropolymerisation of $H_3B·NH_2Me$ with catalyst **1** in air. Conditions: 0.05 mol% [Ir], 5 equiv. $NH_2Me$.

## 5.5 TGA and DSC

1

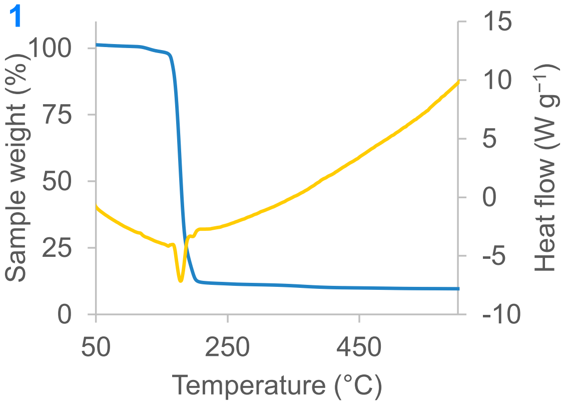


2

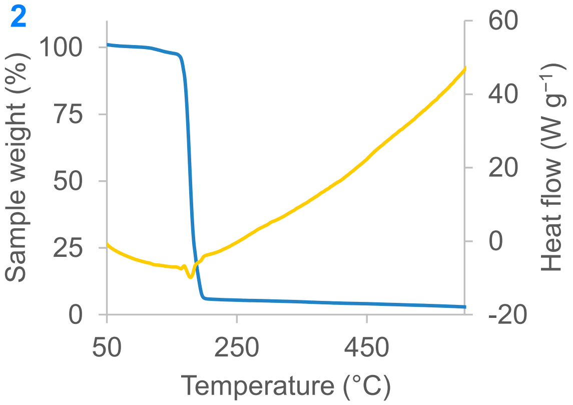


4

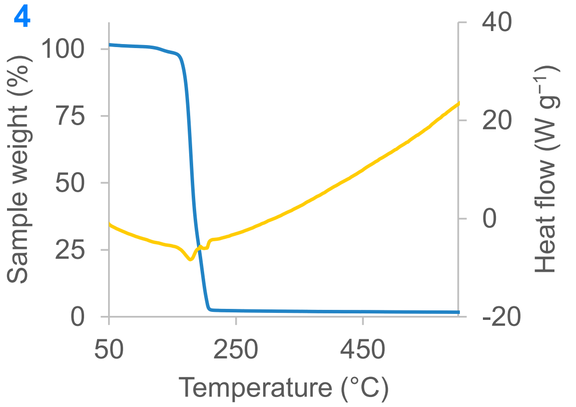


5

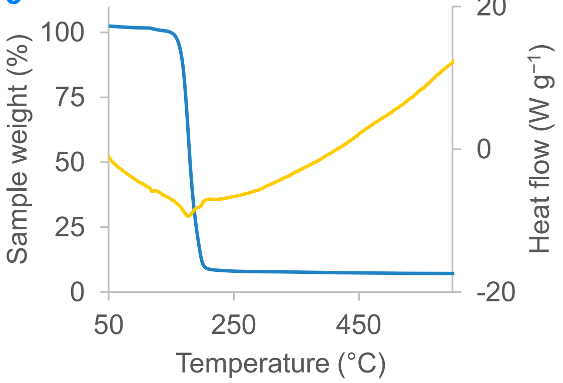


6

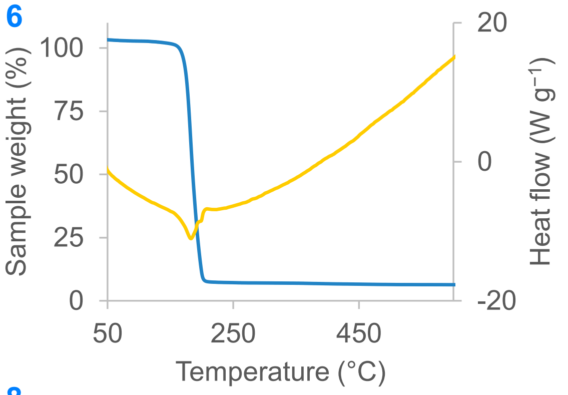


7

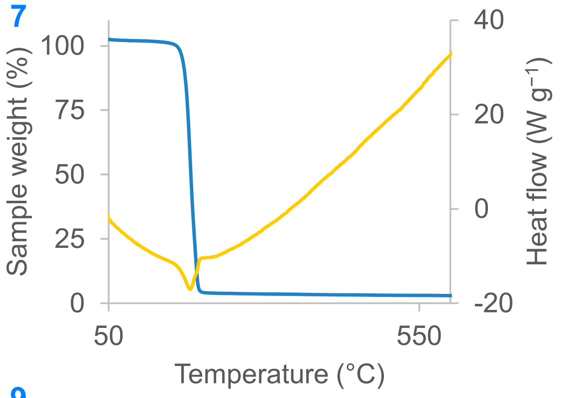


8

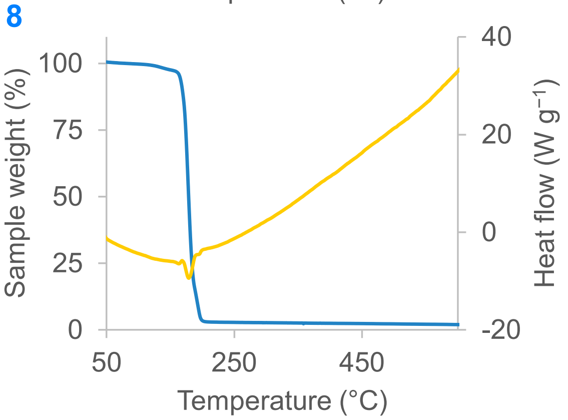


9

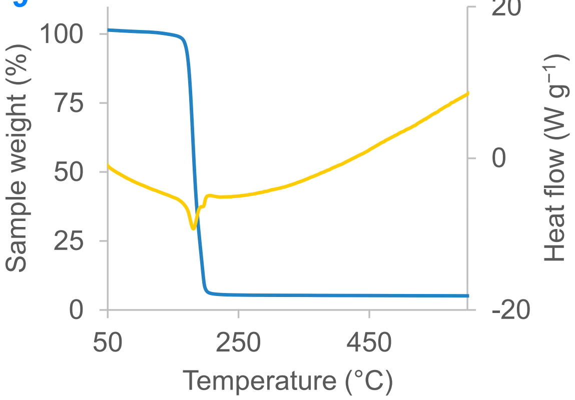

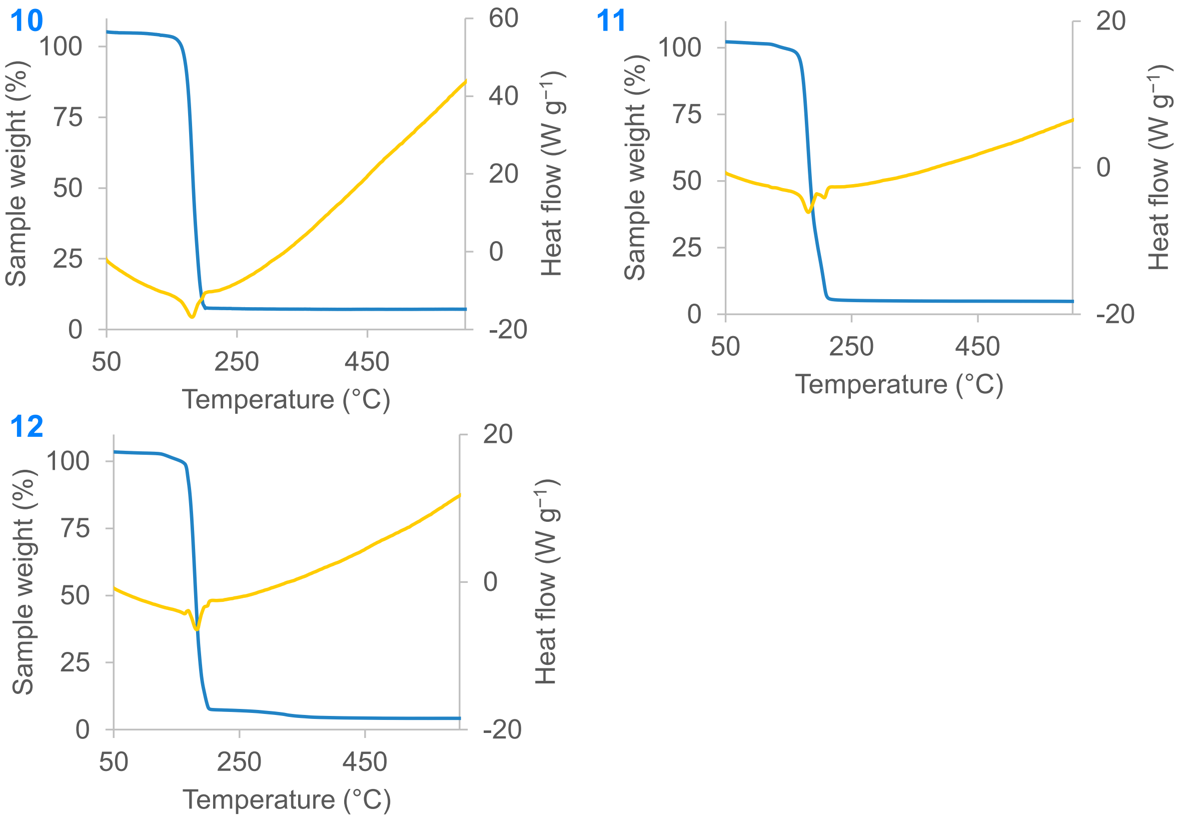


**Figure S72.** TGA data for entries in Table S13 for a range of $M_n$.

DSC data was collected for these samples as well. The glass transitions could not be reliably observed as the signal was weak and inconclusive. We have chosen to not draw conclusions from this data.

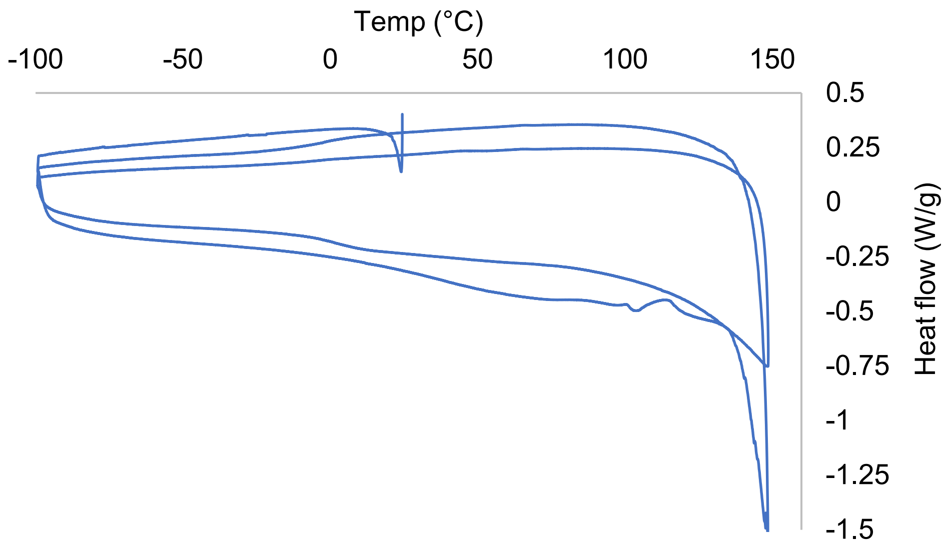


**Figure S73.** DSC data for Entry 5 (Table S13), $M_n$ = 54,700, $T_g$ = −1.5 °C.

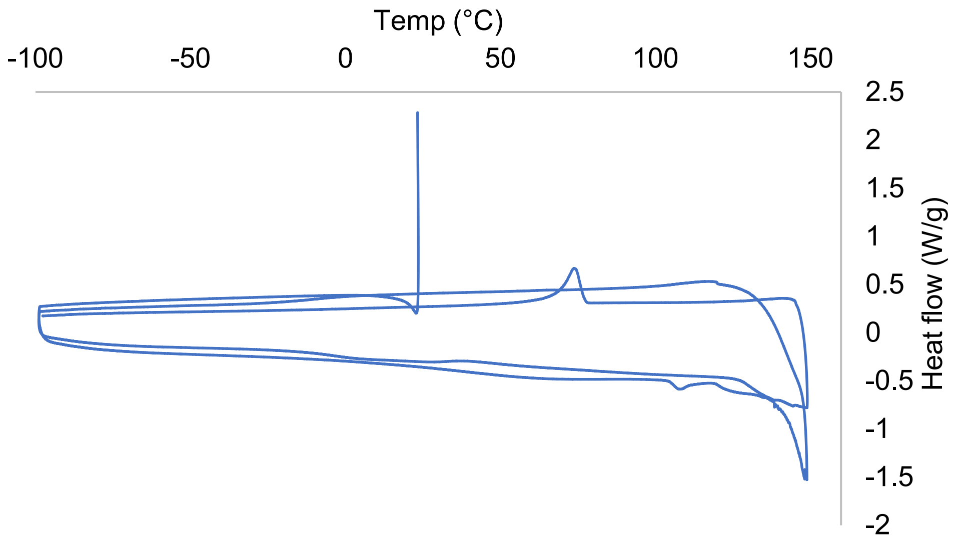


**Figure S74.** DSC data for Entry 2 (Table S13), $M_n$ = 103,200, $T_g$ = −5.0 °C.

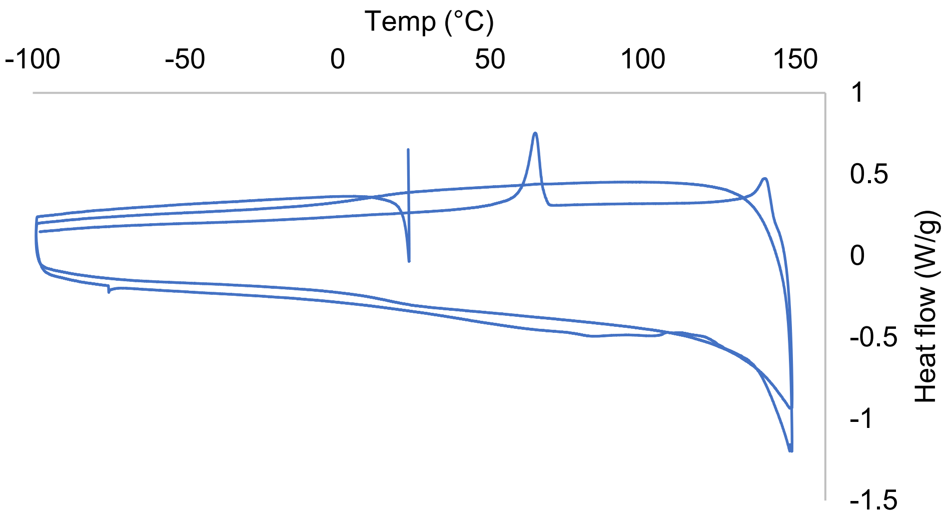


**Figure S75.** DSC data for Entry 6 (Table S13), $M_n$ = 156,000, $T_g$ = 13.2 °C.

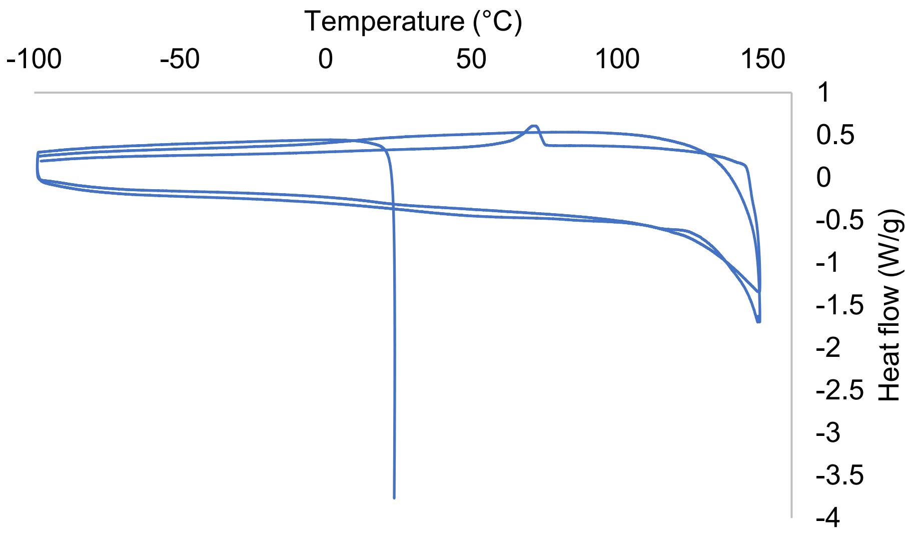


**Figure S76.** DSC data for Entry 10 (Table S16), $M_n$ = 191,200, $T_g$ = 9.6 °C.

### 5.6 ICP

Pre-treatment of polymer samples required prior to acid digestion. Polymer reacts **VIOLENTLY** with concentrated nitric acid or other strong oxidisers.

**Pretreatment**

Samples of polyaminoborane were weighed into 15 mL virgin polypropylene tubes. Pretreatment blank was also prepared in a separate tube. Type I ultrapure water (5.00 mL, Milli-Q benchtop system supplied by Merck) was added to each tube. For samples where acid digestion was required to ensure full suspension, $HNO_3$ (20%, prepared from 70% AR grade supplied by Fisher) was added to digestion tube with care over 1 min. Samples tubes were loosely capped and left for 5 days with occasional gentle agitation. Pretreatment was considered complete when no further solid was observable in the tubes. Solvent was removed under gentle flow of filtered $N_2$, approx. 24 h, to leave a white to off-white residue.

**Digestion**

Samples and pretreatment blank were transferred with washing (1.00 mL type I ultrapure water) to teflon semi-venting digestion tubes. Digestion recovery standard (1.00 mL 10 ppm CCS-2) and digestion blank (1.00 mL ultrapure water) were also added to separate tubes. $HNO_3$ (3.50 mL, 70% AR grade, Fisher chemical) and HCl (1.35 mL, 37% AR grade, Fisher chemical) were added. No effervescence was observable when adding digestion acids. Samples were digested by microwave digestion in Anton-Paar Multiwave Go Plus, ramp rate 17 °C $min^{-1}$, ultimate temperature 170 °C, dwell time 15 minutes. Samples were allowed to cool and transferred to 50 mL volumetric flask, made to mark and transferred to sample tubes (virgin polypropylene, 50 mL) for analysis. Visual inspection showed no remaining solid residue.

**Analysis**

Samples were analysed with Agilent ICP-OES 5800 VDV spectrometer. Plasma flow 12.0 L/min, RF power 1.20 kW, auxiliary flow 1.00 L/min, axial viewing mode. Working standards (0.01 - 10 ppm Ir) were prepared from commercial reference standard CCS-2 (100 ppm Ir) supplied by Inorganic Ventures, traceable to NIST certified reference materials. Analysis was completed at 224.268 nm with internal standard Y (371.029 nm). All standards were matrix matched to the digestion media.

**Table S14.** ICP-OES Analysis of polymer samples with different catalyst loadings to show residual [Ir].

| Catalyst Loading (mol%) | [Ir] (weight ppm) | Ir ppm/mol |
|---|---|---|
| 1.0 | 11063 | 2464 |
| 0.1 | 1002 | 223 |
| 0.01 | 89 | 20 |
| 0.001 | 21 | 5 |

## 5.7 Mass Spectrometry

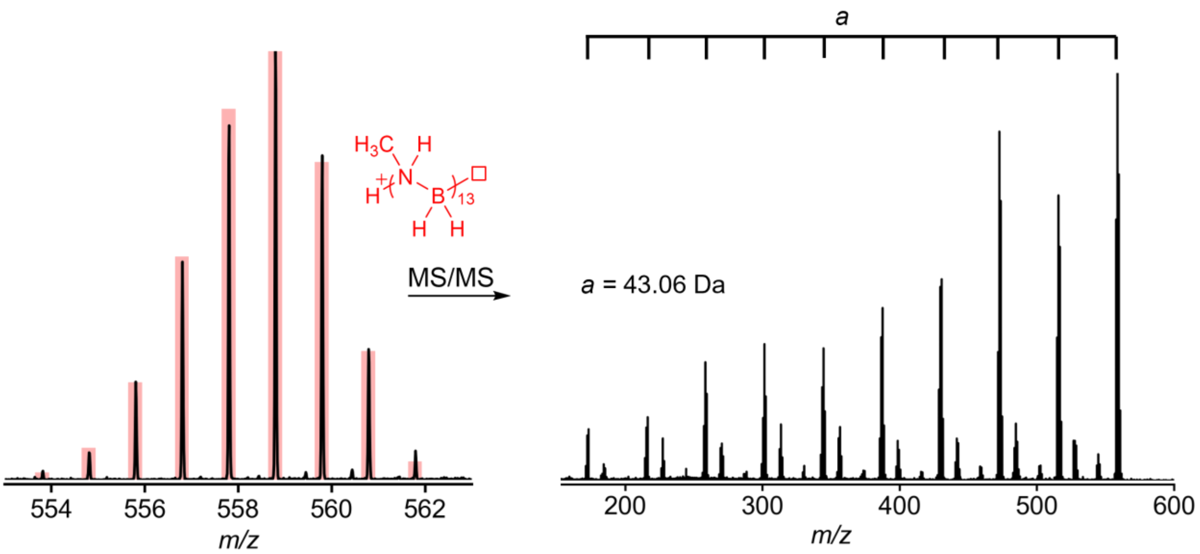


**Figure S77.** ESI-MS/MS of $[H(NMeHBH_2)_n]^+$.

## 6 COPASI

A

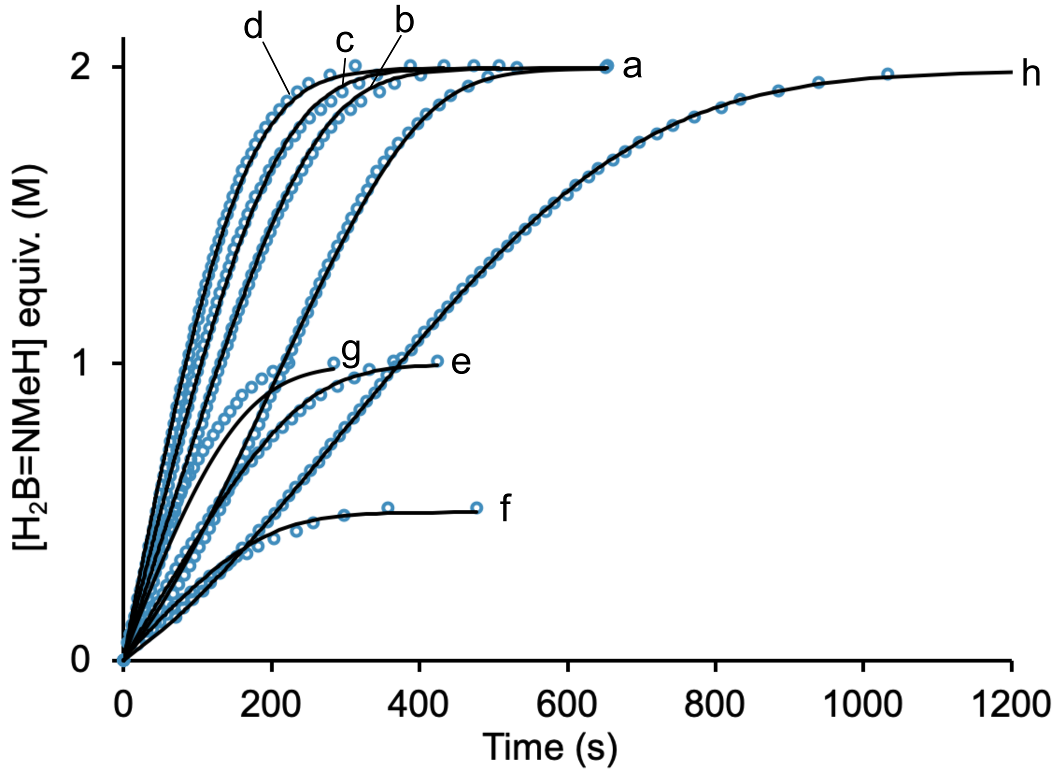


B

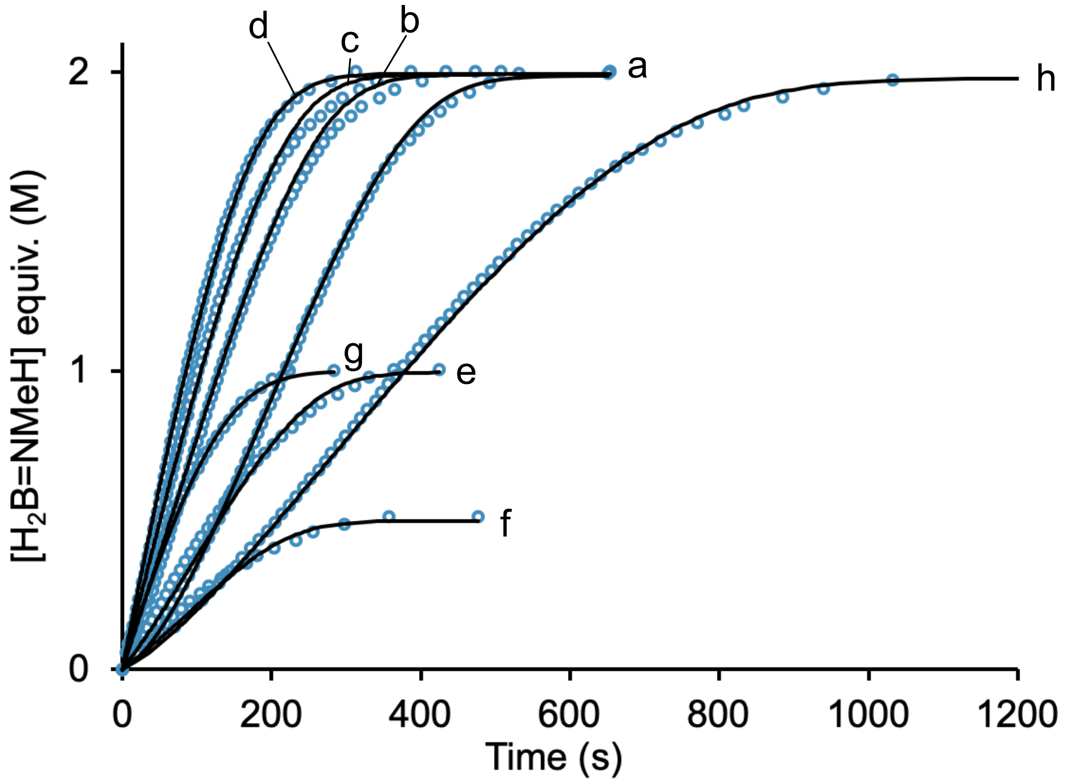


**Figure S78.** a) Concentration of [$H_2B$=NMeH] as a proxy for $H_2$ evolution versus time for eight different starting concentrations of listed in Table S15. [$H_2$] limited to 0.04 M using the "Events" function in COPSAI. Open circles = experimental data; solid lines = holistically simulated data derived for the two catalytic manifolds A and B. Data is time-shifted to remove induction periods.

**Table S15.** Experimental conditions for dehydropolymerisation in THF using catalyst **1** under eudiometric conditions used in the COPSAI simulations.

| Entry | $[Ir]_{TOTAL}$ (mM) | $[H_3B{\cdot}NMeH_2]$ (M) | $[NMeH_2]$ (mM) |
|---|---|---|---|
| a | 2.0 | 2.0 | 1.0 |
| b | 2.0 | 2.0 | 10 |
| c | 2.0 | 2.0 | 20 |
| d | 2.0 | 2.0 | 60 |
| e | 2.0 | 1.0 | 3.0 |
| f | 2.0 | 0.5 | 1.8 |
| g | 2.0 | 1.0 | 10 |
| h | 1.0 | 2.0 | 2.2 |

## 7 Cost Analysis of Ir($^{t}$Bu-POCOP)H$_2$

**Table S16.** Cost Analysis for use of Ir($^{t}$Bu-POCOP)H$_2$ (**1**) in the synthesis of polyaminoboranes.

| Full cost of POCOP ligand (13.5 mmol) | Yield: | 85% | 4.04 | | | | | | | | | | | |
|---|---|---|---|---|---|---|---|---|---|---|---|---|---|---|
| Reagents (purchased through commerical sources) | Purchase Mass(g) | Purchase Volume (mL) | molecular weight (g/mol) | density (g/ml) | moles | Price | volume used (mL) | mass used (g) | moles used | Price/g | price/mole | price/ moles used | links accessed | Access date |
| Resorcinol | 100 | - | 110.11 | - | 0.91 | £24.30 | | 1.32 | 0.0120 | £0.24 | £26.76 | £0.32 | https://www.sigma | 16.01.25 |
| Sodium hydride | 10 | | 24 | | 0.42 | £52.70 | | 0.63 | 0.0262 | £5.27 | £126.48 | £3.31 | https://www.sigma | 16.01.25 |
| THF (dry and degssed) | 2223 | 2500 | 72.11 | 0.889 | 30.82 | £24.30 | 30.00 | 26.67 | 0.3699 | £0.01 | £0.79 | £0.29 | https://www.sigma | 16.01.25 |
| Di-tert-butylchlorophosphine | 24 | 25 | 180.66 | 0.951 | 0.13 | £216.00 | 5.00 | 4.76 | 0.0263 | £9.09 | £1,641.33 | £43.20 | https://www.therm | 16.01.25 |
| Pentane (dry and degrassed) | 1565 | 2500 | 72.15 | 0.626 | 21.69 | £182.00 | 40.00 | 25.04 | 0.3471 | £0.12 | £8.39 | £2.91 | https://www.sigma | 16.1.25 |
| Total | | | | | | | | 33.373 | | | £1,803.74 | £50.03 | | |
| Total (1 mmol) | | | | | | | | | | | | £3.71 | | |
| PMI | | | | | | | | 8.2606 | | | | | | |

| [Ir(COD)Cl]$_2$ (5.37 mmol) | Yield: | 85% | 3.61 | | | | | | | | | | | |
|---|---|---|---|---|---|---|---|---|---|---|---|---|---|---|
| Reagents (purchased through commerical sources) | Purchase Mass(g) | Purchase Volume (mL) | molecular weight (g/mol) | density (g/ml) | moles | Price | volume used (mL) | mass used | moles used | Price/g | price/mole | price/ moles used | Link Accessed | Access date |
| Iridium (III) chloride hydrate | 10 | - | 316.59 | - | 0.032 | £1,470.65 | | 4 | 0.013 | £147.07 | £46,559.31 | £588.26 | https://precmet.co | 16.01.25 |
| 1,5-cyclooctadiene | 882 | 1000 | 108.18 | 0.882 | 8.153 | £42.90 | 12.00 | 10.58 | 0.098 | £0.05 | £5.26 | £0.51 | https://www.therm | 16.01.25 |
| Water | | | 18 | 1 | | | | 60 | 3.333 | | | | | |
| Isopropanol | 1962.5 | 2500 | 60.1 | 0.785 | 32.654 | £90.90 | 120.00 | 94.2 | 1.567 | £0.05 | £2.78 | £4.36 | https://www.sigma | 16.01.25 |
| Methanol | 1977.5 | 2500 | 32.04 | 0.791 | 61.720 | £72.80 | 40.00 | 31.64 | 0.988 | £0.04 | £1.18 | £1.16 | https://www.sigma | 16.01.25 |
| Total | | | | | | | | 200.42 | | | £46,568.53 | £594.30 | | |
| Total (1 mmol) | | | | | | | | | | | | £110.59 | | |
| PMI | | | | | | | | 55.52 | | | | | | |

| Cost of Ir(POCOP)HCl (1.74 mmol) | Yield: | 89% | 1.09 | | | | | | | | | | | |
|---|---|---|---|---|---|---|---|---|---|---|---|---|---|---|
| Reagents (purchased through commerical sources) | Purchase Mass(g) | Purchase Volume (mL) | molecular weight (g/mol) | density (g/ml) | moles | Price | volume used (mL) | mass used (g) | moles used | Price/g | price/mole | price/ moles used | links accessed | Access date |
| $[Ir(COD)Cl]_2$ | | | 671.7 | | | | | 0.651 | 0.0010 | | £46,568.53 | £110.59 | See above (as synthesised p | |
| POCOP | | | 398.5 | | | | | 0.849 | 0.0021 | | £1,803.74 | £7.78 | See above (as synthesised p | |
| Toluene (dry and degassed) | 2163 | 2500 | 92.14 | 0.865 | 23.470 | £178.00 | 6.00 | 5.190 | 0.0563 | £0.08 | £7.58 | £0.43 | https://www.sigma | 16.01.25 |
| Pentane (dry and degrassed) | 1565 | 2500 | 72.15 | 0.626 | 21.691 | £182.00 | 10.00 | 6.260 | 0.0868 | £0.12 | £8.39 | £0.73 | https://www.sigma | 16.1.25 |
| Total | | | | | | | | 6.690 | | | £48,388.24 | £119.53 | | |
| Total (1 mmol) | | | | | | | | | | | | £68.69 | | |
| PMI | | | | | | | | 6.1374 | | | | | | |

| Cost of $Ir(POCOP)H_2$ (0.648 mmol) | Yield: | 81% | 0.384 | | | | | | | | | | | |
|---|---|---|---|---|---|---|---|---|---|---|---|---|---|---|
| Reagents (purchased through commerical sources) | Purchase Mass(g) | Purchase Volume (mL) | molecular weight (g/mol) | density (g/ml) | moles | Price | volume used (mL) | mass used (g) | moles used | Price/g | price/mole | price/ moles used | links accessed | Access date |
| Ir(POCOP)HCl | | | 626.17 | | | | | 0.500 | 0.0008 | | £48,388.24 | £54.96 | See above (as synthesised p | |
| $KO^tBu$ | 100 | | 112.21 | | 0.891 | £45.10 | | 0.117 | 0.0010 | £0.45 | £50.61 | £0.05 | https://www.sigma | 16.01.25 |
| Benzene (dry and degassed) | 874 | 1000 | 78.11 | 0.874 | 11.189 | £126.00 | 20.00 | 17.480 | 0.2238 | £0.14 | £11.26 | £2.52 | https://www.sigma | 16.1.25 |
| Pentane (dry and degrassed) | 1565 | 2500 | 72.15 | 0.626 | 21.691 | £182.00 | 10.00 | 6.260 | 0.0868 | £0.12 | £8.39 | £0.73 | https://www.sigma | 16.1.25 |
| Total | | | | | | | | 18.097 | | | £48,458.50 | £58.26 | | |
| Total (1 mmol) | | | | | | | | | | | | £89.90 | | |
| PMI | | | | | | | | 47.128 | | | | | | |

| Catalyst loading **1** (mol%, μmol) for the synthesis of 1 g of polymer | Catalyst cost |
|---|---|
| 0.1, 25 | £2.25 |
| 0.01, 2.5 | £0.22 |
| 0.001, 0.25 | £0.02 |

## 8 Computational Studies

### 8.1 Computational details.

DFT calculations were run with Gaussian 16 (Revision C.01).[12] Ir and P centers were described with Stuttgart RECPs and associated basis sets,[13] while 6-31G** basis sets[14, 15] were used for all other atoms. An additional set of *d*-orbital polarization functions was added to P ($\zeta^d$=0.387).[16] This basis set is denoted BS1. Structures were optimized using the BP86 functional[17, 18] using BS1 with all stationary points fully characterized via analytical frequency calculations as minima (all positive eigenvalues) or transition states (one negative eigenvalue). The final reported free energies are computed considering electronic energies obtained with the PBE0 functional (the justification for which is detailed below) with the def2-TZVP basis set (denoted as BS2),[19, 20] together with corrections for the effect of THF solvent through the PCM model[21] and dispersion (D3BJ).[22-24] The resultant electronic energies were then added to the thermochemical corrections from frequency calculations on the BP86(BS1) geometries.

The choice of functional for the energy correction with BS2 was based on a benchmark study of $H_2$ dissociation from Ir($^t$Bu-POCOP)$H_4$ in toluene reported by Wendt and co-workers.[25] Functional testing was performed using the BP86(BS1)-optimised geometries with electronic energies recomputed with the functional of choice using BS2 and including a correction for toluene solvent. For PBE,[26, 27] BLYP,[17, 28] TPSS,[29] B3LYP,[17, 28, 30, 31] B3PW91,[18, 30-33] and PBE0[26, 34] a D3BJ dispersion correction was also included; for M06-L,[35] M06, M06-2X,[36] B97-D,[37] B97-D3(BJ) and ωB97X-D,[38] no additional dispersion correction was applied. The DH‡ and DG‡ values computed at 298 K are reported in Table S17; experimental data are: DH‡ = 16.8 ±0.3 kcal/mol and DG‡ = 15.6 ±0.5 kcal/mol.[25]

**Table S17.** Benchmark study based on $H_2$ loss from Ir($^t$Bu-POCOP)$H_4$..

| XC | BP86 | BLYP | B3LYP | PBE | PBE0 | B97D3 | B97D | Experiment[25] |
|---|---|---|---|---|---|---|---|---|
| DG‡ | 14.46 | 9.92 | 11.22 | 14.64 | 15.85 | 10.27 | 8.44 | 15.6 ±0.5 |
| DH‡ | 15.01 | 10.47 | 11.77 | 15.19 | 16.40 | 10.82 | 8.99 | 16.8 ±0.3 |
| **XC** | **M06** | **WB97xD** | **TPSS** | **B3PW91** | **M06L** | **M062X** | **M06** | **Experiment[25]** |
| DG‡ | 6.37 | 13.11 | 13.62 | 15.12 | 7.34 | 7.81 | 6.37 | 15.6 ±0.5 |
| DH‡ | 6.92 | 13.66 | 14.17 | 15.67 | 7.89 | 8.36 | 6.92 | 16.8 ±0.3 |

The PBE0(BS2/toluene/BJD3)//BP86(BS1) protocol (highlighted) provided the closest agreement for both the experimental free energy and enthalpy of activation. With BP86 "Ir($^t$Bu-POCOP)$H_4$" is computed to be more stable as Ir($^t$Bu-POCOP)$(H)_2$($h^2$-$H_2$), **2**, where the $h^2$-$H_2$ is cis to the aryl C. The alternative tetrahydride is 0.2 kcal/mol higher with a transition state linking the two at 0.0 kcal/mol. No minimum with $h^2$-$H_2$ trans to the aryl C was located. The results here refer to data computed with toluene; data with THF are detailed below.

## 8.2 Computed Dehydrogenation Mechanisms.

The lowest energy pathways for Mechanisms A and B shown in Figure 8b in the main text are reproduced here in Figure S79 and Figure S80 respectively. Interconversion between **2** and **2'** is included in Figure S79.

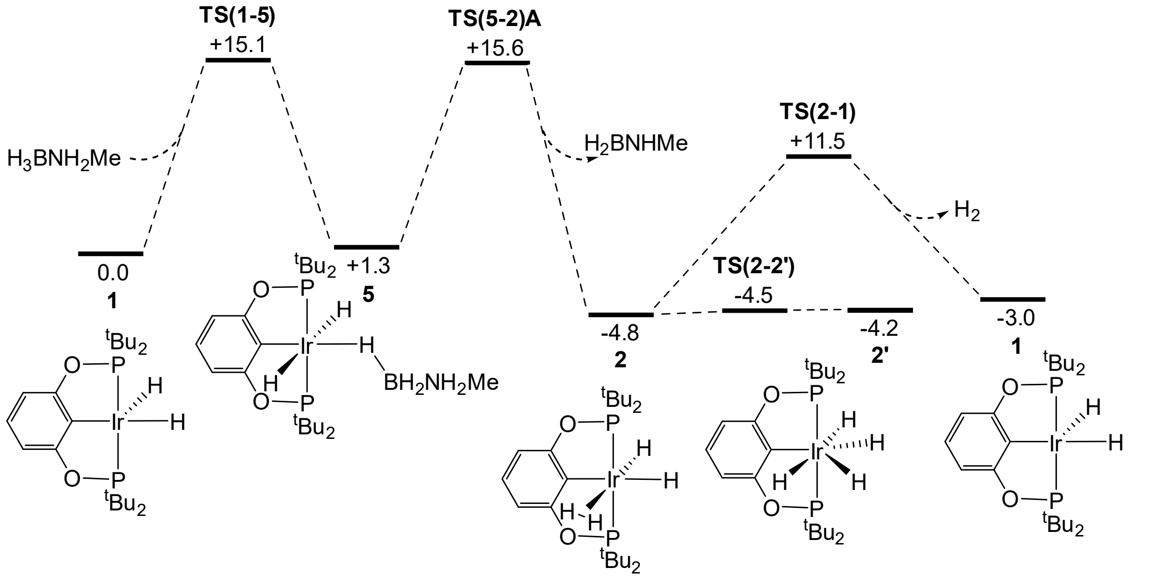


**Figure S79.** Computed free energy profile (kcal/mol) for Mechanism A, concerted B–H/N–H activation at **5** followed by $H_2$ loss from **2**. Level of theory: PBE0(BS2/THF/D3BJ)//BP86(BS1).

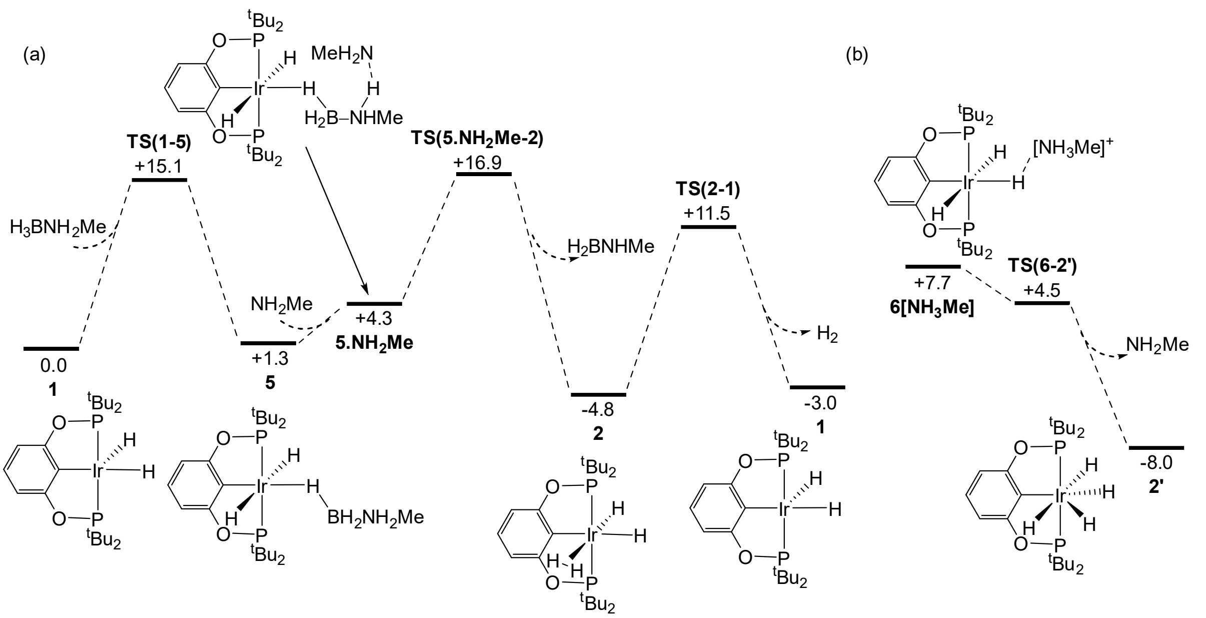


**Figure S80.** (a) Computed free energy profile (kcal/mol) for Mechanism B, base-promoted hydride transfer at **5**. Level of theory: PBE0(BS2/THF/D3BJ)//BP86(BS1). (b) Ion-pair **[6][NH₃Me]** could only be located when solvent was included in the optimization protocol; however ,proton transfer is barrierless on the free energy surface and in this case leads to **2'**. Level of theory: BP86(BS2/THF/D3BJ)//BP86(BS1).

Alternative $H_3BNH_2Me$ dehydrogenation pathways were assessed following Musgrave and Paul's earlier work.[39] Figure S81 involves formation of $\mathbf{5_{cis}}$ followed by $H_2$ loss to an Ir(I) intermediate, **7**, and concerted MMAB dehydrogenation and has an overall barrier of 26.0 kcal/mol.

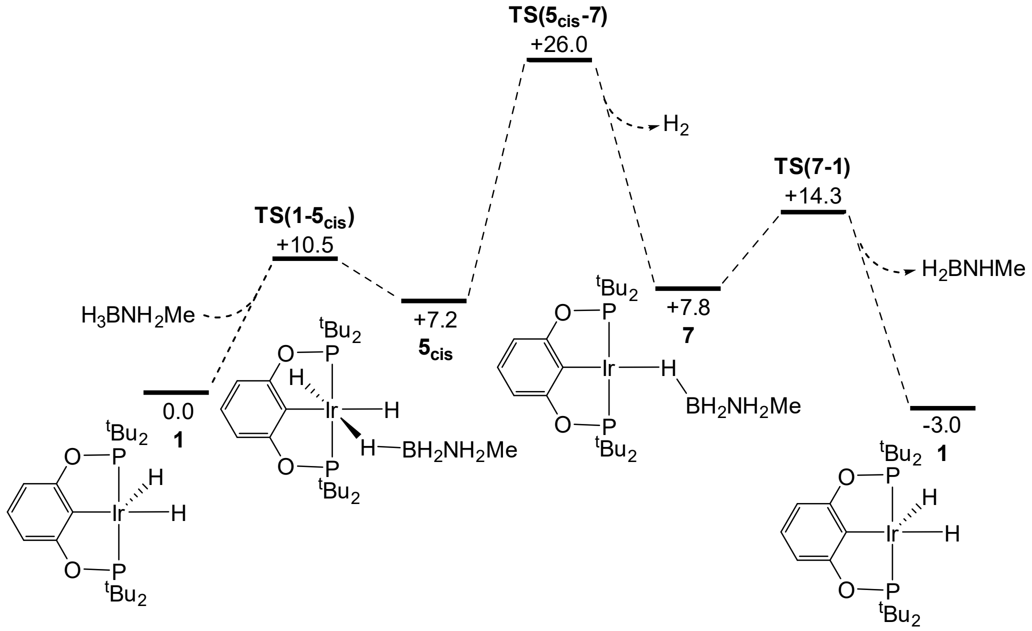


**Figure S81.** Computed free energy profile (kcal/mol) for alternative dehydrogenation mechanism involving $H_2$ loss from $\mathbf{5_{cis}}$ followed by concerted B–H/N–H activation at Ir(I) complex **7**. Level of theory: PBE0(BS2/THF/D3BJ)//BP86(BS1).

Figure S82 shows the case where reaction proceeds via $H_2$ loss from **1**, MMAB addition to form **7** followed by concerted MMAB dehydrogenation. This has an overall barrier of 17.2 kcal/mol and so is potentially competitive with Mechanism A in Figure 8b/Figure S79. However, this process is less likely to occur under conditions where $H_2$ is being produced; moreover, no transition state for $H_2$ loss from **1** could be located and so the energy of 14e **8** represents a lower limit of the barrier involved.

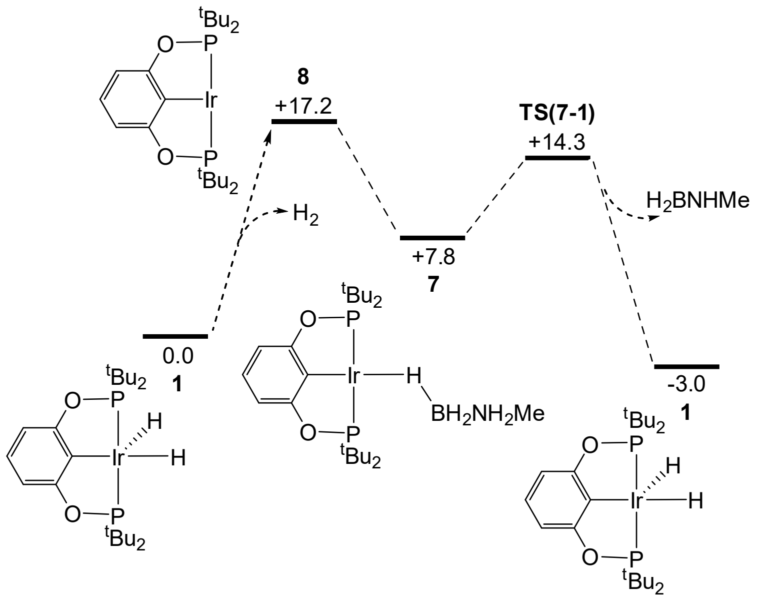


**Figure S82.** Computed free energy profile (kcal/mol) for alternative dehydrogenation mechanism involving $H_2$ loss from **1** followed by concerted B–H/N–H activation at Ir(I) complex **7**. Level of theory: PBE0(BS2/THF/D3BJ)//BP86(BS1).

A mechanism based on initial N-H activation is shown in Figure S83 but this step has a prohibitively high barrier of 34.3 kcal/mol and so can be disregarded.

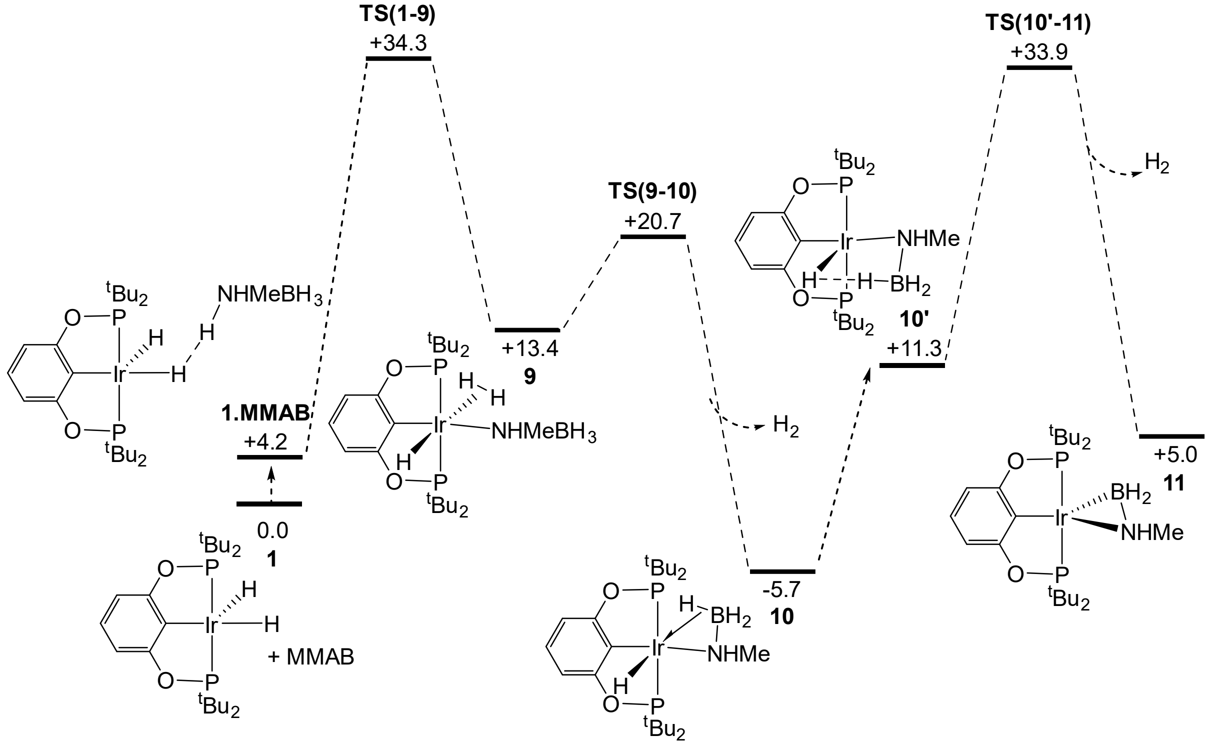


**Figure S83.** Computed free energy profile (kcal/mol) for an alternative dehydrogenation mechanism involving $H_2$ loss from **1** followed by concerted B–H/N–H activation at Ir(I) complex **7**. Level of theory: PBE0(BS2/THF/D3BJ)//BP86(BS1).

Pathways for the formation of the *cis*-isomers of **4** and **5** are shown in Figure S84. In both cases these processes are kinetically more accessible than formation of the trans-isomers, presumably as they do not require any opening of the H–Ir–H angle. However, $\mathbf{4_{cis}}$ and $\mathbf{5_{cis}}$ are respectively 4.6 kcal/mol and 5.9 kcal/mol higher in energy than the trans isomers of **4** and **5**. A transition state for concerted B-H/N-H activation at $\mathbf{5_{cis}}$ was located at +19.9 kcal/mol and led directly to tetrahydride **2'** plus free $H_2B{=}NHMe$; this is 4.3 kcal/mol higher than **TS(5-2)A**. In addition, a transition state for isomerisation between $\mathbf{5_{cis}}$ and **5** was also located at +17.2 kcal/mol and involves transfer of the $\{BH_2NH_2Me\}$ moiety from an axial to an equatorial hydride.

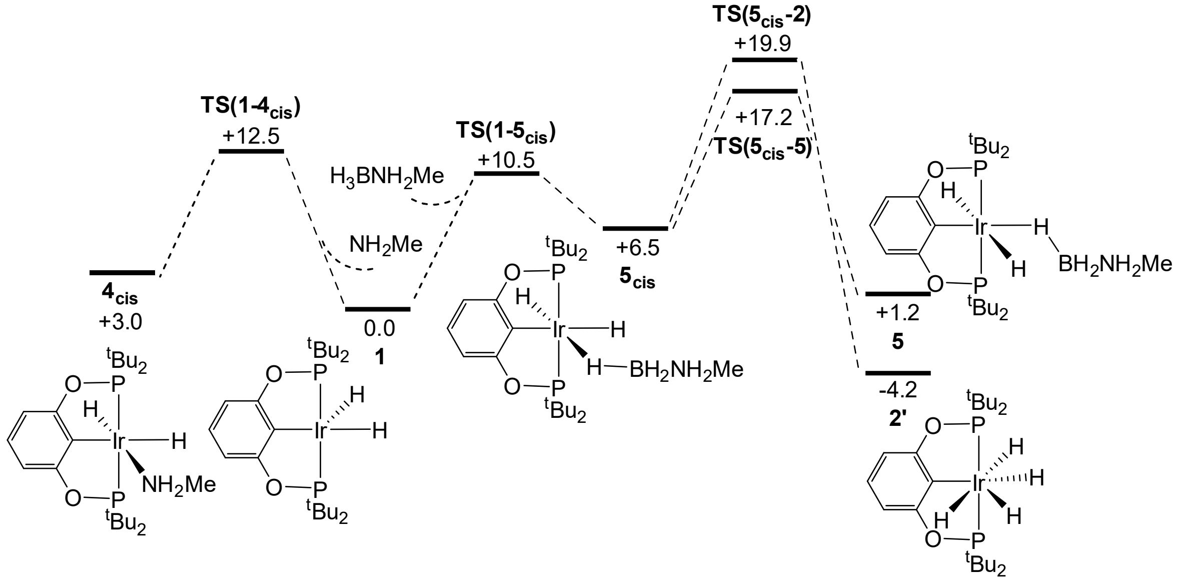


**Figure S84.** Computed free energy profiles (kcal/mol) for interconversion between **1** , $\mathbf{4_{cis}}$, $\mathbf{5_{cis}}$ and **5**. Level of theory: PBE0(BS2/THF/D3BJ)//BP86(BS1).

Figure S85 shows alternative forms of the base-assisted hydride transfer transition state, **TS(5-2)B**, the key transition state along Mechanism B. These differ from **TS(5-2)B** in the main text via the relative orientation of the two NMe groups (**TS(5-6)2**), the positioning of the MMAB moiety, originating from $\mathbf{5_{cis}}$ (**TS(5-6)3**, **TS(5-6)4**) or the positioning of $NH_2Me$ anti to the metal center (**TS(5-6)5**, **TS(5-6)6**). The latter are particularly disfavored, highlighting the importance of the network of dihydrogen and hydrogen bonding interactions in stabilising the lowest energy form of **TS(5-2)B**, as discussed in the main text.

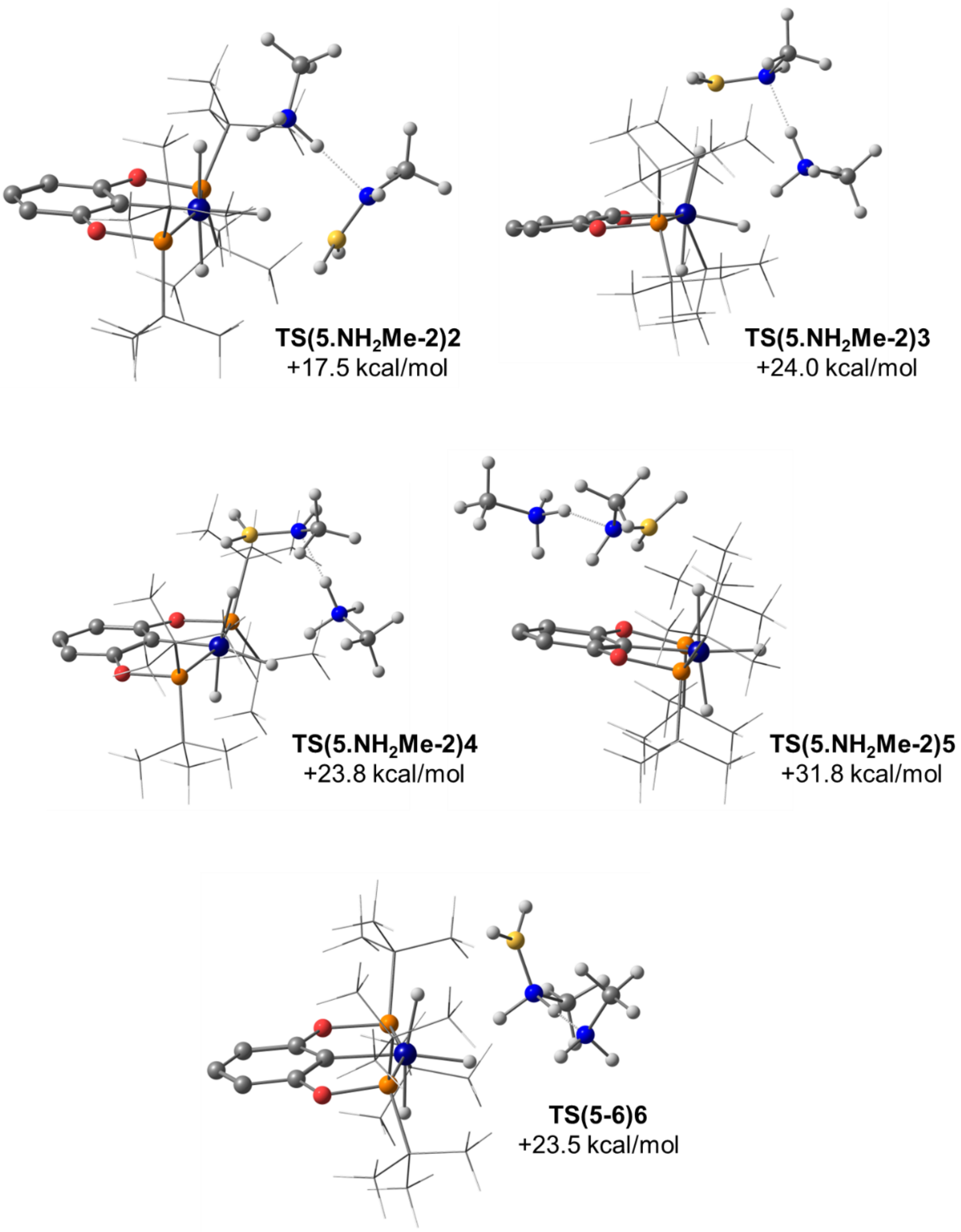


**Figure S85.** Alternative transition states located for Mechanism B, base-assisted hydride transfer. transition states ($^{t}$Bu groups in wireframe; backbone H atoms omitted for clarity). Level of theory: PBE0(BS2/THF/D3BJ)//BP86(BS1).

## 8.3 Functional testing.

Additional functional testing was performed using the BP86(THF/BS1)-optimised geometries with electronic energies recomputed with the functional of choice using BS2 and including a correction for THF solvent. For PBE,[26, 27] BLYP,[17, 28] TPSS,[29] B3LYP,[17, 28, 30, 31] B3PW91,[18, 30-33] and PBE0[26, 34] a D3BJ dispersion correction was also included; for M06-L,[35] M06, M06-2X,[36] B97-D,[37] B97-D3 and ωB97X-D,[38] no additional dispersion correction was applied. The resultant electronic energies were then added to the thermochemical corrections from the original BP86(THF/BS1)-optimised geometries.

Benchmarking was performed against the experimental observations that,

(i) Ir($^t$Bu-POCOP)$(H)_4$, **2**, is the resting state in catalysis;

(ii) The rate-limiting step should be $H_2$ loss from **2** and should have an overall free energy of activation consistent with the experimental value of 16(2) kcal/mol;

(iii) the favorable formation of off-cycle species Ir($^t$Bu-POCOP)(H)$(BH_4)$, **3**, and *trans*-Ir($^t$Bu-POCOP)$(H)_2(NH_2Me)$, **4** from Ir($^t$Bu-POCOP)$(H)_2$, **1**.

The free energy difference for the reaction of Ir($^t$Bu-POCOP)(H)$_2$, **1**, with $H_3BNH_2Me$ to give Ir($^t$Bu-POCOP)($h^2$-$H_2$)(H)$_2$, **2**, and $H_2BNHMe$ computed with a suite of functionals is shown in Figure S86. A negative value indicates this reaction is favored **2** and would be consistent with the observation of **2** as the resting state during catalysis.

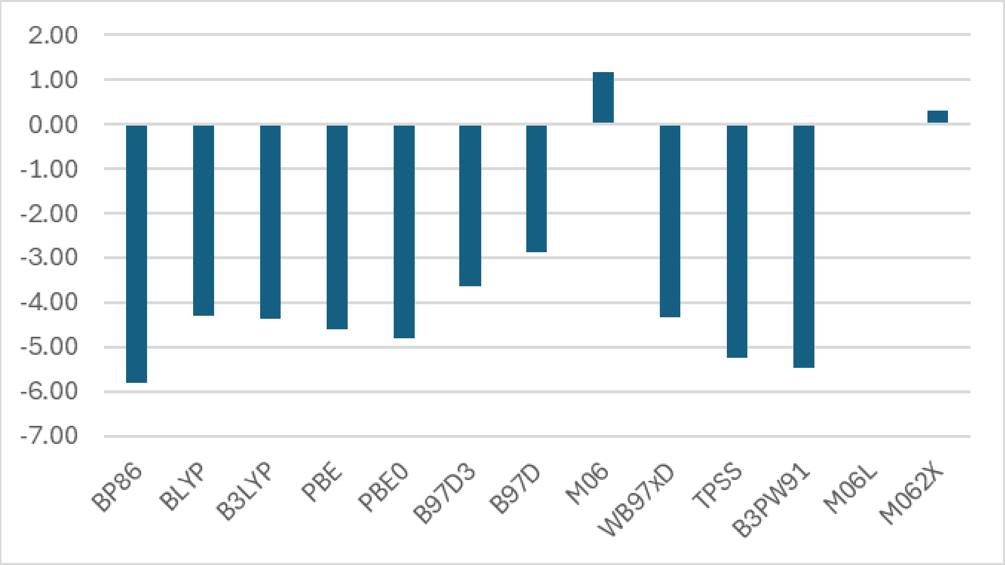


**Figure S86.** Plot of showing free energy differences (kcal/mol) calculated for the reaction **1** + $H_3BNH_2Me$ → **2** + $H_2BNHMe$.

Figure S87 compares computed activation barriers for (i) $H_2$ loss from **2** (via **TS(2-1)**, orange), concerted B–H/N–H dehydrogenation in **5** (via **TS(5-2)A**, Mechanism A, green) and base-promoted hydride transfer from **5** (via **TS(5-2)B**, Mechanism B, blue).

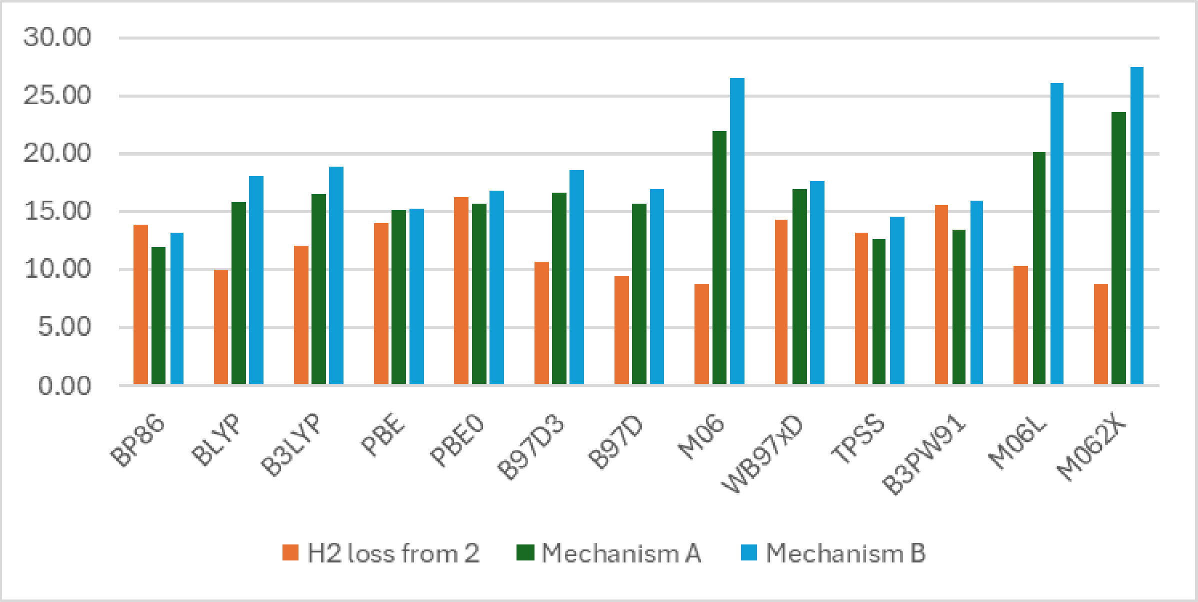


**Figure S87.** Plot of showing computed activation barriers (kcal/mol) for (i) $H_2$ loss from **2** (orange), concerted B–H/N–H dehydrogenation via **TS(2-5)A** (Mechanism A, green) and base-promoted hydride transfer via **TS(5-2)B** (Mechanism B, blue).

With all functionals dehydrogenation via Mechanism A has a lower barrier than the alternative process via Mechanism B. Only BP86, PBE0, TPSS and B3PW91 compute $H_2$ loss from **2** to be rate-limiting as suggested experimentally and of these PBE0, TPSS and B3PW91 provide barriers that are within the experimental value of 16 $\pm$ 2 kcal/mol.

Figure S88 shows the free energies of **3**, **4** and **5** computed relative to **1**. Here amine adduct **4** is expected to be more stable than **1** (and **3**) as this species is seen after catalysis. This is seen for BP86, PBE0, wB97x-D, TPSS and B3PW91. Similarly, these functionals provide small positive or negative free energies for borohydride **3**, consistent with its observation during the induction period.

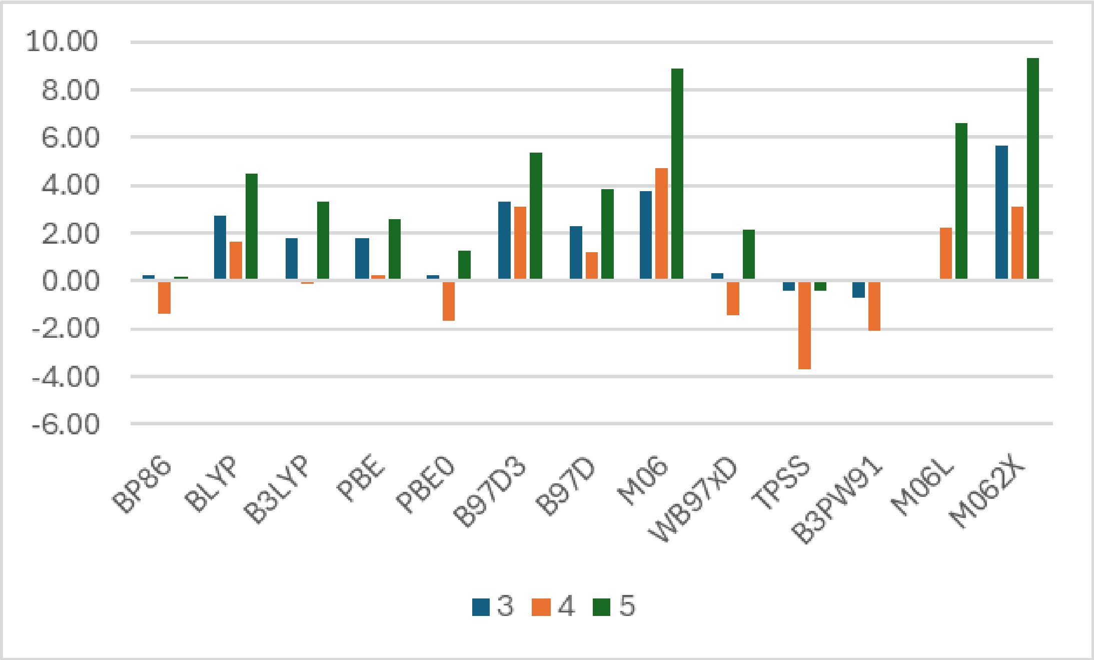


**Figure S88.** Plot of showing computed free energies (kcal/mol) for Ir($^t$Bu-POCOP)(H)($BH_4$), **3**, and *trans*-Ir($^t$Bu-POCOP)$(H)_2$($NH_2$Me), **4**, and *trans*-Ir($^t$Bu-POCOP)$(H)_2$($H_3BNH_2$Me), **5**, relative to Ir($^t$Bu-POCOP)$(H)_2$, **1**.

Barriers for the competing processes originating from **5** are compared in Figure S89; dehydrogenation via **TS(5-2)** vs. B–N bond cleavage via **TS(5-3)**. In all cases dehydrogenation is favored with the difference between the two being lowest with PBE0.

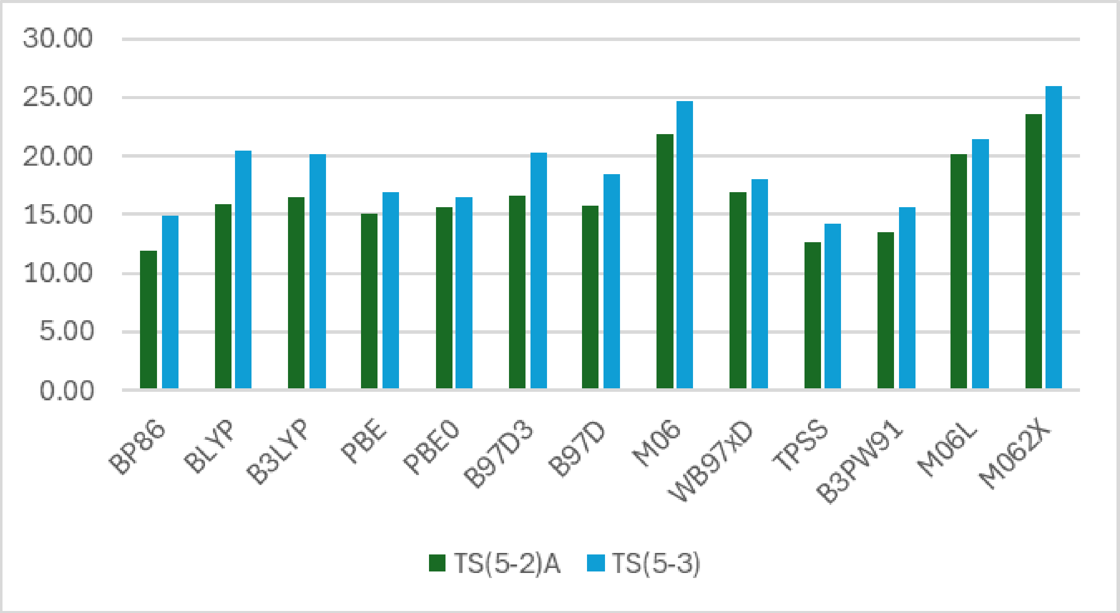


**Figure S89.** Plot comparing computed free energy barriers (kcal/mol) for concerted B-H/N-H dehydrogenation in **5** (via **TS(5-2)**, orange) and B–N bond cleavage (via **TS(5-3)**, blue).

**9 Cartesian coordinates (Å) and energies (a.u.) for the computed structures.**

**SCF(PBE0) refers to electronic energies computed with the PBE0 functional including corrections for THF solvent, a D3BJ correction and with BS2. means**

**(i) Small Molecules**

```
H3BNH2Me
SCF(BP86/BS1)= -122.516499833
G(298 K) = -122.446177
SCF(PBE0) = -122.422162947
Lowest Frequencies = 186.5917cm-1,
244.8337cm-1

11
H3BNH2Me
N  -0.00477  0.55345 -0.00004
B   1.40273 -0.30953  0.00009
C  -1.23637 -0.28574 -0.00004
H  -0.00743  1.17127 -0.82170
H   2.28713  0.53263  0.00007
H   1.36512 -0.97742  1.02234
H   1.36522 -0.97758 -1.02205
H  -0.00751  1.17139  0.82153
H  -1.20489 -0.92867 -0.89090
H  -1.20498 -0.92854  0.89091
H  -2.15471  0.32484 -0.00013

H2BNHMe
SCF(BP86/BS1)= -121.338180465
G(298 K) = -121.288670
SCF(PBE0) = -121.236156743
Lowest Frequencies = 189.3943cm-1,
386.8547cm-1

9
H2BNHMe
B  -1.37055  0.25351 -0.00007
N  -0.14561 -0.42014  0.00000
H  -0.13886 -1.44087  0.00007
H  -2.38704 -0.39475  0.00013
H  -1.37017  1.46038  0.00002
C   1.18980  0.17204  0.00002
H   1.09177  1.26801 -0.00005
H   1.76872 -0.12573  0.89415
H   1.76878 -0.12583 -0.89405

H2
SCF(BP86/BS1)= -1.17646512732
G(298 K) = -1.178045
SCF(PBE0) = -1.16839468293
Lowest Frequencies = 4357.5167cm-1,
cm-1

2
H2
H   0.00000  0.00000  0.37521
H   0.00000  0.00000 -0.37521

NH2Me
SCF(BP86/BS1)= -95.8564889673
G(298 K) = -95.816966
SCF(PBE0) = -95.7821518026
Lowest Frequencies = 327.0701cm-1,
839.2238cm-1

7
NH2Me
N   0.75717 -0.00000 -0.12904
H   1.14376 -0.81560  0.35953
C  -0.70736  0.00000  0.01802
H  -1.09981 -0.00004  1.06009
H  -1.12188  0.88659 -0.49194
H  -1.12190 -0.88654 -0.49201
H   1.14376  0.81560  0.35953

[NH3Me]+
SCF(BP86/BS1)= -96.2194966389
G(298 K) = -96.165228
SCF(PBE0) = -96.2275163278
Lowest Frequencies = 294.3499cm-1,
893.1842cm-1

8
[NH3Me]+
N  -0.71438  0.00000  0.00000
H  -1.09441 -0.67845  0.68250
H  -1.09439  0.93030  0.24625
C   0.80519 -0.00000 -0.00000
H   1.15086 -1.00730 -0.26675
H   1.15089  0.73464 -0.73894
H   1.15090  0.27264  1.00571
H  -1.09438 -0.25185 -0.92878

BH3
SCF(BP86/BS1)= -26.5977256761
G(298 K) = -26.591300
SCF(PBE0) = -26.5669630962
Lowest Frequencies = 1126.3397cm-1,
1168.0122cm-1

4
BH3
B   0.00001  0.00000 -0.00001
H   0.61150 -1.03580  0.00002
H   0.59142  1.04739  0.00002
H  -1.20297 -0.01159  0.00002
```

**(ii) Figure 8(a)**

4
SCF(BP86/BS1)= -1227.11764680
G(298 K) = -1226.546764
SCF(PBE0) = -1895.50621054
Lowest Frequencies = 22.1628cm-1, 33.1617cm-1

```
75
4
Ir -0.00001 -0.42486  0.01717
P   2.28908 -0.00561  0.01553
P  -2.28911 -0.00564  0.01562
O   2.41991  1.67026 -0.35279
C   1.19291  2.33569 -0.44278
O  -2.41998  1.67038 -0.35203
C  -0.00003  1.59330 -0.30799
C  -3.87685 -2.12735 -1.04737
H  -2.99548 -2.77556 -0.89438
H  -4.44599 -2.54954 -1.89655
H  -4.51852 -2.20192 -0.15345
C   3.17489 -0.04937  1.74616
C  -3.47482 -0.67260 -1.36976
C  -1.19298  2.33575 -0.44241
C   3.47499 -0.67309 -1.36944
C   2.87005 -1.41782  2.39459
H   1.77947 -1.55323  2.50215
H   3.31394 -1.45467  3.40706
H   3.29414 -2.26304  1.82292
C   3.87743 -2.12756 -1.04623
H   4.51942 -2.20133 -0.15246
H   4.44641 -2.55018 -1.89530
H   2.99634 -2.77599 -0.89248
C  -2.66969 -0.64068 -2.68814
H  -2.36254  0.38655 -2.94512
H  -3.31056 -1.02276 -3.50506
H  -1.75319 -1.24729 -2.63090
C  -1.21952  3.71993 -0.67737
H  -2.17915  4.23692 -0.76970
C   2.66996 -0.64214 -2.68791
H   1.75365 -1.24901 -2.63044
H   3.31103 -1.02441 -3.50457
H   2.36246  0.38486 -2.94543
C   2.51932  1.06897  2.58830
H   2.77105  2.06634  2.19485
H   2.89537  0.99987  3.62643
H   1.42229  0.96433  2.60524
C  -4.72935  0.20943 -1.56618
H  -5.45280  0.12383 -0.74382
H  -5.24383 -0.11110 -2.49155
H  -4.45408  1.26923 -1.68164
C  -4.69622  0.19384  1.71298
H  -5.06731  0.25982  2.75332
H  -5.25155 -0.62349  1.22422
H  -4.94711  1.14285  1.21273
C  -2.51978  1.06819  2.58868
H  -2.77175  2.06563  2.19555
H  -1.42271  0.96382  2.60559
H  -2.89581  0.99867  3.62678
C   4.69600  0.19482  1.71297
H   5.25152 -0.62252  1.22445
H   5.06704  0.26119  2.75330
H   4.94670  1.14375  1.21244
C  -2.86999 -1.41862  2.39423
H  -3.31404 -1.45589  3.40661
H  -1.77940 -1.55378  2.50193
H  -3.29379 -2.26376  1.82222
C  -3.17506 -0.05003  1.74617
C   4.72931  0.20916 -1.56625
H   4.45375  1.26880 -1.68254
H   5.24409 -0.11191 -2.49127
H   5.45259  0.12439 -0.74366
N   0.00018 -2.65684  0.36520
H   0.81528 -2.85589  0.95527
H  -0.81480 -2.85588  0.95545
C   1.21945  3.71987 -0.67774
H   2.17907  4.23682 -0.77036
C  -0.00004  4.40690 -0.79370
H  -0.00004  5.48627 -0.97641
C  -0.00004 -3.56908 -0.80668
H   0.88549 -3.35447 -1.42141
H  -0.88604 -3.35464 -1.42078
H   0.00017 -4.63631 -0.51455
H  -0.00004 -0.28876  1.69888
H   0.00007 -0.67608 -1.62569
```

TS(1-4)
SCF(BP86/BS1)= -1227.08747854
G(298 K) = -1226.524369
SCF(PBE0) = -1895.46526196
Lowest Frequencies = -117.1088cm-1, 19.9915cm-1

```
75
TS(1-4)
Ir  0.00668  0.31844  0.11892
P  -2.26897 -0.09925  0.07032
P   2.29768  0.00723  0.06787
O  -2.37151 -1.64788 -0.70890
C  -1.14103 -2.26769 -0.92488
O   2.47156 -1.54114 -0.69918
C   0.04957 -1.55857 -0.65304
C   3.93851  2.30696 -0.36674
H   3.12387  2.87554  0.11538
H   4.42623  2.98155 -1.09472
H   4.68573  2.06098  0.40513
C  -3.12372 -0.54229  1.74349
C   3.42219  1.05380 -1.10552
C   1.27056 -2.21521 -0.91981
C  -3.43103  0.91153 -1.09263
C  -3.07500  0.67849  2.68695
H  -2.04425  1.05941  2.78530
H  -3.42546  0.37031  3.68963
H  -3.72414  1.50314  2.35078
C  -3.96908  2.14892 -0.34319
H  -4.69921  1.88252  0.43851
H  -4.48581  2.81028 -1.06340
H  -3.15681  2.73400  0.12142
C   2.51467  1.49354 -2.27821
H   2.12349  0.62515 -2.83374
H   3.11013  2.10649 -2.98109
```

```
H   1.65343  2.08690 -1.92880
C   1.31831 -3.53212 -1.40533
H   2.28596 -4.00878 -1.58675
C  -2.54396  1.38153 -2.26923
H  -1.70402  2.00361 -1.91819
H  -3.16418  1.97541 -2.96718
H  -2.12866  0.52716 -2.82939
C  -2.24933 -1.66859  2.34375
H  -2.27834 -2.58202  1.72883
H  -2.62915 -1.91763  3.35211
H  -1.19796 -1.34740  2.43963
C   4.60584  0.23924 -1.67065
H   5.33479 -0.04621 -0.89756
H   5.13747  0.85881 -2.41725
H   4.25750 -0.67752 -2.16989
C   4.63576 -0.83915  1.57666
H   5.01431 -1.20063  2.55118
H   5.29867 -0.01783  1.25728
H   4.72378 -1.66717  0.85457
C   2.34420 -1.53529  2.35841
H   2.40361 -2.45256  1.75137
H   1.28230 -1.25193  2.45737
H   2.73869 -1.76095  3.36664
C  -4.56846 -1.05200  1.57603
H  -5.26293 -0.25184  1.27060
H  -4.92880 -1.44104  2.54686
H  -4.62823 -1.87270  0.84278
C   3.07904  0.84587  2.67266
H   3.43982  0.56201  3.67885
H   2.03511  1.19039  2.76664
H   3.69831  1.68985  2.32803
C   3.17309 -0.38291  1.74306
C  -4.59474  0.06382 -1.64939
H  -4.22464 -0.84561 -2.14684
H  -5.14438  0.66644 -2.39674
H  -5.31402 -0.23673 -0.87299
N  -0.07466  3.46726  0.00013
H  -0.44674  3.17340  0.90901
H   0.93914  3.53706  0.12500
C  -1.12850 -3.58551 -1.41026
H  -2.07362 -4.10401 -1.59565
C   0.10955 -4.20727 -1.64873
H   0.13273 -5.23586 -2.02247
C  -0.63641  4.75554 -0.43472
H  -1.72454  4.64856 -0.58050
H  -0.20504  5.03201 -1.41159
H  -0.47189  5.60635  0.26113
H  -0.01104  1.08743  1.65490
H   0.00537  0.40998 -1.47184
```

1
SCF(BP86/BS1)= -1131.24545551
G(298 K) = -1130.741666
SCF(PBE0) = -1799.69208534
Lowest Frequencies = 22.9797cm-1, 29.7745cm-1

68
1

```
C  -0.00000  4.30331 -0.16264
C  -1.23059  3.62410 -0.13426
C  -1.21181  2.21893 -0.07368
C  -0.00000  1.48500 -0.04040
C   1.21180  2.21893 -0.07367
C   1.23058  3.62410 -0.13425
Ir -0.00000 -0.58836  0.04900
P   2.26933 -0.19876  0.01103
C   3.28558 -0.51060  1.61603
O   2.42482  1.54919 -0.04845
O  -2.42482  1.54918 -0.04847
P  -2.26933 -0.19877  0.01103
C  -3.28557 -0.51058  1.61604
C  -3.21686 -0.64591 -1.60195
C   3.21685 -0.64589 -1.60196
H   0.00001 -1.98355  0.86738
H  -0.00001 -1.96843 -0.76634
H  -2.17970  4.16642 -0.15930
H   2.17969  4.16642 -0.15928
H  -0.00001  5.39789 -0.20984
C  -3.13990 -2.17493 -1.80974
C  -4.68126 -0.16720 -1.62156
C  -2.42981  0.05718 -2.73388
C  -4.56295  0.34793  1.71803
C  -3.61938 -2.01387  1.71673
C  -2.32650 -0.11811  2.76626
C   4.68125 -0.16720 -1.62156
C   3.13987 -2.17491 -1.80977
C   2.42981  0.05722 -2.73388
C   3.61942 -2.01388  1.71669
C   4.56295  0.34793  1.71803
C   2.32652 -0.11815  2.76627
H  -5.09222 -0.31004 -2.63867
H  -5.31764 -0.74458 -0.93114
H  -4.76666  0.90282 -1.37232
H  -2.86901 -0.23089 -3.70724
H  -2.48053  1.15399 -2.64371
H  -1.36759 -0.24061 -2.72534
H  -3.56981 -2.42462 -2.79767
H  -2.09649 -2.52997 -1.78892
H  -3.71032 -2.73498 -1.05100
H  -4.98880  0.23069  2.73219
H  -4.34202  1.41529  1.56222
H  -5.33871  0.04438  0.99942
H  -4.03057 -2.22716  2.72091
H  -4.38017 -2.32186  0.97987
H  -2.72283 -2.64275  1.58076
H  -2.82791 -0.31057  3.73362
H  -1.39070 -0.70058  2.72766
H  -2.06318  0.95188  2.72620
H   2.86900 -0.23085 -3.70724
H   1.36758 -0.24056 -2.72534
H   2.48054  1.15403 -2.64370
H   5.09221 -0.31004 -2.63868
H   4.76666  0.90282 -1.37232
H   5.31763 -0.74459 -0.93115
H   3.56978 -2.42459 -2.79770
H   3.71029 -2.73497 -1.05103
H   2.09646 -2.52994 -1.78895
H   4.03061 -2.22718  2.72086
H   2.72288 -2.64277  1.58071
H   4.38021 -2.32185  0.97981
H   2.82794 -0.31063  3.73361
```

```
H   2.06318  0.95183  2.72623
H   1.39073 -0.70064  2.72766
H   4.98881  0.23068  2.73218
H   5.33871  0.04442  0.99940
H   4.34200  1.41530  1.56225
```

TS(1-5)
SCF(BP86/BS1)= -1253.75515638
G(298 K) = -1253.159329
SCF(PBE0) = -1922.11187659
Lowest Frequencies = -133.0998cm-1, 23.5797cm-1

```
79
TS(1-5)
C   1.17541 -3.96988 -0.37562
C  -0.05719 -4.64387 -0.41439
C  -1.27141 -3.93808 -0.36504
C  -1.23619 -2.53616 -0.28301
C  -0.02068 -1.81873 -0.23132
C   1.17510 -2.56803 -0.29100
O  -2.44347 -1.84383 -0.25110
P  -2.29979 -0.13148  0.00482
C  -3.15633 -0.00721  1.72964
C  -2.31145 -0.91266  2.65694
Ir  0.00156  0.20641 -0.02532
P   2.28271 -0.19237 -0.00866
C   3.36464  0.42706 -1.47793
C   2.72430 -0.16529 -2.75535
O   2.40182 -1.90484 -0.25526
C  -3.43646  0.47758 -1.42920
C  -2.54940  0.49349 -2.69615
C   3.19547 -0.09427  1.69213
C   2.16279 -0.61284  2.72150
C   4.45240 -0.98677  1.76868
C   3.53858  1.37454  2.01071
C   3.27732  1.96791 -1.53959
C   4.83476 -0.02902 -1.39405
C  -4.62883 -0.47401 -1.67009
C  -3.92933  1.91046 -1.13722
C  -3.07319  1.44705  2.24022
C  -4.61593 -0.50231  1.72707
B  -0.19942  3.68425 -1.29312
N   0.44315  3.93428  0.18140
H  -2.23476 -4.45529 -0.39155
H   2.12492 -4.51168 -0.40946
H  -0.07161 -5.73665 -0.47642
H   0.01643  1.47467  1.15583
H  -0.06492  2.46493 -1.48019
H  -0.01967 -0.12569 -1.56868
H   0.15195  3.11645  0.76580
H  -1.37774  4.00039 -1.21733
H   1.46496  3.84350  0.10679
C   0.10162  5.22971  0.82373
H   0.43711  4.35722 -2.08997
H   3.83500  2.32521 -2.42519
H   2.23289  2.30761 -1.64475
H   3.73135  2.44841 -0.65551
H   5.34137  0.22584 -2.34367
H   5.38469  0.48179 -0.58695
H   4.92230 -1.11801 -1.24976
H   3.29239  0.19294 -3.63363
H   2.75192 -1.26648 -2.75152
H   1.67773  0.15873 -2.87276
H   3.90100  1.44774  3.05293
H   4.33447  1.77105  1.35776
H   2.64849  2.02256  1.92373
H   4.79275 -1.02951  2.82011
H   4.23239 -2.01476  1.44102
H   5.28958 -0.60108  1.16901
H   2.60932 -0.56447  3.73252
H   1.24404 -0.00274  2.71431
H   1.88307 -1.66080  2.52353
H  -4.45854  2.29149 -2.03035
H  -4.64353  1.94349 -0.29744
H  -3.09395  2.60177 -0.93374
H  -3.16913  0.81019 -3.55576
H  -1.71034  1.19964 -2.59483
H  -2.14539 -0.50770 -2.92322
H  -5.16977 -0.13044 -2.57151
H  -4.29269 -1.50745 -1.84464
H  -5.34699 -0.47987 -0.83702
H  -3.44657  1.48289  3.28071
H  -2.03066  1.80697  2.23565
H  -3.68475  2.14258  1.64356
H  -4.97876 -0.55676  2.77071
H  -5.28896  0.18226  1.18520
H  -4.70463 -1.50932  1.28823
H  -2.69690 -0.82622  3.68995
H  -2.36592 -1.97091  2.35612
H  -1.25077 -0.60788  2.65849
H   0.42432  6.04729  0.16197
H  -0.99097  5.28157  0.93050
H   0.58129  5.33444  1.81143
```

5
SCF(BP86/BS1)= -1253.77410896
G(298 K) = -1253.175442
SCF(PBE0) = -1922.13681572
Lowest Frequencies = 22.0845cm-1, 29.3483cm-1

```
79
5
C  -1.28296 -3.51906 -1.60078
C  -0.07258 -4.18758 -1.84615
C   1.15484 -3.59051 -1.51682
C   1.15017 -2.30649 -0.94593
C  -0.03497 -1.58360 -0.69587
C  -1.24025 -2.23715 -1.02833
O   2.38038 -1.73579 -0.62234
P   2.28455 -0.16941  0.09869
C   3.52176  0.71303 -1.11457
C   2.76155  0.91127 -2.44470
Ir -0.01167  0.28320  0.14117
P  -2.30883 -0.10430  0.06833
C  -3.14343 -0.55335  1.76296
C  -2.48229 -1.87095  2.23016
O  -2.45725 -1.59439 -0.78378
C   3.11684 -0.51100  1.81780
C   2.43917 -1.77905  2.38625
C  -3.52081  0.93181 -1.03721
```

```
C   -2.76840  1.23287 -2.35253
C   -4.80945  0.16546 -1.41680
C   -3.86415  2.24804 -0.30794
C   -2.81417  0.57650  2.76362
C   -4.66741 -0.77744  1.70183
C    4.63726 -0.76092  1.76837
C    2.80984  0.69648  2.73044
C    3.92964  2.08253 -0.53509
C    4.77118 -0.14472 -1.42515
B    0.24180  3.00348  0.92588
N   -0.13132  3.50940 -0.55876
H    2.10576 -4.10158 -1.69296
H   -2.24815 -3.97426 -1.84087
H   -0.08651 -5.18732 -2.29199
H    0.02541  1.01834 -1.39072
H   -0.20312  1.80287  1.10525
H   -0.02222 -0.39672  1.64736
H    0.15602  2.71167 -1.17457
H    1.43469  3.11714  1.08174
H   -1.15497  3.54472 -0.65777
C    0.46049  4.81959 -0.94480
H   -0.42584  3.62392  1.73390
H   -3.19866  0.29778  3.76260
H   -1.72656  0.73305  2.84374
H   -3.28397  1.53591  2.48660
H   -5.00817 -1.13809  2.69055
H   -5.22694  0.14813  1.48893
H   -4.94452 -1.54229  0.95857
H   -2.84357 -2.10365  3.24916
H   -2.74685 -2.71192  1.56956
H   -1.38480 -1.78337  2.26295
H   -4.38922  2.93414 -0.99841
H   -4.52528  2.08474  0.55919
H   -2.96326  2.76774  0.06764
H   -5.33962  0.73417 -2.20372
H   -4.57204 -0.83046 -1.82218
H   -5.50544  0.04485 -0.57612
H   -3.41501  1.85268 -3.00169
H   -1.81483  1.75483 -2.18293
H   -2.52766  0.30351 -2.89444
H    3.19966  0.48994  3.74488
H    3.28544  1.62582  2.37394
H    1.72522  0.87352  2.80414
H    2.80813 -1.94288  3.41589
H    1.34371 -1.66938  2.42407
H    2.68097 -2.67010  1.78537
H    4.97906 -1.03597  2.78393
H    4.89948 -1.59299  1.09538
H    5.20625  0.13428  1.46938
H    4.52794  2.63517 -1.28389
H    3.05069  2.69722 -0.27699
H    4.54918  1.98586  0.37238
H    5.30833  0.31680 -2.27497
H    5.47716 -0.20003 -0.58579
H    4.49084 -1.16831 -1.71824
H    3.43027  1.42069 -3.16359
H    2.45909 -0.05415 -2.88327
H    1.84691  1.50967 -2.31771
H    0.14773  5.57997 -0.21361
H    1.55508  4.72931 -0.91138
H    0.14115  5.11782 -1.95588
```

TS(5-3)
SCF(BP86/BS1)= -1253.74934804
G(298 K) = -1253.154386
SCF(PBE0) = -1922.10875649
Lowest Frequencies = -413.5633cm-1, 21.5952cm-1

```
79
TS(5-3)
Ir -0.00000  0.28006 -0.00015
P   2.30385 -0.16010  0.02926
P  -2.30386 -0.16006  0.02926
O  -2.41856 -1.84535 -0.32099
O   2.41852 -1.84539 -0.32098
C  -0.00002 -1.76645 -0.34528
C   3.14811 -0.09814  1.77578
C   1.22103 -3.89294 -0.66613
H   2.17909 -4.41499 -0.74255
C   1.19481 -2.50509 -0.44553
C  -1.19486 -2.50507 -0.44554
C  -3.14812 -0.09809  1.77577
C  -3.51367  0.46142 -1.35070
C  -1.22110 -3.89292 -0.66613
H  -2.17918 -4.41495 -0.74256
C   3.51367  0.46135 -1.35069
C   4.66921 -0.35095  1.76954
H   4.92239 -1.31712  1.30446
H   5.02463 -0.38442  2.81674
H   5.23410  0.44751  1.26207
C  -0.00005 -4.57697 -0.78033
H  -0.00006 -5.65789 -0.95433
C  -4.66922 -0.35087  1.76953
H  -5.23410  0.44760  1.26207
H  -5.02464 -0.38434  2.81673
H  -4.92243 -1.31703  1.30445
C   2.84219  1.28646  2.38955
H   3.30562  2.10906  1.81742
H   3.24816  1.32755  3.41775
H   1.75499  1.46177  2.43856
C  -4.74534 -0.45925 -1.51625
H  -4.44605 -1.51156 -1.63810
H  -5.28643 -0.15066 -2.43029
H  -5.45367 -0.39129 -0.67917
C   2.48301 -1.20174  2.62936
H   1.38731 -1.09060  2.65538
H   2.86527 -1.12698  3.66454
H   2.72089 -2.20600  2.24444
C   4.74532 -0.45935 -1.51625
H   5.45366 -0.39140 -0.67916
H   5.28641 -0.15077 -2.43029
H   4.44601 -1.51165 -1.63810
C  -2.71560  0.41916 -2.67479
H  -1.85129  1.09788 -2.66110
H  -3.38854  0.72142 -3.49900
H  -2.34924 -0.59796 -2.89218
C  -2.84218  1.28651  2.38955
H  -1.75498  1.46179  2.43856
H  -3.24815  1.32760  3.41775
H  -3.30560  2.10911  1.81743
C  -2.48304 -1.20171  2.62935
```

H -2.72094 -2.20595 2.24442
H -2.86530 -1.12694 3.66453
H -1.38734 -1.09058 2.65537
C -3.95261 1.91085 -1.05098
H -4.58605 1.98092 -0.15058
H -4.55021 2.28991 -1.90120
H -3.08622 2.58134 -0.92891
C 3.95265 1.91077 -1.05098
H 3.08628 2.58129 -0.92891
H 4.55025 2.28981 -1.90120
H 4.58609 1.98083 -0.15058
C 2.71560 0.41911 -2.67478
H 2.34922 -0.59801 -2.89218
H 3.38855 0.72135 -3.49900
H 1.85131 1.09785 -2.66110
H -0.00001 -0.14763 1.58585
H 0.00000 1.95544 0.44659
B 0.00003 2.38598 -0.86845
H -1.03119 2.79388 -1.34456
H 1.03126 2.79384 -1.34453
H 0.00000 0.79469 -1.62537
N 0.00002 4.24003 0.41408
H 0.82437 4.22934 1.02351
H -0.82451 4.22943 1.02326
C 0.00021 5.42774 -0.45572
H -0.88736 5.38813 -1.10680
H 0.00017 6.39210 0.08909
H 0.88799 5.38805 -1.10652

3
SCF(BP86/BS1)= -1157.90400780
G(298 K) = -1157.365166
SCF(PBE0) = -1826.33592485
Lowest Frequencies = 18.6119cm-1, 28.1052cm-1

72
3
Ir -0.00000 -0.54239 -0.05710
P -2.31481 -0.12611 0.02313
P 2.31481 -0.12612 0.02312
O 2.42130 1.59186 -0.10609
O -2.42130 1.59186 -0.10604
C -0.00000 1.51530 -0.18054
C -3.14150 -0.43653 1.74216
C -1.22206 3.66171 -0.25817
H -2.17916 4.19050 -0.27318
C -1.19829 2.25862 -0.18478
C 1.19829 2.25862 -0.18480
C 3.14147 -0.43650 1.74217
C 3.49845 -0.56748 -1.43875
C 1.22207 3.66171 -0.25818
H 2.17916 4.19049 -0.27321
C -3.49842 -0.56745 -1.43878
C -4.66203 -0.17937 1.77435
H -4.91405 0.84560 1.45772
H -5.01716 -0.30239 2.81459
H -5.22633 -0.89319 1.15359
C 0.00000 4.35216 -0.30192
H 0.00000 5.44520 -0.36209
C 4.66201 -0.17933 1.77437
H 5.22630 -0.89311 1.15355
H 5.01714 -0.30242 2.81460
H 4.91401 0.84566 1.45782
C -2.83791 -1.89921 2.13974
H -3.30688 -2.62717 1.45694
H -3.23711 -2.08854 3.15355
H -1.75342 -2.09526 2.15310
C 4.73313 0.36203 -1.49065
H 4.43982 1.42302 -1.48960
H 5.27575 0.15870 -2.43248
H 5.43744 0.19243 -0.66460
C -2.46991 0.52793 2.74639
H -1.37880 0.38356 2.79319
H -2.88189 0.33057 3.75322
H -2.66752 1.58137 2.49229
C -4.73306 0.36211 -1.49074
H -5.43740 0.19254 -0.66471
H -5.27566 0.15879 -2.43258
H -4.43971 1.42309 -1.48969
C 2.67925 -0.34167 -2.73181
H 1.83413 -1.03932 -2.81403
H 3.34490 -0.50248 -3.59989
H 2.29024 0.68878 -2.79272
C 2.83788 -1.89917 2.13978
H 1.75339 -2.09523 2.15311
H 3.23705 -2.08848 3.15361
H 3.30687 -2.62715 1.45700
C 2.46987 0.52798 2.74638
H 2.66749 1.58142 2.49227
H 2.88182 0.33063 3.75322
H 1.37876 0.38361 2.79316
C 3.92651 -2.04730 -1.34160
H 4.58488 -2.23810 -0.47764
H 4.49631 -2.31478 -2.25087
H 3.05749 -2.72293 -1.28279
C -3.92654 -2.04725 -1.34163
H -3.05755 -2.72292 -1.28279
H -4.49632 -2.31471 -2.25092
H -4.58494 -2.23803 -0.47769
C -2.67915 -0.34169 -2.73180
H -2.29013 0.68875 -2.79273
H -3.34474 -0.50254 -3.59991
H -1.83401 -1.03934 -2.81394
H -0.00000 -0.14501 1.47261
H 0.00001 -2.32844 0.14508
B -0.00002 -2.51501 -1.19818
H 1.01656 -3.10458 -1.49154
H -1.01661 -3.10457 -1.49151
H -0.00001 -1.32247 -1.76980

**(iii) Figure 8(b)/Figure S79**

1
SCF(BP86/BS1)= -1131.24545551
G(298 K) = -1130.741666
SCF(PBE0) = -1799.69208534
Lowest Frequencies = 22.9797cm-1, 29.7745cm-1

```
68
1
C  -0.00000  4.30331 -0.16264
C  -1.23059  3.62410 -0.13426
C  -1.21181  2.21893 -0.07368
C  -0.00000  1.48500 -0.04040
C   1.21180  2.21893 -0.07367
C   1.23058  3.62410 -0.13425
Ir -0.00000 -0.58836  0.04900
P   2.26933 -0.19876  0.01103
C   3.28558 -0.51060  1.61603
O   2.42482  1.54919 -0.04845
O  -2.42482  1.54918 -0.04847
P  -2.26933 -0.19877  0.01103
C  -3.28557 -0.51058  1.61604
C  -3.21686 -0.64591 -1.60195
C   3.21685 -0.64589 -1.60196
H   0.00001 -1.98355  0.86738
H  -0.00001 -1.96843 -0.76634
H  -2.17970  4.16642 -0.15930
H   2.17969  4.16642 -0.15928
H  -0.00001  5.39789 -0.20984
C  -3.13990 -2.17493 -1.80974
C  -4.68126 -0.16720 -1.62156
C  -2.42981  0.05718 -2.73388
C  -4.56295  0.34793  1.71803
C  -3.61938 -2.01387  1.71673
C  -2.32650 -0.11811  2.76626
C   4.68125 -0.16720 -1.62156
C   3.13987 -2.17491 -1.80977
C   2.42981  0.05722 -2.73388
C   3.61942 -2.01388  1.71669
C   4.56295  0.34793  1.71803
C   2.32652 -0.11815  2.76627
H  -5.09222 -0.31004 -2.63867
H  -5.31764 -0.74458 -0.93114
H  -4.76666  0.90282 -1.37232
H  -2.86901 -0.23089 -3.70724
H  -2.48053  1.15399 -2.64371
H  -1.36759 -0.24061 -2.72534
H  -3.56981 -2.42462 -2.79767
H  -2.09649 -2.52997 -1.78892
H  -3.71032 -2.73498 -1.05100
H  -4.98880  0.23069  2.73219
H  -4.34202  1.41529  1.56222
H  -5.33871  0.04438  0.99942
H  -4.03057 -2.22716  2.72091
H  -4.38017 -2.32186  0.97987
H  -2.72283 -2.64275  1.58076
H  -2.82791 -0.31057  3.73362
H  -1.39070 -0.70058  2.72766
H  -2.06318  0.95188  2.72620
H   2.86900 -0.23085 -3.70724
H   1.36758 -0.24056 -2.72534
H   2.48054  1.15403 -2.64370
H   5.09221 -0.31004 -2.63868
H   4.76666  0.90282 -1.37232
H   5.31763 -0.74459 -0.93115
H   3.56978 -2.42459 -2.79770
H   3.71029 -2.73497 -1.05103
H   2.09646 -2.52994 -1.78895
H   4.03061 -2.22718  2.72086
H   2.72288 -2.64277  1.58071
H   4.38021 -2.32185  0.97981
H   2.82794 -0.31063  3.73361
H   2.06318  0.95183  2.72623
H   1.39073 -0.70064  2.72766
H   4.98881  0.23068  2.73218
H   5.33871  0.04442  0.99940
H   4.34200  1.41530  1.56225
```

TS(1-5)
SCF(BP86/BS1)= -1253.75515638
G(298 K) = -1253.159329
SCF(PBE0) = -1922.11187659
Lowest Frequencies = -133.0998cm-1, 23.5797cm-1

```
79
TS(1-5)
C   1.17541 -3.96988 -0.37562
C  -0.05719 -4.64387 -0.41439
C  -1.27141 -3.93808 -0.36504
C  -1.23619 -2.53616 -0.28301
C  -0.02068 -1.81873 -0.23132
C   1.17510 -2.56803 -0.29100
O  -2.44347 -1.84383 -0.25110
P  -2.29979 -0.13148  0.00482
C  -3.15633 -0.00721  1.72964
C  -2.31145 -0.91266  2.65694
Ir  0.00156  0.20641 -0.02532
P   2.28271 -0.19237 -0.00866
C   3.36464  0.42706 -1.47793
C   2.72430 -0.16529 -2.75535
O   2.40182 -1.90484 -0.25526
C  -3.43646  0.47758 -1.42920
C  -2.54940  0.49349 -2.69615
C   3.19547 -0.09427  1.69213
C   2.16279 -0.61284  2.72150
C   4.45240 -0.98677  1.76868
C   3.53858  1.37454  2.01071
C   3.27732  1.96791 -1.53959
C   4.83476 -0.02902 -1.39405
C  -4.62883 -0.47401 -1.67009
C  -3.92933  1.91046 -1.13722
C  -3.07319  1.44705  2.24022
C  -4.61593 -0.50231  1.72707
B  -0.19942  3.68425 -1.29312
N   0.44315  3.93428  0.18140
H  -2.23476 -4.45529 -0.39155
H   2.12492 -4.51168 -0.40946
H  -0.07161 -5.73665 -0.47642
H   0.01643  1.47467  1.15583
```

H -0.06492 2.46493 -1.48019
H -0.01967 -0.12569 -1.56868
H 0.15195 3.11645 0.76580
H -1.37774 4.00039 -1.21733
H 1.46496 3.84350 0.10679
C 0.10162 5.22971 0.82373
H 0.43711 4.35722 -2.08997
H 3.83500 2.32521 -2.42519
H 2.23289 2.30761 -1.64475
H 3.73135 2.44841 -0.65551
H 5.34137 0.22584 -2.34367
H 5.38469 0.48179 -0.58695
H 4.92230 -1.11801 -1.24976
H 3.29239 0.19294 -3.63363
H 2.75192 -1.26648 -2.75152
H 1.67773 0.15873 -2.87276
H 3.90100 1.44774 3.05293
H 4.33447 1.77105 1.35776
H 2.64849 2.02256 1.92373
H 4.79275 -1.02951 2.82011
H 4.23239 -2.01476 1.44102
H 5.28958 -0.60108 1.16901
H 2.60932 -0.56447 3.73252
H 1.24404 -0.00274 2.71431
H 1.88307 -1.66080 2.52353
H -4.45854 2.29149 -2.03035
H -4.64353 1.94349 -0.29744
H -3.09395 2.60177 -0.93374
H -3.16913 0.81019 -3.55576
H -1.71034 1.19964 -2.59483
H -2.14539 -0.50770 -2.92322
H -5.16977 -0.13044 -2.57151
H -4.29269 -1.50745 -1.84464
H -5.34699 -0.47987 -0.83702
H -3.44657 1.48289 3.28071
H -2.03066 1.80697 2.23565
H -3.68475 2.14258 1.64356
H -4.97876 -0.55676 2.77071
H -5.28896 0.18226 1.18520
H -4.70463 -1.50932 1.28823
H -2.69690 -0.82622 3.68995
H -2.36592 -1.97091 2.35612
H -1.25077 -0.60788 2.65849
H 0.42432 6.04729 0.16197
H -0.99097 5.28157 0.93050
H 0.58129 5.33444 1.81143

5
SCF(BP86/BS1)= -1253.77410896
G(298 K) = -1253.175442
SCF(PBE0) = -1922.13681572
Lowest Frequencies = 22.0845cm-1, 29.3483cm-1

79
5
C -1.28296 -3.51906 -1.60078
C -0.07258 -4.18758 -1.84615
C 1.15484 -3.59051 -1.51682
C 1.15017 -2.30649 -0.94593
C -0.03497 -1.58360 -0.69587
C -1.24025 -2.23715 -1.02833
O 2.38038 -1.73579 -0.62234
P 2.28455 -0.16941 0.09869
C 3.52176 0.71303 -1.11457
C 2.76155 0.91127 -2.44470
Ir -0.01167 0.28320 0.14117
P -2.30883 -0.10430 0.06833
C -3.14343 -0.55335 1.76296
C -2.48229 -1.87095 2.23016
O -2.45725 -1.59439 -0.78378
C 3.11684 -0.51100 1.81780
C 2.43917 -1.77905 2.38625
C -3.52081 0.93181 -1.03721
C -2.76840 1.23287 -2.35253
C -4.80945 0.16546 -1.41680
C -3.86415 2.24804 -0.30794
C -2.81417 0.57650 2.76362
C -4.66741 -0.77744 1.70183
C 4.63726 -0.76092 1.76837
C 2.80984 0.69648 2.73044
C 3.92964 2.08253 -0.53509
C 4.77118 -0.14472 -1.42515
B 0.24180 3.00348 0.92588
N -0.13132 3.50940 -0.55876
H 2.10576 -4.10158 -1.69296
H -2.24815 -3.97426 -1.84087
H -0.08651 -5.18732 -2.29199
H 0.02541 1.01834 -1.39072
H -0.20312 1.80287 1.10525
H -0.02222 -0.39672 1.64736
H 0.15602 2.71167 -1.17457
H 1.43469 3.11714 1.08174
H -1.15497 3.54472 -0.65777
C 0.46049 4.81959 -0.94480
H -0.42584 3.62392 1.73390
H -3.19866 0.29778 3.76260
H -1.72656 0.73305 2.84374
H -3.28397 1.53591 2.48660
H -5.00817 -1.13809 2.69055
H -5.22694 0.14813 1.48893
H -4.94452 -1.54229 0.95857
H -2.84357 -2.10365 3.24916
H -2.74685 -2.71192 1.56956
H -1.38480 -1.78337 2.26295
H -4.38922 2.93414 -0.99841
H -4.52528 2.08474 0.55919
H -2.96326 2.76774 0.06764
H -5.33962 0.73417 -2.20372
H -4.57204 -0.83046 -1.82218
H -5.50544 0.04485 -0.57612
H -3.41501 1.85268 -3.00169
H -1.81483 1.75483 -2.18293
H -2.52766 0.30351 -2.89444
H 3.19966 0.48994 3.74488
H 3.28544 1.62582 2.37394
H 1.72522 0.87352 2.80414
H 2.80813 -1.94288 3.41589
H 1.34371 -1.66938 2.42407
H 2.68097 -2.67010 1.78537
H 4.97906 -1.03597 2.78393
H 4.89948 -1.59299 1.09538

```
H   5.20625  0.13428  1.46938
H   4.52794  2.63517 -1.28389
H   3.05069  2.69722 -0.27699
H   4.54918  1.98586  0.37238
H   5.30833  0.31680 -2.27497
H   5.47716 -0.20003 -0.58579
H   4.49084 -1.16831 -1.71824
H   3.43027  1.42069 -3.16359
H   2.45909 -0.05415 -2.88327
H   1.84691  1.50967 -2.31771
H   0.14773  5.57997 -0.21361
H   1.55508  4.72931 -0.91138
H   0.14115  5.11782 -1.95588
```

TS(5-2)A
SCF(BP86/BS1)= -1253.75156640
G(298 K) = -1253.157313
SCF(PBE0) = -1922.10950791
Lowest Frequencies = -444.5698cm-1, 25.9697cm-1

```
79
TS(5-2)A
C  -1.29229 -3.39585 -1.84570
C  -0.07887 -4.01926 -2.18141
C   1.15483 -3.43557 -1.84814
C   1.15190 -2.20076 -1.17644
C  -0.03698 -1.52705 -0.83735
C  -1.24758 -2.16191 -1.17394
O   2.38432 -1.63160 -0.84326
P   2.28782 -0.18625  0.10230
C   3.55377  0.87849 -0.89168
C   2.81601  1.37165 -2.15800
Ir -0.00496  0.29686  0.14484
P  -2.31399 -0.10045  0.08947
C  -3.08726 -0.64275  1.77789
C  -2.41166 -1.97833  2.16630
O  -2.46035 -1.55292 -0.83812
C   3.02693 -0.78179  1.78742
C   2.29203 -2.09114  2.15748
C  -3.53364  0.99407 -0.93246
C  -2.77983  1.40678 -2.21871
C  -4.80643  0.22916 -1.36241
C  -3.88865  2.25228 -0.11073
C  -2.74167  0.44419  2.82044
C  -4.61154 -0.87492  1.73730
C   4.53946 -1.08140  1.74629
C   2.72528  0.30552  2.84328
C   3.97016  2.09431 -0.03616
C   4.78436  0.06261 -1.34942
B   0.00421  3.13132  0.92364
N  -0.16956  3.53075 -0.55180
H   2.10254 -3.92100 -2.09774
H  -2.25579 -3.85007 -2.09360
H  -0.09501 -4.98034 -2.70549
H   0.03928  1.10538 -1.46518
H   0.03548  1.74886  1.12183
H  -0.02546 -0.54111  1.49703
H  -0.00747  1.88817 -1.04531
H   1.10041  3.41327  1.37247
H  -1.14262  3.78536 -0.74847
C   0.70285  4.60492 -1.05230
H  -0.93458  3.37918  1.66324
H  -3.09427  0.11332  3.81510
H  -1.65576  0.61959  2.87778
H  -3.22732  1.40905  2.59984
H  -4.93066 -1.27269  2.71870
H  -5.17900  0.05404  1.56723
H  -4.89733 -1.61471  0.97220
H  -2.77399 -2.27614  3.16749
H  -2.66148 -2.78167  1.45520
H  -1.31516 -1.88881  2.21448
H  -4.42823  2.96965 -0.75660
H  -4.54874  2.02145  0.74178
H  -2.99138  2.75992  0.28475
H  -5.37599  0.86685 -2.06380
H  -4.55291 -0.70689 -1.88390
H  -5.47258 -0.00859 -0.52232
H  -3.48091  1.95428 -2.87538
H  -1.92608  2.06782 -2.01085
H  -2.41241  0.52926 -2.77672
H   3.05751 -0.05442  3.83485
H   3.25452  1.25045  2.63804
H   1.64757  0.52712  2.89860
H   2.63795 -2.41699  3.15573
H   1.20037 -1.95424  2.20393
H   2.50847 -2.89609  1.43730
H   4.83946 -1.50342  2.72359
H   4.79152 -1.82555  0.97352
H   5.14840 -0.17764  1.58515
H   4.55331  2.79457 -0.66269
H   3.09475  2.64147  0.35546
H   4.61032  1.80981  0.81535
H   5.39062  0.69675 -2.02263
H   5.43291 -0.24539 -0.51821
H   4.48293 -0.83660 -1.90878
H   3.54218  1.89931 -2.80338
H   2.39399  0.53514 -2.74031
H   2.00584  2.07488 -1.91760
H   0.58175  5.56611 -0.50911
H   1.75693  4.30177 -0.94014
H   0.51783  4.79632 -2.12535
```

2
SCF(BP86/BS1)= -1132.43360729
G(298 K) = -1131.910731
SCF(PBE0) = -1800.88403119
Lowest Frequencies = 14.9412cm-1, 32.2674cm-1

```
70
2
Ir -0.00094 -0.62013 -0.07779
C   0.00539  1.49496 -0.17749
C   1.20697  2.21570 -0.26324
C  -1.18657  2.22973 -0.07863
C   1.23905  3.62148 -0.29742
C  -1.20654  3.63611 -0.11068
C   0.01852  4.31591 -0.22651
H   2.19278  4.15197 -0.37002
H  -2.15396  4.17800 -0.03962
H   0.02303  5.41067 -0.25145
```

```
P  -2.28842 -0.18075  0.03863
P   2.29265 -0.18691 -0.01325
C  -3.33734 -0.55189 -1.54903
C  -3.27858 -0.57675  1.64962
C   3.48114 -0.79946 -1.40858
C   3.12280 -0.31328  1.73124
O  -2.40179  1.55117  0.06957
O   2.42075  1.51947 -0.31156
H   0.02950 -0.40624  1.50866
H  -0.02817 -1.50977 -1.53706
H  -0.02534 -0.59180 -1.81686
H  -0.00534 -2.20603  0.41097
C  -2.74031  0.32062 -2.67860
H  -1.66865  0.12511 -2.84140
H  -3.27211  0.09098 -3.62034
H  -2.86200  1.39421 -2.46586
C  -3.18469 -2.04698 -1.90729
H  -3.68929 -2.24051 -2.87221
H  -2.12746 -2.33955 -2.00986
H  -3.64439 -2.70792 -1.15470
C  -4.83100 -0.19227 -1.40983
H  -5.31698 -0.31104 -2.39642
H  -5.36131 -0.85504 -0.70735
H  -4.97389  0.85317 -1.09242
C  -2.31633 -0.35097  2.83845
H  -2.87822 -0.51040  3.77750
H  -1.46820 -1.05164  2.81689
H  -1.91690  0.67688  2.85280
C  -3.70484 -2.06029  1.62435
H  -4.48563 -2.26188  0.87225
H  -2.84684 -2.72656  1.42892
H  -4.12258 -2.33342  2.61097
C  -4.49639  0.35548  1.84224
H  -5.29371  0.18317  1.10644
H  -4.92593  0.16753  2.84365
H  -4.19936  1.41447  1.79170
C   2.68229 -0.75993 -2.73202
H   3.36894 -0.99774 -3.56512
H   1.87123 -1.50355 -2.74590
H   2.25093  0.23678 -2.92399
C   4.71709  0.11373 -1.57709
H   5.40358  0.07258 -0.72065
H   5.28059 -0.22030 -2.46812
H   4.41964  1.16208 -1.73374
C   3.89642 -2.25727 -1.11581
H   4.56802 -2.33686 -0.24523
H   3.01914 -2.90357 -0.93921
H   4.44116 -2.66220 -1.98871
C   2.86320 -1.73174  2.28581
H   1.78802 -1.97111  2.28568
H   3.38637 -2.51142  1.70759
H   3.23514 -1.78426  3.32609
C   4.63416 -0.00538  1.73459
H   4.98800  0.00755  2.78242
H   5.22524 -0.76985  1.20492
H   4.85384  0.98272  1.29878
C   2.41927  0.73157  2.62909
H   1.32945  0.57717  2.66690
H   2.81532  0.63312  3.65673
H   2.61105  1.75946  2.28280
```

```
TS(2-1)
SCF(BP86/BS1)= -1132.41105268
G(298 K) = -1131.891623
SCF(PBE0) = -1800.85461114
Lowest Frequencies = -318.0418cm-1,
26.4506cm-1

70
TS(2-1)
Ir -0.00381 -0.60780 -0.21527
C   0.00238  1.48330 -0.14680
C   1.21012  2.21668 -0.18923
C  -1.19736  2.22604 -0.05748
C   1.23857  3.62213 -0.18822
C  -1.21397  3.63221 -0.05543
C   0.01442  4.31178 -0.12922
H   2.19165  4.15719 -0.22386
H  -2.16108  4.17504  0.01022
H   0.01875  5.40708 -0.13017
P  -2.27368 -0.18337  0.01724
P   2.27559 -0.19402 -0.02462
C  -3.47670 -0.55283 -1.45693
C  -3.08333 -0.58425  1.72413
C   3.59341 -0.70718 -1.34500
C   2.96844 -0.40337  1.77004
O  -2.41180  1.55626  0.05700
O   2.42445  1.53593 -0.21434
H   0.00209 -1.48624  1.07391
H  -0.10223 -0.96967 -2.31041
H  -0.13273 -0.65803 -3.02476
H  -0.00193 -2.22579 -0.56737
C  -3.06250  0.39890 -2.60373
H  -1.99552  0.31305 -2.85745
H  -3.64483  0.13772 -3.50678
H  -3.27235  1.44878 -2.34720
C  -3.25434 -2.02285 -1.87895
H  -3.84962 -2.23244 -2.78724
H  -2.19535 -2.22864 -2.09968
H  -3.57921 -2.73234 -1.09939
C  -4.96901 -0.31420 -1.15254
H  -5.54376 -0.45126 -2.08799
H  -5.37119 -1.03215 -0.41957
H  -5.16099  0.70958 -0.79334
C  -2.02248 -0.24686  2.79807
H  -2.46357 -0.41757  3.79793
H  -1.12664 -0.87942  2.70252
H  -1.70486  0.80736  2.74011
C  -3.41151 -2.09136  1.77979
H  -4.22843 -2.36814  1.09229
H  -2.52965 -2.70960  1.53899
H  -3.73954 -2.35542  2.80234
C  -4.33463  0.27416  2.01535
H  -5.19835  0.00293  1.39346
H  -4.62760  0.11930  3.07041
H  -4.12466  1.34617  1.87528
C   2.87208 -0.65673 -2.70950
H   3.60540 -0.87540 -3.50794
H   2.06635 -1.40275 -2.77068
H   2.44155  0.33860 -2.91120
C   4.80216  0.25274 -1.39908
H   5.42363  0.21538 -0.49350
```

```
H   5.44276 -0.04003 -2.25206
H   4.47963  1.29349 -1.55458
C   4.04698 -2.15743 -1.07446
H   4.67900 -2.23969 -0.17487
H   3.18795 -2.84205 -0.96505
H   4.64889 -2.51291 -1.93131
C   2.80840 -1.87659  2.20755
H   1.76400 -2.21622  2.11714
H   3.44101 -2.56420  1.62377
H   3.10854 -1.96941  3.26811
C   4.43751  0.03996  1.91936
H   4.70280  0.03228  2.99328
H   5.13835 -0.63999  1.40856
H   4.59547  1.06387  1.54391
C   2.08965  0.49658  2.67029
H   1.02164  0.24582  2.57677
H   2.39682  0.35217  3.72298
H   2.21190  1.56280  2.42105
```

TS(2-2')
SCF(BP86/BS1)= -1132.43291718
G(298 K) = -1131.911626
SCF(PBE0) = -1800.88199172
Lowest Frequencies = -491.7191cm-1, 15.0865cm-1

```
70
TS(2-2')
C  -1.22621  3.64010 -0.07248
C  -0.00000  4.32730 -0.08459
C   1.22621  3.64010 -0.07247
C   1.20067  2.23364 -0.06311
C   0.00000  1.50703 -0.09278
C  -1.20067  2.23364 -0.06313
O   2.41845  1.54818  0.00049
P   2.29520 -0.18247  0.03145
C   3.33463 -0.53625  1.62236
C   2.41190 -0.26141  2.83194
Ir  0.00000 -0.60623  0.00199
P  -2.29520 -0.18247  0.03145
C  -3.28860 -0.60690 -1.57719
C  -2.65932  0.23245 -2.71410
O  -2.41845  1.54818  0.00046
C   3.28860 -0.60687 -1.57719
C   2.65932  0.23249 -2.71409
C  -3.33463 -0.53622  1.62236
C  -2.41190 -0.26135  2.83194
C  -4.56641  0.38902  1.74544
C  -3.74505 -2.02432  1.63206
C  -3.12022 -2.11115 -1.88662
C  -4.78733 -0.25000 -1.49437
C   4.78733 -0.24998 -1.49437
C   3.12022 -2.11112 -1.88665
C   3.74507 -2.02433  1.63203
C   4.56641  0.38900  1.74545
H   2.17955  4.17593 -0.05848
H  -2.17956  4.17593 -0.05851
H  -0.00000  5.42232 -0.09103
H   0.00000 -0.29038  1.58272
H   0.00000 -1.64054 -1.30806
H   0.00000 -0.61361 -1.69791
H   0.00000 -2.13194  0.64191
H  -3.60772 -2.33639 -2.85340
H  -2.05936 -2.39758 -1.96524
H  -3.58803 -2.75308 -1.12260
H  -5.24097 -0.39719 -2.49241
H  -5.33752 -0.89639 -0.79188
H  -4.94421  0.80286 -1.20988
H  -3.16246 -0.02727 -3.66375
H  -2.78874  1.31173 -2.53772
H  -1.58293  0.03237 -2.83569
H  -4.19242 -2.26991  2.61292
H  -4.49820 -2.25923  0.86165
H  -2.87432 -2.68716  1.48742
H  -5.02067  0.23613  2.74187
H  -4.27903  1.44855  1.66098
H  -5.34210  0.17877  0.99651
H  -3.00037 -0.40046  3.75787
H  -1.55374 -0.94929  2.85701
H  -2.02571  0.77161  2.82730
H   3.60772 -2.33635 -2.85344
H   3.58802 -2.75306 -1.12263
H   2.05936 -2.39755 -1.96528
H   3.16245 -0.02722 -3.66375
H   1.58292  0.03241 -2.83568
H   2.78874  1.31177 -2.53770
H   5.24097 -0.39716 -2.49241
H   4.94420  0.80288 -1.20987
H   5.33751 -0.89637 -0.79189
H   4.19245 -2.26994  2.61287
H   2.87435 -2.68719  1.48738
H   4.49822 -2.25922  0.86161
H   5.02067  0.23610  2.74188
H   5.34210  0.17879  0.99652
H   4.27902  1.44854  1.66102
H   3.00038 -0.40054  3.75787
H   2.02571  0.77155  2.82733
H   1.55374 -0.94936  2.85700
```

2'
SCF(BP86/BS1)= -1132.43853014
G(298 K) = -1131.914424
SCF(PBE0) = -1800.88422704
Lowest Frequencies = 21.6226cm-1, 34.1667cm-1

```
70
2'
C   1.22680  3.63855 -0.14310
C   0.00001  4.32469 -0.15843
C  -1.22679  3.63856 -0.14310
C  -1.20324  2.23261 -0.10620
C   0.00001  1.51226 -0.07188
C   1.20326  2.23261 -0.10619
O  -2.41871  1.54350 -0.12274
P  -2.30001 -0.18410 -0.01443
C  -3.40010 -0.66045 -1.53013
C  -2.53566 -0.45696 -2.79543
Ir  0.00000 -0.59874 -0.04615
P   2.30002 -0.18410 -0.01443
C   3.22284 -0.48826  1.66139
C   2.58218  0.46481  2.69660
```

| | | | |
|---|---|---|---|
| O | 2.41872 | 1.54350 | -0.12273 |
| C | -3.22286 | -0.48825 | 1.66138 |
| C | -2.58220 | 0.46479 | 2.69661 |
| C | 3.40011 | -0.66045 | -1.53014 |
| C | 2.53561 | -0.45708 | -2.79542 |
| C | 4.65191 | 0.23661 | -1.66300 |
| C | 3.78532 | -2.15126 | -1.41636 |
| C | 2.98024 | -1.95261 | 2.08942 |
| C | 4.73569 | -0.19307 | 1.60611 |
| C | -4.73569 | -0.19303 | 1.60606 |
| C | -2.98030 | -1.95262 | 2.08937 |
| C | -3.78521 | -2.15129 | -1.41640 |
| C | -4.65198 | 0.23653 | -1.66289 |
| H | -2.17996 | 4.17417 | -0.16542 |
| H | 2.17998 | 4.17416 | -0.16541 |
| H | 0.00001 | 5.41930 | -0.18668 |
| H | 0.00001 | -0.25461 | -1.64880 |
| H | 0.00000 | -2.00487 | 0.78256 |
| H | 0.00001 | -0.32523 | 1.58220 |
| H | 0.00000 | -1.95199 | -0.95590 |
| H | 3.43258 | -2.11464 | 3.08546 |
| H | 1.90393 | -2.17628 | 2.15859 |
| H | 3.43893 | -2.67722 | 1.39626 |
| H | 5.14824 | -0.27942 | 2.62867 |
| H | 5.28477 | -0.91060 | 0.97549 |
| H | 4.94387 | 0.82874 | 1.25004 |
| H | 3.03202 | 0.26128 | 3.68587 |
| H | 2.76667 | 1.52079 | 2.44372 |
| H | 1.49434 | 0.31123 | 2.77715 |
| H | 4.27868 | -2.47073 | -2.35282 |
| H | 4.49213 | -2.34327 | -0.59207 |
| H | 2.89768 | -2.79171 | -1.27301 |
| H | 5.13953 | 0.01531 | -2.63041 |
| H | 4.38002 | 1.30370 | -1.65615 |
| H | 5.39603 | 0.06176 | -0.87412 |
| H | 3.15839 | -0.67828 | -3.68206 |
| H | 1.66034 | -1.12302 | -2.80804 |
| H | 2.17678 | 0.58195 | -2.88346 |
| H | -3.43267 | -2.11466 | 3.08540 |
| H | -3.43899 | -2.67721 | 1.39618 |
| H | -1.90400 | -2.17632 | 2.15857 |
| H | -3.03205 | 0.26124 | 3.68587 |
| H | -1.49435 | 0.31118 | 2.77718 |
| H | -2.76666 | 1.52077 | 2.44375 |
| H | -5.14828 | -0.27944 | 2.62860 |
| H | -4.94385 | 0.82881 | 1.25005 |
| H | -5.28477 | -0.91051 | 0.97536 |
| H | -4.27860 | -2.47075 | -2.35285 |
| H | -2.89752 | -2.79169 | -1.27312 |
| H | -4.49196 | -2.34338 | -0.59208 |
| H | -5.13964 | 0.01522 | -2.63028 |
| H | -5.39603 | 0.06160 | -0.87397 |
| H | -4.38016 | 1.30363 | -1.65603 |
| H | -3.15845 | -0.67817 | -3.68206 |
| H | -2.17692 | 0.58211 | -2.88344 |
| H | -1.66034 | -1.12284 | -2.80810 |

**(iv) Figure 8(b)/Figure S80(a)**

```
1
SCF(BP86/BS1)= -1131.24545551
G(298 K) = -1130.741666
SCF(PBE0) = -1799.69208534
Lowest Frequencies = 22.9797cm-1, 29.7745cm-1

68
1
C  -0.00000  4.30331 -0.16264
C  -1.23059  3.62410 -0.13426
C  -1.21181  2.21893 -0.07368
C  -0.00000  1.48500 -0.04040
C   1.21180  2.21893 -0.07367
C   1.23058  3.62410 -0.13425
Ir -0.00000 -0.58836  0.04900
P   2.26933 -0.19876  0.01103
C   3.28558 -0.51060  1.61603
O   2.42482  1.54919 -0.04845
O  -2.42482  1.54918 -0.04847
P  -2.26933 -0.19877  0.01103
C  -3.28557 -0.51058  1.61604
C  -3.21686 -0.64591 -1.60195
C   3.21685 -0.64589 -1.60196
H   0.00001 -1.98355  0.86738
H  -0.00001 -1.96843 -0.76634
H  -2.17970  4.16642 -0.15930
H   2.17969  4.16642 -0.15928
H  -0.00001  5.39789 -0.20984
C  -3.13990 -2.17493 -1.80974
C  -4.68126 -0.16720 -1.62156
C  -2.42981  0.05718 -2.73388
C  -4.56295  0.34793  1.71803
C  -3.61938 -2.01387  1.71673
C  -2.32650 -0.11811  2.76626
C   4.68125 -0.16720 -1.62156
C   3.13987 -2.17491 -1.80977
C   2.42981  0.05722 -2.73388
C   3.61942 -2.01388  1.71669
C   4.56295  0.34793  1.71803
C   2.32652 -0.11815  2.76627
H  -5.09222 -0.31004 -2.63867
H  -5.31764 -0.74458 -0.93114
H  -4.76666  0.90282 -1.37232
H  -2.86901 -0.23089 -3.70724
H  -2.48053  1.15399 -2.64371
H  -1.36759 -0.24061 -2.72534
H  -3.56981 -2.42462 -2.79767
H  -2.09649 -2.52997 -1.78892
H  -3.71032 -2.73498 -1.05100
H  -4.98880  0.23069  2.73219
H  -4.34202  1.41529  1.56222
H  -5.33871  0.04438  0.99942
H  -4.03057 -2.22716  2.72091
H  -4.38017 -2.32186  0.97987
H  -2.72283 -2.64275  1.58076
H  -2.82791 -0.31057  3.73362
H  -1.39070 -0.70058  2.72766
H  -2.06318  0.95188  2.72620
H   2.86900 -0.23085 -3.70724
H   1.36758 -0.24056 -2.72534
H   2.48054  1.15403 -2.64370
H   5.09221 -0.31004 -2.63868
H   4.76666  0.90282 -1.37232
H   5.31763 -0.74459 -0.93115
H   3.56978 -2.42459 -2.79770
H   3.71029 -2.73497 -1.05103
H   2.09646 -2.52994 -1.78895
H   4.03061 -2.22718  2.72086
H   2.72288 -2.64277  1.58071
H   4.38021 -2.32185  0.97981
H   2.82794 -0.31063  3.73361
H   2.06318  0.95183  2.72623
H   1.39073 -0.70064  2.72766
H   4.98881  0.23068  2.73218
H   5.33871  0.04442  0.99940
H   4.34200  1.41530  1.56225

TS(1-5)
SCF(BP86/BS1)= -1253.75515638
G(298 K) = -1253.159329
SCF(PBE0) = -1922.11187659
Lowest Frequencies = -133.0998cm-1, 23.5797cm-1

79
TS(1-5)
C   1.17541 -3.96988 -0.37562
C  -0.05719 -4.64387 -0.41439
C  -1.27141 -3.93808 -0.36504
C  -1.23619 -2.53616 -0.28301
C  -0.02068 -1.81873 -0.23132
C   1.17510 -2.56803 -0.29100
O  -2.44347 -1.84383 -0.25110
P  -2.29979 -0.13148  0.00482
C  -3.15633 -0.00721  1.72964
C  -2.31145 -0.91266  2.65694
Ir  0.00156  0.20641 -0.02532
P   2.28271 -0.19237 -0.00866
C   3.36464  0.42706 -1.47793
C   2.72430 -0.16529 -2.75535
O   2.40182 -1.90484 -0.25526
C  -3.43646  0.47758 -1.42920
C  -2.54940  0.49349 -2.69615
C   3.19547 -0.09427  1.69213
C   2.16279 -0.61284  2.72150
C   4.45240 -0.98677  1.76868
C   3.53858  1.37454  2.01071
C   3.27732  1.96791 -1.53959
C   4.83476 -0.02902 -1.39405
C  -4.62883 -0.47401 -1.67009
C  -3.92933  1.91046 -1.13722
C  -3.07319  1.44705  2.24022
C  -4.61593 -0.50231  1.72707
B  -0.19942  3.68425 -1.29312
N   0.44315  3.93428  0.18140
H  -2.23476 -4.45529 -0.39155
H   2.12492 -4.51168 -0.40946
H  -0.07161 -5.73665 -0.47642
```

```
H   0.01643  1.47467  1.15583
H  -0.06492  2.46493 -1.48019
H  -0.01967 -0.12569 -1.56868
H   0.15195  3.11645  0.76580
H  -1.37774  4.00039 -1.21733
H   1.46496  3.84350  0.10679
C   0.10162  5.22971  0.82373
H   0.43711  4.35722 -2.08997
H   3.83500  2.32521 -2.42519
H   2.23289  2.30761 -1.64475
H   3.73135  2.44841 -0.65551
H   5.34137  0.22584 -2.34367
H   5.38469  0.48179 -0.58695
H   4.92230 -1.11801 -1.24976
H   3.29239  0.19294 -3.63363
H   2.75192 -1.26648 -2.75152
H   1.67773  0.15873 -2.87276
H   3.90100  1.44774  3.05293
H   4.33447  1.77105  1.35776
H   2.64849  2.02256  1.92373
H   4.79275 -1.02951  2.82011
H   4.23239 -2.01476  1.44102
H   5.28958 -0.60108  1.16901
H   2.60932 -0.56447  3.73252
H   1.24404 -0.00274  2.71431
H   1.88307 -1.66080  2.52353
H  -4.45854  2.29149 -2.03035
H  -4.64353  1.94349 -0.29744
H  -3.09395  2.60177 -0.93374
H  -3.16913  0.81019 -3.55576
H  -1.71034  1.19964 -2.59483
H  -2.14539 -0.50770 -2.92322
H  -5.16977 -0.13044 -2.57151
H  -4.29269 -1.50745 -1.84464
H  -5.34699 -0.47987 -0.83702
H  -3.44657  1.48289  3.28071
H  -2.03066  1.80697  2.23565
H  -3.68475  2.14258  1.64356
H  -4.97876 -0.55676  2.77071
H  -5.28896  0.18226  1.18520
H  -4.70463 -1.50932  1.28823
H  -2.69690 -0.82622  3.68995
H  -2.36592 -1.97091  2.35612
H  -1.25077 -0.60788  2.65849
H   0.42432  6.04729  0.16197
H  -0.99097  5.28157  0.93050
H   0.58129  5.33444  1.81143
```

5
SCF(BP86/BS1)= -1253.77410896
G(298 K) = -1253.175442
SCF(PBE0) = -1922.13681572
Lowest Frequencies = 22.0845cm-1, 29.3483cm-1

```
79
5
C  -1.28296 -3.51906 -1.60078
C  -0.07258 -4.18758 -1.84615
C   1.15484 -3.59051 -1.51682
C   1.15017 -2.30649 -0.94593
C  -0.03497 -1.58360 -0.69587
C  -1.24025 -2.23715 -1.02833
O   2.38038 -1.73579 -0.62234
P   2.28455 -0.16941  0.09869
C   3.52176  0.71303 -1.11457
C   2.76155  0.91127 -2.44470
Ir -0.01167  0.28320  0.14117
P  -2.30883 -0.10430  0.06833
C  -3.14343 -0.55335  1.76296
C  -2.48229 -1.87095  2.23016
O  -2.45725 -1.59439 -0.78378
C   3.11684 -0.51100  1.81780
C   2.43917 -1.77905  2.38625
C  -3.52081  0.93181 -1.03721
C  -2.76840  1.23287 -2.35253
C  -4.80945  0.16546 -1.41680
C  -3.86415  2.24804 -0.30794
C  -2.81417  0.57650  2.76362
C  -4.66741 -0.77744  1.70183
C   4.63726 -0.76092  1.76837
C   2.80984  0.69648  2.73044
C   3.92964  2.08253 -0.53509
C   4.77118 -0.14472 -1.42515
B   0.24180  3.00348  0.92588
N  -0.13132  3.50940 -0.55876
H   2.10576 -4.10158 -1.69296
H  -2.24815 -3.97426 -1.84087
H  -0.08651 -5.18732 -2.29199
H   0.02541  1.01834 -1.39072
H  -0.20312  1.80287  1.10525
H  -0.02222 -0.39672  1.64736
H   0.15602  2.71167 -1.17457
H   1.43469  3.11714  1.08174
H  -1.15497  3.54472 -0.65777
C   0.46049  4.81959 -0.94480
H  -0.42584  3.62392  1.73390
H  -3.19866  0.29778  3.76260
H  -1.72656  0.73305  2.84374
H  -3.28397  1.53591  2.48660
H  -5.00817 -1.13809  2.69055
H  -5.22694  0.14813  1.48893
H  -4.94452 -1.54229  0.95857
H  -2.84357 -2.10365  3.24916
H  -2.74685 -2.71192  1.56956
H  -1.38480 -1.78337  2.26295
H  -4.38922  2.93414 -0.99841
H  -4.52528  2.08474  0.55919
H  -2.96326  2.76774  0.06764
H  -5.33962  0.73417 -2.20372
H  -4.57204 -0.83046 -1.82218
H  -5.50544  0.04485 -0.57612
H  -3.41501  1.85268 -3.00169
H  -1.81483  1.75483 -2.18293
H  -2.52766  0.30351 -2.89444
H   3.19966  0.48994  3.74488
H   3.28544  1.62582  2.37394
H   1.72522  0.87352  2.80414
H   2.80813 -1.94288  3.41589
H   1.34371 -1.66938  2.42407
H   2.68097 -2.67010  1.78537
H   4.97906 -1.03597  2.78393
```

H   4.89948 -1.59299  1.09538
H   5.20625  0.13428  1.46938
H   4.52794  2.63517 -1.28389
H   3.05069  2.69722 -0.27699
H   4.54918  1.98586  0.37238
H   5.30833  0.31680 -2.27497
H   5.47716 -0.20003 -0.58579
H   4.49084 -1.16831 -1.71824
H   3.43027  1.42069 -3.16359
H   2.45909 -0.05415 -2.88327
H   1.84691  1.50967 -2.31771
H   0.14773  5.57997 -0.21361
H   1.55508  4.72931 -0.91138
H   0.14115  5.11782 -1.95588

5.NH2Me
SCF(BP86/BS1)= -1349.64106339
G(298 K) = -1348.987773
SCF(PBE0) = -2017.92920513
Lowest Frequencies = 10.2053cm-1, 15.1607cm-1

86
5.NH2Me
Ir -0.06964 -0.05749  0.06437
P   1.80926 -1.42470  0.07332
P  -2.31685  0.57809  0.03192
O  -3.14797 -0.87980 -0.37863
O   1.17952 -3.02363 -0.06952
C  -0.94651 -1.87719 -0.24601
C  -2.96969 -3.25898 -0.59611
H  -4.05591 -3.30255 -0.71857
C  -0.20378 -3.07725 -0.25405
C  -2.89400  3.17703 -1.02609
H  -3.47709  3.49538 -0.14574
H  -3.23092  3.79176 -1.88239
H  -1.83312  3.40973 -0.83599
C  -2.18033 -4.42017 -0.61489
H  -2.65393 -5.39636 -0.76101
C  -2.34156 -2.01630 -0.40790
C  -0.78925 -4.34061 -0.43956
H  -0.15889 -5.23468 -0.44223
C   2.78242 -1.57680  1.74747
C  -3.12251  0.96926  1.75504
C  -3.10041  1.68538 -1.36017
C   4.06850 -2.42399  1.69705
H   3.88308 -3.44027  1.31467
H   4.46459 -2.52350  2.72546
H   4.86118 -1.95470  1.09111
C   3.00996 -1.52558 -1.45663
C  -2.28541  2.08291  2.42074
H  -1.22390  1.79410  2.48483
H  -2.66243  2.25449  3.44685
H  -2.34991  3.04084  1.87735
C   2.10184 -1.53191 -2.70710
H   1.48616 -2.44567 -2.74419
H   2.73931 -1.51216 -3.61129
H   1.41702 -0.66922 -2.72219
C   3.85581 -2.81936 -1.48990
H   4.68776 -2.81369 -0.77238
H   4.29457 -2.92639 -2.49978
H   3.23088 -3.70641 -1.30068
C   1.80669 -2.22723  2.75468
H   0.85541 -1.67322  2.80453
H   2.27402 -2.21616  3.75733
H   1.58858 -3.27241  2.48405
C   3.11997 -0.14280  2.21169
H   3.83119  0.35943  1.53049
H   3.59963 -0.18598  3.20770
H   2.21235  0.47638  2.29043
C  -4.59959  1.37990 -1.58635
H  -4.77040  0.30284 -1.73583
H  -4.92808  1.90844 -2.50123
H  -5.23989  1.72427 -0.76283
C   3.91950 -0.28221 -1.48225
H   3.31779  0.64231 -1.53886
H   4.56212 -0.30589 -2.38240
H   4.58401 -0.22275 -0.60271
C  -2.35281  1.34179 -2.66705
H  -1.27561  1.54872 -2.58971
H  -2.78190  1.94468 -3.48986
H  -2.46475  0.27628 -2.92746
C  -3.00832 -0.32931  2.58652
H  -3.64082 -1.13091  2.17278
H  -3.34696 -0.11807  3.61825
H  -1.96767 -0.68913  2.62757
C  -4.60814  1.37707  1.70284
H  -4.76827  2.35041  1.21090
H  -4.98259  1.47410  2.73963
H  -5.22656  0.61656  1.19911
H   0.81994  1.41125  0.61345
H   0.07677  0.26259 -1.57399
H  -0.18447 -0.28683  1.71364
B   0.92925  2.68471  0.52049
H  -0.08279  3.29937  0.27827
H   1.43335  2.95868  1.59862
N   2.01643  3.01072 -0.61166
H   2.40199  3.97629 -0.37646
H   2.79628  2.34547 -0.51838
N   2.79781  5.60187  0.34921
H   2.16825  6.31816 -0.02980
C   4.18051  6.12259  0.39763
H   4.29999  7.05016  0.99119
H   4.52943  6.32517 -0.62792
H   4.84172  5.35435  0.82981
H   2.45905  5.41286  1.30027
C   1.52432  2.98270 -2.01836
H   2.34535  3.17825 -2.72764
H   1.06952  1.99534 -2.20318
H   0.75032  3.75643 -2.12581

TS(5-2)B
SCF(BP86/BS1)= -1349.61482596
G(298 K) = -1348.964752
SCF(PBE0) = -2017.90593542
Lowest Frequencies = -39.7767cm-1, 13.9132cm-1

86
TS(5-2)B
Ir  0.01072  0.08004  0.06673
P   2.30468 -0.25794  0.09682

```
P  -2.22284 -0.53486  0.09177
O  -2.26832 -2.17071 -0.48531
O   2.55358 -1.83545 -0.58097
C   0.13710 -1.93029 -0.55719
C  -0.95164 -4.07301 -1.13921
H  -1.87313 -4.64951 -1.26463
C   1.37489 -2.56249 -0.77953
C  -3.93814  1.58082 -0.74287
H  -4.58222  1.56814  0.15266
H  -4.50796  2.07866 -1.55076
H  -3.06065  2.20643 -0.49526
C   0.30951 -4.64718 -1.37391
H   0.37573 -5.69265 -1.69340
C  -1.00873 -2.72874 -0.72980
C   1.48741 -3.90274 -1.19028
H   2.47401 -4.34643 -1.35435
C   3.10392 -0.50669  1.85163
C  -3.03185 -0.75857  1.84410
C  -3.52543  0.15989 -1.17846
C   4.63230 -0.70464  1.85270
H   4.94820 -1.51566  1.17711
H   4.95009 -0.98107  2.87590
H   5.17644  0.21640  1.58653
C   3.51507  0.67983 -1.09760
C  -2.82103  0.55534  2.62733
H  -1.75897  0.84748  2.63467
H  -3.15990  0.41549  3.67132
H  -3.40388  1.39119  2.20160
C   2.83133  0.68307 -2.48288
H   2.74243 -0.34085 -2.88156
H   3.44711  1.27474 -3.18706
H   1.81583  1.10402 -2.44292
C   4.88824 -0.00714 -1.27714
H   5.54811  0.11020 -0.40727
H   5.40312  0.45180 -2.14259
H   4.76937 -1.08171 -1.48817
C   2.45404 -1.78262  2.43605
H   1.35493 -1.71418  2.43399
H   2.79372 -1.90376  3.48178
H   2.74730 -2.68093  1.86996
C   2.73176  0.71143  2.72259
H   3.21404  1.63945  2.36965
H   3.07398  0.53451  3.75995
H   1.64284  0.87566  2.73231
C  -4.77250 -0.73524 -1.36285
H  -4.48218 -1.78544 -1.52254
H  -5.32527 -0.39622 -2.25977
H  -5.46841 -0.68810 -0.51463
C   3.68298  2.12473 -0.58498
H   2.70507  2.60309 -0.39838
H   4.23117  2.72846 -1.33351
H   4.25897  2.16687  0.35524
C  -2.80305  0.20094 -2.54438
H  -1.85046  0.75031 -2.50446
H  -3.46886  0.66901 -3.29447
H  -2.56169 -0.81786 -2.88913
C  -2.25615 -1.90589  2.53169
H  -2.41788 -2.86631  2.01691
H  -2.61712 -2.00278  3.57279
H  -1.17408 -1.70291  2.55663
C  -4.52736 -1.13149  1.83584
H  -5.16745 -0.30780  1.47871
H  -4.84037 -1.35369  2.87361
H  -4.72871 -2.02961  1.23014
H  -0.06018  1.64688  0.71383
H   0.01882  0.59031 -1.57058
H   0.02785 -0.36850  1.63579
B   0.12840  3.68760  1.79537
H  -0.81927  3.30070  2.42632
H   1.25843  3.35343  2.02271
N  -0.07346  4.74997  0.86168
H  -0.25061  3.49895 -0.74380
N  -0.48162  2.98434 -1.62565
H  -0.20899  1.85873 -1.38591
C   0.19200  3.54043 -2.82622
H  -0.03892  2.90504 -3.69395
H   1.27807  3.52302 -2.65903
H  -0.13341  4.57288 -3.02991
H  -1.50528  2.99136 -1.72533
C  -1.31569  5.50775  0.68397
H  -1.58618  5.63049 -0.38347
H  -2.13176  4.96863  1.18840
H  -1.24741  6.51849  1.12664
H   0.75056  5.23012  0.49455

2
SCF(BP86/BS1)= -1132.43360729
G(298 K) = -1131.910731
SCF(PBE0) = -1800.88403119
Lowest  Frequencies  =  14.9412cm-1,
32.2674cm-1

70
2
Ir -0.00094 -0.62013 -0.07779
C   0.00539  1.49496 -0.17749
C   1.20697  2.21570 -0.26324
C  -1.18657  2.22973 -0.07863
C   1.23905  3.62148 -0.29742
C  -1.20654  3.63611 -0.11068
C   0.01852  4.31591 -0.22651
H   2.19278  4.15197 -0.37002
H  -2.15396  4.17800 -0.03962
H   0.02303  5.41067 -0.25145
P  -2.28842 -0.18075  0.03863
P   2.29265 -0.18691 -0.01325
C  -3.33734 -0.55189 -1.54903
C  -3.27858 -0.57675  1.64962
C   3.48114 -0.79946 -1.40858
C   3.12280 -0.31328  1.73124
O  -2.40179  1.55117  0.06957
O   2.42075  1.51947 -0.31156
H   0.02950 -0.40624  1.50866
H  -0.02817 -1.50977 -1.53706
H  -0.02534 -0.59180 -1.81686
H  -0.00534 -2.20603  0.41097
C  -2.74031  0.32062 -2.67860
H  -1.66865  0.12511 -2.84140
H  -3.27211  0.09098 -3.62034
H  -2.86200  1.39421 -2.46586
C  -3.18469 -2.04698 -1.90729
H  -3.68929 -2.24051 -2.87221
```

H -2.12746 -2.33955 -2.00986
H -3.64439 -2.70792 -1.15470
C -4.83100 -0.19227 -1.40983
H -5.31698 -0.31104 -2.39642
H -5.36131 -0.85504 -0.70735
H -4.97389 0.85317 -1.09242
C -2.31633 -0.35097 2.83845
H -2.87822 -0.51040 3.77750
H -1.46820 -1.05164 2.81689
H -1.91690 0.67688 2.85280
C -3.70484 -2.06029 1.62435
H -4.48563 -2.26188 0.87225
H -2.84684 -2.72656 1.42892
H -4.12258 -2.33342 2.61097
C -4.49639 0.35548 1.84224
H -5.29371 0.18317 1.10644
H -4.92593 0.16753 2.84365
H -4.19936 1.41447 1.79170
C 2.68229 -0.75993 -2.73202
H 3.36894 -0.99774 -3.56512
H 1.87123 -1.50355 -2.74590
H 2.25093 0.23678 -2.92399
C 4.71709 0.11373 -1.57709
H 5.40358 0.07258 -0.72065
H 5.28059 -0.22030 -2.46812
H 4.41964 1.16208 -1.73374
C 3.89642 -2.25727 -1.11581
H 4.56802 -2.33686 -0.24523
H 3.01914 -2.90357 -0.93921
H 4.44116 -2.66220 -1.98871
C 2.86320 -1.73174 2.28581
H 1.78802 -1.97111 2.28568
H 3.38637 -2.51142 1.70759
H 3.23514 -1.78426 3.32609
C 4.63416 -0.00538 1.73459
H 4.98800 0.00755 2.78242
H 5.22524 -0.76985 1.20492
H 4.85384 0.98272 1.29878
C 2.41927 0.73157 2.62909
H 1.32945 0.57717 2.66690
H 2.81532 0.63312 3.65673
H 2.61105 1.75946 2.28280

**Figure S80(b)**

NB: Structures in tis section are optimized in THF solvent and follow a BP86(BS2/THF/BJD3)//BP86(BS1) protocol.

**6[$NH_3$Me]**
SCF(BP86/BS1)= -1228.29787706
G(298 K) = -1227.713401
SCF(BP86+BJD3/BS2) = -1898.55544008
Lowest Frequencies = 22.6677cm-1, 34.8621cm-1

77
Ir 0.00447 0.35031 -0.14199
P -2.27006 -0.09686 -0.03737
P 2.28363 -0.05515 -0.10024
O 2.42730 -1.78548 0.06226
O -2.37831 -1.73952 0.53843
C 0.02930 -1.68334 0.34459
C 1.26765 -3.80845 0.64363
H 2.22453 -4.34017 0.65135
C -1.15528 -2.40830 0.58906
C 3.08583 1.97200 1.73632
H 3.48202 2.59227 0.91496
H 3.61193 2.26651 2.66358
H 2.01312 2.18852 1.86855
C 0.05835 -4.47734 0.90760
H 0.07026 -5.54954 1.13148
C 1.22576 -2.43018 0.35856
C -1.16672 -3.78653 0.87775
H -2.11439 -4.30154 1.06500
C -3.20395 -0.27742 -1.73749
C 3.39834 0.16545 -1.67589
C 3.27777 0.46000 1.49632
C -4.71474 -0.56619 -1.63444
H -4.92622 -1.45654 -1.02059
H -5.10453 -0.76462 -2.65081
H -5.28548 0.28703 -1.23338
C -3.44972 0.69029 1.29315
C 2.53188 -0.18116 -2.90762
H 2.15135 -1.21502 -2.86046
H 3.15944 -0.08821 -3.81400
H 1.66508 0.48852 -3.00465
C -2.63664 0.80188 2.60182
H -2.36514 -0.19526 2.98768
H -3.25769 1.30534 3.36627
H -1.70002 1.36322 2.46222
C -4.69675 -0.17088 1.60235
H -5.43247 -0.17743 0.78725
H -5.19892 0.24776 2.49471
H -4.41610 -1.21106 1.82976
C -2.53734 -1.46311 -2.47292
H -1.44598 -1.32658 -2.54387
H -2.95181 -1.52330 -3.49666
H -2.74049 -2.41877 -1.96331
C -2.97103 1.01863 -2.54458
H -3.45069 1.89599 -2.07743
H -3.40780 0.90131 -3.55428
H -1.89388 1.22426 -2.65053
C 4.78052 0.11870 1.46537
H 4.95491 -0.94686 1.24582
H 5.21002 0.32616 2.46382
H 5.34250 0.72890 0.73990
C -3.86980 2.09832 0.82158
H -2.99925 2.72072 0.55069
H -4.41069 2.61558 1.63574
H -4.54301 2.06526 -0.05092
C 2.62670 -0.31160 2.66709
H 1.53809 -0.14079 2.69371
H 3.06421 0.04667 3.61783
H 2.81287 -1.39446 2.59020
C 4.62612 -0.77416 -1.69273
H 5.37632 -0.52374 -0.93080
H 5.11848 -0.68987 -2.67961
H 4.32536 -1.82423 -1.55296
C 3.83474 1.64340 -1.77452
H 2.96852 2.32725 -1.73497

H 4.34324 1.80986 -2.74235
H 4.54295 1.92964 -0.97945
H -0.01327 1.91757 -0.77512
H 0.05877 0.90135 1.45350
H -0.03676 -0.06753 -1.74643
N -0.09005 3.79055 0.85644
H -0.06996 2.78954 0.36237
H -0.84369 3.77976 1.55728
C -0.27214 4.88600 -0.14364
H -1.22656 4.72933 -0.66327
H -0.27099 5.86083 0.36142
H 0.55150 4.83251 -0.86770
H 0.79821 3.88996 1.36722

**TS(6-2')**
SCF(BP86/BS1)= -1228.29373983
G(298 K) = -1227.716013
SCF(BP86+BJD3/BS2) = -1898.55372738
Lowest Frequencies = -827.3973cm-1, 20.0799cm-1

77
**TS(6-2')**
Ir 0.00748 -0.33910 0.12636
P -2.27314 0.11422 0.01997
P 2.30135 0.03195 0.08710
O 2.45313 1.76240 0.00424
O -2.35594 1.77471 -0.48936
C 0.05024 1.69678 -0.26370
C 1.30822 3.81664 -0.48866
H 2.26990 4.33900 -0.47844
C -1.12759 2.43638 -0.49491
C 2.99682 -1.93867 -1.83496
H 3.39818 -2.60400 -1.05180
H 3.48763 -2.21184 -2.78763
H 1.91503 -2.11977 -1.94153
C 0.10518 4.50431 -0.72933
H 0.12675 5.58384 -0.91268
C 1.25510 2.42958 -0.25530
C -1.12550 3.82383 -0.73241
H -2.06768 4.35215 -0.90846
C -3.21528 0.22287 1.71722
C 3.41294 -0.27506 1.64498
C 3.25498 -0.44571 -1.54030
C -4.72736 0.50545 1.61449
H -4.94022 1.42127 1.03975
H -5.12569 0.65581 2.63553
H -5.28891 -0.33258 1.17104
C -3.42378 -0.63269 -1.35075
C 2.57209 0.09016 2.88900
H 2.26532 1.14923 2.87408
H 3.19268 -0.07269 3.78994
H 1.66128 -0.52107 2.96765
C -2.59107 -0.71169 -2.64938
H -2.28442 0.29146 -2.99038
H -3.21460 -1.16518 -3.44226
H -1.67606 -1.30929 -2.52068
C -4.66380 0.24174 -1.64951
H -5.40280 0.23832 -0.83744
H -5.16387 -0.15801 -2.55132
H -4.37622 1.28424 -1.85733
C -2.55931 1.38337 2.50096
H -1.46667 1.25475 2.56819
H -2.97474 1.39658 3.52573
H -2.77175 2.35750 2.03175
C -2.97598 -1.10396 2.47128
H -3.44974 -1.96434 1.96857
H -3.41496 -1.02940 3.48377
H -1.89775 -1.30761 2.57309
C 4.77096 -0.16838 -1.51108
H 4.99465 0.87861 -1.25047
H 5.18106 -0.35312 -2.52179
H 5.31205 -0.83251 -0.81794
C -3.84739 -2.05427 -0.92384
H -2.97752 -2.68273 -0.66518
H -4.37677 -2.54674 -1.76031
H -4.53198 -2.04820 -0.05981
C 2.62541 0.40246 -2.66945
H 1.53143 0.27172 -2.70353
H 3.04791 0.07284 -3.63690
H 2.85056 1.47337 -2.54387
C 4.68483 0.60394 1.66884
H 5.42636 0.31653 0.91167
H 5.16604 0.49463 2.65851
H 4.43738 1.66807 1.52998
C 3.77562 -1.77477 1.70500
H 2.87598 -2.41398 1.66950
H 4.29351 -1.98601 2.65875
H 4.45294 -2.07913 0.88998
H -0.02344 -1.90670 0.78963
H 0.05427 -0.72806 -1.50578
H -0.03209 0.03157 1.74893
N -0.10210 -3.71791 -0.76951
H -0.05414 -2.43660 -0.12643
H -0.74534 -3.65271 -1.56695
C -0.51010 -4.78375 0.17341
H -1.51923 -4.55962 0.55134
H -0.51480 -5.78422 -0.28982
H 0.18554 -4.78690 1.02673
H 0.83180 -3.90312 -1.15372

**2'**
SCF(BP86/BS1)= -1132.44446789
G(298 K) = -1131.921629
SCF(BP86+BJD3/BS2) = -1802.65519905
Lowest Frequencies = 18.7237cm-1, 34.0242cm-1

70
C -1.22744 3.64201 0.12435
C -0.00000 4.32871 0.13673
C 1.22743 3.64200 0.12412
C 1.20470 2.23422 0.09397
C -0.00003 1.51411 0.06436
C -1.20473 2.23423 0.09419
O 2.41811 1.54754 0.11253
P 2.30784 -0.18708 0.01473
C 3.40242 -0.65175 1.53754
C 2.53530 -0.45138 2.80149
Ir 0.00001 -0.59489 0.04487
P -2.30783 -0.18708 0.01479
C -3.23281 -0.50014 -1.65732

| | | | |
|---|---|---|---|
| C | -2.59469 | 0.44242 | -2.70361 |
| O | -2.41814 | 1.54754 | 0.11288 |
| C | 3.23281 | -0.50048 | -1.65732 |
| C | 2.59446 | 0.44169 | -2.70381 |
| C | -3.40245 | -0.65211 | 1.53745 |
| C | -2.53525 | -0.45225 | 2.80143 |
| C | -4.64989 | 0.25131 | 1.67081 |
| C | -3.79932 | -2.14065 | 1.42806 |
| C | -2.99378 | -1.96898 | -2.07346 |
| C | -4.74602 | -0.20492 | -1.60038 |
| C | 4.74598 | -0.20505 | -1.60057 |
| C | 2.99404 | -1.96948 | -2.07307 |
| C | 3.79915 | -2.14037 | 1.42858 |
| C | 4.64995 | 0.25158 | 1.67063 |
| H | 2.17994 | 4.17950 | 0.14278 |
| H | -2.17994 | 4.17952 | 0.14319 |
| H | 0.00000 | 5.42352 | 0.15953 |
| H | 0.00011 | -0.24585 | 1.64609 |
| H | 0.00024 | -1.98937 | -0.80448 |
| H | -0.00027 | -0.30880 | -1.57973 |
| H | 0.00013 | -1.93962 | 0.96849 |
| H | -3.45820 | -2.13860 | -3.06220 |
| H | -1.91842 | -2.19357 | -2.15536 |
| H | -3.44470 | -2.68688 | -1.36879 |
| H | -5.15871 | -0.30339 | -2.62133 |
| H | -5.29293 | -0.91617 | -0.96154 |
| H | -4.95581 | 0.82057 | -1.25617 |
| H | -3.04897 | 0.22923 | -3.68832 |
| H | -2.77961 | 1.50111 | -2.46178 |
| H | -1.50723 | 0.28687 | -2.78875 |
| H | -4.29415 | -2.44979 | 2.36674 |
| H | -4.50873 | -2.33018 | 0.60605 |
| H | -2.91864 | -2.79141 | 1.28820 |
| H | -5.14374 | 0.01959 | 2.63220 |
| H | -4.37376 | 1.31729 | 1.68034 |
| H | -5.38924 | 0.08953 | 0.87515 |
| H | -3.15946 | -0.66909 | 3.68758 |
| H | -1.66488 | -1.12471 | 2.81576 |
| H | -2.17083 | 0.58500 | 2.89047 |
| H | 3.45840 | -2.13923 | -3.06181 |
| H | 3.44521 | -2.68709 | -1.36826 |
| H | 1.91873 | -2.19436 | -2.15481 |
| H | 3.04881 | 0.22841 | -3.68848 |
| H | 1.50704 | 0.28583 | -2.78891 |
| H | 2.77911 | 1.50047 | -2.46223 |
| H | 5.15860 | -0.30364 | -2.62154 |
| H | 4.95571 | 0.82052 | -1.25655 |
| H | 5.29304 | -0.91613 | -0.96167 |
| H | 4.29396 | -2.44931 | 2.36735 |
| H | 2.91844 | -2.79110 | 1.28889 |
| H | 4.50856 | -2.33017 | 0.60664 |
| H | 5.14381 | 0.02003 | 2.63206 |
| H | 5.38926 | 0.08958 | 0.87499 |
| H | 4.37390 | 1.31758 | 1.67992 |
| H | 3.15964 | -0.66767 | 3.68769 |
| H | 2.17074 | 0.58586 | 2.89000 |
| H | 1.66506 | -1.12398 | 2.81627 |

**(v) Figure S81**

```
1
SCF(BP86/BS1)= -1131.24545551
G(298 K) = -1130.741666
SCF(PBE0) = -1799.69208534
Lowest Frequencies = 22.9797cm-1,
29.7745cm-1

68
1
C  -0.00000  4.30331 -0.16264
C  -1.23059  3.62410 -0.13426
C  -1.21181  2.21893 -0.07368
C  -0.00000  1.48500 -0.04040
C   1.21180  2.21893 -0.07367
C   1.23058  3.62410 -0.13425
Ir -0.00000 -0.58836  0.04900
P   2.26933 -0.19876  0.01103
C   3.28558 -0.51060  1.61603
O   2.42482  1.54919 -0.04845
O  -2.42482  1.54918 -0.04847
P  -2.26933 -0.19877  0.01103
C  -3.28557 -0.51058  1.61604
C  -3.21686 -0.64591 -1.60195
C   3.21685 -0.64589 -1.60196
H   0.00001 -1.98355  0.86738
H  -0.00001 -1.96843 -0.76634
H  -2.17970  4.16642 -0.15930
H   2.17969  4.16642 -0.15928
H  -0.00001  5.39789 -0.20984
C  -3.13990 -2.17493 -1.80974
C  -4.68126 -0.16720 -1.62156
C  -2.42981  0.05718 -2.73388
C  -4.56295  0.34793  1.71803
C  -3.61938 -2.01387  1.71673
C  -2.32650 -0.11811  2.76626
C   4.68125 -0.16720 -1.62156
C   3.13987 -2.17491 -1.80977
C   2.42981  0.05722 -2.73388
C   3.61942 -2.01388  1.71669
C   4.56295  0.34793  1.71803
C   2.32652 -0.11815  2.76627
H  -5.09222 -0.31004 -2.63867
H  -5.31764 -0.74458 -0.93114
H  -4.76666  0.90282 -1.37232
H  -2.86901 -0.23089 -3.70724
H  -2.48053  1.15399 -2.64371
H  -1.36759 -0.24061 -2.72534
H  -3.56981 -2.42462 -2.79767
H  -2.09649 -2.52997 -1.78892
H  -3.71032 -2.73498 -1.05100
H  -4.98880  0.23069  2.73219
H  -4.34202  1.41529  1.56222
H  -5.33871  0.04438  0.99942
H  -4.03057 -2.22716  2.72091
H  -4.38017 -2.32186  0.97987
H  -2.72283 -2.64275  1.58076
H  -2.82791 -0.31057  3.73362
H  -1.39070 -0.70058  2.72766
H  -2.06318  0.95188  2.72620
H   2.86900 -0.23085 -3.70724
H   1.36758 -0.24056 -2.72534
H   2.48054  1.15403 -2.64370
H   5.09221 -0.31004 -2.63868
H   4.76666  0.90282 -1.37232
H   5.31763 -0.74459 -0.93115
H   3.56978 -2.42459 -2.79770
H   3.71029 -2.73497 -1.05103
H   2.09646 -2.52994 -1.78895
H   4.03061 -2.22718  2.72086
H   2.72288 -2.64277  1.58071
H   4.38021 -2.32185  0.97981
H   2.82794 -0.31063  3.73361
H   2.06318  0.95183  2.72623
H   1.39073 -0.70064  2.72766
H   4.98881  0.23068  2.73218
H   5.33871  0.04442  0.99940
H   4.34200  1.41530  1.56225

TS(1-5cis)
SCF(BP86/BS1)= -1253.76290018
G(298 K) = -1253.164254
SCF(PBE0) = -1922.12203342
Lowest Frequencies = -52.4572cm-1,
20.6293cm-1

79
TS(1-5cis)
Ir  0.00006 -0.56553 -0.56646
P   2.26751 -0.47099 -0.12466
P  -2.26741 -0.47115 -0.12468
O  -2.42202  0.83209  1.05133
O   2.42200  0.83208  1.05156
C  -0.00001  0.77132  1.03563
C  -1.23041  2.24992  2.58788
H  -2.18153  2.61294  2.98877
C   1.20896  1.27846  1.56336
C  -2.79243 -3.25498  0.18623
H  -3.41175 -3.31479 -0.72211
H  -3.09287 -4.08804  0.84905
H  -1.74243 -3.41429 -0.10902
C  -0.00010  2.71760  3.09111
H  -0.00013  3.46176  3.89480
C  -1.20902  1.27846  1.56325
C   1.23025  2.24992  2.58799
H   2.18135  2.61294  2.98897
C   3.55864  0.11141 -1.43644
C  -3.55864  0.11083 -1.43655
C  -2.97284 -1.92141  0.94573
C   4.74498  0.88707 -0.82685
H   4.39793  1.74116 -0.22517
H   5.36857  1.28047 -1.65138
H   5.39014  0.25998 -0.19385
C   2.97313 -1.92137  0.94544
C  -2.78622  1.03924 -2.39720
H  -2.41111  1.94663 -1.90116
H  -3.46641  1.35492 -3.21074
H  -1.91900  0.52829 -2.84781
C   2.13063 -1.96039  2.24105
H   2.25134 -1.04099  2.83545
H   2.46671 -2.81510  2.85711
```

```
H   1.05871 -2.08913  2.02563
C   4.45131 -1.71483  1.33563
H   5.13326 -1.78846  0.47354
H   4.74476 -2.50590  2.05103
H   4.60835 -0.74247  1.83027
C   2.78612  1.04014 -2.39668
H   1.91877  0.52941 -2.84730
H   3.46618  1.35605 -3.21024
H   2.41115  1.94740 -1.90027
C   4.04709 -1.11739 -2.23523
H   4.70046 -1.78070 -1.64548
H   4.63549 -0.76276 -3.10156
H   3.20370 -1.70972 -2.63044
C  -4.45103 -1.71491  1.33593
H  -4.60812 -0.74245  1.83036
H  -4.74437 -2.50584  2.05153
H  -5.13301 -1.78880  0.47390
C   2.79282 -3.25480  0.18566
H   1.74286 -3.41405 -0.10977
H   3.09315 -4.08798  0.84838
H   3.41228 -3.31444 -0.72259
C  -2.13030 -1.96009  2.24133
H  -1.05835 -2.08859  2.02590
H  -2.46616 -2.81481  2.85748
H  -2.25122 -1.04066  2.83562
C  -4.74492  0.88667 -0.82706
H  -5.39000  0.25977 -0.19379
H  -5.36862  1.27980 -1.65165
H  -4.39781  1.74096 -0.22570
C  -4.04718 -1.11821 -2.23492
H  -3.20384 -1.71064 -2.63006
H  -4.63569 -0.76384 -3.10128
H  -4.70048 -1.78136 -1.64490
H   0.00010 -1.45347 -1.96925
H   0.00011 -2.03434 -0.07618
H  -0.00036  2.04917 -1.57604
B  -0.00061  3.28715 -1.67075
H   1.01889  3.70820 -2.19627
H  -1.02060  3.70779 -2.19564
N  -0.00029  3.85324 -0.14854
H  -0.80655  3.47057  0.37006
H   0.80647  3.47099  0.36957
C  -0.00065  5.33785 -0.04355
H  -0.00036  5.66405  1.00896
H   0.89102  5.72176 -0.55974
H  -0.89290  5.72128 -0.55909
```

5cis
SCF(BP86/BS1)= -1253.76896295
G(298 K) = -1253.168205
SCF(PBE0) = -1922.12950672
Lowest Frequencies = 16.8448cm-1, 35.1147cm-1

```
79
5cis
Ir  0.05555 -0.26005 -0.67829
P   2.32477 -0.23873 -0.16285
P  -2.19363 -0.47428 -0.16315
O  -2.41338  0.63767  1.19465
O   2.42745  0.83135  1.22945
C   0.01149  0.65722  1.20879
C  -1.27281  1.67869  3.05656
H  -2.23653  1.90108  3.52473
C   1.19953  1.09568  1.83450
C  -2.34040 -3.32794 -0.21835
H  -2.94534 -3.34711 -1.13841
H  -2.52752 -4.27437  0.32297
H  -1.27890 -3.30465 -0.51301
C  -0.06164  2.09307  3.64478
H  -0.08817  2.64486  4.59039
C  -1.21140  0.97201  1.83969
C   1.18215  1.81611  3.04639
H   2.11778  2.14483  3.50841
C   3.73170  0.51518 -1.26703
C  -3.66707  0.10148 -1.29269
C  -2.69780 -2.14344  0.70734
C   4.90187  1.10657 -0.45061
H   4.53988  1.83263  0.29397
H   5.57814  1.63916 -1.14551
H   5.49895  0.34623  0.07136
C   2.99071 -1.87948  0.65055
C  -3.15117  1.27345 -2.14940
H  -3.00340  2.18977 -1.55433
H  -3.90958  1.51781 -2.91661
H  -2.20278  1.03647 -2.65369
C   2.14815 -2.12863  1.92022
H   2.28153 -1.32936  2.66609
H   2.46991 -3.08307  2.37704
H   1.07729 -2.20575  1.68621
C   4.46785 -1.79459  1.08979
H   5.16849 -1.73878  0.24239
H   4.71815 -2.71157  1.65575
H   4.64513 -0.93506  1.75644
C   3.09766  1.65213 -2.08966
H   2.22257  1.30988 -2.66393
H   3.85362  2.04006 -2.79819
H   2.77425  2.48542 -1.44989
C   4.21945 -0.56946 -2.25415
H   4.79432 -1.37281 -1.76731
H   4.88611 -0.09425 -2.99742
H   3.37882 -1.02272 -2.80779
C  -4.18538 -2.21146  1.11270
H  -4.47711 -1.35155  1.73724
H  -4.34134 -3.12620  1.71488
H  -4.86775 -2.27511  0.25075
C   2.78794 -3.05686 -0.32949
H   1.73961 -3.12958 -0.66168
H   3.05361 -4.00026  0.18377
H   3.42170 -2.97812 -1.22642
C  -1.86491 -2.24142  2.00460
H  -0.78787 -2.15078  1.80712
H  -2.05036 -3.22756  2.46947
H  -2.14501 -1.46243  2.73096
C  -4.91199  0.57619 -0.51279
H  -5.43534 -0.23777  0.00691
H  -5.62651  1.02613 -1.22770
H  -4.64934  1.34377  0.23274
C  -4.00858 -1.05844 -2.25615
H  -3.11181 -1.41785 -2.79013
H  -4.72276 -0.69150 -3.01637
H  -4.48227 -1.91327 -1.74917
```

```
H   0.09599 -0.89080 -2.21713
H   0.13852 -1.77663 -0.27213
H  -0.01456  1.45624 -1.51627
B  -0.15016  2.74365 -1.53355
H   0.93375  3.27793 -1.55268
H  -0.86709  3.01033 -2.47434
N  -0.86003  3.21160 -0.17310
H  -1.77444  2.74684 -0.06580
H  -0.30747  2.83912  0.62015
C  -1.02417  4.68729 -0.05459
H  -1.48660  4.95367  0.90857
H  -0.03176  5.15237 -0.13703
H  -1.64982  5.04183 -0.88655
```

TS(5cis-7)
SCF(BP86/BS1)= -1253.73453002
G(298 K) = -1253.142047
SCF(PBE0) = -1922.09114687
Lowest Frequencies = -411.8945cm-1, 19.0353cm-1

```
79
TS(5cis-7)
C  -0.28459 -4.64325 -0.08370
C  -1.46282 -3.88005 -0.04532
C  -1.36363 -2.48054 -0.00000
C  -0.12785 -1.78427  0.01209
C   1.02317 -2.61108 -0.03564
C   0.97039 -4.01278 -0.08189
Ir -0.01825  0.24351  0.10811
P   2.23059 -0.29183  0.00651
C   3.18665  0.06314 -1.63855
O   2.28545 -2.01493 -0.04070
O  -2.55312 -1.75381  0.02922
P  -2.32174 -0.04410  0.02239
C  -3.24501  0.37529 -1.62576
C  -3.38082  0.40219  1.57387
C   3.34139 -0.02052  1.56818
B   0.12040  3.18285 -1.12166
N   0.86523  3.81022  0.16470
H   0.01996  1.77358  1.61481
H   0.01477  0.87104  1.69813
H   0.05703  1.91812 -0.91283
H  -0.99484  3.66395 -1.16155
H   0.80763  3.38841 -2.10468
H   0.43994  3.35331  1.00433
H   1.84546  3.49444  0.17541
H  -2.45024 -4.35085 -0.04997
H   1.90038 -4.58785 -0.11520
H  -0.34473 -5.73566 -0.11879
C  -3.25141  1.91303  1.86270
C  -4.86451  0.00682  1.43434
C  -2.75784 -0.39974  2.74115
C  -4.46596 -0.53755 -1.87191
C  -3.66104  1.86021 -1.64619
C  -2.20709  0.11572 -2.74397
C   4.77769 -0.55700  1.41030
C   3.37179  1.48032  1.92599
C   2.63689 -0.79757  2.70565
C   3.66591  1.52822 -1.67519
C   4.36810 -0.90201 -1.87603
C   2.13933 -0.16141 -2.75616
H  -5.36700  0.14623  2.41019
H  -5.39801  0.63438  0.70190
H  -4.97913 -1.05068  1.14643
H  -3.28030 -0.13266  3.67878
H  -2.86156 -1.48487  2.58352
H  -1.68580 -0.17566  2.86594
H  -3.75874  2.14379  2.81814
H  -2.19467  2.21563  1.95901
H  -3.71719  2.53377  1.08034
H  -4.84699 -0.34429 -2.89237
H  -4.19334 -1.60179 -1.80350
H  -5.29221 -0.34653 -1.17124
H  -4.02647  2.11791 -2.65809
H  -4.48198  2.07194 -0.94046
H  -2.81407  2.52958 -1.42035
H  -2.68060  0.31600 -3.72393
H  -1.32614  0.76877 -2.64127
H  -1.85832 -0.93040 -2.74115
H   3.17334 -0.61255  3.65516
H   1.58862 -0.47922  2.82962
H   2.64041 -1.88138  2.51106
H   5.28404 -0.52737  2.39372
H   4.78623 -1.60180  1.06096
H   5.37918  0.05371  0.71704
H   3.93501  1.62157  2.86734
H   3.87455  2.08782  1.15284
H   2.35274  1.87294  2.08761
H   4.10179  1.74419 -2.66868
H   2.82681  2.23227 -1.53665
H   4.44864  1.73386 -0.92505
H   2.61891  0.01912 -3.73701
H   1.75660 -1.19530 -2.74883
H   1.28110  0.52132 -2.65425
H   4.72489 -0.77247 -2.91510
H   5.22199 -0.70848 -1.21018
H   4.05846 -1.95101 -1.75043
C   0.80978  5.29562  0.26881
H   1.33562  5.65292  1.16923
H   1.26858  5.72925 -0.63152
H  -0.24641  5.59684  0.30424
```

7
SCF(BP86/BS1)= -1252.57496072
G(298 K) = -1251.994592
SCF(PBE0) = -1920.93809891
Lowest Frequencies = 21.7005cm-1, 22.6815cm-1

```
77
7
C   0.08306 -4.07210 -2.00922
C  -1.14712 -3.47914 -1.68149
C  -1.14514 -2.21814 -1.06244
C   0.04073 -1.49939 -0.76396
C   1.24971 -2.16633 -1.09014
C   1.29367 -3.42580 -1.70966
Ir  0.01182  0.29375  0.17502
P  -2.25498 -0.15016  0.10695
O  -2.37874 -1.66624 -0.72510
O   2.46761 -1.56157 -0.77688
```

```
P   2.28584 -0.07371  0.08881
C   3.09722 -0.54787  1.77482
C   3.48209  0.97038 -1.01616
C  -3.51295  0.80763 -1.01187
C  -3.03459 -0.62450  1.80731
B  -0.16041  2.95600  0.97045
N   0.11154  3.26044 -0.59449
H  -2.09690 -3.97984 -1.89077
H   2.25945 -3.88464 -1.94053
H   0.09897 -5.05444 -2.49255
C   3.79893  2.30612 -0.31088
C   4.78984  0.23991 -1.39198
C   2.69521  1.21495 -2.32612
C   4.57805 -0.96288  1.69002
C   2.92104  0.64004  2.74645
C   2.26430 -1.74693  2.28579
C  -4.73806 -0.04122 -1.41799
C  -3.96392  2.10497 -0.31092
C  -2.72860  1.14450 -2.30138
C  -2.89772  0.58732  2.75563
C  -4.49939 -1.09564  1.73862
C  -2.15533 -1.78171  2.33627
H  -0.16285  2.34968 -1.05547
H   1.12452  3.33164 -0.75598
H   0.64046  3.58311  1.64021
H  -1.31740  3.22182  1.21507
H   0.09864  1.72277  1.28577
H   3.28009  1.88001 -2.98946
H   2.51641  0.26727 -2.85967
H   1.70565  1.66496 -2.14506
H   5.31034  0.81993 -2.17767
H   5.48292  0.14162 -0.54441
H   4.58174 -0.76428 -1.79265
H   4.27130  3.00726 -1.02439
H   2.89960  2.79454  0.10758
H   4.50321  2.16894  0.52676
H   4.88849 -1.38523  2.66442
H   4.74584 -1.73551  0.92226
H   5.24226 -0.10715  1.48466
H   3.26830  0.33955  3.75291
H   3.50879  1.52398  2.44705
H   1.86313  0.94050  2.82264
H   2.60933 -2.01735  3.30146
H   1.19203 -1.49212  2.33352
H   2.37736 -2.62866  1.63472
H  -3.39487  1.67799 -3.00557
H  -1.85458  1.78430 -2.09903
H  -2.35944  0.23276 -2.79898
H  -5.31682  0.51526 -2.17956
H  -4.43111 -1.00306 -1.85614
H  -5.41469 -0.24282 -0.57551
H  -4.52001  2.73813 -1.02826
H  -4.64145  1.90146  0.53532
H  -3.11028  2.68920  0.07443
H  -4.79523 -1.49171  2.72857
H  -5.19393 -0.27315  1.50031
H  -4.63727 -1.90291  1.00107
H  -3.22914  0.29200  3.76904
H  -1.85145  0.92794  2.81940
H  -3.51679  1.44382  2.44177
H  -2.48889 -2.04939  3.35654
H  -2.23339 -2.67747  1.69934
H  -1.09424 -1.48288  2.37902
C  -0.57433  4.46157 -1.14827
H  -1.65943  4.31201 -1.06510
H  -0.29214  5.33908 -0.54695
H  -0.29951  4.62487 -2.20252
```

TS(7-1)
SCF(BP86/BS1)= -1252.56269187
G(298 K) = -1251.986746
SCF(PBE0) = -1920.92330065
Lowest Frequencies = -63.1422cm-1, 23.7973cm-1

```
77
TS(7-1)
C   0.09794 -4.04746 -2.08977
C  -1.14006 -3.45547 -1.78560
C  -1.14445 -2.20738 -1.14088
C   0.04595 -1.52649 -0.78885
C   1.26335 -2.17415 -1.10892
C   1.31056 -3.42177 -1.75317
Ir  0.00662  0.24757  0.23319
P  -2.27357 -0.16504  0.08649
O  -2.37705 -1.63152 -0.83916
O   2.47173 -1.56508 -0.77539
P   2.30751 -0.06229  0.08264
C   3.11810 -0.53891  1.76001
C   3.43636  1.01315 -1.04167
C  -3.45658  0.91834 -0.96854
C  -3.05804 -0.74436  1.74693
B   0.06313  3.08223  0.90363
N   0.10770  3.44995 -0.55902
H  -2.08414 -3.94555 -2.03960
H   2.27423 -3.88531 -1.98232
H   0.11803 -5.01966 -2.59311
C   3.77015  2.33853 -0.32355
C   4.72804  0.27841 -1.46062
C   2.59792  1.28983 -2.31324
C   4.54167 -1.11392  1.63680
C   3.09455  0.69405  2.69077
C   2.17668 -1.62297  2.33824
C  -4.67979  0.12971 -1.48424
C  -3.89763  2.14266 -0.13908
C  -2.61722  1.37965 -2.18364
C  -2.96408  0.41383  2.76600
C  -4.50959 -1.24349  1.61606
C  -2.16140 -1.91158  2.22438
H  -0.03368  1.26340 -0.94207
H   1.05667  3.59806 -0.90910
H   1.08702  3.27122  1.54362
H  -0.96239  3.43046  1.46480
H  -0.07086  1.67867  1.22551
H   3.21019  1.88198 -3.01867
H   2.30905  0.35353 -2.81906
H   1.67875  1.85435 -2.09043
H   5.25344  0.89481 -2.21371
H   5.42157  0.12539 -0.62049
H   4.50659 -0.70001 -1.91407
H   4.20006  3.04812 -1.05472
H   2.88295  2.81072  0.13297
```

H 4.52224 2.19540 0.47011
H 4.84617 -1.53544 2.61319
H 4.59492 -1.92126 0.88868
H 5.27998 -0.33890 1.37542
H 3.36979 0.37673 3.71394
H 3.81517 1.46736 2.38122
H 2.09424 1.15699 2.73015
H 2.55140 -1.93488 3.33082
H 1.15254 -1.23032 2.47752
H 2.12219 -2.51476 1.69328
H -3.26286 1.97988 -2.85146
H -1.75927 1.99909 -1.87709
H -2.23767 0.52085 -2.76266
H -5.24513 0.77893 -2.17844
H -4.37364 -0.77376 -2.03357
H -5.36738 -0.16892 -0.67936
H -4.40067 2.86708 -0.80609
H -4.61977 1.86991 0.64872
H -3.04183 2.65589 0.33230
H -4.82120 -1.69933 2.57469
H -5.21369 -0.42246 1.40428
H -4.61103 -2.01014 0.83079
H -3.26211 0.03919 3.76310
H -1.93717 0.80951 2.83718
H -3.63256 1.25232 2.51479
H -2.52563 -2.26514 3.20677
H -2.17831 -2.76100 1.52309
H -1.11181 -1.59297 2.35276
C -0.77613 4.50068 -1.06546
H -1.81892 4.27309 -0.78986
H -0.54977 5.51147 -0.65826
H -0.72889 4.56885 -2.16871

1
SCF(BP86/BS1)= -1131.24545551
G(298 K) = -1130.741666
SCF(PBE0) = -1799.69208534
Lowest Frequencies = 22.9797cm-1, 29.7745cm-1

68
1
C -0.00000 4.30331 -0.16264
C -1.23059 3.62410 -0.13426
C -1.21181 2.21893 -0.07368
C -0.00000 1.48500 -0.04040
C 1.21180 2.21893 -0.07367
C 1.23058 3.62410 -0.13425
Ir -0.00000 -0.58836 0.04900
P 2.26933 -0.19876 0.01103
C 3.28558 -0.51060 1.61603
O 2.42482 1.54919 -0.04845
O -2.42482 1.54918 -0.04847
P -2.26933 -0.19877 0.01103
C -3.28557 -0.51058 1.61604
C -3.21686 -0.64591 -1.60195
C 3.21685 -0.64589 -1.60196
H 0.00001 -1.98355 0.86738
H -0.00001 -1.96843 -0.76634
H -2.17970 4.16642 -0.15930
H 2.17969 4.16642 -0.15928
H -0.00001 5.39789 -0.20984
C -3.13990 -2.17493 -1.80974
C -4.68126 -0.16720 -1.62156
C -2.42981 0.05718 -2.73388
C -4.56295 0.34793 1.71803
C -3.61938 -2.01387 1.71673
C -2.32650 -0.11811 2.76626
C 4.68125 -0.16720 -1.62156
C 3.13987 -2.17491 -1.80977
C 2.42981 0.05722 -2.73388
C 3.61942 -2.01388 1.71669
C 4.56295 0.34793 1.71803
C 2.32652 -0.11815 2.76627
H -5.09222 -0.31004 -2.63867
H -5.31764 -0.74458 -0.93114
H -4.76666 0.90282 -1.37232
H -2.86901 -0.23089 -3.70724
H -2.48053 1.15399 -2.64371
H -1.36759 -0.24061 -2.72534
H -3.56981 -2.42462 -2.79767
H -2.09649 -2.52997 -1.78892
H -3.71032 -2.73498 -1.05100
H -4.98880 0.23069 2.73219
H -4.34202 1.41529 1.56222
H -5.33871 0.04438 0.99942
H -4.03057 -2.22716 2.72091
H -4.38017 -2.32186 0.97987
H -2.72283 -2.64275 1.58076
H -2.82791 -0.31057 3.73362
H -1.39070 -0.70058 2.72766
H -2.06318 0.95188 2.72620
H 2.86900 -0.23085 -3.70724
H 1.36758 -0.24056 -2.72534
H 2.48054 1.15403 -2.64370
H 5.09221 -0.31004 -2.63868
H 4.76666 0.90282 -1.37232
H 5.31763 -0.74459 -0.93115
H 3.56978 -2.42459 -2.79770
H 3.71029 -2.73497 -1.05103
H 2.09646 -2.52994 -1.78895
H 4.03061 -2.22718 2.72086
H 2.72288 -2.64277 1.58071
H 4.38021 -2.32185 0.97981
H 2.82794 -0.31063 3.73361
H 2.06318 0.95183 2.72623
H 1.39073 -0.70064 2.72766
H 4.98881 0.23068 2.73218
H 5.33871 0.04442 0.99940
H 4.34200 1.41530 1.56225

**(vi) Figure S82**

1
SCF(BP86/BS1)= -1131.24545551
G(298 K) = -1130.741666
SCF(PBE0) = -1799.69208534
Lowest Frequencies = 22.9797cm-1, 29.7745cm-1

68
1
```
C  -0.00000  4.30331 -0.16264
C  -1.23059  3.62410 -0.13426
C  -1.21181  2.21893 -0.07368
C  -0.00000  1.48500 -0.04040
C   1.21180  2.21893 -0.07367
C   1.23058  3.62410 -0.13425
Ir -0.00000 -0.58836  0.04900
P   2.26933 -0.19876  0.01103
C   3.28558 -0.51060  1.61603
O   2.42482  1.54919 -0.04845
O  -2.42482  1.54918 -0.04847
P  -2.26933 -0.19877  0.01103
C  -3.28557 -0.51058  1.61604
C  -3.21686 -0.64591 -1.60195
C   3.21685 -0.64589 -1.60196
H   0.00001 -1.98355  0.86738
H  -0.00001 -1.96843 -0.76634
H  -2.17970  4.16642 -0.15930
H   2.17969  4.16642 -0.15928
H  -0.00001  5.39789 -0.20984
C  -3.13990 -2.17493 -1.80974
C  -4.68126 -0.16720 -1.62156
C  -2.42981  0.05718 -2.73388
C  -4.56295  0.34793  1.71803
C  -3.61938 -2.01387  1.71673
C  -2.32650 -0.11811  2.76626
C   4.68125 -0.16720 -1.62156
C   3.13987 -2.17491 -1.80977
C   2.42981  0.05722 -2.73388
C   3.61942 -2.01388  1.71669
C   4.56295  0.34793  1.71803
C   2.32652 -0.11815  2.76627
H  -5.09222 -0.31004 -2.63867
H  -5.31764 -0.74458 -0.93114
H  -4.76666  0.90282 -1.37232
H  -2.86901 -0.23089 -3.70724
H  -2.48053  1.15399 -2.64371
H  -1.36759 -0.24061 -2.72534
H  -3.56981 -2.42462 -2.79767
H  -2.09649 -2.52997 -1.78892
H  -3.71032 -2.73498 -1.05100
H  -4.98880  0.23069  2.73219
H  -4.34202  1.41529  1.56222
H  -5.33871  0.04438  0.99942
H  -4.03057 -2.22716  2.72091
H  -4.38017 -2.32186  0.97987
H  -2.72283 -2.64275  1.58076
H  -2.82791 -0.31057  3.73362
H  -1.39070 -0.70058  2.72766
H  -2.06318  0.95188  2.72620
H   2.86900 -0.23085 -3.70724
H   1.36758 -0.24056 -2.72534
H   2.48054  1.15403 -2.64370
H   5.09221 -0.31004 -2.63868
H   4.76666  0.90282 -1.37232
H   5.31763 -0.74459 -0.93115
H   3.56978 -2.42459 -2.79770
H   3.71029 -2.73497 -1.05103
H   2.09646 -2.52994 -1.78895
H   4.03061 -2.22718  2.72086
H   2.72288 -2.64277  1.58071
H   4.38021 -2.32185  0.97981
H   2.82794 -0.31063  3.73361
H   2.06318  0.95183  2.72623
H   1.39073 -0.70064  2.72766
H   4.98881  0.23068  2.73218
H   5.33871  0.04442  0.99940
H   4.34200  1.41530  1.56225
```

8
SCF(BP86/BS1)= -1130.02067650
G(298 K) = -1129.531146
SCF(PBE0) = -1798.48049030
Lowest Frequencies = 23.8909cm-1, 28.5828cm-1

66
8
```
Ir  0.00000 -0.51792  0.08781
C  -0.00001  1.43628 -0.13330
C  -1.20858  2.17715 -0.22452
C   1.20856  2.17717 -0.22451
C  -1.22183  3.57040 -0.39005
C   1.22178  3.57042 -0.39004
C  -0.00003  4.25935 -0.47042
H  -2.18002  4.09431 -0.45450
H   2.17996  4.09434 -0.45448
H  -0.00003  5.34620 -0.59986
P   2.27472 -0.20004  0.03198
P  -2.27471 -0.20007  0.03197
C   3.30534 -0.41998  1.64120
C   3.19083 -0.82076 -1.54150
C  -3.30531 -0.42000  1.64123
C  -3.19084 -0.82078 -1.54150
O   2.43403  1.51498 -0.14922
O  -2.43405  1.51494 -0.14924
C   2.38317  0.11160  2.76483
H   1.41280 -0.41397  2.77315
H   2.88236 -0.03332  3.74164
H   2.17839  1.18802  2.64136
C   3.57490 -1.92267  1.86443
H   4.01417 -2.07131  2.86842
H   2.64478 -2.51731  1.82005
H   4.28788 -2.33438  1.13018
C   4.61916  0.38828  1.65145
H   5.05205  0.35455  2.66895
H   5.37406 -0.01440  0.95990
H   4.43859  1.44420  1.39512
C   2.49070 -0.09884 -2.71663
H   2.87013 -0.51089 -3.67042
```

```
H   1.39740 -0.24927 -2.67748
H   2.68917  0.98461 -2.70263
C   2.92214 -2.33895 -1.65372
H   3.40240 -2.91762 -0.84693
H   1.83751 -2.54856 -1.63600
H   3.32463 -2.71194 -2.61418
C   4.70195 -0.52939 -1.58169
H   5.26293 -1.12366 -0.84146
H   5.09617 -0.79764 -2.58009
H   4.91812  0.53835 -1.41402
C  -2.38323  0.11187  2.76480
H  -2.88241 -0.03295  3.74162
H  -1.41279 -0.41358  2.77321
H  -2.17857  1.18829  2.64114
C  -4.61925  0.38804  1.65139
H  -5.37410 -0.01487  0.95991
H  -5.05211  0.35440  2.66890
H  -4.43886  1.44395  1.39488
C  -3.57464 -1.92270  1.86466
H  -4.28762 -2.33459  1.13052
H  -2.64444 -2.51722  1.82028
H  -4.01381 -2.07128  2.86870
C  -2.92189 -2.33891 -1.65395
H  -1.83722 -2.54833 -1.63632
H  -3.40200 -2.91779 -0.84721
H  -3.32436 -2.71184 -2.61444
C  -4.70203 -0.52967 -1.58152
H  -5.09630 -0.79796 -2.57988
H  -5.26282 -1.12403 -0.84123
H  -4.91836  0.53804 -1.41381
C  -2.49096 -0.09859 -2.71660
H  -1.39761 -0.24877 -2.67752
H  -2.87036 -0.51061 -3.67041
H  -2.68966  0.98482 -2.70246
```

7
SCF(BP86/BS1)= -1252.57496072
G(298 K) = -1251.994592
SCF(PBE0) = -1920.93809891
Lowest Frequencies = 21.7005cm-1, 22.6815cm-1

```
77
7
C   0.08306 -4.07210 -2.00922
C  -1.14712 -3.47914 -1.68149
C  -1.14514 -2.21814 -1.06244
C   0.04073 -1.49939 -0.76396
C   1.24971 -2.16633 -1.09014
C   1.29367 -3.42580 -1.70966
Ir  0.01182  0.29375  0.17502
P  -2.25498 -0.15016  0.10695
O  -2.37874 -1.66624 -0.72510
O   2.46761 -1.56157 -0.77688
P   2.28584 -0.07371  0.08881
C   3.09722 -0.54787  1.77482
C   3.48209  0.97038 -1.01616
C  -3.51295  0.80763 -1.01187
C  -3.03459 -0.62450  1.80731
B  -0.16041  2.95600  0.97045
N   0.11154  3.26044 -0.59449
H  -2.09690 -3.97984 -1.89077
H   2.25945 -3.88464 -1.94053
H   0.09897 -5.05444 -2.49255
C   3.79893  2.30612 -0.31088
C   4.78984  0.23991 -1.39198
C   2.69521  1.21495 -2.32612
C   4.57805 -0.96288  1.69002
C   2.92104  0.64004  2.74645
C   2.26430 -1.74693  2.28579
C  -4.73806 -0.04122 -1.41799
C  -3.96392  2.10497 -0.31092
C  -2.72860  1.14450 -2.30138
C  -2.89772  0.58732  2.75563
C  -4.49939 -1.09564  1.73862
C  -2.15533 -1.78171  2.33627
H  -0.16285  2.34968 -1.05547
H   1.12452  3.33164 -0.75598
H   0.64046  3.58311  1.64021
H  -1.31740  3.22182  1.21507
H   0.09864  1.72277  1.28577
H   3.28009  1.88001 -2.98946
H   2.51641  0.26727 -2.85967
H   1.70565  1.66496 -2.14506
H   5.31034  0.81993 -2.17767
H   5.48292  0.14162 -0.54441
H   4.58174 -0.76428 -1.79265
H   4.27130  3.00726 -1.02439
H   2.89960  2.79454  0.10758
H   4.50321  2.16894  0.52676
H   4.88849 -1.38523  2.66442
H   4.74584 -1.73551  0.92226
H   5.24226 -0.10715  1.48466
H   3.26830  0.33955  3.75291
H   3.50879  1.52398  2.44705
H   1.86313  0.94050  2.82264
H   2.60933 -2.01735  3.30146
H   1.19203 -1.49212  2.33352
H   2.37736 -2.62866  1.63472
H  -3.39487  1.67799 -3.00557
H  -1.85458  1.78430 -2.09903
H  -2.35944  0.23276 -2.79898
H  -5.31682  0.51526 -2.17956
H  -4.43111 -1.00306 -1.85614
H  -5.41469 -0.24282 -0.57551
H  -4.52001  2.73813 -1.02826
H  -4.64145  1.90146  0.53532
H  -3.11028  2.68920  0.07443
H  -4.79523 -1.49171  2.72857
H  -5.19393 -0.27315  1.50031
H  -4.63727 -1.90291  1.00107
H  -3.22914  0.29200  3.76904
H  -1.85145  0.92794  2.81940
H  -3.51679  1.44382  2.44177
H  -2.48889 -2.04939  3.35654
H  -2.23339 -2.67747  1.69934
H  -1.09424 -1.48288  2.37902
C  -0.57433  4.46157 -1.14827
H  -1.65943  4.31201 -1.06510
H  -0.29214  5.33908 -0.54695
H  -0.29951  4.62487 -2.20252
```

TS(7-1)
SCF(BP86/BS1)= -1252.56269187
G(298 K) = -1251.986746
SCF(PBE0) = -1920.92330065
Lowest Frequencies = -63.1422cm-1, 23.7973cm-1

77
TS(7-1)

```
C   0.09794 -4.04746 -2.08977
C  -1.14006 -3.45547 -1.78560
C  -1.14445 -2.20738 -1.14088
C   0.04595 -1.52649 -0.78885
C   1.26335 -2.17415 -1.10892
C   1.31056 -3.42177 -1.75317
Ir  0.00662  0.24757  0.23319
P  -2.27357 -0.16504  0.08649
O  -2.37705 -1.63152 -0.83916
O   2.47173 -1.56508 -0.77539
P   2.30751 -0.06229  0.08264
C   3.11810 -0.53891  1.76001
C   3.43636  1.01315 -1.04167
C  -3.45658  0.91834 -0.96854
C  -3.05804 -0.74436  1.74693
B   0.06313  3.08223  0.90363
N   0.10770  3.44995 -0.55902
H  -2.08414 -3.94555 -2.03960
H   2.27423 -3.88531 -1.98232
H   0.11803 -5.01966 -2.59311
C   3.77015  2.33853 -0.32355
C   4.72804  0.27841 -1.46062
C   2.59792  1.28983 -2.31324
C   4.54167 -1.11392  1.63680
C   3.09455  0.69405  2.69077
C   2.17668 -1.62297  2.33824
C  -4.67979  0.12971 -1.48424
C  -3.89763  2.14266 -0.13908
C  -2.61722  1.37965 -2.18364
C  -2.96408  0.41383  2.76600
C  -4.50959 -1.24349  1.61606
C  -2.16140 -1.91158  2.22438
H  -0.03368  1.26340 -0.94207
H   1.05667  3.59806 -0.90910
H   1.08702  3.27122  1.54362
H  -0.96239  3.43046  1.46480
H  -0.07086  1.67867  1.22551
H   3.21019  1.88198 -3.01867
H   2.30905  0.35353 -2.81906
H   1.67875  1.85435 -2.09043
H   5.25344  0.89481 -2.21371
H   5.42157  0.12539 -0.62049
H   4.50659 -0.70001 -1.91407
H   4.20006  3.04812 -1.05472
H   2.88295  2.81072  0.13297
H   4.52224  2.19540  0.47011
H   4.84617 -1.53544  2.61319
H   4.59492 -1.92126  0.88868
H   5.27998 -0.33890  1.37542
H   3.36979  0.37673  3.71394
H   3.81517  1.46736  2.38122
H   2.09424  1.15699  2.73015
H   2.55140 -1.93488  3.33082
H   1.15254 -1.23032  2.47752
H   2.12219 -2.51476  1.69328
H  -3.26286  1.97988 -2.85146
H  -1.75927  1.99909 -1.87709
H  -2.23767  0.52085 -2.76266
H  -5.24513  0.77893 -2.17844
H  -4.37364 -0.77376 -2.03357
H  -5.36738 -0.16892 -0.67936
H  -4.40067  2.86708 -0.80609
H  -4.61977  1.86991  0.64872
H  -3.04183  2.65589  0.33230
H  -4.82120 -1.69933  2.57469
H  -5.21369 -0.42246  1.40428
H  -4.61103 -2.01014  0.83079
H  -3.26211  0.03919  3.76310
H  -1.93717  0.80951  2.83718
H  -3.63256  1.25232  2.51479
H  -2.52563 -2.26514  3.20677
H  -2.17831 -2.76100  1.52309
H  -1.11181 -1.59297  2.35276
C  -0.77613  4.50068 -1.06546
H  -1.81892  4.27309 -0.78986
H  -0.54977  5.51147 -0.65826
H  -0.72889  4.56885 -2.16871
```

1
SCF(BP86/BS1)= -1131.24545551
G(298 K) = -1130.741666
SCF(PBE0) = -1799.69208534
Lowest Frequencies = 22.9797cm-1, 29.7745cm-1

68
1

```
C  -0.00000  4.30331 -0.16264
C  -1.23059  3.62410 -0.13426
C  -1.21181  2.21893 -0.07368
C  -0.00000  1.48500 -0.04040
C   1.21180  2.21893 -0.07367
C   1.23058  3.62410 -0.13425
Ir -0.00000 -0.58836  0.04900
P   2.26933 -0.19876  0.01103
C   3.28558 -0.51060  1.61603
O   2.42482  1.54919 -0.04845
O  -2.42482  1.54918 -0.04847
P  -2.26933 -0.19877  0.01103
C  -3.28557 -0.51058  1.61604
C  -3.21686 -0.64591 -1.60195
C   3.21685 -0.64589 -1.60196
H   0.00001 -1.98355  0.86738
H  -0.00001 -1.96843 -0.76634
H  -2.17970  4.16642 -0.15930
H   2.17969  4.16642 -0.15928
H  -0.00001  5.39789 -0.20984
C  -3.13990 -2.17493 -1.80974
C  -4.68126 -0.16720 -1.62156
C  -2.42981  0.05718 -2.73388
C  -4.56295  0.34793  1.71803
C  -3.61938 -2.01387  1.71673
C  -2.32650 -0.11811  2.76626
```

| | | | |
|---|---|---|---|
| C | 4.68125 | -0.16720 | -1.62156 |
| C | 3.13987 | -2.17491 | -1.80977 |
| C | 2.42981 | 0.05722 | -2.73388 |
| C | 3.61942 | -2.01388 | 1.71669 |
| C | 4.56295 | 0.34793 | 1.71803 |
| C | 2.32652 | -0.11815 | 2.76627 |
| H | -5.09222 | -0.31004 | -2.63867 |
| H | -5.31764 | -0.74458 | -0.93114 |
| H | -4.76666 | 0.90282 | -1.37232 |
| H | -2.86901 | -0.23089 | -3.70724 |
| H | -2.48053 | 1.15399 | -2.64371 |
| H | -1.36759 | -0.24061 | -2.72534 |
| H | -3.56981 | -2.42462 | -2.79767 |
| H | -2.09649 | -2.52997 | -1.78892 |
| H | -3.71032 | -2.73498 | -1.05100 |
| H | -4.98880 | 0.23069 | 2.73219 |
| H | -4.34202 | 1.41529 | 1.56222 |
| H | -5.33871 | 0.04438 | 0.99942 |
| H | -4.03057 | -2.22716 | 2.72091 |
| H | -4.38017 | -2.32186 | 0.97987 |
| H | -2.72283 | -2.64275 | 1.58076 |
| H | -2.82791 | -0.31057 | 3.73362 |
| H | -1.39070 | -0.70058 | 2.72766 |
| H | -2.06318 | 0.95188 | 2.72620 |
| H | 2.86900 | -0.23085 | -3.70724 |
| H | 1.36758 | -0.24056 | -2.72534 |
| H | 2.48054 | 1.15403 | -2.64370 |
| H | 5.09221 | -0.31004 | -2.63868 |
| H | 4.76666 | 0.90282 | -1.37232 |
| H | 5.31763 | -0.74459 | -0.93115 |
| H | 3.56978 | -2.42459 | -2.79770 |
| H | 3.71029 | -2.73497 | -1.05103 |
| H | 2.09646 | -2.52994 | -1.78895 |
| H | 4.03061 | -2.22718 | 2.72086 |
| H | 2.72288 | -2.64277 | 1.58071 |
| H | 4.38021 | -2.32185 | 0.97981 |
| H | 2.82794 | -0.31063 | 3.73361 |
| H | 2.06318 | 0.95183 | 2.72623 |
| H | 1.39073 | -0.70064 | 2.72766 |
| H | 4.98881 | 0.23068 | 2.73218 |
| H | 5.33871 | 0.04442 | 0.99940 |
| H | 4.34200 | 1.41530 | 1.56225 |

**(vii) Figure S83**

1
SCF(BP86/BS1)= -1131.24545551
G(298 K) = -1130.741666
SCF(PBE0) = -1799.69208534
Lowest Frequencies = 22.9797cm-1, 29.7745cm-1

```
68
1
C  -0.00000  4.30331 -0.16264
C  -1.23059  3.62410 -0.13426
C  -1.21181  2.21893 -0.07368
C  -0.00000  1.48500 -0.04040
C   1.21180  2.21893 -0.07367
C   1.23058  3.62410 -0.13425
Ir -0.00000 -0.58836  0.04900
P   2.26933 -0.19876  0.01103
C   3.28558 -0.51060  1.61603
O   2.42482  1.54919 -0.04845
O  -2.42482  1.54918 -0.04847
P  -2.26933 -0.19877  0.01103
C  -3.28557 -0.51058  1.61604
C  -3.21686 -0.64591 -1.60195
C   3.21685 -0.64589 -1.60196
H   0.00001 -1.98355  0.86738
H  -0.00001 -1.96843 -0.76634
H  -2.17970  4.16642 -0.15930
H   2.17969  4.16642 -0.15928
H  -0.00001  5.39789 -0.20984
C  -3.13990 -2.17493 -1.80974
C  -4.68126 -0.16720 -1.62156
C  -2.42981  0.05718 -2.73388
C  -4.56295  0.34793  1.71803
C  -3.61938 -2.01387  1.71673
C  -2.32650 -0.11811  2.76626
C   4.68125 -0.16720 -1.62156
C   3.13987 -2.17491 -1.80977
C   2.42981  0.05722 -2.73388
C   3.61942 -2.01388  1.71669
C   4.56295  0.34793  1.71803
C   2.32652 -0.11815  2.76627
H  -5.09222 -0.31004 -2.63867
H  -5.31764 -0.74458 -0.93114
H  -4.76666  0.90282 -1.37232
H  -2.86901 -0.23089 -3.70724
H  -2.48053  1.15399 -2.64371
H  -1.36759 -0.24061 -2.72534
H  -3.56981 -2.42462 -2.79767
H  -2.09649 -2.52997 -1.78892
H  -3.71032 -2.73498 -1.05100
H  -4.98880  0.23069  2.73219
H  -4.34202  1.41529  1.56222
H  -5.33871  0.04438  0.99942
H  -4.03057 -2.22716  2.72091
H  -4.38017 -2.32186  0.97987
H  -2.72283 -2.64275  1.58076
H  -2.82791 -0.31057  3.73362
H  -1.39070 -0.70058  2.72766
H  -2.06318  0.95188  2.72620
H   2.86900 -0.23085 -3.70724
H   1.36758 -0.24056 -2.72534
H   2.48054  1.15403 -2.64370
H   5.09221 -0.31004 -2.63868
H   4.76666  0.90282 -1.37232
H   5.31763 -0.74459 -0.93115
H   3.56978 -2.42459 -2.79770
H   3.71029 -2.73497 -1.05103
H   2.09646 -2.52994 -1.78895
H   4.03061 -2.22718  2.72086
H   2.72288 -2.64277  1.58071
H   4.38021 -2.32185  0.97981
H   2.82794 -0.31063  3.73361
H   2.06318  0.95183  2.72623
H   1.39073 -0.70064  2.72766
H   4.98881  0.23068  2.73218
H   5.33871  0.04442  0.99940
H   4.34200  1.41530  1.56225
```

TS(1-9)
SCF(BP86/BS1)= -1253.71906804
G(298 K) = -1253.124950
SCF(PBE0) = -1922.07957462
Lowest Frequencies = -1117.9251cm-1, 21.9459cm-1

```
79
TS(1-9)
C   1.21383  3.77723 -0.79335
C  -0.00008  4.48156 -0.81686
C  -1.21796  3.82072 -0.59251
C  -1.20147  2.43767 -0.34814
C  -0.00856  1.68607 -0.32080
C   1.18851  2.39488 -0.54534
O  -2.42525  1.80265 -0.13373
P  -2.33077  0.09797  0.06310
C  -3.46727 -0.42673 -1.40971
C  -2.64852 -0.17871 -2.69845
Ir -0.01879 -0.32090  0.02744
P   2.30877  0.07609  0.00762
C   3.16864  0.22701  1.73941
C   2.48433  1.41137  2.46079
O   2.40233  1.71338 -0.52561
C  -3.19433 -0.14096  1.77805
C  -2.59689  0.92045  2.72927
C   3.48075 -0.68819 -1.33058
C   2.66176 -0.79151 -2.63861
C   4.70473  0.21860 -1.60263
C   3.93118 -2.10024 -0.90200
C   2.90130 -1.07317  2.52565
C   4.68341  0.50649  1.67527
C  -4.72745  0.01989  1.76611
C  -2.81682 -1.55506  2.27570
C  -3.81054 -1.92755 -1.29988
C  -4.74948  0.43476 -1.48519
N  -0.23242 -2.86354 -0.12637
B  -0.16998 -3.78744 -1.48347
H  -2.17216  4.35467 -0.60831
H   2.17130  4.27785 -0.96220
H   0.00282  5.55860 -1.01108
H  -0.00217 -0.80607 -1.62694
```

H -0.01792 0.05069 1.62319
C 0.61157 -3.43438 0.95067
H -1.20824 -2.85383 0.18812
H -0.91092 -3.26460 -2.30829
H 0.99904 -3.78005 -1.85174
H -0.55350 -4.89895 -1.13782
H -0.00743 -1.77283 -0.96431
H 2.86899 1.45956 3.49633
H 2.70585 2.37052 1.96717
H 1.39050 1.28638 2.50620
H 5.05079 0.67898 2.70418
H 5.25607 -0.34113 1.26554
H 4.91130 1.40989 1.08738
H 5.23700 -0.18278 -2.48446
H 4.40031 1.25126 -1.83164
H 5.42149 0.23984 -0.77060
H 4.57797 -2.52195 -1.69311
H 4.51764 -2.09886 0.03223
H 3.07578 -2.78822 -0.79361
H 3.34222 -1.12045 -3.44546
H 1.84798 -1.52835 -2.56575
H 2.23328 0.18065 -2.93383
H 3.32240 -0.97330 3.54330
H 1.81956 -1.25986 2.62250
H 3.37145 -1.95566 2.05960
H -5.09657 -0.06704 2.80507
H -5.03659 1.00741 1.38821
H -5.23260 -0.76172 1.17645
H -3.23067 -1.70814 3.28950
H -3.23010 -2.35291 1.63346
H -1.72141 -1.67313 2.33798
H -2.98473 0.73776 3.74845
H -1.49702 0.86521 2.76327
H -2.88448 1.93942 2.42641
H -3.29188 -0.40983 -3.56712
H -2.32936 0.87343 -2.78471
H -1.75700 -0.82061 -2.75516
H -4.42858 -2.21641 -2.16991
H -2.90878 -2.56362 -1.33519
H -4.39454 -2.16752 -0.39501
H -5.24592 0.22884 -2.45117
H -5.47348 0.20476 -0.69169
H -4.51554 1.51038 -1.44949
H 0.41491 -4.51998 1.01582
H 1.66964 -3.29690 0.68956
H 0.41669 -2.95028 1.92072

9
SCF(BP86/BS1)= -1253.75289991
G(298 K) = -1253.152484
SCF(PBE0) = -1922.11922754
Lowest Frequencies = 31.2221cm-1, 39.0144cm-1

79
9
C 1.22279 3.78389 -0.67628
C 0.00692 4.48679 -0.68700
C -1.21633 3.81792 -0.51957
C -1.19732 2.42392 -0.34053
C -0.00740 1.67200 -0.31106
C 1.18947 2.39077 -0.49234
O -2.42735 1.77095 -0.21012
P -2.33023 0.06858 0.02635
C -3.56249 -0.48922 -1.35336
C -2.80106 -0.37051 -2.69522
Ir -0.02607 -0.40098 -0.10743
P 2.30395 0.04300 -0.01357
C 3.08663 0.21722 1.75446
C 2.28235 1.31826 2.48461
O 2.40049 1.70017 -0.50675
C -3.09856 -0.08778 1.79566
C -2.37066 0.94863 2.68267
C 3.55858 -0.67625 -1.29782
C 2.77939 -0.84138 -2.62349
C 4.75271 0.27123 -1.55544
C 4.04263 -2.06179 -0.82050
C 2.94403 -1.10867 2.52722
C 4.56716 0.64849 1.71493
C -4.61215 0.20306 1.85646
C -2.81327 -1.51122 2.32286
C -3.95578 -1.96491 -1.12591
C -4.80952 0.42129 -1.42624
N -0.21642 -2.61959 0.07455
B -0.02236 -3.40654 -1.32043
H -2.16899 4.35460 -0.53470
H 2.18029 4.29474 -0.81102
H 0.01258 5.57221 -0.82916
H 0.03633 -0.25198 -1.92713
H -0.01443 -0.26763 1.47412
C 0.53518 -3.30197 1.15270
H -1.20473 -2.74898 0.31787
H -0.72207 -2.83838 -2.16915
H 1.16610 -3.35120 -1.62719
H -0.38584 -4.57287 -1.16717
H -0.07851 -1.07243 -1.81991
H 2.65287 1.38906 3.52404
H 2.40847 2.30290 2.00844
H 1.20577 1.08862 2.52135
H 4.89488 0.86888 2.74804
H 5.22751 -0.14407 1.32807
H 4.71342 1.56188 1.11686
H 5.33271 -0.13532 -2.40442
H 4.41534 1.28307 -1.82730
H 5.43721 0.34911 -0.69978
H 4.68255 -2.50455 -1.60577
H 4.64769 -2.00477 0.09988
H 3.19916 -2.75518 -0.66258
H 3.48996 -1.17131 -3.40345
H 1.99329 -1.60834 -2.54292
H 2.33747 0.11196 -2.96125
H 3.36559 -0.97793 3.54133
H 1.88807 -1.39878 2.63667
H 3.48511 -1.94207 2.04991
H -4.92687 0.19844 2.91669
H -4.85914 1.19364 1.44235
H -5.21258 -0.56150 1.33799
H -3.19772 -1.59813 3.35591
H -3.31025 -2.29337 1.72382
H -1.73131 -1.72021 2.34991
H -2.72364 0.83037 3.72368
H -1.27858 0.80475 2.67495

```
H  -2.58518  1.97932  2.35980
H  -3.51802 -0.54639 -3.51778
H  -2.36908  0.63446 -2.83986
H  -2.00627 -1.12726 -2.78350
H  -4.58906 -2.29967 -1.96801
H  -3.07336 -2.62815 -1.10874
H  -4.53981 -2.11169 -0.20197
H  -5.38425  0.14702 -2.32992
H  -5.48112  0.30612 -0.56481
H  -4.52801  1.48235 -1.51227
H   0.28947 -4.38072  1.15842
H   1.61320 -3.20294  0.95672
H   0.31742 -2.87397  2.14758
```

TS(9-10)
SCF(BP86/BS1)= -1253.73851921
G(298 K) = -1253.142867
SCF(PBE0) = -1922.10283277
Lowest Frequencies = -309.8875cm-1, 27.3117cm-1

```
79
TS(9-10)
C   1.20618  3.76759 -0.73036
C  -0.01505  4.45966 -0.78273
C  -1.23621  3.78522 -0.61906
C  -1.20975  2.39691 -0.40621
C  -0.01471  1.64582 -0.35038
C   1.18120  2.37943 -0.51434
O  -2.43885  1.74536 -0.25911
P  -2.32590  0.05189  0.03488
C  -3.03187 -0.05325  1.83500
C  -2.26826  1.00056  2.66962
Ir -0.02353 -0.40683 -0.11132
P   2.29585  0.04460  0.00488
C   3.00357  0.19357  1.80825
C   2.18416  1.29643  2.51793
O   2.40101  1.70767 -0.47045
N  -0.19753 -2.60057 -0.06889
B   0.06381 -3.15781 -1.54920
C  -3.61314 -0.55039 -1.27574
C  -2.89947 -0.45807 -2.64295
C  -3.98282 -2.02339 -0.99800
C  -4.87344  0.34236 -1.32644
C   3.61632 -0.64968 -1.22774
C   2.89927 -0.77820 -2.59060
C   4.82518  0.29676 -1.40692
C   4.07230 -2.04663 -0.75758
C   2.80894 -1.14366  2.55153
C   4.48959  0.60456  1.84554
C  -4.53996  0.25088  1.93997
C  -2.73993 -1.46553  2.38922
H  -2.19201  4.31507 -0.65896
H   2.16179  4.28453 -0.85513
H  -0.01528  5.54118 -0.95236
H   0.03669  0.40771 -2.56949
H  -0.00745 -0.35661  1.43044
C   0.52084 -3.40214  0.94674
H  -1.19172 -2.76974  0.11700
H  -0.39556 -2.33647 -2.35023
H   1.27275 -3.29372 -1.70282
H  -0.52388 -4.23567 -1.67095
H   0.06002 -0.32443 -2.76480
H  -5.45972  0.05733 -2.21955
H  -5.52867  0.22054 -0.45313
H  -4.60839  1.40678 -1.42239
H  -3.61763 -0.75026 -3.43123
H  -2.56345  0.56931 -2.86020
H  -2.03673 -1.14034 -2.70201
H  -4.65341 -2.37936 -1.80164
H  -3.09735 -2.68242 -1.01171
H  -4.51948 -2.15742 -0.04382
H  -4.81867  0.28352  3.00984
H  -4.79526  1.22826  1.50046
H  -5.16141 -0.52624  1.46736
H  -3.08045 -1.52043  3.43985
H  -3.26995 -2.25702  1.83275
H  -1.66106 -1.69203  2.37247
H  -2.59753  0.92516  3.72238
H  -1.17836  0.84260  2.64347
H  -2.47208  2.02332  2.31586
H   3.62393 -1.17375 -3.32644
H   2.05012 -1.47824 -2.54524
H   2.55121  0.20098 -2.95821
H   5.42497 -0.07831 -2.25686
H   4.50509  1.32260 -1.64524
H   5.48656  0.32939 -0.53021
H   4.76442 -2.46474 -1.51161
H   4.61546 -2.01555  0.20197
H   3.22274 -2.74534 -0.67678
H   4.77301  0.80270  2.89625
H   5.15666 -0.18963  1.47433
H   4.67533  1.52571  1.27082
H   3.17409 -1.03433  3.58988
H   1.74677 -1.43184  2.59594
H   3.36980 -1.97267  2.08966
H   2.52331  1.36496  3.56822
H   2.32704  2.28095  2.04606
H   1.10501  1.07627  2.52321
H   0.28912 -4.47690  0.81793
H   1.60440 -3.27185  0.80994
H   0.25647 -3.09880  1.97656
```

10
SCF(BP86/BS1)= -1252.59677257
G(298 K) = -1252.011998
SCF(PBE0) = -1920.96408799
Lowest Frequencies = 27.8108cm-1, 37.0525cm-1

```
77
10
C   1.18718  3.79533 -0.38655
C  -0.03283  4.48913 -0.38394
C  -1.24715  3.79287 -0.28090
C  -1.21374  2.39094 -0.19966
C  -0.02271  1.63463 -0.23804
C   1.16486  2.39295 -0.30280
O  -2.43956  1.73180 -0.06745
P  -2.32833  0.02350  0.03781
C  -3.12640 -0.30133  1.77605
C  -2.46824  0.69458  2.75772
```

```
Ir -0.02317 -0.42816 -0.08149
P   2.29538  0.03546  0.01112
C   3.07836 -0.05429  1.78994
C   2.36151  1.02517  2.63363
O   2.39134  1.73290 -0.27296
N  -0.19456 -2.63574 -0.21609
B  -0.03831 -2.29060 -1.71113
C  -3.55131 -0.38597 -1.40449
C  -2.80295 -0.00409 -2.70395
C  -3.87902 -1.89407 -1.39884
C  -4.84679  0.45691 -1.34956
C   3.56405 -0.45160 -1.37426
C   2.83668 -0.20118 -2.71668
C   4.83032  0.43793 -1.36198
C   3.95177 -1.93834 -1.24569
C   2.78086 -1.44910  2.37764
C   4.59430  0.21895  1.84709
C  -4.65414 -0.10540  1.84089
C  -2.76749 -1.74771  2.18512
H  -2.20789  4.31499 -0.25839
H   2.14511  4.31995 -0.44502
H  -0.03715  5.58165 -0.45187
H  -0.02190 -0.20070  1.50520
C   0.71873 -3.60371  0.41580
H  -1.15320 -2.89345  0.02928
H   0.00624 -0.90694 -1.83425
H   1.02181 -2.66580 -2.16692
H  -1.00700 -2.55871 -2.39522
H  -5.36967  0.34585 -2.31766
H  -5.54347  0.13251 -0.56478
H  -4.62572  1.52658 -1.20881
H  -3.46783 -0.21163 -3.56292
H  -2.55565  1.07016 -2.72046
H  -1.87778 -0.58019 -2.83956
H  -4.52289 -2.12798 -2.26712
H  -2.97048 -2.51166 -1.49802
H  -4.43055 -2.20176 -0.49366
H  -4.97873 -0.22115  2.89207
H  -4.95686  0.90072  1.51025
H  -5.20311 -0.85534  1.24882
H  -3.15236 -1.94667  3.20266
H  -3.22303 -2.49830  1.51431
H  -1.67498 -1.89625  2.19779
H  -2.82346  0.47051  3.78076
H  -1.36960  0.61296  2.75035
H  -2.73816  1.73467  2.51661
H   3.53253 -0.44651 -3.54052
H   1.93969 -0.82426 -2.82985
H   2.54802  0.85743 -2.82174
H   5.37075  0.27230 -2.31252
H   4.57507  1.50715 -1.30285
H   5.52453  0.19196 -0.54724
H   4.65966 -2.19847 -2.05465
H   4.45548 -2.16067 -0.28871
H   3.07625 -2.59858 -1.35309
H   4.90447  0.24874  2.90864
H   5.18557 -0.57358  1.36027
H   4.85833  1.18918  1.39792
H   3.14667 -1.48951  3.42081
H   1.69845 -1.65436  2.38497
H   3.28694 -2.25642  1.82060
H   2.70200  0.93616  3.68209
H   2.59843  2.04000  2.27802
H   1.26695  0.90119  2.61707
H   0.53960 -4.62711  0.03356
H   1.75633 -3.32741  0.18444
H   0.59494 -3.60740  1.51231
```

10'

SCF(BP86/BS1)= -1252.56352983

G(298 K) = -1251.982290

SCF(PBE0) = -1920.93347612

Lowest Frequencies = 26.8340cm-1, 33.2346cm-1

```
77
10'
C  -1.21705  3.80314 -0.45682
C  -0.00072  4.50507 -0.45709
C   1.22532  3.82966 -0.33730
C   1.20766  2.43129 -0.21413
C   0.01555  1.66802 -0.18978
C  -1.18481  2.40366 -0.33352
O   2.43865  1.77812 -0.11794
P   2.32244  0.06142 -0.01228
C   3.44477 -0.43485 -1.48448
C   2.80517  0.22556 -2.72916
Ir  0.03607 -0.37671 -0.02131
P  -2.28770  0.02112 -0.03167
C  -3.46647 -0.58063 -1.43487
C  -2.57830 -0.69184 -2.69743
O  -2.39351  1.72368 -0.36268
N   0.24282 -2.58999  0.03433
B  -0.15393 -3.30919 -1.35143
C   3.14309 -0.18957  1.71439
C   2.00000  0.12190  2.71255
C   3.59657 -1.65315  1.89220
C   4.31478  0.77801  1.97918
C  -3.14050  0.05306  1.70833
C  -2.22511  0.93306  2.59305
C  -4.54827  0.68007  1.66288
C  -3.20891 -1.36197  2.31676
C  -4.04675 -1.96581 -1.08191
C  -4.60421  0.42613 -1.71533
C   4.88985  0.07583 -1.31582
C   3.41610 -1.96991 -1.65352
H   2.17784  4.36663 -0.34753
H  -2.17516  4.32030 -0.55892
H  -0.00749  5.59536 -0.55609
H   0.02196 -0.58615 -1.54777
H   1.25629 -2.73598  0.10370
C  -0.33180 -3.32322  1.18345
H  -1.37818 -3.37085 -1.41059
H   0.29188 -2.63582 -2.28964
H   0.34978 -4.43628 -1.35018
H  -5.16705  0.06452 -2.59573
H  -5.31712  0.51307 -0.88231
H  -4.21429  1.42877 -1.94558
H  -4.59599 -2.35088 -1.96082
H  -3.25354 -2.69670 -0.85077
H  -4.76444 -1.91640 -0.24548
H  -3.22080 -0.97630 -3.55158
```

```
H  -2.10360  0.27333 -2.94508
H  -1.80033 -1.46390 -2.58406
H  -4.90013  0.84133  2.69936
H  -4.54613  1.65502  1.15057
H  -5.28059  0.01970  1.17079
H  -3.68220 -1.29797  3.31454
H  -3.80780 -2.06041  1.71124
H  -2.20652 -1.79579  2.45190
H  -2.63661  0.95806  3.61936
H  -1.19912  0.52897  2.65470
H  -2.16110  1.96854  2.22351
H   3.91446 -1.80859  2.93992
H   2.78470 -2.37334  1.68970
H   4.45470 -1.90573  1.24824
H   2.36982  0.00103  3.74812
H   1.62899  1.15444  2.60204
H   1.14758 -0.57274  2.58106
H   4.62142  0.68360  3.03776
H   5.19564  0.55315  1.35950
H   4.02170  1.82332  1.79775
H   4.02058 -2.23679 -2.54015
H   3.85608 -2.50154 -0.79349
H   2.39428 -2.34875 -1.83173
H   3.39855 -0.05564 -3.61829
H   1.77270 -0.12467 -2.89122
H   2.79771  1.32444 -2.65263
H   5.44185 -0.10279 -2.25710
H   4.92344  1.15736 -1.10631
H   5.42987 -0.45908 -0.51720
H  -0.01969 -4.38574  1.15854
H  -0.03065 -2.88387  2.15477
H  -1.42873 -3.29945  1.10515
```

TS(10'-11)
SCF(BP86/BS1)= -1252.53459554
G(298 K) = -1251.957985
SCF(PBE0) = -1920.89282494
Lowest Frequencies = -654.0922cm-1, 28.5272cm-1

```
77
TS(10'-11)
C  -1.20630  3.76597 -0.61804
C   0.00896  4.46920 -0.61012
C   1.22484  3.78735 -0.44441
C   1.20176  2.39029 -0.30440
C   0.01282  1.61951 -0.32513
C  -1.18012  2.36958 -0.47303
O   2.42872  1.74677 -0.13732
P   2.29294  0.03844  0.03773
C   3.64378 -0.43582 -1.27002
C   2.96320 -0.24533 -2.64512
Ir  0.02546 -0.40438 -0.18244
P  -2.26808  0.03702 -0.01080
C  -3.64750 -0.59819 -1.22284
C  -2.99426 -0.62316 -2.62296
O  -2.40207  1.70242 -0.46101
N   0.23268 -2.74924 -0.08239
B   0.14599 -2.94307 -1.54857
C   2.96452 -0.18427  1.84218
C   2.09070  0.74001  2.71910
C   2.72332 -1.64859  2.27040
C   4.44518  0.19167  2.03749
C  -2.94195  0.14724  1.81297
C  -2.10960  1.25409  2.49954
C  -4.43343  0.51159  1.93511
C  -2.66511 -1.19337  2.52144
C  -4.07971 -2.02555 -0.83212
C  -4.87584  0.33834 -1.29167
C   4.88364  0.48506 -1.22077
C   4.05854 -1.91128 -1.09364
H   2.18143  4.31744 -0.42547
H  -2.16518  4.28000 -0.73139
H   0.00794  5.55786 -0.72518
H  -0.01162 -0.83015 -2.01560
H   1.18857 -2.84213  0.26820
C  -0.67931 -3.52706  0.78636
H  -0.91275 -3.32105 -1.98119
H  -0.03258 -1.41389 -2.64180
H   1.17226 -3.09738 -2.15802
H  -5.50328  0.02270 -2.14636
H  -5.50495  0.29385 -0.39231
H  -4.57538  1.38370 -1.45970
H  -4.81560 -2.39829 -1.56887
H  -3.22887 -2.72789 -0.84277
H  -4.56069 -2.06676  0.15997
H  -3.75631 -0.92524 -3.36556
H  -2.61701  0.37211 -2.91071
H  -2.16106 -1.33800 -2.68208
H  -4.66938  0.68018  3.00314
H  -4.67509  1.43873  1.39171
H  -5.09809 -0.29358  1.58121
H  -2.98429 -1.12202  3.57850
H  -3.21235 -2.03749  2.06798
H  -1.58741 -1.42329  2.50400
H  -2.35997  1.27175  3.57701
H  -1.02885  1.06816  2.39326
H  -2.32570  2.24851  2.07884
H   3.05414 -1.78572  3.31703
H   1.64792 -1.89367  2.22661
H   3.28553 -2.37310  1.65540
H   2.35406  0.58649  3.78260
H   2.24807  1.80195  2.47237
H   1.02064  0.51053  2.58270
H   4.67986  0.17028  3.11870
H   5.13025 -0.51677  1.54373
H   4.66058  1.20789  1.67051
H   4.73050 -2.20284 -1.92239
H   4.60821 -2.08454 -0.15289
H   3.19151 -2.59393 -1.13092
H   3.70405 -0.45019 -3.44050
H   2.11172 -0.92627 -2.78833
H   2.60285  0.78850 -2.77667
H   5.51035  0.27442 -2.10772
H   4.59455  1.54670 -1.25402
H   5.50949  0.32092 -0.33240
H  -0.48257 -4.61288  0.70453
H  -0.56148 -3.22255  1.83752
H  -1.71795 -3.33499  0.48531
```

11
SCF(BP86/BS1)= -1251.39282802

G(298 K) = -1250.828162
SCF(PBE0) = -1919.75672181
Lowest Frequencies = 25.9552cm-1, 26.3637cm-1

75
11

| | | | |
|---|---|---|---|
| C | -1.20290 | 3.78281 | -0.10770 |
| C | 0.01669 | 4.47444 | -0.05065 |
| C | 1.23498 | 3.77920 | -0.00082 |
| C | 1.20947 | 2.37489 | -0.01833 |
| C | 0.01404 | 1.61277 | -0.09417 |
| C | -1.18049 | 2.37759 | -0.12256 |
| O | 2.43789 | 1.72437 | 0.04222 |
| P | 2.31284 | 0.00360 | 0.00138 |
| C | 3.47295 | -0.28001 | -1.51568 |
| C | 2.64581 | 0.22244 | -2.72505 |
| Ir | 0.02199 | -0.42685 | -0.08516 |
| P | -2.27607 | -0.00585 | -0.02752 |
| C | -3.50270 | -0.38779 | -1.47871 |
| C | -2.68202 | -0.11552 | -2.76337 |
| O | -2.39813 | 1.72356 | -0.16758 |
| N | 0.29082 | -2.67042 | -0.14833 |
| B | -0.05174 | -2.22341 | -1.52028 |
| C | 3.16292 | -0.39389 | 1.69117 |
| C | 2.29926 | 0.33590 | 2.74681 |
| C | 3.08419 | -1.91444 | 1.94954 |
| C | 4.62065 | 0.09131 | 1.80558 |
| C | -3.11696 | -0.16673 | 1.71463 |
| C | -2.17673 | 0.59086 | 2.68215 |
| C | -4.52132 | 0.46411 | 1.78476 |
| C | -3.18065 | -1.64594 | 2.14278 |
| C | -3.96723 | -1.85813 | -1.42598 |
| C | -4.73468 | 0.54550 | -1.50013 |
| C | 4.78111 | 0.53758 | -1.45350 |
| C | 3.78599 | -1.78358 | -1.66429 |
| H | 2.19289 | 4.30410 | 0.05171 |
| H | -2.16019 | 4.31070 | -0.13732 |
| H | 0.01811 | 5.56937 | -0.03934 |
| H | 1.28143 | -2.84472 | 0.03040 |
| C | -0.53746 | -3.59482 | 0.63863 |
| H | -1.15380 | -2.54095 | -1.89657 |
| H | 0.83500 | -2.14583 | -2.33730 |
| H | -5.25681 | 0.40112 | -2.46469 |
| H | -5.45720 | 0.32106 | -0.70208 |
| H | -4.44464 | 1.60434 | -1.42608 |
| H | -4.55441 | -2.08280 | -2.33615 |
| H | -3.12181 | -2.56373 | -1.39809 |
| H | -4.62392 | -2.05114 | -0.56027 |
| H | -3.31365 | -0.34211 | -3.64304 |
| H | -2.38535 | 0.94466 | -2.82248 |
| H | -1.77347 | -0.73228 | -2.81673 |
| H | -4.82821 | 0.53264 | 2.84571 |
| H | -4.53538 | 1.48117 | 1.36272 |
| H | -5.27909 | -0.14677 | 1.26808 |
| H | -3.63482 | -1.71061 | 3.14963 |
| H | -3.79609 | -2.26184 | 1.46567 |
| H | -2.17459 | -2.08831 | 2.20233 |
| H | -2.55681 | 0.47621 | 3.71503 |
| H | -1.15039 | 0.18785 | 2.63756 |
| H | -2.13043 | 1.66695 | 2.45067 |
| H | 3.53496 | -2.14092 | 2.93390 |
| H | 2.03614 | -2.25750 | 1.98202 |
| H | 3.63476 | -2.50614 | 1.19770 |
| H | 2.66546 | 0.07377 | 3.75727 |
| H | 2.35549 | 1.42984 | 2.63131 |
| H | 1.23951 | 0.03616 | 2.66921 |
| H | 4.94584 | 0.00405 | 2.85958 |
| H | 5.31255 | -0.51730 | 1.20131 |
| H | 4.72581 | 1.14689 | 1.50863 |
| H | 4.35179 | -1.94431 | -2.60097 |
| H | 4.41321 | -2.16167 | -0.83847 |
| H | 2.86951 | -2.39350 | -1.73471 |
| H | 3.21802 | 0.02798 | -3.65162 |
| H | 1.67093 | -0.28243 | -2.79866 |
| H | 2.46795 | 1.30817 | -2.65697 |
| H | 5.26314 | 0.49771 | -2.44836 |
| H | 4.58547 | 1.59490 | -1.21594 |
| H | 5.50271 | 0.13917 | -0.72522 |
| H | -0.28935 | -4.64619 | 0.39787 |
| H | -0.39564 | -3.43788 | 1.72240 |
| H | -1.59428 | -3.42365 | 0.39158 |

**(viii) Figure S84**

4cis
SCF(BP86/BS1)= -1227.11295190
G(298 K) = -1226.539506
SCF(PBE0) = -1895.49965675
Lowest Frequencies = 29.3980cm-1, 51.5893cm-1

75
4cis
Ir -0.01256 -0.16258 -0.64196
P -2.26118 0.18363 -0.18179
P 2.24989 0.12533 -0.21399
O -2.39833 0.05599 1.55347
C -1.17360 -0.09658 2.21615
O 2.42902 -0.33898 1.46254
C 0.01387 -0.15178 1.46153
C 4.20267 -0.27543 -2.26880
H 3.36897 -0.06523 -2.96148
H 4.89271 -0.97180 -2.78125
H 4.75540 0.66022 -2.09424
C -2.97233 1.97621 -0.40392
C 3.70606 -0.93238 -0.96169
C 1.21948 -0.29334 2.17534
C -3.61172 -1.12780 -0.71754
C -3.22174 2.22482 -1.90642
H -2.31228 2.02952 -2.50004
H -3.50987 3.28138 -2.06157
H -4.03880 1.60168 -2.30815
C -5.09144 -0.70480 -0.61464
H -5.37423 -0.42477 0.41224
H -5.72233 -1.56624 -0.90732
H -5.34581 0.12387 -1.29406
C 3.11874 -2.30847 -1.34110
H 2.72685 -2.84537 -0.46135
H 3.91664 -2.93687 -1.77880
H 2.32813 -2.19582 -2.10358
C 1.26326 -0.39651 3.57711
H 2.21995 -0.50415 4.09703
C -3.30018 -1.49503 -2.18735
H -3.52765 -0.66042 -2.87207
H -3.92130 -2.35752 -2.49476
H -2.23637 -1.73992 -2.34358
C -1.90408 2.96946 0.10663
H -1.61750 2.76050 1.15072
H -2.32710 3.99053 0.06656
H -0.99550 2.95139 -0.51255
C 4.86729 -1.15846 0.03006
H 5.40196 -0.23151 0.27962
H 5.59884 -1.85119 -0.42801
H 4.50845 -1.60692 0.96930
C 4.30067 2.13499 0.34376
H 4.50453 3.20854 0.51575
H 5.02646 1.78751 -0.40830
H 4.48899 1.60622 1.29243
C 1.94944 2.66402 0.96326
H 2.13327 2.25141 1.96777
H 0.87796 2.55880 0.74260
H 2.20009 3.74131 0.98450
C -4.24777 2.21947 0.43701
H -5.13509 1.70904 0.04199
H -4.46826 3.30296 0.43374
H -4.09863 1.90958 1.48379
C 2.60492 2.67176 -1.46934
H 2.80328 3.75466 -1.35986
H 1.56568 2.54571 -1.81430
H 3.27348 2.29208 -2.25796
C 2.83268 1.98008 -0.10691
C -3.43228 -2.35598 0.20309
H -2.39980 -2.73208 0.23164
H -4.07914 -3.17739 -0.15815
H -3.71430 -2.11861 1.24041
N -0.03621 -2.47804 -0.61305
H 0.68543 -2.73948 -1.29044
H -0.92562 -2.76066 -1.03728
C -1.18203 -0.19713 3.62018
H -2.12481 -0.15346 4.17374
C 0.04777 -0.35196 4.28521
H 0.06012 -0.43171 5.37744
C 0.17648 -3.23052 0.65003
H 0.14510 -4.32547 0.49777
H -0.58766 -2.93681 1.38380
H 1.14924 -2.94675 1.07639
H 0.01150 1.39142 -0.89754
H -0.03342 -0.27144 -2.31210

TS(1-4cis)
SCF(BP86/BS1)= -1227.09682792
G(298 K) = -1226.531107
SCF(PBE0) = -1895.47665792
Lowest Frequencies = -69.1107cm-1, 27.2266cm-1

75
TS(1-4cis)
Ir 0.00350 -0.21310 -0.61644
P 2.26725 -0.27796 -0.15857
P -2.25866 -0.29093 -0.16801
O 2.42272 0.46560 1.42387
C 1.20358 0.73510 2.04270
O -2.42880 0.51216 1.38393
C -0.00276 0.44709 1.36310
C -3.88956 -0.06768 -2.45975
H -2.98039 -0.38305 -3.00050
H -4.47731 0.57700 -3.13929
H -4.50075 -0.96007 -2.24624
C 3.04094 -2.00332 0.25386
C -3.54546 0.72915 -1.18245
C -1.21364 0.75348 2.02609
C 3.49241 0.81385 -1.17788
C 3.38018 -2.74385 -1.05691
H 2.51665 -2.77947 -1.74310
H 3.66583 -3.78605 -0.82115
H 4.23006 -2.28508 -1.58918
C 4.97283 0.71018 -0.76462
H 5.11770 0.90272 0.31089
H 5.55017 1.47277 -1.32099
H 5.41177 -0.26983 -1.01282
C -2.81543 2.02896 -1.58916
H -2.66779 2.68779 -0.71832
H -3.44062 2.57775 -2.31836

H -1.82993 1.82812 -2.04125
C -1.24013 1.30926 3.31868
H -2.19231 1.52912 3.81014
C 3.31591 0.42445 -2.66429
H 3.67562 -0.59485 -2.88010
H 3.90406 1.12258 -3.28892
H 2.25821 0.48510 -2.96893
C 1.95787 -2.79513 1.02269
H 1.63747 -2.26904 1.93694
H 2.37830 -3.77444 1.31958
H 1.06555 -2.97229 0.40290
C -4.81926 1.10745 -0.39798
H -5.47277 0.24615 -0.19823
H -5.40228 1.83220 -0.99709
H -4.56988 1.58380 0.56337
C -4.43771 -2.02033 0.69878
H -4.70895 -3.01505 1.09973
H -5.10535 -1.82340 -0.15570
H -4.63871 -1.27674 1.48694
C -2.11364 -2.51788 1.50206
H -2.28799 -1.89075 2.39074
H -1.03533 -2.50236 1.28010
H -2.40943 -3.55466 1.74894
C 4.28310 -1.89445 1.16612
H 5.15385 -1.45727 0.65809
H 4.56883 -2.91172 1.49288
H 4.06714 -1.29596 2.06483
C -2.73138 -3.00481 -0.89195
H -3.04196 -4.02152 -0.58616
H -1.67149 -3.04606 -1.19067
H -3.32318 -2.73120 -1.77996
C -2.95029 -2.03796 0.29323
C 3.01650 2.26997 -0.98605
H 1.94828 2.38158 -1.23685
H 3.60610 2.92700 -1.65246
H 3.17294 2.60792 0.05200
N -0.03911 3.16152 -0.10003
H -0.88241 2.95793 0.44512
H 0.73274 3.08060 0.56985
C 1.21907 1.29415 3.33469
H 2.16730 1.50249 3.83867
C -0.01282 1.57206 3.95486
H -0.01659 2.00628 4.96046
C -0.09854 4.51725 -0.66693
H 0.82158 4.71156 -1.24409
H -0.21298 5.33792 0.07323
H -0.94450 4.57966 -1.37221
H 0.01714 -1.72871 -0.97376
H -0.00093 -0.38101 -2.26020

1
SCF(BP86/BS1)= -1131.24545551
G(298 K) = -1130.741666
SCF(PBE0) = -1799.69208534
Lowest Frequencies = 22.9797cm-1, 29.7745cm-1

68
1
C -0.00000 4.30331 -0.16264
C -1.23059 3.62410 -0.13426
C -1.21181 2.21893 -0.07368
C -0.00000 1.48500 -0.04040
C 1.21180 2.21893 -0.07367
C 1.23058 3.62410 -0.13425
Ir -0.00000 -0.58836 0.04900
P 2.26933 -0.19876 0.01103
C 3.28558 -0.51060 1.61603
O 2.42482 1.54919 -0.04845
O -2.42482 1.54918 -0.04847
P -2.26933 -0.19877 0.01103
C -3.28557 -0.51058 1.61604
C -3.21686 -0.64591 -1.60195
C 3.21685 -0.64589 -1.60196
H 0.00001 -1.98355 0.86738
H -0.00001 -1.96843 -0.76634
H -2.17970 4.16642 -0.15930
H 2.17969 4.16642 -0.15928
H -0.00001 5.39789 -0.20984
C -3.13990 -2.17493 -1.80974
C -4.68126 -0.16720 -1.62156
C -2.42981 0.05718 -2.73388
C -4.56295 0.34793 1.71803
C -3.61938 -2.01387 1.71673
C -2.32650 -0.11811 2.76626
C 4.68125 -0.16720 -1.62156
C 3.13987 -2.17491 -1.80977
C 2.42981 0.05722 -2.73388
C 3.61942 -2.01388 1.71669
C 4.56295 0.34793 1.71803
C 2.32652 -0.11815 2.76627
H -5.09222 -0.31004 -2.63867
H -5.31764 -0.74458 -0.93114
H -4.76666 0.90282 -1.37232
H -2.86901 -0.23089 -3.70724
H -2.48053 1.15399 -2.64371
H -1.36759 -0.24061 -2.72534
H -3.56981 -2.42462 -2.79767
H -2.09649 -2.52997 -1.78892
H -3.71032 -2.73498 -1.05100
H -4.98880 0.23069 2.73219
H -4.34202 1.41529 1.56222
H -5.33871 0.04438 0.99942
H -4.03057 -2.22716 2.72091
H -4.38017 -2.32186 0.97987
H -2.72283 -2.64275 1.58076
H -2.82791 -0.31057 3.73362
H -1.39070 -0.70058 2.72766
H -2.06318 0.95188 2.72620
H 2.86900 -0.23085 -3.70724
H 1.36758 -0.24056 -2.72534
H 2.48054 1.15403 -2.64370
H 5.09221 -0.31004 -2.63868
H 4.76666 0.90282 -1.37232
H 5.31763 -0.74459 -0.93115
H 3.56978 -2.42459 -2.79770
H 3.71029 -2.73497 -1.05103
H 2.09646 -2.52994 -1.78895
H 4.03061 -2.22718 2.72086
H 2.72288 -2.64277 1.58071
H 4.38021 -2.32185 0.97981
H 2.82794 -0.31063 3.73361

H   2.06318  0.95183  2.72623
H   1.39073 -0.70064  2.72766
H   4.98881  0.23068  2.73218
H   5.33871  0.04442  0.99940
H   4.34200  1.41530  1.56225

TS(1-5cis)
SCF(BP86/BS1)= -1253.76290018
G(298 K) = -1253.164254
SCF(PBE0) = -1922.12203342
Lowest Frequencies = -52.4572cm-1, 20.6293cm-1

79
TS(1-5cis)
Ir  0.00006 -0.56553 -0.56646
P   2.26751 -0.47099 -0.12466
P  -2.26741 -0.47115 -0.12468
O  -2.42202  0.83209  1.05133
O   2.42200  0.83208  1.05156
C  -0.00001  0.77132  1.03563
C  -1.23041  2.24992  2.58788
H  -2.18153  2.61294  2.98877
C   1.20896  1.27846  1.56336
C  -2.79243 -3.25498  0.18623
H  -3.41175 -3.31479 -0.72211
H  -3.09287 -4.08804  0.84905
H  -1.74243 -3.41429 -0.10902
C  -0.00010  2.71760  3.09111
H  -0.00013  3.46176  3.89480
C  -1.20902  1.27846  1.56325
C   1.23025  2.24992  2.58799
H   2.18135  2.61294  2.98897
C   3.55864  0.11141 -1.43644
C  -3.55864  0.11083 -1.43655
C  -2.97284 -1.92141  0.94573
C   4.74498  0.88707 -0.82685
H   4.39793  1.74116 -0.22517
H   5.36857  1.28047 -1.65138
H   5.39014  0.25998 -0.19385
C   2.97313 -1.92137  0.94544
C  -2.78622  1.03924 -2.39720
H  -2.41111  1.94663 -1.90116
H  -3.46641  1.35492 -3.21074
H  -1.91900  0.52829 -2.84781
C   2.13063 -1.96039  2.24105
H   2.25134 -1.04099  2.83545
H   2.46671 -2.81510  2.85711
H   1.05871 -2.08913  2.02563
C   4.45131 -1.71483  1.33563
H   5.13326 -1.78846  0.47354
H   4.74476 -2.50590  2.05103
H   4.60835 -0.74247  1.83027
C   2.78612  1.04014 -2.39668
H   1.91877  0.52941 -2.84730
H   3.46618  1.35605 -3.21024
H   2.41115  1.94740 -1.90027
C   4.04709 -1.11739 -2.23523
H   4.70046 -1.78070 -1.64548
H   4.63549 -0.76276 -3.10156
H   3.20370 -1.70972 -2.63044
C  -4.45103 -1.71491  1.33593
H  -4.60812 -0.74245  1.83036
H  -4.74437 -2.50584  2.05153
H  -5.13301 -1.78880  0.47390
C   2.79282 -3.25480  0.18566
H   1.74286 -3.41405 -0.10977
H   3.09315 -4.08798  0.84838
H   3.41228 -3.31444 -0.72259
C  -2.13030 -1.96009  2.24133
H  -1.05835 -2.08859  2.02590
H  -2.46616 -2.81481  2.85748
H  -2.25122 -1.04066  2.83562
C  -4.74492  0.88667 -0.82706
H  -5.39000  0.25977 -0.19379
H  -5.36862  1.27980 -1.65165
H  -4.39781  1.74096 -0.22570
C  -4.04718 -1.11821 -2.23492
H  -3.20384 -1.71064 -2.63006
H  -4.63569 -0.76384 -3.10128
H  -4.70048 -1.78136 -1.64490
H   0.00010 -1.45347 -1.96925
H   0.00011 -2.03434 -0.07618
H  -0.00036  2.04917 -1.57604
B  -0.00061  3.28715 -1.67075
H   1.01889  3.70820 -2.19627
H  -1.02060  3.70779 -2.19564
N  -0.00029  3.85324 -0.14854
H  -0.80655  3.47057  0.37006
H   0.80647  3.47099  0.36957
C  -0.00065  5.33785 -0.04355
H  -0.00036  5.66405  1.00896
H   0.89102  5.72176 -0.55974
H  -0.89290  5.72128 -0.55909

5cis
SCF(BP86/BS1)= -1253.77009775
G(298 K) = -1253.169801
SCF(PBE0) = -1922.13001496
Lowest Frequencies = 20.8987cm-1, 28.6401cm-1

79
5cis
C  -1.30679 -3.40496 -1.76060
C  -0.10404 -3.98034 -2.20221
C   1.12780 -3.34874 -1.96193
C   1.12323 -2.10953 -1.29850
C  -0.05588 -1.44741 -0.90399
C  -1.25370 -2.16366 -1.10110
O   2.36032 -1.53234 -0.99136
P   2.26162 -0.19196  0.09905
C   3.66927  0.83534 -0.75763
C   3.05646  1.37357 -2.06914
Ir -0.01374  0.36354  0.17844
P  -2.31504 -0.08864  0.14485
C  -3.08306 -0.46425  1.88733
C  -2.36694 -1.71945  2.43593
O  -2.44998 -1.64412 -0.60502
C   2.91529 -0.98655  1.75061
C   2.10481 -2.28474  1.97387
C  -3.58670  0.79931 -1.02088

C -2.89038 0.87864 -2.40094
C -4.89225 -0.00524 -1.22619
C -3.90169 2.20425 -0.46964
C -2.78748 0.74790 2.79885
C -4.59885 -0.74915 1.87966
C 4.40936 -1.36776 1.73916
C 2.64098 0.01144 2.89775
C 4.09280 1.99708 0.16441
C 4.90293 -0.00982 -1.15524
B -0.11550 2.85062 -1.47852
N 0.67390 3.61693 -0.30410
H 2.07402 -3.80847 -2.26176
H -2.26741 -3.90732 -1.90683
H -0.12488 -4.94493 -2.72009
H 0.02448 1.58129 -1.29831
H -0.01751 1.66466 1.25957
H -0.02622 -0.51514 1.47688
H 0.41902 3.05426 0.54891
H -1.27143 3.19324 -1.40083
H 1.68874 3.48686 -0.41651
C 0.35789 5.06473 -0.17801
H 0.40275 3.08749 -2.55348
H -3.14285 0.52613 3.82278
H -1.70901 0.96837 2.83649
H -3.30853 1.65945 2.45811
H -4.90129 -1.06236 2.89666
H -5.19254 0.14422 1.62853
H -4.86803 -1.56283 1.18773
H -2.75514 -1.93432 3.44892
H -2.54860 -2.59997 1.79954
H -1.27849 -1.57150 2.51661
H -4.52615 2.75266 -1.19965
H -4.46904 2.15599 0.47610
H -2.98673 2.79426 -0.30317
H -5.42488 0.42620 -2.09431
H -4.68368 -1.06241 -1.45311
H -5.57580 0.04751 -0.36810
H -3.55186 1.42915 -3.09568
H -1.92660 1.40106 -2.36471
H -2.72743 -0.12955 -2.81572
H 2.90982 -0.46014 3.86173
H 3.24439 0.93092 2.80198
H 1.57976 0.30434 2.93306
H 2.40229 -2.72363 2.94447
H 1.01976 -2.10180 2.00030
H 2.30427 -3.02658 1.18431
H 4.63983 -1.91052 2.67519
H 4.65980 -2.03540 0.89945
H 5.07275 -0.48829 1.70790
H 4.74149 2.69980 -0.39109
H 3.22766 2.56496 0.55210
H 4.66334 1.64584 1.04041
H 5.53634 0.59717 -1.82926
H 5.52530 -0.30266 -0.29953
H 4.60268 -0.91718 -1.70109
H 3.81265 1.98626 -2.59492
H 2.76801 0.54516 -2.73661
H 2.15791 1.98790 -1.91917
H 0.94374 5.53021 0.63058
H 0.57688 5.56551 -1.13310
H -0.71559 5.16658 0.03362

TS(5cis-5)
SCF(BP86/BS1)= -1253.74844628
G(298 K) = -1253.148703
SCF(PBE0) = -1922.11241831
Lowest Frequencies = -361.5975cm-1, 23.2071cm-1

79
TS(5cis-5)
C -1.09733 -3.06995 -2.34158
C 0.13087 -3.45327 -2.90699
C 1.32563 -2.79927 -2.56046
C 1.25965 -1.73038 -1.64806
C 0.05025 -1.26977 -1.10163
C -1.10707 -1.99349 -1.43538
O 2.45705 -1.12282 -1.25783
P 2.31455 -0.07626 0.12053
C 3.57500 1.25984 -0.49739
C 2.82631 2.11302 -1.54674
Ir 0.00971 0.28377 0.35573
P -2.25651 -0.25572 0.19542
C -3.10673 -0.95576 1.78967
C -2.38438 -2.27677 2.13948
O -2.32240 -1.64933 -0.83752
C 3.12062 -1.17324 1.50827
C 2.42279 -2.55206 1.44818
C -3.49165 0.82291 -0.85279
C -2.73744 1.13135 -2.16920
C -4.79340 0.09366 -1.25742
C -3.81449 2.11794 -0.07984
C -2.88238 0.07628 2.91760
C -4.61264 -1.25488 1.64695
C 4.63626 -1.40793 1.34561
C 2.83151 -0.50187 2.86930
C 3.98889 2.15928 0.68687
C 4.81238 0.65029 -1.19562
B -0.11213 2.63917 0.44153
N -0.46631 3.58467 -0.91999
H 2.28920 -3.11668 -2.96969
H -2.02541 -3.59611 -2.58353
H 0.16000 -4.28850 -3.61477
H 0.03841 1.43355 -0.85223
H 0.02849 1.03442 1.80963
H 0.04723 -0.96686 1.40570
H 0.23723 3.41742 -1.65428
H 0.95399 3.10931 0.78995
H -1.36345 3.27445 -1.31920
C -0.53161 5.03976 -0.60483
H -1.03161 2.97111 1.16827
H -3.28002 -0.33216 3.86550
H -1.81152 0.29760 3.04971
H -3.40560 1.02843 2.72254
H -4.95800 -1.76095 2.56795
H -5.21457 -0.33809 1.54053
H -4.82828 -1.92506 0.79964
H -2.79672 -2.66168 3.09074
H -2.53789 -3.03943 1.35950
H -1.30120 -2.12658 2.26964
H -4.37558 2.81526 -0.73150
H -4.44944 1.91780 0.80030

H -2.90472 2.62641 0.28494
H -5.28235 0.67004 -2.06578
H -4.58125 -0.91501 -1.64604
H -5.51649 0.01277 -0.43471
H -3.28858 1.90721 -2.73446
H -1.69893 1.45813 -1.99849
H -2.67356 0.23262 -2.80406
H 3.19502 -1.16023 3.68064
H 3.34457 0.46898 2.97568
H 1.75192 -0.33533 3.01030
H 2.79302 -3.16916 2.28803
H 1.32917 -2.46479 1.54219
H 2.64829 -3.07959 0.50770
H 4.96468 -2.12429 2.12206
H 4.88233 -1.84797 0.36573
H 5.22890 -0.48954 1.48557
H 4.58713 3.00948 0.30815
H 3.10973 2.57235 1.20980
H 4.61320 1.62355 1.42069
H 5.39309 1.47239 -1.65523
H 5.48353 0.12434 -0.50409
H 4.51709 -0.04793 -1.99378
H 3.53904 2.82590 -2.00222
H 2.39971 1.48938 -2.34997
H 2.02170 2.68575 -1.06438
H -1.29006 5.18539 0.17736
H 0.44432 5.34734 -0.20332
H -0.78237 5.63818 -1.49589

5
SCF(BP86/BS1)= -1253.77410882
G(298 K) = -1253.175437
SCF(PBE0) = -1922.13681746
Lowest Frequencies = 22.0867cm-1, 29.3508cm-1

79
5
C -1.28286 -3.51914 -1.60058
C -0.07246 -4.18769 -1.84581
C 1.15493 -3.59057 -1.51650
C 1.15023 -2.30648 -0.94579
C -0.03492 -1.58355 -0.69586
C -1.24016 -2.23716 -1.02830
O 2.38043 -1.73572 -0.62226
P 2.28460 -0.16931 0.09864
C 3.52192 0.71292 -1.11468
C 2.76175 0.91122 -2.44481
Ir -0.01165 0.28330 0.14112
P -2.30879 -0.10432 0.06828
C -3.14333 -0.55347 1.76293
C -2.48203 -1.87099 2.23011
O -2.45717 -1.59437 -0.78387
C 3.11684 -0.51069 1.81782
C 2.43921 -1.77871 2.38639
C -3.52098 0.93166 -1.03718
C -2.76862 1.23309 -2.35243
C -4.80941 0.16502 -1.41691
C -3.86472 2.24770 -0.30774
C -2.81419 0.57640 2.76361
C -4.66729 -0.77776 1.70179
C 4.63727 -0.76056 1.76846
C 2.80978 0.69688 2.73031
C 3.93008 2.08238 -0.53531
C 4.77119 -0.14508 -1.42523
B 0.24188 3.00335 0.92582
N -0.13204 3.50938 -0.55858
H 2.10587 -4.10165 -1.69253
H -2.24803 -3.97437 -1.84064
H -0.08637 -5.18749 -2.29150
H 0.02538 1.01837 -1.39077
H -0.20295 1.80278 1.10559
H -0.02212 -0.39665 1.64733
H 0.15480 2.71157 -1.17453
H 1.43485 3.11710 1.08101
H -1.15573 3.54501 -0.65699
C 0.45996 4.81939 -0.94493
H -0.42528 3.62381 1.73423
H -3.19860 0.29760 3.76259
H -1.72660 0.73309 2.84368
H -3.28413 1.53575 2.48664
H -5.00802 -1.13838 2.69053
H -5.22696 0.14771 1.48880
H -4.94426 -1.54271 0.95858
H -2.84330 -2.10377 3.24910
H -2.74646 -2.71199 1.56949
H -1.38456 -1.78327 2.26293
H -4.38993 2.93376 -0.99815
H -4.52585 2.08411 0.55932
H -2.96398 2.76758 0.06794
H -5.33976 0.73374 -2.20370
H -4.57170 -0.83074 -1.82249
H -5.50535 0.04402 -0.57625
H -3.41541 1.85274 -3.00157
H -1.81524 1.75537 -2.18275
H -2.52752 0.30387 -2.89440
H 3.19961 0.49051 3.74477
H 3.28531 1.62621 2.37368
H 1.72514 0.87386 2.80398
H 2.80817 -1.94242 3.41606
H 1.34375 -1.66907 2.42419
H 2.68106 -2.66981 1.78561
H 4.97909 -1.03528 2.78410
H 4.89952 -1.59283 1.09572
H 5.20621 0.13458 1.46919
H 4.52864 2.63476 -1.28409
H 3.05124 2.69734 -0.27743
H 4.54946 1.98566 0.37228
H 5.30841 0.31633 -2.27507
H 5.47717 -0.20049 -0.58589
H 4.49066 -1.16862 -1.71827
H 3.43069 1.42013 -3.16386
H 2.45874 -0.05413 -2.88315
H 1.84743 1.51012 -2.31793
H 0.14801 5.57981 -0.21343
H 1.55454 4.72871 -0.91231
H 0.13999 5.11785 -1.95575

TS(5cis-2)
SCF(BP86/BS1)= -1253.74364781
G(298 K) = -1253.151100
SCF(PBE0) = -1922.10090066

Lowest Frequencies = -414.3634cm-1, 16.0839cm-1

79
TS(5cis-2)

| | | | |
|---|---|---|---|
| C | 1.14596 | 3.77847 | -0.96093 |
| C | -0.08501 | 4.43229 | -1.12464 |
| C | -1.29278 | 3.75015 | -0.91023 |
| C | -1.24412 | 2.39083 | -0.55754 |
| C | -0.03886 | 1.66272 | -0.45995 |
| C | 1.14371 | 2.41822 | -0.60762 |
| O | -2.45294 | 1.76162 | -0.26422 |
| P | -2.32876 | 0.08423 | 0.10668 |
| C | -3.43597 | -0.54089 | -1.35042 |
| C | -2.61475 | -0.25182 | -2.63021 |
| Ir | -0.00512 | -0.30455 | 0.17317 |
| P | 2.30473 | 0.15591 | 0.08879 |
| C | 3.20661 | 0.19853 | 1.80327 |
| C | 2.55573 | 1.33640 | 2.62236 |
| O | 2.37656 | 1.81726 | -0.36431 |
| C | -3.23419 | 0.00835 | 1.81587 |
| C | -2.62684 | 1.12421 | 2.69611 |
| C | 3.44246 | -0.50033 | -1.33045 |
| C | 2.61477 | -0.33538 | -2.62770 |
| C | 4.73403 | 0.33465 | -1.49855 |
| C | 3.78212 | -1.98241 | -1.08027 |
| C | 2.95352 | -1.15673 | 2.50084 |
| C | 4.72349 | 0.46436 | 1.72408 |
| C | -4.75980 | 0.21951 | 1.74525 |
| C | -2.92897 | -1.37218 | 2.43974 |
| C | -3.69280 | -2.05213 | -1.19268 |
| C | -4.77094 | 0.23001 | -1.47362 |
| B | -0.02315 | -2.81525 | -1.70667 |
| N | -0.29030 | -3.68365 | -0.51055 |
| H | -2.25929 | 4.25483 | -0.99373 |
| H | 2.09617 | 4.30567 | -1.08321 |
| H | -0.10311 | 5.49077 | -1.40322 |
| H | 0.01888 | -1.37493 | -1.30794 |
| H | 0.11294 | -1.73603 | 1.19293 |
| H | -0.01979 | 0.46040 | 1.58284 |
| H | -0.16144 | -2.21090 | 0.49421 |
| H | 1.09393 | -2.90959 | -2.17019 |
| H | -1.21610 | -4.11398 | -0.49118 |
| C | 0.73388 | -4.60347 | -0.01127 |
| H | -0.93037 | -2.72602 | -2.50718 |
| H | 3.43589 | -1.14640 | 3.49591 |
| H | 1.87722 | -1.34109 | 2.64676 |
| H | 3.37656 | -2.00606 | 1.93742 |
| H | 5.11243 | 0.58161 | 2.75287 |
| H | 5.27339 | -0.37201 | 1.26383 |
| H | 4.95618 | 1.38988 | 1.17415 |
| H | 2.99680 | 1.33822 | 3.63635 |
| H | 2.73894 | 2.32064 | 2.16319 |
| H | 1.46696 | 1.20066 | 2.72273 |
| H | 4.36073 | -2.37091 | -1.93884 |
| H | 4.40369 | -2.12032 | -0.17832 |
| H | 2.87452 | -2.59992 | -0.99250 |
| H | 5.18643 | 0.06529 | -2.47086 |
| H | 4.52034 | 1.41482 | -1.51752 |
| H | 5.48654 | 0.13308 | -0.72397 |
| H | 3.20715 | -0.73760 | -3.47027 |
| H | 1.66067 | -0.87761 | -2.59052 |
| H | 2.41217 | 0.72841 | -2.83450 |
| H | -3.40648 | -1.43242 | 3.43527 |
| H | -3.32429 | -2.20502 | 1.83299 |
| H | -1.84618 | -1.52562 | 2.57065 |
| H | -3.06933 | 1.05617 | 3.70723 |
| H | -1.53412 | 1.02227 | 2.79274 |
| H | -2.84473 | 2.12405 | 2.28870 |
| H | -5.15441 | 0.27269 | 2.77715 |
| H | -5.02771 | 1.15988 | 1.23833 |
| H | -5.27570 | -0.61503 | 1.24418 |
| H | -4.24808 | -2.42090 | -2.07490 |
| H | -2.74995 | -2.61747 | -1.13984 |
| H | -4.30318 | -2.28014 | -0.30145 |
| H | -5.20271 | 0.00486 | -2.46642 |
| H | -5.51524 | -0.06557 | -0.72139 |
| H | -4.61725 | 1.31917 | -1.41637 |
| H | -3.17105 | -0.65172 | -3.49813 |
| H | -2.48724 | 0.83267 | -2.78088 |
| H | -1.62435 | -0.72640 | -2.61081 |
| H | 0.37149 | -5.15526 | 0.87504 |
| H | 1.07171 | -5.34676 | -0.76293 |
| H | 1.62616 | -4.03125 | 0.30274 |

2
SCF(BP86/BS1)= -1132.43360729
G(298 K) = -1131.910731
SCF(PBE0) = -1800.88403119
Lowest Frequencies = 14.9412cm-1, 32.2674cm-1

70
2

| | | | |
|---|---|---|---|
| Ir | -0.00094 | -0.62013 | -0.07779 |
| C | 0.00539 | 1.49496 | -0.17749 |
| C | 1.20697 | 2.21570 | -0.26324 |
| C | -1.18657 | 2.22973 | -0.07863 |
| C | 1.23905 | 3.62148 | -0.29742 |
| C | -1.20654 | 3.63611 | -0.11068 |
| C | 0.01852 | 4.31591 | -0.22651 |
| H | 2.19278 | 4.15197 | -0.37002 |
| H | -2.15396 | 4.17800 | -0.03962 |
| H | 0.02303 | 5.41067 | -0.25145 |
| P | -2.28842 | -0.18075 | 0.03863 |
| P | 2.29265 | -0.18691 | -0.01325 |
| C | -3.33734 | -0.55189 | -1.54903 |
| C | -3.27858 | -0.57675 | 1.64962 |
| C | 3.48114 | -0.79946 | -1.40858 |
| C | 3.12280 | -0.31328 | 1.73124 |
| O | -2.40179 | 1.55117 | 0.06957 |
| O | 2.42075 | 1.51947 | -0.31156 |
| H | 0.02950 | -0.40624 | 1.50866 |
| H | -0.02817 | -1.50977 | -1.53706 |
| H | -0.02534 | -0.59180 | -1.81686 |
| H | -0.00534 | -2.20603 | 0.41097 |
| C | -2.74031 | 0.32062 | -2.67860 |
| H | -1.66865 | 0.12511 | -2.84140 |
| H | -3.27211 | 0.09098 | -3.62034 |
| H | -2.86200 | 1.39421 | -2.46586 |
| C | -3.18469 | -2.04698 | -1.90729 |
| H | -3.68929 | -2.24051 | -2.87221 |

| | | | |
|---|---|---|---|
| H | -2.12746 | -2.33955 | -2.00986 |
| H | -3.64439 | -2.70792 | -1.15470 |
| C | -4.83100 | -0.19227 | -1.40983 |
| H | -5.31698 | -0.31104 | -2.39642 |
| H | -5.36131 | -0.85504 | -0.70735 |
| H | -4.97389 | 0.85317 | -1.09242 |
| C | -2.31633 | -0.35097 | 2.83845 |
| H | -2.87822 | -0.51040 | 3.77750 |
| H | -1.46820 | -1.05164 | 2.81689 |
| H | -1.91690 | 0.67688 | 2.85280 |
| C | -3.70484 | -2.06029 | 1.62435 |
| H | -4.48563 | -2.26188 | 0.87225 |
| H | -2.84684 | -2.72656 | 1.42892 |
| H | -4.12258 | -2.33342 | 2.61097 |
| C | -4.49639 | 0.35548 | 1.84224 |
| H | -5.29371 | 0.18317 | 1.10644 |
| H | -4.92593 | 0.16753 | 2.84365 |
| H | -4.19936 | 1.41447 | 1.79170 |
| C | 2.68229 | -0.75993 | -2.73202 |
| H | 3.36894 | -0.99774 | -3.56512 |
| H | 1.87123 | -1.50355 | -2.74590 |
| H | 2.25093 | 0.23678 | -2.92399 |
| C | 4.71709 | 0.11373 | -1.57709 |
| H | 5.40358 | 0.07258 | -0.72065 |
| H | 5.28059 | -0.22030 | -2.46812 |
| H | 4.41964 | 1.16208 | -1.73374 |
| C | 3.89642 | -2.25727 | -1.11581 |
| H | 4.56802 | -2.33686 | -0.24523 |
| H | 3.01914 | -2.90357 | -0.93921 |
| H | 4.44116 | -2.66220 | -1.98871 |
| C | 2.86320 | -1.73174 | 2.28581 |
| H | 1.78802 | -1.97111 | 2.28568 |
| H | 3.38637 | -2.51142 | 1.70759 |
| H | 3.23514 | -1.78426 | 3.32609 |
| C | 4.63416 | -0.00538 | 1.73459 |
| H | 4.98800 | 0.00755 | 2.78242 |
| H | 5.22524 | -0.76985 | 1.20492 |
| H | 4.85384 | 0.98272 | 1.29878 |
| C | 2.41927 | 0.73157 | 2.62909 |
| H | 1.32945 | 0.57717 | 2.66690 |
| H | 2.81532 | 0.63312 | 3.65673 |
| H | 2.61105 | 1.75946 | 2.28280 |

**(ix) Figure S85**

TS(5-6)2
SCF(BP86/BS1)= -1349.61453527
G(298 K) = -1348.962976
SCF(PBE0) = -2017.90638104
Lowest Frequencies = -56.9489cm-1, 14.0467cm-1

```
86
TS(5-6)2
Ir -0.04195  0.09790  0.06384
P   2.12533 -0.72623  0.08421
P  -2.35572 -0.01124  0.09806
O  -2.76079 -1.58465 -0.50832
O   2.01188 -2.35943 -0.49097
C  -0.36234 -1.89993 -0.52491
C  -1.89945 -3.75060 -1.09766
H  -2.92549 -4.10453 -1.23600
C   0.70262 -2.80260 -0.70426
C  -3.52924  2.45599 -0.68450
H  -4.14120  2.58032  0.22491
H  -3.98209  3.08567 -1.47419
H  -2.52459  2.85360 -0.44910
C  -0.79823 -4.60256 -1.28944
H  -0.96586 -5.64264 -1.58875
C  -1.65667 -2.41990 -0.71327
C   0.51436 -4.14232 -1.08847
H   1.37679 -4.80270 -1.22000
C   2.90956 -1.03313  1.83594
C  -3.18099 -0.08820  1.85554
C  -3.47110  0.98488 -1.14554
C   4.36334 -1.54478  1.82811
H   4.48502 -2.44684  1.20738
H   4.64696 -1.81291  2.86351
H   5.07868 -0.77752  1.48900
C   3.46922 -0.15054 -1.19618
C  -2.68322  1.12906  2.66372
H  -1.58302  1.17869  2.67147
H  -3.04312  1.04468  3.70668
H  -3.06507  2.08267  2.25944
C   2.75642 -0.10109 -2.56623
H   2.44081 -1.10888 -2.88243
H   3.45996  0.29298 -3.32440
H   1.85394  0.52809 -2.54329
C   4.66126 -1.12340 -1.34473
H   5.36188 -1.08247 -0.49995
H   5.22956 -0.85096 -2.25437
H   4.31245 -2.16124 -1.46507
C   2.02615 -2.10521  2.51536
H   0.96660 -1.80514  2.53049
H   2.36712 -2.23380  3.55978
H   2.10512 -3.07657  2.00193
C   2.82276  0.28885  2.62741
H   3.47700  1.07079  2.20269
H   3.15258  0.11294  3.66889
H   1.79195  0.67617  2.64212
C  -4.89590  0.41106 -1.32167
H  -4.86406 -0.67600 -1.49588
H  -5.36268  0.88210 -2.20771
H  -5.55157  0.61082 -0.46379
C   3.95455  1.25791 -0.79930
H   3.10532  1.94754 -0.64648
H   4.60174  1.67311 -1.59560
H   4.54748  1.24552  0.13144
C  -2.77652  0.87776 -2.52223
H  -1.72012  1.18397 -2.48792
H  -3.32054  1.50615 -3.25311
H  -2.79021 -0.16210 -2.88762
C  -2.66957 -1.39301  2.50953
H  -3.04792 -2.28361  1.98296
H  -3.02864 -1.42769  3.55519
H  -1.56920 -1.43610  2.51885
C  -4.72197 -0.12536  1.85668
H  -5.16857  0.82971  1.53399
H  -5.06828 -0.30716  2.89173
H  -5.11959 -0.93766  1.22724
H   0.20451  1.65948  0.68987
H   0.05208  0.57608 -1.57986
H  -0.10722 -0.32511  1.63900
B   0.40719  3.61917  1.74028
H  -0.79256  3.66958  1.82240
H   1.09237  2.97356  2.48500
N   1.04125  4.56109  0.86841
H   0.57309  3.46428 -0.79538
H   0.46936  5.31191  0.47630
N   0.18997  3.01720 -1.66187
H   0.17199  1.86696 -1.41309
C   0.93274  3.37217 -2.89741
H   0.50583  2.81624 -3.74518
H   1.98261  3.07210 -2.77379
H   0.87969  4.45306 -3.10282
H  -0.80274  3.28021 -1.72443
C   2.47844  4.85417  0.83876
H   2.86616  4.94041 -0.19369
H   2.71783  5.79545  1.36804
H   3.01316  4.03379  1.33955
```

TS(5-6)3
SCF(BP86/BS1)= -1349.60704758
G(298 K) = -1348.952908
SCF(PBE0) = -2017.89865931
Lowest Frequencies = -144.5915cm-1, 17.7406cm-1

```
86
TS(5-6)3
C  -1.18378 -3.59662 -1.96839
C  -1.16557 -2.40221 -1.22722
C   0.01961 -1.70464 -0.91517
C   1.21908 -2.36392 -1.25555
C   1.25777 -3.55742 -1.99730
C   0.04177 -4.15750 -2.36505
Ir  0.00856 -0.00764  0.31117
P   2.29282 -0.38201  0.11208
C   3.32259 -0.90674  1.67450
C   2.39962 -1.75084  2.58198
O  -2.38465 -1.92079 -0.74338
P  -2.26474 -0.46467  0.18384
C  -3.22322 -1.04665  1.77383
C  -3.68079  0.19552  2.56771
O   2.43397 -1.84415 -0.80103
```

C -3.42430 0.63272 -0.92196
C -3.28979 2.09798 -0.45988
C 3.37041 0.75057 -1.04066
C 2.78822 0.52433 -2.45564
C -4.91011 0.22426 -0.93602
C -2.86545 0.47194 -2.35531
C 4.87640 0.42716 -1.08534
C 3.16476 2.21431 -0.59857
C 3.74704 0.36305 2.44272
C 4.54888 -1.77815 1.31863
C -2.23423 -1.85421 2.64422
C -4.41958 -1.97027 1.44978
N -0.24204 3.38218 1.25782
H -1.22104 3.58660 1.49360
N 0.09650 4.23630 -1.44803
C -0.93676 5.28452 -1.43248
B -0.06188 2.98491 -2.18316
H 2.21961 -4.01095 -2.25436
H 4.49110 0.96261 1.89175
H 4.20729 0.07381 3.40618
H 2.87648 1.00382 2.66961
H 0.04982 -5.08587 -2.94577
H -2.13640 -4.08087 -2.20280
H -5.03959 -0.83782 -1.19816
H -5.43113 0.82198 -1.70840
H -5.41922 0.42119 0.02200
H 5.39528 0.66244 -0.14130
H 5.34455 1.04557 -1.87518
H 5.06090 -0.62830 -1.34124
H -1.82255 -2.71891 2.09793
H -2.77620 -2.23547 3.53068
H -1.38926 -1.23738 2.98467
H -5.25411 -1.44597 0.96483
H -4.80422 -2.39610 2.39555
H -4.11028 -2.80477 0.80144
H -1.77520 0.62008 -2.38698
H -3.33757 1.22480 -3.01412
H -3.09329 -0.52920 -2.75267
H -3.69209 2.25301 0.55701
H -3.85774 2.75518 -1.14580
H -2.23243 2.40417 -0.48862
H 4.25766 -2.63084 0.68569
H 4.98151 -2.17898 2.25453
H 5.34337 -1.22036 0.80467
H -2.83370 0.87403 2.77448
H -4.09521 -0.12267 3.54277
H -4.46955 0.76443 2.04723
H 1.53814 -1.17038 2.94416
H 2.98613 -2.09845 3.45380
H 2.01198 -2.63871 2.05559
H 2.08981 2.44828 -0.54838
H 3.63938 2.89277 -1.33332
H 3.62518 2.41614 0.38482
H 3.07657 -0.46546 -2.84199
H 3.18737 1.29642 -3.13988
H 1.68980 0.59222 -2.46115
H 0.04723 1.02276 1.65378
H 0.05236 -1.11275 1.48513
H -0.04907 1.35297 -0.85675
H -1.17172 2.72561 -2.56039
H 0.91667 2.50616 -2.67691
H -0.06513 3.69033 0.24904
H 1.04248 4.62630 -1.44493
H -1.92583 4.80701 -1.50836
H -0.90791 5.88493 -0.50206
H -0.83605 5.98399 -2.28325
H -0.13533 2.24363 1.23661
C 0.70402 3.98289 2.23013
H 0.58867 5.07656 2.27527
H 0.52916 3.55031 3.22667
H 1.72697 3.73410 1.91329

TS(5-6)4
SCF(BP86/BS1)= -1349.60675476
G(298 K) = -1348.952909
SCF(PBE0) = -2017.89861340
Lowest Frequencies = -141.9884cm-1, 16.0862cm-1

86
TS(5-6)4
Ir 0.00748 -0.00373 0.31160
P -2.25124 -0.52273 0.13699
P 2.30297 -0.32012 0.15728
O 2.49774 -1.81571 -0.69143
O -2.31754 -1.95762 -0.82548
C 0.08966 -1.71155 -0.89947
C 1.39366 -3.55722 -1.91570
H 2.37083 -3.99989 -2.13046
C -1.07320 -2.42632 -1.25428
C 3.74638 0.55707 2.45183
H 4.48327 1.13700 1.87105
H 4.20938 0.32210 3.42853
H 2.86844 1.19819 2.65063
C 0.20110 -4.17581 -2.32708
H 0.24399 -5.10876 -2.89885
C 1.30935 -2.35816 -1.18701
C -1.04610 -3.62681 -1.98549
H -1.98251 -4.12467 -2.25408
C -3.41818 0.58096 -0.95017
C 3.37393 0.77639 -1.04051
C 3.33329 -0.75375 1.74912
C -4.89859 0.15391 -0.98851
H -5.01418 -0.90130 -1.28322
H -5.42021 0.76842 -1.74716
H -5.41744 0.31705 -0.02947
C -3.21002 -1.16609 1.70190
C 4.87890 0.44765 -1.07180
H 5.39988 0.72618 -0.14061
H 5.34860 1.02509 -1.89120
H 5.05837 -0.61989 -1.27559
C -2.21728 -1.99191 2.55070
H -1.78667 -2.82861 1.97597
H -2.76228 -2.41529 3.41587
H -1.38580 -1.37599 2.92400
C -4.39275 -2.09295 1.33879
H -5.23072 -1.56301 0.86624
H -4.77794 -2.55445 2.26744
H -4.06928 -2.90223 0.66564
C -2.84596 0.45933 -2.38223
H -1.75685 0.61633 -2.40212
H -3.31884 1.22399 -3.02655

| | | | |
|---|---|---|---|
| H | -3.06278 | -0.53419 | -2.80449 |
| C | -3.30908 | 2.03390 | -0.44242 |
| H | -3.76024 | 2.15633 | 0.55783 |
| H | -3.84773 | 2.70752 | -1.13600 |
| H | -2.25385 | 2.34668 | -0.40263 |
| C | 4.56802 | -1.63350 | 1.44790 |
| H | 4.28589 | -2.52099 | 0.86058 |
| H | 4.99894 | -1.97956 | 2.40624 |
| H | 5.36084 | -1.09871 | 0.90741 |
| C | -3.68604 | 0.04556 | 2.53078 |
| H | -2.84923 | 0.72971 | 2.75743 |
| H | -4.09933 | -0.30731 | 3.49438 |
| H | -4.48088 | 0.61835 | 2.02395 |
| C | 2.41089 | -1.55229 | 2.69719 |
| H | 1.53669 | -0.96264 | 3.01129 |
| H | 2.99092 | -1.83760 | 3.59561 |
| H | 2.04103 | -2.47561 | 2.22173 |
| C | 3.16882 | 2.25805 | -0.66712 |
| H | 2.09575 | 2.50293 | -0.68750 |
| H | 3.68406 | 2.90089 | -1.40650 |
| H | 3.58591 | 2.49984 | 0.32710 |
| C | 2.78877 | 0.50219 | -2.44582 |
| H | 3.06169 | -0.50583 | -2.79405 |
| H | 3.19992 | 1.24060 | -3.15976 |
| H | 1.69125 | 0.58723 | -2.45323 |
| H | -0.13092 | 1.03777 | 1.63806 |
| H | 0.06413 | -1.09653 | 1.49822 |
| H | -0.06537 | 1.34311 | -0.86419 |
| B | -0.12024 | 2.98741 | -2.19299 |
| H | -1.25549 | 2.73681 | -2.48598 |
| H | 0.81764 | 2.49013 | -2.74792 |
| N | 0.10680 | 4.24031 | -1.48037 |
| H | 0.24229 | 3.70362 | 0.23979 |
| H | 1.04329 | 4.63907 | -1.58742 |
| C | -0.92825 | 5.28176 | -1.36595 |
| H | -1.91188 | 4.79506 | -1.28197 |
| H | -0.77606 | 5.92071 | -0.47519 |
| H | -0.95560 | 5.94455 | -2.25128 |
| C | -0.64218 | 3.98765 | 2.17765 |
| H | -0.47850 | 5.07195 | 2.26917 |
| H | -0.55492 | 3.51353 | 3.16702 |
| N | 0.33929 | 3.37104 | 1.25017 |
| H | 0.17991 | 2.23880 | 1.20642 |
| H | 1.30822 | 3.52691 | 1.55491 |
| H | -1.65142 | 3.79844 | 1.78558 |

TS(5-6)5
SCF(BP86/BS1)= -1349.59348347
G(298 K) = -1348.937919
SCF(PBE0) = -2017.88773685
Lowest Frequencies = -90.4517cm-1, 26.2548cm-1

86
TS(5-6)5

| | | | |
|---|---|---|---|
| Ir | 0.00418 | -0.88672 | -0.50247 |
| P | 2.22632 | -0.73946 | -0.00692 |
| P | -2.25624 | -0.74249 | -0.19563 |
| O | -2.46685 | 0.73597 | 0.79037 |
| O | 2.35286 | 0.75089 | 0.96753 |
| C | -0.05724 | 0.65062 | 0.88451 |
| C | -1.34134 | 2.27955 | 2.25428 |
| H | -2.30764 | 2.58052 | 2.67816 |
| C | 1.12955 | 1.23632 | 1.39357 |
| C | -2.82615 | -3.43731 | 0.52972 |
| H | -3.40044 | -3.65663 | -0.38463 |
| H | -3.14348 | -4.16175 | 1.30382 |
| H | -1.75703 | -3.60056 | 0.31532 |
| C | -0.12168 | 2.79650 | 2.77618 |
| H | -0.15014 | 3.56971 | 3.55233 |
| C | -1.28281 | 1.21849 | 1.31027 |
| C | 1.11692 | 2.30708 | 2.32233 |
| H | 2.05883 | 2.69987 | 2.71876 |
| C | 3.59681 | -0.36205 | -1.33531 |
| C | -3.53299 | -0.35310 | -1.61243 |
| C | -3.06409 | -2.00380 | 1.05391 |
| C | 4.74737 | 0.51814 | -0.80263 |
| H | 4.36471 | 1.44959 | -0.35508 |
| H | 5.41384 | 0.78762 | -1.64425 |
| H | 5.36183 | 0.01075 | -0.04484 |
| C | 2.94482 | -1.97597 | 1.31477 |
| C | -2.77810 | 0.44986 | -2.69200 |
| H | -2.49159 | 1.44922 | -2.32984 |
| H | -3.44343 | 0.58138 | -3.56698 |
| H | -1.85547 | -0.06064 | -3.00843 |
| C | 2.07597 | -1.80709 | 2.58206 |
| H | 2.20736 | -0.81247 | 3.03766 |
| H | 2.38045 | -2.56812 | 3.32504 |
| H | 1.00909 | -1.94989 | 2.35023 |
| C | 4.41212 | -1.70008 | 1.70080 |
| H | 5.11918 | -1.91256 | 0.88276 |
| H | 4.68894 | -2.35945 | 2.54539 |
| H | 4.55539 | -0.65830 | 2.03254 |
| C | 2.89671 | 0.37458 | -2.49609 |
| H | 2.07735 | -0.22670 | -2.91855 |
| H | 3.64269 | 0.58690 | -3.28606 |
| H | 2.45437 | 1.32790 | -2.16953 |
| C | 4.13425 | -1.70074 | -1.89027 |
| H | 4.75740 | -2.24795 | -1.16503 |
| H | 4.76529 | -1.49411 | -2.77519 |
| H | 3.31207 | -2.36058 | -2.21704 |
| C | -4.56377 | -1.76384 | 1.32019 |
| H | -4.75949 | -0.72939 | 1.64808 |
| H | -4.89298 | -2.43808 | 2.13374 |
| H | -5.19609 | -1.98525 | 0.44536 |
| C | 2.77560 | -3.41790 | 0.78748 |
| H | 1.73166 | -3.60004 | 0.48260 |
| H | 3.03403 | -4.13010 | 1.59411 |
| H | 3.43092 | -3.63718 | -0.07043 |
| C | -2.30804 | -1.83230 | 2.39126 |
| H | -1.22232 | -1.94563 | 2.24735 |
| H | -2.65395 | -2.61078 | 3.09712 |
| H | -2.50221 | -0.84754 | 2.84611 |
| C | -4.75237 | 0.47306 | -1.15061 |
| H | -5.41610 | -0.07698 | -0.46848 |
| H | -5.34991 | 0.75599 | -2.03848 |
| H | -4.43604 | 1.39886 | -0.64359 |
| C | -3.97427 | -1.69349 | -2.24315 |
| H | -3.10417 | -2.31026 | -2.52758 |
| H | -4.54981 | -1.48530 | -3.16475 |
| H | -4.62271 | -2.28707 | -1.57909 |
| H | 0.06143 | -1.96699 | -1.77088 |

```
H  -0.03952 -2.26557  0.41558
H   0.07923  0.26149 -1.74113
B   0.08377  2.81703 -1.93110
H   0.19679  2.98836 -3.11888
H  -0.93096  2.41660 -1.43272
N   1.05281  3.40016 -1.05467
H  -0.39135  4.32949 -0.26633
H   1.11037  2.99667 -0.10573
C   2.31245  4.02410 -1.48332
H   2.54309  4.91793 -0.87621
H   3.16281  3.32497 -1.39709
H   2.22238  4.32964 -2.53690
N  -1.26038  4.50106  0.32636
H  -2.07116  4.42532 -0.30274
H  -1.29771  3.66219  1.01886
C  -1.21858  5.79394  1.06651
H  -1.09809  6.62933  0.36237
H  -0.36496  5.75611  1.75738
H  -2.14639  5.91964  1.64214
```

TS(5-6)6
SCF(BP86/BS1)= -1349.60779025
G(298 K) = -1348.961539
SCF(PBE0) = -2017.89164212
Lowest Frequencies = -600.8260cm-1, 11.5366cm-1

```
86
TS(5-6)6
Ir  0.12046  0.01318  0.20590
P  -1.87178 -1.23128  0.12449
P   2.42364  0.48249  0.07041
O   3.11396 -0.99197 -0.50002
O  -1.36649 -2.79399 -0.39683
C   0.82222 -1.76920 -0.57391
C   2.70832 -3.22923 -1.24022
H   3.78360 -3.34215 -1.40458
C  -0.01662 -2.89306 -0.73163
C   2.59706  1.77334  2.57164
H   2.63601  2.75844  2.07544
H   3.07656  1.88179  3.56241
H   1.53944  1.50862  2.73023
C   1.81571 -4.29108 -1.45364
H   2.19432 -5.25324 -1.81332
C   2.19801 -2.00210 -0.78286
C   0.44582 -4.13907 -1.18806
H  -0.25780 -4.96707 -1.31192
C  -3.20142 -0.96745 -1.25415
C   3.17811  1.60808 -1.31080
C   3.33656  0.68017  1.76866
C  -4.13265 -2.18661 -1.44704
H  -3.56045 -3.12577 -1.50722
H  -4.66852 -2.05876 -2.40601
H  -4.89319 -2.28287 -0.65995
C  -2.66548 -1.64717  1.84176
C   2.42538  1.22036 -2.60595
H   2.63132  0.17372 -2.88475
H   2.78796  1.86791 -3.42576
H   1.33914  1.35501 -2.51512
C  -1.65015 -2.52900  2.60357
H  -1.50838 -3.50179  2.10686
H  -2.03576 -2.71151  3.62391
H  -0.66663 -2.04014  2.68986
C  -4.00711 -2.40355  1.76943
H  -4.81590 -1.78619  1.34635
H  -4.31135 -2.67711  2.79723
H  -3.92964 -3.33564  1.18769
C  -2.39426 -0.79102 -2.56261
H  -1.66649  0.02923 -2.49944
H  -3.10195 -0.56539 -3.38198
H  -1.86028 -1.71965 -2.82311
C  -4.01963  0.30020 -0.93748
H  -4.64453  0.17088 -0.03578
H  -4.70059  0.51049 -1.78404
H  -3.37204  1.18102 -0.80401
C   4.83195  1.04002  1.65961
H   5.39008  0.31654  1.04463
H   5.27128  1.02280  2.67469
H   4.99323  2.05194  1.25440
C  -2.86265 -0.31124  2.59321
H  -1.90714  0.21996  2.72711
H  -3.28511 -0.51800  3.59419
H  -3.56108  0.36556  2.07135
C   3.21475 -0.67526  2.50168
H   2.16420 -0.98811  2.61326
H   3.65295 -0.57118  3.51170
H   3.75657 -1.47266  1.96909
C   4.68215  1.33722 -1.55056
H   5.32980  1.76374 -0.77238
H   4.96398  1.81475 -2.50733
H   4.89179  0.25986 -1.63996
C   2.94181  3.09052 -0.96423
H   1.87220  3.31412 -0.83112
H   3.30791  3.71899 -1.79744
H   3.49079  3.39898 -0.05723
H  -0.30922  1.30960  1.31572
H   0.44830 -0.78377  1.56720
H  -0.31027  1.09869 -1.17797
B  -0.93031  2.50684 -1.44329
H  -1.83095  2.12265 -2.15876
H   0.04262  2.98304 -1.99104
N  -1.34869  3.13231 -0.16210
H  -2.36327  3.27131 -0.01893
H  -0.74451  1.72303  0.64762
C  -0.56270  4.19677  0.46012
H  -1.13678  4.67748  1.27216
H   0.36431  3.79152  0.91607
H  -0.25341  4.98656 -0.25412
C  -4.42736  5.13993 -0.65765
H  -3.85203  5.92523 -0.14048
H  -5.45883  5.51954 -0.81402
N  -4.33031  3.88345  0.11022
H  -4.88775  3.15665 -0.35104
H  -4.74770  4.01009  1.03854
H  -3.96238  4.99200 -1.64569
```

## 10 Single Crystal X-Ray Diffraction Data

Single crystal X-ray diffraction data were collected on a Rigaku SuperNova diffractometer with Cu Kα (λ = 1.54184 Å) radiation, equipped with a nitrogen gas Oxford Cryosystems Cryostream unit[40] and a Hybrid pixel (Hypix) array detector. Diffraction images from raw frame data were reduced using CrysAlisPro suite of programmes. The structures were solved using SHELXT,[41] and refined to convergence on $F^2$ against all independent reflections by full-matrix least-squares using SHELXL[42] (version 2018/3) through the Olex2 GUI.[43] All non-hydrogen atoms were refined anisotropically and non-hydride hydrogen atoms were geometrically placed and allowed to ride on their parent atoms. The hydride ligands in [**6**][Na(18-crown-6)(THF)$_2$] were located in the difference map and confirmed by altering the resolution of the data. Distances and angles were calculated using the full covariance matrix. Low data completeness in the dataset of [**6**][Na(18-crown-6)(THF)$_2$] (96.2%; full to 135.4 °, 92% to 153.7 °) was caused by weak diffraction at high angles, causing an absence of expected high angle reflections. Crystallographic data are available free of charge via the Cambridge Crystallographic Data Centre, under deposition numbers 2486059 and 248060.

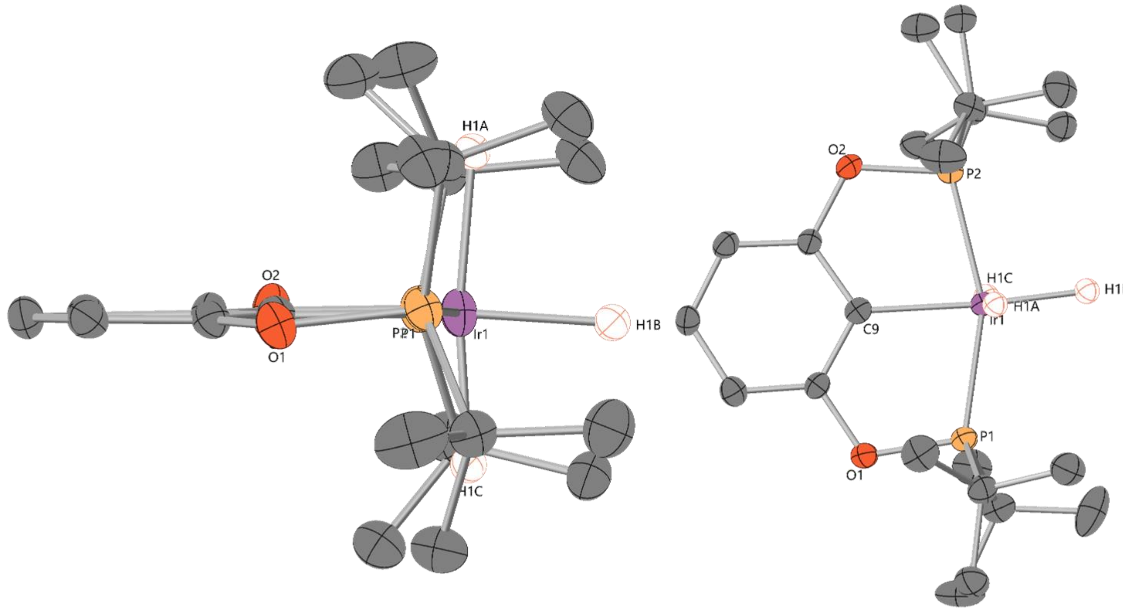


**Figure S79.** The molecular structure of [Ir($^t$Bu-POCOP)H$_3$][Na(18-crown-6)(THF)$_2$], displacement ellipsoids are set at 50% probability, hydrogen atoms and the cation are omitted for clarity. Selected bond lengths (Å): Ir1–P1 2.2514(12), Ir1–P2 2.2468(12), Ir1–C9 2.062(5), Ir–H1A 1.83(8), Ir–H1B 1.74(7), Ir–H1C 1.73(7). Selected bond angles (°): P1–Ir1–P2 158.06(5), P1–Ir1–C9 78.97(14), P2–Ir1–C9 79.21(14).

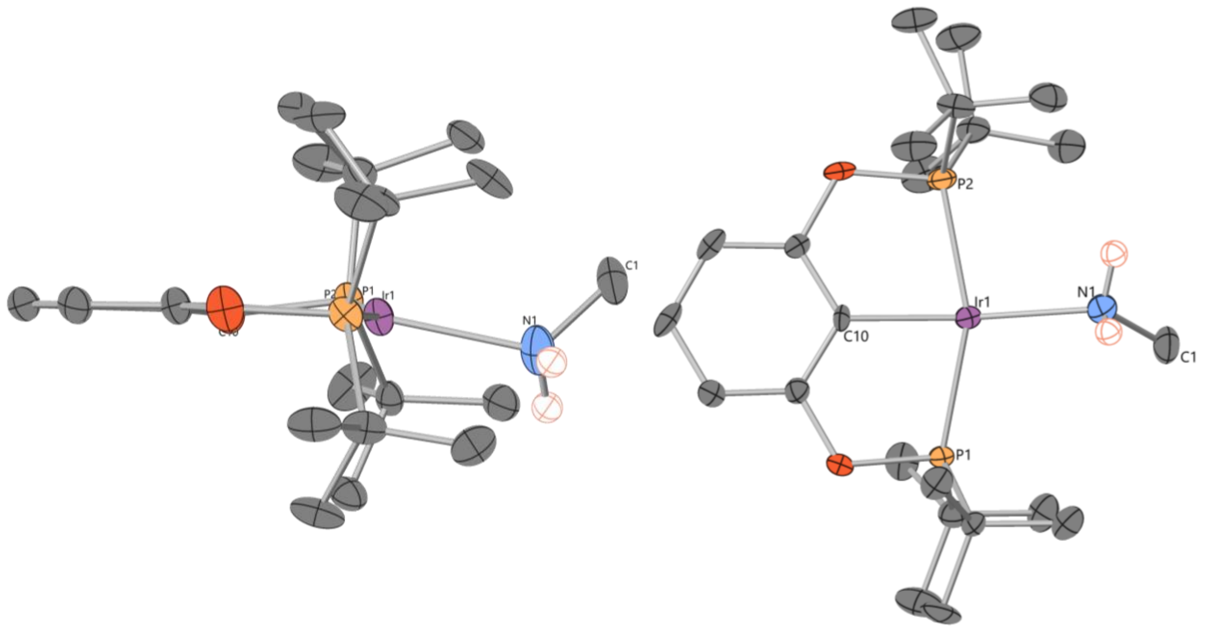


**Figure S80.** The molecular structure of [Ir($^t$Bu-POCOP)$H_2$($NH_2$Me)], displacement ellipsoids are set at 50% probability, only one complex from the asymmetric unit is shown and selected hydrogen atoms have been omitted for clarity. Selected bond lengths (Å): Ir1–N1 2.223(10), Ir1–P1 2.289(3), Ir1–P2 2.288(3), N1–C1 1.476(19), Ir1–C10 2.048(9). Selected bond angles (°): Ir1–N1–C1 121.8(7).

## 10.1 Crystallographic Data Tables

| | $[Ir(^tBu\text{-}POCOP)H_2(NH_2Me)$ | $[Ir(^tBu\text{-}POCOP)H_3][Na\text{-}18\text{-}crown\text{-}6\text{-}(THF)_2]$ |
|---|---|---|
| CCDC Deposition Number | 2486059 | 2486060 |
| Empirical formula | $C_{23}H_{44}IrNO_2P_2$ | $C_{42}H_{82}O_{10}NaP_2Ir$ |
| Formula weight | 620.73 | 1024.20 |
| Temperature/K | 110.05(10) | 110.00(10) |
| Crystal system | monoclinic | monoclinic |
| Space group | $P\ 2_1$ | $C$ 2/c |
| a/Å | 7.9232(1) | 30.2040(2) |
| b/Å | 21.2029(3) | 10.61750(10) |
| c/Å | 15.4615(2) | 30.3569(2) |
| α/° | 90 | 90 |
| β/° | 91.090(1) | 90.0680(10) |
| γ/° | 90 | 90 |
| Volume/Å$^3$ | 2596.98(6) | 9735.18(13) |
| Z | 4 | 8 |
| $\rho_{calc}$ g/cm$^3$ | 1.588 | 1.398 |
| μ/mm$^{-1}$ | 11.250 | 6.424 |
| F(000) | 1248.0 | 4256.0 |
| Crystal size/mm$^3$ | 0.186 × 0.083 × 0.047 | 0.153 × 0.082 × 0.073 |
| Radiation | Cu Kα (λ = 1.54184) | Cu Kα (λ = 1.54184) |
| 2Θ range/° | 7.076 to 154.11 | 8.254 to 153.708 |
| Index ranges | -10 ≤ h ≤ 9, -26 ≤ k ≤ 26, -19 ≤ l ≤ 19 | -34 ≤ h ≤ 38, -11 ≤ k ≤ 13, -38 ≤ l ≤ 37 |
| Reflections collected | 17246 | 33891 |
| Independent reflections | 17246 [$R_{int}$ = –, $R_{sigma}$ = 0.0319] | 9452 [$R_{int}$ = 0.0339, $R_{sigma}$ = 0.0298] |
| Data/restraints/parameters | 17246/1/550 | 9452/18/548 |
| Goodness-of-fit on F$^2$ | 1.033 | 1.261 |
| Completeness | 100 | 96.2 |
| Final R indexes [I>=2σ (I)] | $R_1$ = 0.0392, $wR_2$ = 0.1029 | $R_1$ = 0.0419, $wR_2$ = 0.1039 |
| Final R indexes [all data] | $R_1$ = 0.0403, $wR_2$ = 0.1042 | $R_1$ = 0.0431, $wR_2$ = 0.1044 |
| Largest diff. peak/hole/ e Å$^{-3}$ | 2.47 / -1.60 | 1.17 / -1.64 |
| Flack parameter | -0.031(7) | – |

## References

(1) Pangborn, A. B.; Giardello, M. A.; Grubbs, R. H.; Rosen, R. K.; Timmers, F. J. Safe and Convenient Procedure for Solvent Purification. *Organometallics* **1996**, *15* (5), 1518–1520.

(2) Göttker-Schnetmann, I.; White, P.; Brookhart, M. Iridium Bis(phosphinite) p-XPCP Pincer Complexes: Highly Active Catalysts for the Transfer Dehydrogenation of Alkanes. *J. Am. Chem. Soc.* **2004**, *126*, 1804–1811.

(3) Göttker-Schnetmann, I.; White, P. S.; Brookhart, M. Synthesis and Properties of Iridium Bis(phosphinite) Pincer Complexes (p-XPCP)Ir$H_2$, (p-XPCP)Ir(CO), (p-XPCP)Ir(H)(aryl), and {(p-XPCP)Ir}$H_2${μ-$N_2$} and Their Relevance in Alkane Transfer Dehydrogenation. *Organometallics* **2004**, *23*, 1766–1776.

(4) Denney, M. C.; Pons, V.; Hebden, T. J.; Heinekey, D. M.; Goldberg, K. I. Efficient catalysis of ammonia borane dehydrogenation. *J. Am. Chem. Soc.* **2006**, *128*, 12048–12049.

(5) Adams, G. M.; Colebatch, A. L.; Skornia, J. T.; McKay, A. I.; Johnson, H. C.; Lloyd-Jones, G. C.; Macgregor, S. A.; Beattie, N. A.; Weller, A. S. Dehydropolymerization of $H_3B{\cdot}NMeH_2$ to Form Polyaminoboranes Using [Rh(Xantphos-alkyl)] Catalysts. *J. Am. Chem. Soc.* **2018**, *140*, 1481–1495.

(6) Göttker-Schnetmann, I.; White, P.; Brookhart, M. Iridium Bis(phosphinite) p-XPCP Pincer Complexes: Highly Active Catalysts for the Transfer Dehydrogenation of Alkanes. *J. Am. Chem. Soc.* **2004**, *126* (6), 1804–1811.

(7) Titova, E. M.; Osipova, E. S.; Pavlov, A. A.; Filippov, O. A.; Safronov, S. V.; Shubina, E. S.; Belkova, N. V. Mechanism of Dimethylamine–Borane Dehydrogenation Catalyzed by an Iridium(III) PCP-Pincer Complex. *ACS Catal.* **2017**, *7* (4), 2325–2333.

(8) Hasche, P.; Haak, J.; Anke, F.; Kubis, C.; Baumann, W.; Drexler, H.-J.; Jiao, H.; Beweries, T. Dehydropolymerisation of methylamine borane using highly active rhodium(iii) bis(thiophosphinite) pincer complexes: catalytic and mechanistic insights. *Catal. Sci. Technol.* **2021**, *11* (10), 3514–3526.

(9) Saib, A.; Bara-Estaún, A.; Harper, O. J.; Berry, D. B. G.; Thomlinson, I. A.; Broomfield-Tagg, R.; Lowe, J. P.; Lyall, C. L.; Hintermair, U. Engineering aspects of FlowNMR spectroscopy setups for online analysis of solution-phase processes. *React. Chem. Eng.* **2021**, *6* (9), 1548–1573.

(10) Bara-Estaun, A.; Harder, M. C.; Lyall, C. L.; Lowe, J. P.; Suturina, E.; Hintermair, U. Paramagnetic Relaxation Agents for Enhancing Temporal Resolution and Sensitivity in Multinuclear FlowNMR Spectroscopy. *Chem. Eur. J.* **2023**, *29* (38), e202300215.

(11) Hall, A. M. R.; Chouler, J. C.; Codina, A.; Gierth, P. T.; Lowe, J. P.; Hintermair, U. Practical aspects of real-time reaction monitoring using multi-nuclear high resolution FlowNMR spectroscopy. *Cat. Sci. Technol.* **2016**, *6* (24), 8406–8417.

(12) Gaussian 16, Revision C.01; Frisch, M. J.; Trucks, G. W.; Schlegel, H. B.; Scuseria, G. E.; Robb, M. A.; Cheeseman, J. R.; Scalmani, G.; V. Barone; Petersson, G. A.; Nakatsuji, H.; et al. Gaussian, Inc.: Wallingford CT, 2019.

(13) Andrae, D.; Häußermann, U.; Dolg, M.; Stoll, H.; Preuß, H. Energy-adjusted ab initio pseudopotentials for the second and third row transition elements. *Theor. Chim. Acta* **1990**, *77* (2), 123–141.

(14) Hariharan, P. C.; Pople, J. A. The influence of polarization functions on molecular orbital hydrogenation energies. *Theor. Chim. Acta* **1973**, *28* (3), 213–222.

(15) Hehre, W. J.; Ditchfield, R.; Pople, J. A. Self—Consistent Molecular Orbital Methods. XII. Further Extensions of Gaussian—Type Basis Sets for Use in Molecular Orbital Studies of Organic Molecules. *J. Chem. Phys.* **1972**, *56* (5), 2257–2261.

(16) Höllwarth, A.; Böhme, M.; Dapprich, S.; Ehlers, A. W.; Gobbi, A.; Jonas, V.; Köhler, K. F.; Stegmann, R.; Veldkamp, A.; Frenking, G. A set of d-polarization functions for pseudo-potential basis sets of the main group elements Al-Bi and f-type polarization functions for Zn, Cd, Hg. *Chem. Phys. Lett.* **1993**, *208* (3), 237–240.

(17) Becke, A. D. Density-functional exchange-energy approximation with correct asymptotic behavior. *Phys. Rev. A* **1988**, *38* (6), 3098–3100.

(18) Perdew, J. P. Density-functional approximation for the correlation energy of the inhomogeneous electron gas. *Phys. Rev. B* **1986**, *33* (12), 8822–8824.

(19) Weigend, F. Accurate Coulomb-fitting basis sets for H to Rn. *Phys. Chem. Chem. Phys.* **2006**, *8* (9), 1057–1065.

(20) Weigend, F.; Ahlrichs, R. Balanced basis sets of split valence, triple zeta valence and quadruple zeta valence quality for H to Rn: Design and assessment of accuracy. *Phys. Chem. Chem. Phys.* **2005**, *7* (18), 3297–3305.

(21) Tomasi, J.; Mennucci, B.; Cammi, R. Quantum Mechanical Continuum Solvation Models. *Chem. Rev.* ***2005**, 105* (8), 2999-3094.

(22) Grimme, S.; Antony, J.; Ehrlich, S.; Krieg, H. A consistent and accurate ab initio parametrization of density functional dispersion correction (DFT-D) for the 94 elements H-Pu. *J. Chem. Phys.* **2010**, *132* (15), 154104.

(23) Grimme, S.; Ehrlich, S.; Goerigk, L. Effect of the damping function in dispersion corrected density functional theory. *J. Comput. Chem.* **2011**, *32* (7), 1456–1465.

(24) Johnson, E. R.; Becke, A. D. A post-Hartree–Fock model of intermolecular interactions. *J. Chem. Phys.* **2005**, *123* (2).

(25) Spangenberg, A.; Kovalenko, O. O.; Ahlquist, M. S. G.; Wendt, O. F. Electron-Poor Iridium Pincer Complexes as Dehydrogenation Catalysts: Investigations into Deactivation through Formation of N2, CO, and Hydride Complexes. *Organometallics* **2024**, *43* (24), 3242-3250.

(26) Perdew, J. P.; Burke, K.; Ernzerhof, M. Generalized Gradient Approximation Made Simple. *Phys. Rev. Lett.* **1996**, *77* (18), 3865–3868.

(27) Perdew, J. P.; Burke, K.; Ernzerhof, M. Generalized Gradient Approximation Made Simple [Phys. Rev. Lett. 77, 3865 (1996)]. *Phys. Rev. Lett.* **1997**, *78* (7), 1396–1396.

(28) Lee, C.; Yang, W.; Parr, R. G. Development of the Colle-Salvetti correlation-energy formula into a functional of the electron density. *Phys. Rev. B* **1988**, *37* (2), 785–789.

(29) Tao, J.; Perdew, J. P.; Staroverov, V. N.; Scuseria, G. E. Climbing the Density Functional Ladder: Nonempirical Meta--Generalized Gradient Approximation Designed for Molecules and Solids. *Phys. Rev. Lett.* **2003**, *91* (14), 146401.

(30) Becke, A. D. Density-functional thermochemistry. III. The role of exact exchange. *J. Chem. Phys.* **1993**, *98* (7), 5648–5652.

(31) Stephens, P. J.; Devlin, F. J.; Chabalowski, C. F.; Frisch, M. J. Ab Initio Calculation of Vibrational Absorption and Circular Dichroism Spectra Using Density Functional Force Fields. *J. Phys. Chem.* **1994**, *98* (45), 11623–11627.

(32) Perdew, J. P.; Chevary, J. A.; Vosko, S. H.; Jackson, K. A.; Pederson, M. R.; Singh, D. J.; Fiolhais, C. Atoms, molecules, solids, and surfaces: Applications of the generalized gradient approximation for exchange and correlation. *Phys. Rev. B* **1992**, *46* (11), 6671–6687.

(33) Perdew, J. P.; Chevary, J. A.; Vosko, S. H.; Jackson, K. A.; Pederson, M. R.; Singh, D. J.; Fiolhais, C. Erratum: Atoms, molecules, solids, and surfaces: Applications of the generalized gradient approximation for exchange and correlation. *Phys. Rev. B* **1993**, *48* (7), 4978–4978.

(34) Adamo, C.; Barone, V. Toward reliable density functional methods without adjustable parameters: The PBE0 model. *J. Chem. Phys.* **1999**, *110* (13), 6158–6170.

(35) Zhao, Y.; Truhlar, D. G. A new local density functional for main-group thermochemistry, transition metal bonding, thermochemical kinetics, and noncovalent interactions. *J. Chem. Phys.* **2006**, *125* (19), 194101.

(36) Zhao, Y.; Truhlar, D. G. The M06 suite of density functionals for main group thermochemistry, thermochemical kinetics, noncovalent interactions, excited states, and transition elements: two new functionals and systematic testing of four M06-class functionals and 12 other functionals. *Theor. Chem. Acc.* **2008**, *120* (1), 215–241.

(37) Grimme, S. Semiempirical GGA-type density functional constructed with a long-range dispersion correction. *J. Comput. Chem.* **2006**, *27* (15), 1787–1799.

(38) Chai, J.-D.; Head-Gordon, M. Long-range corrected hybrid density functionals with damped atom–atom dispersion corrections. *Phys. Chem. Chem. Phys.* **2008**, *10* (44), 6615–6620.

(39) Paul, A.; Musgrave, C. B. Catalyzed Dehydrogenation of Ammonia–Borane by Iridium Dihydrogen Pincer Complex Differs from Ethane Dehydrogenation. *Angew. Chem. Int. Ed.* **2007**, *46* (43), 8153–8156.

(40) Cosier, J.; Glazer, A. M. A nitrogen-gas-stream cryostat for general X-ray diffraction studies. *J. Appl. Crystallogr.* **1986**, *19* (2), 105–107.

(40) Sheldrick, G. M. SHELXT - integrated space-group and crystal-structure determination. *Acta Crystallogr. A*, **2015**, *71*, 3–8.

(41) Sheldrick, G. M. A short history of SHELX. *Acta Crystallogr. A* **2008**, *64*, 112–122.

(42) Dolomanov, O. V.; Bourhis, L. J.; Gildea, R. J.; Howard, J. A. K.; Puschmann, H. OLEX2: a complete structure solution, refinement and analysis program. *J. Appl. Crystallogr.* **2009**, *42* (2), 339–341.
